# Unconventional chalcogen-containing azolylidene metal complexes as potential anticancer therapeutics

**DOI:** 10.1039/d5sc05555e

**Published:** 2025-12-29

**Authors:** Jan Romano-deGea, Irina L. Sinenko, Peter M. F. Pânzar, Adriana Neves Vieira, Lindsey E. K. Frederiksen, Kseniya Glinkina, Farzaneh Fadaei-Tirani, Rosario Scopelliti, Fabien Kuttler, Kelvin Lau, Paul J. Dyson

**Affiliations:** a Institute of Chemical Sciences and Engineering, École Polytechnique Fédérale de Lausanne (EPFL) 1015 Lausanne Switzerland jan.romanodegea@epfl.ch paul.dyson@epfl.ch; b Biomolecular Screening Facility, École Polytechnique Fédérale de Lausanne (EPFL) 1015 Lausanne Switzerland; c Protein Production and Structure Core Facility, École Polytechnique Fédérale de Lausanne (EPFL) 1015 Lausanne Switzerland

## Abstract

Organometallic compounds with N-heterocyclic carbene (NHC) ligands have been studied for their anticancer and antimicrobial properties, with imidazole and benzimidazole derivatives being the predominant scaffolds for potential NHC-containing drugs. In contrast, chalcogen-containing azolylidene ligands, (N,Y)HCs (Y = O, S, Se), remain largely unexplored in both medicinal inorganic chemistry and, more generally, in inorganic chemistry. Consequently, to study the effect of the incorporation of a chalcogen atom in the ligand, classical (N,N)HC complexes of platinum, gold and ruthenium were selected based on their previously reported biological activity and proposed mechanisms of action, and their (N,Y)HC (Y = O, S, Se) analogues were synthesised. The electronic and steric properties of the ligands and complexes were explored and their biological activity was evaluated. The introduction of a chalcogen atom within the heterocyclic scaffold of the ligands was found to modulate their interaction with biomolecules and regulate the cytotoxicity of the metal complexes towards ovarian cancer cells.

## Abstract 2

Substituting a nitrogen for a chalcogen in N-heterocyclic carbenes alters the electronic and steric features of the resulting metal complexes, influencing their biological activity and creating new opportunities for metallocarbene drug candidates.

## Graphical abstract

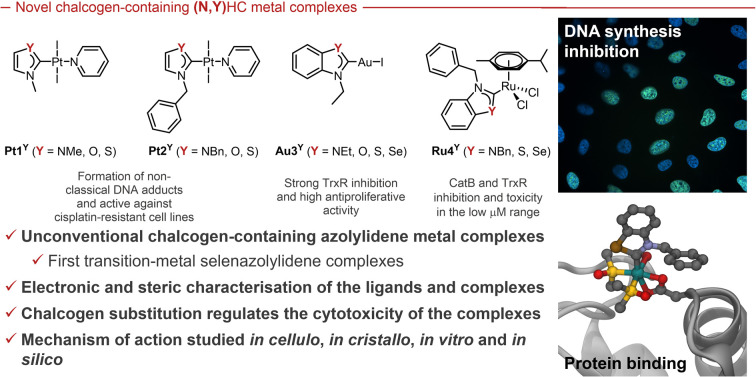

## Introduction

N-heterocyclic carbene (NHC) metal complexes display remarkable stability and tuneability, which explains their widespread use in many areas of chemistry.^1,2^ In recent years, NHC complexes, in particular platinum, gold, and ruthenium compounds, have been evaluated as anticancer, antimicrobial, antiviral and antiparasitic agents.^3–7^

Platinum-NHC complexes display cytotoxic effects comparable, or superior, to cisplatin against a variety of cancer cell lines.^8^ Traditionally, the mechanism of action (MoA) of platinum-based anticancer compounds has been related to their ability to bind the minor groove of DNA to then form 1,2-intrastrand crosslinks between nucleobases, blocking the translation and replication of DNA.^4^ In contrast, due to geometric constraints, *trans*-(NHC)PtX_2_(amine) complexes presumably form long-range DNA intra- and inter-strand adducts.^9,10^ These alternative crosslinks are less likely to be recognised as defects by repair proteins in cisplatin-resistant tumours.^11^ Therefore, such complexes are more likely to be active against cisplatin-resistant cell lines.^12^

In comparison, gold-NHC complexes are reported to inhibit proteins, such as thioredoxin reductase (TrxR), an enzyme overexpressed in some solid tumours.^13,14^ TrxR inhibition is associated with inhibition of mitochondrial respiration, potentially inducing apoptosis *via* mitochondria-mediated pathways.^15,16^ Furthermore, gold-NHC complexes tend to display high antiproliferative activity.^4,13,17^

Ruthenium-NHC complexes have been reported as inhibitors of cysteine- and selenocysteine-containing biomolecules, including TrxR and cathepsin B (CatB).^18^ The latter is a cysteine protease for which elevated expression levels are often associated with the progression of various tumours.^19^ Additionally, (*p*-cymene)(NHC)RuCl_2_ complexes act as antiproliferative agents, with IC_50_ values frequently in the low micromolar range.^20,21^

The modulation of the biological properties of metal NHC complexes is usually achieved through structural modifications introduced on the nitrogen atoms, also known as wingtips, or through the substituents on the heterocyclic backbone.^4,5^ Other carbene ligand classes such as triazoles and cyclic(alkylamino)carbenes (cAACs) have also been employed as scaffolds in medicinal inorganic chemistry.^22,23^ In contrast, metal complexes with chalcogen-containing azolylidene ligands, (N,Y)HCs (Y = O, S, Se), are rare,^24^ and studies of their biological properties are very scarce.^25,26^ In particular, only a single selenium-containing carbene metal complex has been previously reported.^27^ The effect on the biological activity of substituting the nitrogen atom in (N,N)HC ligands by a chalcogen atom remains, to the best of our knowledge, unexplored. (N,Y)HC (Y = O, S, Se) ligands present different steric and electronic properties compared to their classical nitrogen-containing counterparts. The chalcogen atoms are not alkylated, and hence they are more exposed than nitrogen atoms in (N,N)HCs, resulting in the characteristic “missing-wingtip” shape of these ligands.^28^ The metal atoms are also more exposed, albeit to a lesser extent. Besides modulating the electron donating abilities, the chalcogen atoms affect the aromaticity of the heterocycles.^29^ The chalcogen atoms in the azolylidene ligands have lone pairs that can act as acceptors in hydrogen bonds (HBs).^29–31^ Additionally, sulphur- and selenium-containing molecules can form intra- and intermolecular chalcogen bonds (ChBs).^31^ The presence of these interactions has ramifications in a wide range of fields and applications, including catalysis and biology, particularly in substrate and ligand–protein binding.^32–36^

**Fig. 1:**
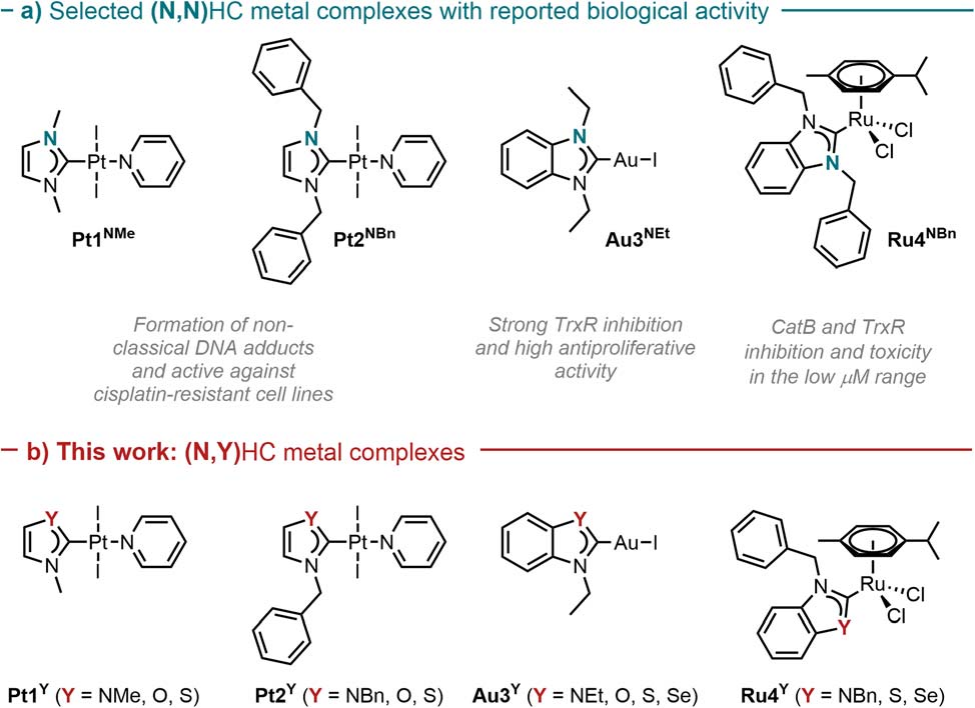
Selected parent (N,N)HC metal complexes (a) and their azolylidene (N,Y)HC (Y = NR, S, O, Se) analogues (b).

To explore the effect of the introduction of a chalcogen atom to the cytotoxicity and to evaluate structure–activity relationships (SAR) in unconventional chalcogen azolylidene metal complexes, four parent (N,N)HC metal complexes were selected based on reported examples in the literature demonstrating considerable cytotoxic effects and with a hypothesis on the mechanism of action (Fig. 1a): two *trans*-(NHC)PtI_2_(amine) complexes bearing non-fused 1,3-dimethylimidazolylidene or 1,3-dibenzylimidazolylidene ligands (Pt1^NMe^ and Pt2^NBn^) capable of overcoming cisplatin-acquired resistance;^12^ a highly antiproliferative TrxR inhibitor gold(i) iodido complex bearing a benzoannulated 1,3-diethylbenzimidazolylidene ligand (Au3^NEt^);^37^ and a ruthenium cymene complex bearing a fused 1,3-dibenzylbenzimidazolylidene ligand (Ru4^NBn^) with TrxR and CatB inhibition properties.^38^ Their azolylidene analogues were successfully synthesised (Fig. 1b) and their biological behaviour was evaluated.

## Results and discussion

### Synthesis and characterisation

The non-fused and benzoannulated azolium (N,Y)HC proligands, 1^Y^·HX–4^Y^·HX (Y = NR, O, S or Se; X = halide), were prepared in one step by heating the appropriate azole in acetonitrile (or neat) with the corresponding alkyl or benzyl halide to obtain the desired products in good to excellent yields. The azolium salts are hygroscopic and, in some cases, deliquescent. Therefore, special care should be taken with their isolation, purification and storage. The yields of the oxazolium salts are consistently lower than the others due to their tendency to ring open when exposed to heat, moisture or basic conditions.^39^ The corresponding (N,Y)HC metal complexes were synthesised using either silver transmetalation or a weak base as described previously.^1,24,40,41^ All compounds were characterised by ^1^H and ^13^C NMR spectroscopy and high-resolution mass spectrometry (HRMS).

### Platinum (N,Y)HC complexes

**Scheme 1:**
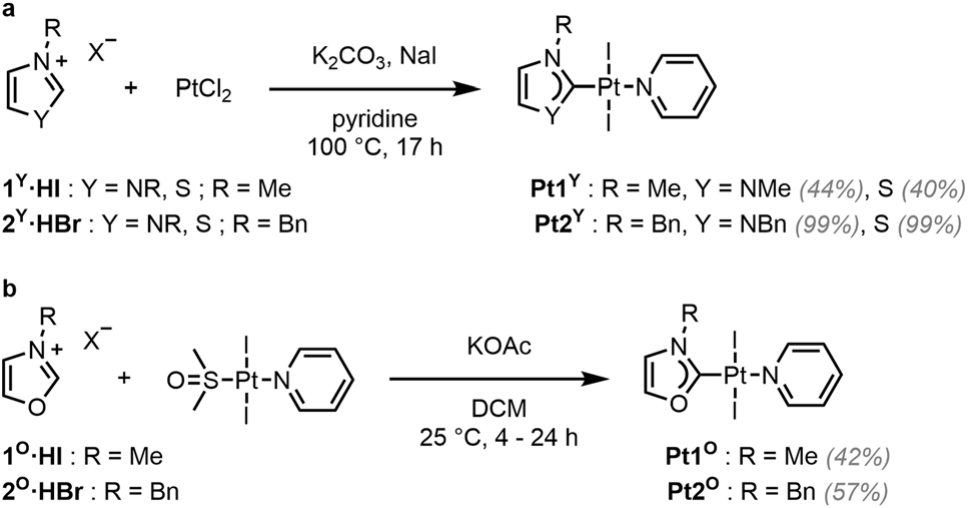
Synthesis of Pt1^Y^ and Pt2^Y^ (Y = NR, S) (a) and Pt1^O^ and Pt2^O^ (b).

Complexes Pt1^Y^ (Y = NMe, S) and Pt2^Y^ (Y = NBn, S) were synthesised in one step using a slightly modified literature procedure (Scheme 1a).^42^ The respective proligands (1^Y^·HI and 2^Y^·HBr), PtCl_2_, K_2_CO_3_ (the base required to deprotonate the carbene precursors), and NaI (as the iodide source) were heated under reflux in pyridine (acting as both the solvent and ligand). When applying these conditions to the synthesis of Pt1^O^ and Pt2^O^, only *trans*-[Pt(py)_2_I_2_] was isolated from the mixtures, and ring opening of the oxazolium salts was observed by ^1^H NMR spectroscopy.^39^ Instead, in order to obtain Pt1^O^ and Pt2^O^, *trans*-[Pt(pyridine)(dmso)I_2_] was reacted in DCM with the corresponding oxazolium salt in the presence of potassium acetate (KOAc), a weaker base, to yield the desired products in acceptable yields (Scheme 1b).

The desymmetrisation of the (N,Y)HC ligand by the introduction of the chalcogen atom is apparent in the ^1^H NMR spectra of Pt1^Y^ and of Pt2^Y^ (Y = NR, O, S). One signal is present for the protons in the heterocyclic backbone in Pt1^NMe^ and Pt2^NBn^, whereas two distinct signals are observed in Pt1^Y^ and Pt2^Y^ (Y = O, S) (SI Fig. 1). No major differences were observed in the ^1^H NMR spectra for the peaks corresponding to the pyridine ligand. The ^195^Pt NMR chemical shift of Pt1^Y^ and Pt2^Y^ (Y = NR, O, S) ranges between −4100 and −4400 ppm, in keeping with previous reports on platinum(ii) (N,N)HC complexes.^43^ The introduction of the chalcogen atom into the carbene ligands leads to a slight upfield shift of the ^195^Pt NMR signals of Pt1^O^ and Pt2^O^ compared to Pt1^NMe^ and Pt2^NBn^, whereas a considerable downfield shift is observed for Pt1^S^ and Pt2^S^. The upfield shift is indicative of a more electron-rich metal centre, due to a more electron-donating and less π-accepting ligand.^44,45^ Therefore, it could be expected that the carbene ligands in Pt1^O^ and Pt2^O^ form weaker bonds with the Pt(ii) centre.

**Fig. 2:**
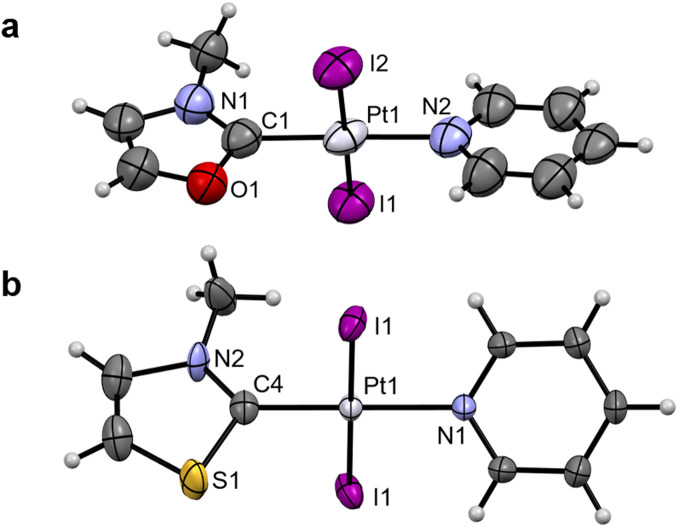
Single-crystal XRD structures of Pt1^O^ (a) and Pt1^S^ (b). Thermal ellipsoids are drawn with a 50% probability. Symmetry elements have been omitted for clarity. The structures of Pt1^NMe^ and Pt2^NBn^ can be found in SI Fig. 4.

Crystals suitable for single-crystal X-ray diffraction (scXRD) were grown for Pt1^NMe^, Pt1^O^, Pt1^S^ and Pt2^NBn^ (see Fig. 2 and SI Fig. 4a and b). The structures confirmed the presence of the coordinated (N,Y)HC ligands and of the expected *trans*-configuration. In Pt1^Y^ (Y = NMe, O, S), the Pt–C_NYHC_ bond length decreases from Pt1^O^ to Pt1^NMe^ and then to Pt1^S^ (Table 1), consistent with the ^195^Pt NMR chemical shift. Additionally, the (N,Y)HC ligands in Pt1^O^ and Pt1^S^ are slightly tilted with respect to the platinum atom (172.2° and 174.5°, respectively), deviating from the ideal “linear” structure observed in Pt1^NMe^ and Pt2^NBn^ (180°). Presumably, the elongated Y–C_NYHC_ (Y = O, S) bond results in the distorted geometries. All other bonds and angles are within the expected ranges.

**Table 1:** Selected bond lengths (Å) and angles (°) for Pt1^NMe^, Pt1^O^, and Pt1^S^

	Pt1^NMe^	Pt1^O^	Pt1^S^
Pt–C_NYHC_	1.961(5)	2.079(18)	1.948(7)
Pt–N_Pyr_	2.082(4)	1.960(18)	2.071(5)
Pt–I_Avg_	2.5935(3)	2.5953(21)	2.5932(3)
NR–C_NYHC_	1.347(5)	1.37(3)	1.251(11)
Y–C_NYHC_	1.347(5)	1.31(3)	1.801(5)
C_NYHC_–Pt–N_Pyr_	180.0	178.07(7)	180.0
NR–C_NYHC_–Y	105.1(5)	107(2)	106.8(7)
NYHC centroid–C_NYHC_–Pt	180.0	172.2	174.5
NYHC–Pyr	35.7	11.9	22.2

### Gold (N,Y)HC complexes

**Scheme 2:**
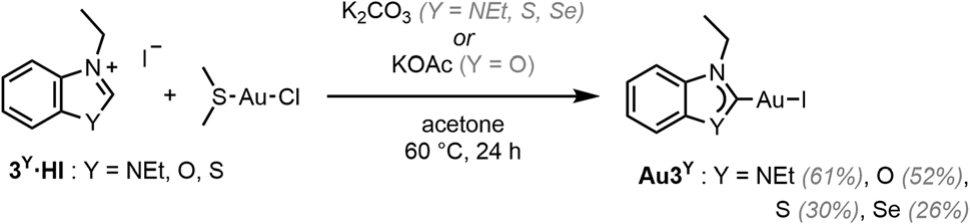
Synthesis of Au3^Y^ (Y = NEt, O, S, Se).

Au3^NEt^, Au3^S^ and Au3^Se^ were prepared from the reaction between the corresponding proligand, Au(SMe_2_)Cl, and K_2_CO_3_ in acetone at 60 °C (Scheme 2).^41^ A modified approach, using KOAc in place of K_2_CO_3_, was employed in the preparation of the oxazolylidene complex Au3^O^, to avoid the ring opening of the (N,O)HC ligand.

**Fig. 3:**
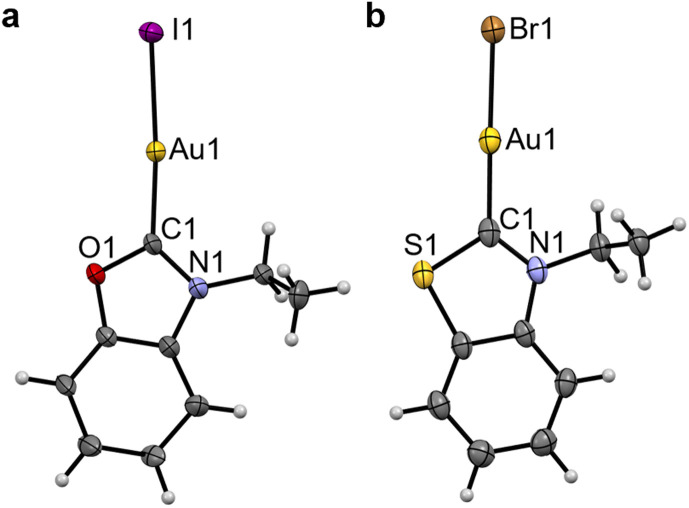
Single-crystal XRD structures of Au3^O^ (a) and Au3^S^ (X = Br) (b). Thermal ellipsoids are drawn with a 50% probability. The structure of Au3^NEt^ can be found in SI Fig. 4.

The benzothiazole peaks split in the ^1^H NMR spectra of Au3^O^ due to the desymmetrisation of the ligand, and become fully resolved in Au3^S^ and Au3^Se^ (SI Fig. 2). Additionally, the ethyl “wingtips” peaks shift downfield, indicating a more deshielded environment due to the introduction of the chalcogen atom or close proximity to the metal due to the lower steric hindrance. As observed in the platinum complexes, the Au–C_NYHC_ bond length in the single-crystal structures decreases from Au3^O^ to Au3^NEt^ and then to Au3^S^ (Fig. 3, Table 2, and SI Fig. 4c). Similarly, the (N,Y)HC ligands in Au3^O^ and Au3^S^ are slightly tilted with respect to the gold atom compared to the ideal structure in Au3^NEt^ (173.0° and 174.5°, respectively, *vs.* 180°). All other bonds and angles are within the expected ranges.

**Table 2:** Selected bond lengths (Å) and angles (°) for Au3^NEt^, Au3^O^, and Au3^S^

	Au3^NEt^	Au3^O^	Au3^S^ (X = Br)
Au–C_NYHC_	2.008(10)	2.05(4)	1.981(3)
Au–X	2.5449(8)	2.556(3)	2.4023(3)
NR–C_NYHC_	1.330(8)	1.33(5)	1.331(4)
Y–C_NYHC_	1.330(8)	1.35(5)	1.718(3)
C_NYHC_–Au–X	180.0	174.7(11)	176.45(8)
NR–C_NYHC_–Y	107.8(8)	110(3)	110.4(2)
NYHC centroid–C_NYHC_–Au	180.0	173.0	174.5

### Ruthenium (N,Y)HC complexes

**Scheme 3:**
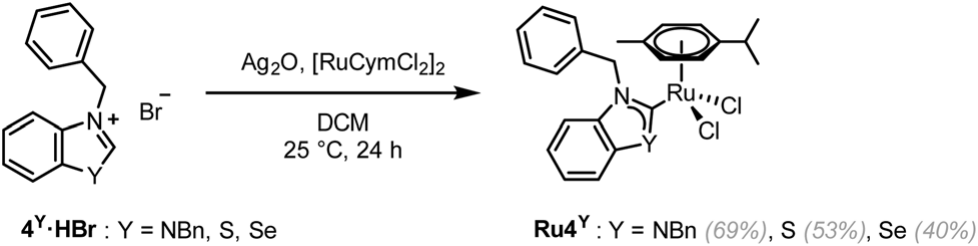
Synthesis of Ru4^Y^ (Y = NBn, S, Se).

Ru4^NBn^ was synthesised in two steps using a transmetalation route from a silver-(N,N)HC intermediate.^46^ As the isolation of the silver-(N,S)HC and silver-(N,Se)HC intermediate was unsuccessful, a previously reported one-pot route was employed to obtain Ru4^S^ and Ru4^Se^ (Scheme 3).^47^ Unfortunately, the synthesis of Ru4^O^ was unsuccessful both *via* the one-step or two-step transmetalation routes, as well as by using the weak base (KOAc) route.^41^ A similar approach to the synthesis of Pt1^O^ or Pt2^O^ was also attempted, using KOAc and a ruthenium complex bearing a labile ligand, (η^6^-*p*-cymene)RuCl_2_(DMSO). However, the (N,O)HC ruthenium complex was not isolated.

**Fig. 4:**
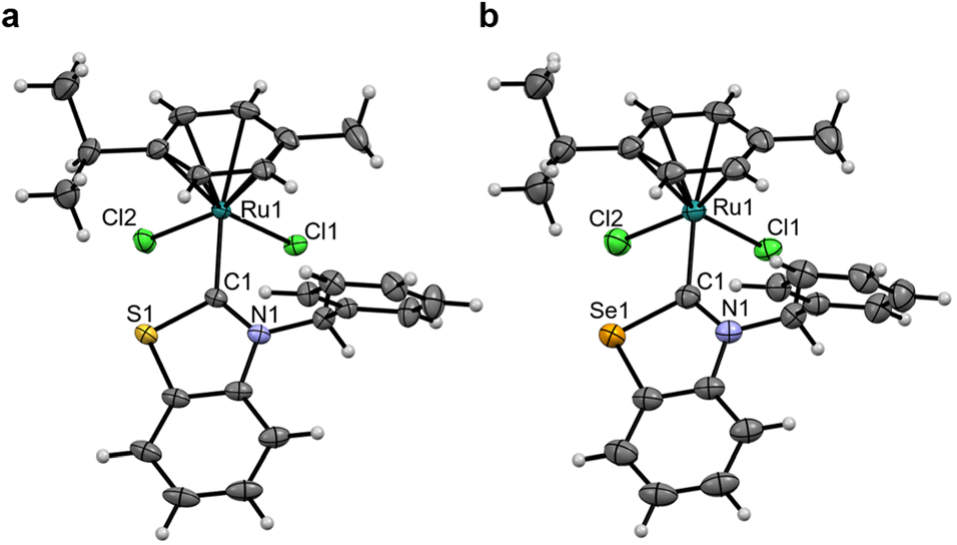
Single-crystal XRD structures of Ru4^S^ (a), and Ru4^Se^ (b). Thermal ellipsoids are drawn with a 50% probability. Solvates have been omitted for clarity. The structure of Ru4^NBn^ can be found in SI Fig. 4.

The rotation of the Ru–C_NYHC_ bond is restricted in Ru4^NBn^, with the benzylic N–CH_2_–Ph ^1^H NMR peaks appearing as two coupling doublets (at 5.84 and 6.56 ppm). In contrast, these peaks converge into singlets in Ru4^S^ and Ru4^Se^ (at 6.36 and 6.37 ppm, respectively), suggesting that the carbene is able to freely rotate (SI Fig. 3). Single-crystals of Ru4^Y^ (Y = NBn, S, Se) suitable for scXRD were grown (Fig. 4, Table 3 and SI Fig. 4d). To the best of our knowledge, the structure of Ru4^Se^ is the first example of a selenazolylidene complex to be reported. Only one other selenium-containing carbene complex has been previously reported, *i.e.* a chromium(0) (arylseleno)(diethylamino)carbene Fischer complex.^27^Ru4^Se^ features a central Ru(ii) ion in the typical distorted pseudo-octahedral geometry of piano-stool complexes also exhibited by Ru4^NBn^ and Ru4^S^. The ruthenium atom is coordinated by two chlorido ligands, an η^6^-bound cymene ligand and 4^Se^, a selenium-containing (N,Se)HC ligand. The C_NYHC_–Se distance is 1.875(3) Å and the N–C_NYHC_–Y angle is 109.5(2)°. All other bonds and angles are within range of the values previously reported in Ru(ii)-arene NHC complexes. The crystal packing is stabilised by π–π stacking of adjacent benzoselenazolylidene rings. Close Cl⋯H contacts may contribute to the packing stability, and a close contact between the selenium atom and the oxygen atom in THF is also observed (*d*_Se–O_ = 3.157 Å and *θ*_C–Se–O_ = 162.6°), constituting evidence of a ChB.^48^ The Ru–C_NYHC_ bond length decreases from 2.097(7) Å in Ru4^NBn^ to 2.038(2) and 2.032(2) Å in Ru4^S^ and Ru4^Se^, respectively, indicating that the interaction between the metal and the (N,Y)HC ligands might be stronger.

**Table 3:** Selected bond lengths (Å) and angles (°) for Ru4^NBn^, Ru4^S^, and Ru4^Se^

	Ru4^NBn^	Ru4^S^	Ru4^Se^
Ru–C_NYHC_	2.097(7)	2.038(2)	2.032(2)
Ru–Arene	1.699	1.702	1.705
Ru–Cl_avg_	2.422(2)	2.412(9)	2.413(1)
NR–C_NYHC_	1.359(8)	1.346(3)	1.338(3)
Y–C_NYHC_	1.390(9)	1.725(2)	1.875(3)
C_NYHC_–Ru–Arene	126.3	129.1	129.4
Cl_1_–Ru–Cl_2_	84.32(6)	86.48(2)	86.89(2)
NR–C_NYHC_–Y	105.6(5)	109.2(2)	109.5(2)
N–CH_2_–Phenyl	86.6 ± 0.5	79.1	79.5
NYHC centroid–C_NYHC_–Ru	178.5	176.2	175.0

### Electronic and steric analysis of the ligands and complexes

**Fig. 5:**
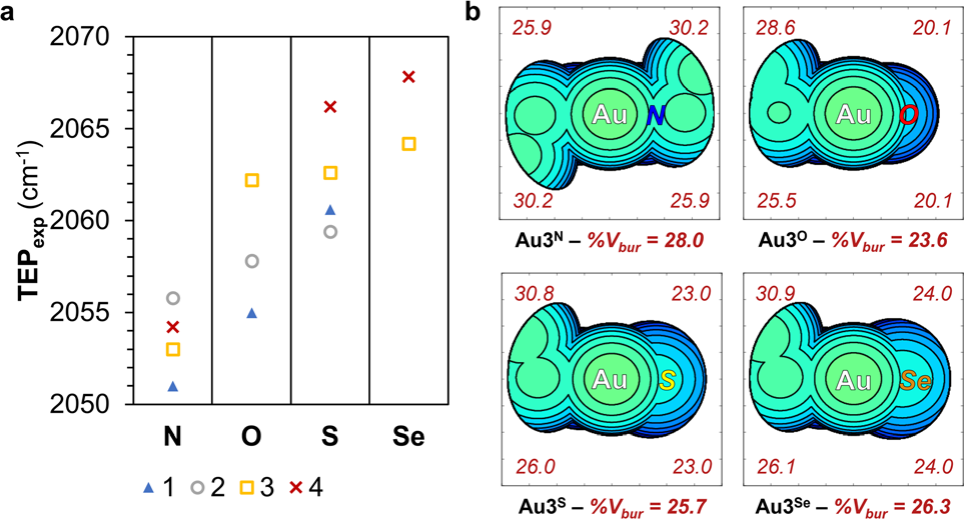
TEP_exp_ of carbene ligands 1^Y^–4^Y^ (Y = NR, O, S, Se) (a) and steric map of Au3^Y^ (Y = NEt, O, S, Se) showing total and per quadrant buried volume (%*V*_bur_) (b).

The electronic and steric properties of 1^Y^–4^Y^ (Y = NR, O, S, Se) were evaluated.^49^ The net electronic influence was assessed using Tolman electronic parameter (TEP) values, further separated into the σ-donating ability and π-accepting contributions by analysing the one-bond coupling constant, ^1^*J*_CH_, of the carbene carbon atom in the ^1^H NMR of the azolium salts and the ^77^Se NMR chemical shift, *δ*_Se_, of the selenium adducts.^50^ Additionally, DFT calculations were performed to support the experimental findings.^51^ Experimental TEP (TEP_exp_) were determined from the CO stretching frequencies of Rh[(N,Y)HC](CO)_2_Cl, Rh1^Y^–Rh4^Y^ (Fig. 5a), with the donor ability of the carbene atom following the trend, N ≫ O > S > Se. The σ-donor strength was extracted from the one-bond coupling constant, ^1^*J*_CH_, of the carbene atom of 1^Y^·HX–4^Y^·HX (Y = NR, O, S or Se; X = halide) in *d*_6_-DMSO.^52,53^ The extent of σ-donation of the ligands is slightly lower in (N,O)HCs than in classical (N,N)HCs, whereas (N,S)HCs and (N,Se)HCs are slightly stronger σ-donors (SI Fig. 5a). The π-backbonding properties were assessed using a method based on the ^77^Se NMR chemical shift, *δ*_Se_, of the selenium adducts Se1^Y^–Se4^Y^ (Y = NR, O, S, Se).^54^ Based on the magnitude of *δ*_Se_, the π-accepting properties of the carbene ligands increases according to the following sequence N < O < S < Se (SI Fig. 5b). Calculated TEP values (TEP_comp_), obtained from the analysis of the molecular electrostatic potential surface as reported previously, are in agreement with the trend observed from the experimental data (SI Fig. 6a).^55^ The HOMO and LUMO energy of 1^Y^–4^Y^ (Y = NR, O, S, Se) correlate well with the experimentally measured σ-donating and π-accepting character of the ligands (SI Fig. 6b).^56^ The electron occupancy in the M–C_NYHC_ bond decreases in the order (N,O)HC > (N,N)HC > (N,Se)HC > (N,S)HC (SI Table 8), in agreement with the ^195^Pt NMR chemical shifts and crystallographically determined bond lengths.

The study of the electrostatic surface of the optimised 4^Y^ (Y = O, S, Se) structures reveals the presence of a highly polarised p-orbital shaped lone pair on the chalcogen atom and perpendicular to the ring (SI Fig. 7a). These lone pairs, which become more diffuse when descending Group 16, could engage in HB interactions. Furthermore, sigma-holes, which are directly related to the ChB ability of the molecules, are observed in 4^S^ and 4^Se^ (SI Fig. 7b and SI Table 6).^31^ The potential to form ChB is evidenced by the Se–O close contact present in the crystal structure of Ru4^Se^, which adheres to the crystallographic definition of a ChB.^57,58^

Complementary to the electronic characterisation, the steric effects of the ligands were evaluated. The percentage of buried volume (*V*_bur_) around the metal centre was estimated from the experimentally determined or optimised structures of Pt1^Y^, Pt2^Y^, Au3^Y^, and Ru4^Y^ (Y = NR, O, S, Se) (Fig. 5b and SI Fig. 10).^59^ The total %*V*_bur_ decreases upon substitution of the alkylated nitrogen for a chalcogen, and then increases with the increasing atomic size of the Group 16 element, following the trend N ≈ Se < S < O. Furthermore, the %*V*_bur_ in the chalcogen-containing quadrants display less buried volume, highlighting that the coordination sphere surrounding the metal is less sterically crowded in the (N,Y)HCs (Y = O, S, Se) complexes.^28,60^

The steric and electronic analysis indicates that the unconventional azolylidene carbenes ligands, 1^Y^–4^Y^ (Y = NR, O, S, Se), are stronger π-acceptor ligands than classical (N,N)HCs with similar σ-donation ability (with the exception of the oxazolylidenes). Furthermore, they present a less congested binding sphere and the incorporation of chalcogen atoms potentially enables HB and ChB interactions.

### *In vitro* studies

The cytotoxicity of Pt1^Y^, Pt2^Y^, Au3^Y^, and Ru4^Y^ (Y = NR, O, S, Se) was evaluated against the A2780 ovarian cancer cell line, A2780cis cells with acquired resistance to cisplatin, and non-cancerous human embryonic kidney HEK293T cells (Table 4), using the PrestoBlue assay.^61^ FDA-approved drugs cisplatin and auranofin, and the experimental drug RAPTA-C were included as controls. Note that the cytotoxicity of proligands 1^Y^·HX–4^Y^·HX (Y = NR, O, S, Se; X = halide) is lower than that of the related complexes against the screened cell lines (SI Table 9). Complexes Pt1^Y^ and Pt2^Y^ (Y = NR, O, S) exhibit cytotoxicity values in the range of 0.6 to 30 µM against the ovarian cancer cell lines. Complexes based on the benzylazolylidene scaffold, Pt2^Y^ (Y = NBn, O, S), are more cytotoxic than the methylazolylidene analogues, Pt1^Y^ (Y = NMe, O, S), probably as a consequence of their higher lipophilicity or the presence of a benzyl moiety capable of intercalating DNA bases.^65,66^ In both these cell lines, the most cytotoxic complexes are Pt1^NMe^ and Pt2^NBn^ (≈3 µM and ≈1 µM, respectively). The toxicity of the complexes diminished when replacing the imidazole ring for an oxazole (Pt1^O^ and Pt2^O^, ≈10 µM and ≈5 µM), and further decreased when exchanging the oxygen atom for a sulphur atom (Pt1^S^ and Pt2^S^, ≈15 µM and ≈8 µM). Although less cytotoxic than cisplatin to the A2780 cell line, Pt1^Y^ and Pt2^Y^ (Y = NR, O, S) are able to overcome acquired cisplatin-resistance in the A2780cis cell line (resistance index, RI = 1.0–2.4 *vs.* 24.1 for cisplatin), indicating that they probably operate *via* a different MoA to cisplatin. In particular, Pt2^NBn^ presents comparable efficacy and selectivity for cancer cells to cisplatin, while being much more effective in the cisplatin-resistant cell line (1.2 ± 0.1 µM *vs.* 8.4 ± 4.6 µM).

**Table 4:** IC_50_ values of Pt1^Y^, Pt2^Y^, Au3^Y^, and Ru4^Y^ (Y = NR, O, S, Se), cisplatin, auranofin, and RAPTA-C in A2780, A2780cis and HEK293T cell lines after 72 h evaluated using the PrestoBlue assay.^61^ Resistance index (RI)^a^, and *n*-octanol/water partition coefficients (log*P*_OW_)

Compound	IC_50_ (µM) after 72 h			RI^a^	log*P*_OW_
	A2780	A2780cis	HEK293T		
Pt1^NMe^	2.9 ± 0.2	2.8 ± 0.7	4 ± 1	1.0	1.3 ± 0.6
Pt1^O^	8 ± 2	16 ± 4	18 ± 6	2.0	1.0 ± 0.5
Pt1^S^	13 ± 8	30 ± 29	47 ± 23	2.4	1.3 ± 1.1
Pt2^NBn^	0.6 ± 0.2	1.2 ± 0.1	1.4 ± 0.1	1.9	2.1 ± 0.8
Pt2^O^	4 ± 1	5 ± 2	6 ± 2	1.5	1.6 ± 0.2
Pt2^S^	6 ± 1	12 ± 3	9 ± 2	2.2	1.8 ± 1.0
Cisplatin	0.4 ± 0.1	8 ± 5	1.5 ± 0.2	24.1	−2.19 (ref. 62)
Au3^NEt^	0.6 ± 0.2	2.7 ± 0.1	2.1 ± 0.6	4.9	1.6 ± 1.1
Au3^O^	0.2 ± 0.2	5 ± 2	1.0 ± 0.1	31.5	1.2 ± 0.4
Au3^S^	0.2 ± 0.1	6.4 ± 0.4	1.9 ± 0.3	29.2	1.5 ± 0.1
Au3^Se^	0.1 ± 0.1	0.2 ± 0.1	0.1 ± 0.1	3.4	1.4 ± 0.3
Auranofin	0.1 ± 0.1	1.9 ± 0.4	0.4 ± 0.1	20.6	1.6 (ref. 63)
Ru4^NBn^	5.9 ± 1.0	12.8 ± 0.9	13 ± 2	2.2	2.7 ± 0.3
Ru4^S^	10 ± 1	26 ± 5	20 ± 3	2.7	2.2 ± 0.2
Ru4^Se^	3.8 ± 0.8	9 ± 3	5 ± 1	2.2	2.4 ± 0.7
RAPTA-C	>100 µM	>100 µM	>100 µM		−1.8 (ref. 64)

a Resistance index (RI) = (IC_50_ A2780cis/IC_50_ A2780).

The gold complexes, Au3^Y^ (Y = NEt, O, S, Se) have IC_50_ values in all three cell lines ranging from 0.1 to 6.4 µM, with the complexes having different behaviour depending on the cell line. In the ovarian cancer A2780 cell line, Au3^Se^ (0.1 ± 0.1 µM), Au3^S^ (0.2 ± 0.1 µM) and Au3^O^ (0.2 ± 0.2 µM) exhibit cytotoxicity comparable to auranofin (0.1 ± 0.1 µM), whereas Au3^NEt^ (0.6 ± 0.2 µM) is less cytotoxic. In contrast, in the cisplatin-resistant cell line A2780cis, Au3^Se^ (0.2 ± 0.1 µM) has the lowest IC_50_ value, followed by Au3^NEt^, Au3^O^ and then Au3^S^. With the exception of Au3^NEt^ and Au3^Se^ (RI = 4.9 and 3.4), the complexes did not overcome acquired cisplatin resistance (RI = 20–31), indicating that the MoA of Au3^Y^ (Y = O, S) likely involves interactions with DNA (that would be more efficiently repaired in the cisplatin resistant cells and would result in lower cytotoxicity).

Compared to RAPTA-C, which is not cytotoxic *in vitro* (IC_50_ > 100 µM), but effective *in vivo*,^64,67^Ru4^Y^ (Y = NBn, S, Se) are considerably more cytotoxic, with IC_50_ values in a similar range to Pt1^Y^ (Y = NMe, O, S), *i.e.* 0.6 to 30 µM in the three cell lines. Ru4^Se^ is the most cytotoxic compound of the series in all three cell lines, which was also observed in Au3^Se^. Additionally, all the complexes are active against cisplatin-resistant cells (RI = 2.2–2.7).

The lipophilicity of the complexes Pt1^Y^, Pt2^Y^, Au3^Y^, and Ru4^Y^ (Y = NR, O, S, Se) decreases with the introduction of the chalcogen atom (Table 4 and SI Table 6), with the (N,O)HCs complexes exhibiting the highest hydrophilic character, likely due to the formation of HBs with water. Although the cytotoxicity of some NHC complexes has been previously linked with their lipophilicity,^68^ this does not appear to be the case for Pt1^Y^, Pt2^Y^, Au3^Y^, and Ru4^Y^ (Y = NR, O, S, Se). SAR analysis (based on the experimental and computational data from this study) was performed to identify major chemical, physical, structural and electronic properties modulating the cytotoxicity of Pt1^Y^, Pt2^Y^, Au3^Y^ and Ru4^Y^ (Y = NR, O, S, Se). Notably, typical factors such as lipophilicity or aromaticity do not appear to affect the cytotoxicity of the complexes to a great extent (SI Fig. S13). Instead, cytotoxicity appears to be correlated with the charge at the metal, the energy of the σ-hole and lone pair energy, or the Y–C_NYHC_ bond length, and inversely correlated with percentage of *V*_bur_ in the NR quadrant or the energy of the π-donor orbital. These properties are directly linked to the nature of the azolylidene ligand. Despite the potential interest in Pt1^Se^ and Pt2^Se^ given the high cytotoxicity exhibited by Au3^Se^ and Ru4^Se^, we did not synthesise them due to stability and accessibility challenges of unsubstituted selenazoles.^69^

The cytotoxicity of the platinum complexes, Pt1^NMe^ and Pt2^NBn^, is similar to other previously reported *trans*-(NHC)PtX_2_(amine) complexes (between 0.9 and 3.1 µM).^12^ Additionally, similar to the platinum compounds in this study, some of the related complexes reported also overcome acquired cisplatin-resistance. The cytotoxicity of Ru4^NBn^ in A2780 cells is also comparable to those reported in the literature (2.1 ± 0.9 µM and 2.4 ± 1.0 µM against MCF-7 breast adenocarcinoma and HT-29 colon carcinoma cells, respectively).^38^ In contrast, a 10-fold increase in the toxicity is observed in Au3^NEt^ compared to the same complex bearing a chlorido ligand instead of an iodido ligand (0.6 ± 0.2 µM *vs.* 6.4 ± 2.0 µM).^37^Au3^S^ demonstrated comparable activity to a previously-reported peptide-derivatised (N,S)HC gold complex in lung carcinoma (A549) (IC_50_ = 0.4 ± 0.01 µM).^26^ We were not able to find any reports of the cytotoxicity of (N,O)HC complexes or of (N,Se)HC complexes, which had remained unexplored until now.

### Mechanistic studies

As previously mentioned in the introduction, the parent (N,N)HC complexes, Pt1^NMe^, Pt2^NBn^, Au3^NEt^, and Ru4^NBn^, were selected as they had demonstrated anticancer activity and a MoA had been proposed. Interactions with DNA were proposed for Pt1^NMe^ and Pt2^NBn^,^12^ whereas Au3^NEt^ and Ru4^NBn^ were reported to interact with thioredoxin reductase (TrxR) and cathepsin B (CatB).^37,38^

**Fig. 6:**
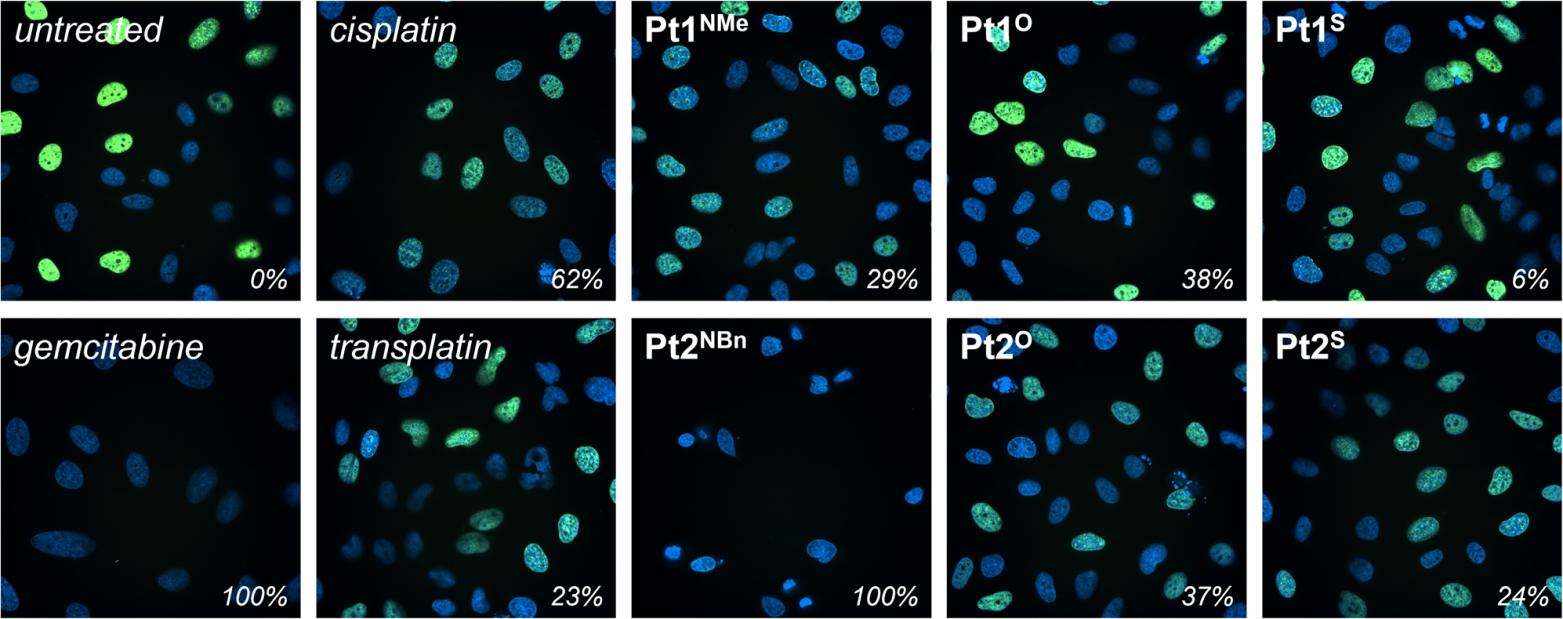
Fluorescence microscopy images of HELA cells treated with Pt1^Y^ and Pt2^Y^ (Y = NR, O, S), cisplatin, and transplatin (3 µM) and gemcitabine (1 µM) with the degree of inhibition of DNA synthesis indicated (bottom right). Cell nuclei were labelled with Hoechst 33 342 (blue), and nuclei undergoing DNA synthesis with Edu-AlexaFluor-488 (green). Gemcitabine and DMSO were used as a positive (100%) and negative (0%) control, respectively.

The inhibitory effect of Pt1^Y^ and Pt2^Y^ (Y = NR, O, S) on DNA synthesis during cell proliferation was quantified *in cellulo* using an EdU incorporation assay and fluorescence cell microscopy (Fig. 6 and SI Fig. 12).^70^ Gemcitabine, a clinically-approved DNA synthesis inhibitor,^71^ was used as a positive control (100% inhibition), and the cells treated with an equivalent amount of DMSO served as a negative control (“untreated”, 0% inhibition). Cisplatin and transplatin were included as references. DNA synthesis was blocked to various degrees in the cells treated with the different platinum complexes. As expected based on their structure and cytotoxicity, cisplatin was a better DNA synthesis inhibitor than transplatin (62% *vs.* 23%), and Pt1^Y^ (Y = NMe, O, S) displayed a lower degree of inhibition than Pt2^Y^ (Y = NBn, O, S), presumably as a consequence of the ability of the benzyl wingtip to intercalate DNA. In particular, Pt2^NBn^, the compound with the highest cytotoxicity, inhibited DNA synthesis more effectively than cisplatin under the tested conditions and reached comparable inhibition to the positive control (gemcitabine, 100%). Overall, the extent of inhibition of DNA synthesis correlates well with the cytotoxicity of the complexes, suggesting that the inhibition of DNA synthesis, likely by the formation of non-classical DNA adducts, is a key MoA of Pt1^Y^ and Pt2^Y^ (Y = NR, O, S).

**Fig. 7:**
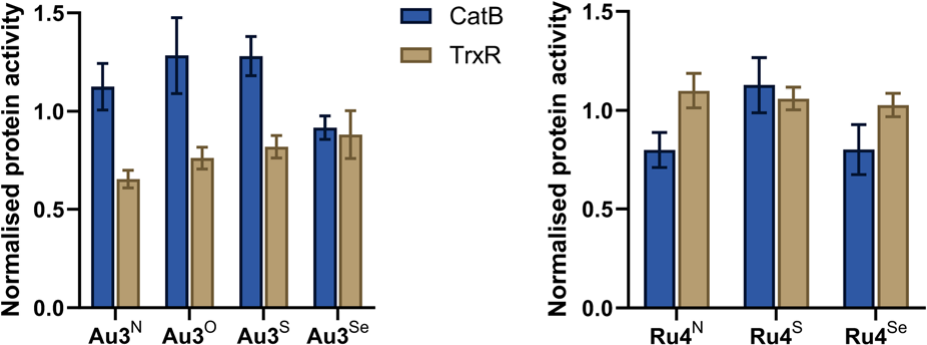
Inhibition of Cathepsin B (CatB) and Thioredoxin Reductase (TrxR) by Au3^Y^ (Y = NEt, O, S, Se) and Ru4^Y^ (Y = NBn, S, Se). 1 µM and 5 µM of Au3^Y^, and 10 µM and 5 µM of Ru4^Y^ were used for the TrxR and CatB assay, respectively. The untreated control was used to normalise enzyme activity.

The inhibitory activity of Au3^Y^ (Y = NEt, O, S, Se) and Ru4^Y^ (Y = NBn, S, Se) against CatB and TrxR was studied in A2780 cells using commercially available assays (Fig. 7). Au3^Y^ (Y = NEt, O, S, Se) inhibit the enzymatic activity of TrxR but are inactive against CatB. However, the degree of inhibition does not correlate with the cytotoxicity of the complexes, which further highlights that Au3^Y^ (Y = O, S, Se) may have other molecular targets. It is worth noting that other gold complexes, such as auranofin, have promiscuous multi-target activity.^72,73^ In contrast, Ru4^Y^ (Y = NBn, S, Se) are inactive for TrxR, whereas Ru4^Y^ (Y = NBn, Se) inhibit CatB activity, correlating well with their cytotoxicity and indicating that cathepsin B is a likely biological target of Ru4^Y^ (Y = NBn, Se), although other targets cannot be excluded.^74^

### Structural studies

**Fig. 8:**
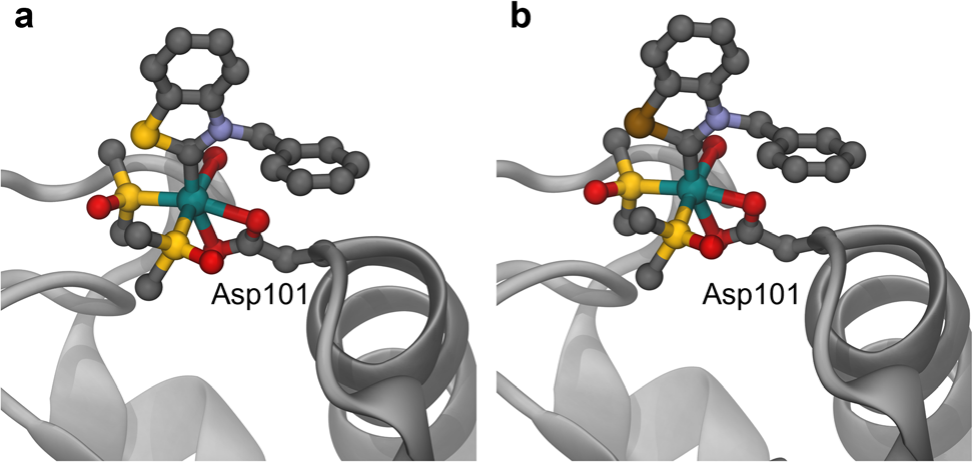
Binding sites of Ru4^S^ (a) and Ru4^Se^ (b) with HEWL (see SI for details).

Crystals of adducts between Ru4^Y^ (Y = S, Se) and hen egg white lysozyme (HEWL), a model protein, were obtained by crystal soaking and analysed by single-crystal X-ray diffraction (Fig. 8). Both crystals presented anomalous electron density around Asp101, where the ruthenium atoms and some of the Ru4^Y^ (Y = S, Se) ligands were modelled.

The ruthenium centre is coordinated in a bidentate fashion to the carboxylic acid of Asp101. The average Ru–O_Asp101_ bond length is 2.227 and 2.152 Å for Ru4^S^ and Ru4^Se^, respectively, indicating that Ru4^Se^ might bind the protein more strongly. It is conceivable that binding of Ru4^S^ to CatB is also weaker than that of Ru4^Se^, which might explain the weaker CatB inhibition exhibited by Ru4^S^ (Fig. 7), also supported by the electronic description of 4^S^ as a weaker π-acceptor. In both structures, the (N,Y)HC ligand remains coordinated to the metal centre, but the arene and chlorido ligands have been substituted by solvent molecules and by the oxygen atoms in Asp101. The Ru–C_NYHC_ distance in the protein crystals adducts is 1.987 and 1.990 Å for Ru4^S^ and Ru4^Se^, respectively, slightly shorter than in the intact Ru4^S^ and Ru4^Se^ structures (2.038(2) and 2.032(2) Å, respectively). In both crystallised adducts, the metal drug fragments have an occupancy of 1.0, and no further additional isomers were observed. A comparison of the ruthenated HEWL structures to the native protein crystals (PDB:4NHI) revealed no major structural perturbations (RMSD of 0.920 and 0.894 Å).

Presumably, the binding in Ru4^Y^ (Y = S, Se) is driven by the electrostatic interactions between the resulting cationic complex from the substitution of the chlorido ligands in Ru4^Y^ (Y = S, Se) and the negatively charged catalytic site of HEWL. It should be noted that the different soaking times (5–10 s *vs.* 3 days) could affect the binding sites. Other ruthenium complexes have been shown to interact with Asp101 in HEWL, however, only as naked ruthenium ions.^75,76^ Crystals of adducts between HEWL and dichloro(1,3-dimethylbenzimidazol-2-ylidene)(η^6^-*p*-cymene)ruthenium(ii), a complex related to Ru4^NBn^, revealed His15 and Lys33 as the preferred binding sites.^75^ Hence, replacement of the nitrogen atom for a chalcogen atom in Ru4^Y^ (Y = NBn, S, Se) modulates the preferential binding site of the ruthenium complexes, likely a consequence of the more electron-poor ruthenium centres. Previously, other (N,N)HC ruthenium complexes have been reported to bind to both nitrogen- and oxygen-containing residues in several proteins,^77–80^ as well as to interact with carboxylic acid-containing amino acid residues.^81^

In order to elucidate the molecular interaction between the complexes and their studied biological targets, molecular docking was employed. However, the prediction of the binding site of metal complexes is non-trivial due to the reactivity of metal complexes with respect to nucleophiles (for example, water, amino acids, or nucleobases) and the scarcity of force fields able to describe metal atoms. Several solutions have been reported or adapted to address this challenge.^82–85^ We developed and validated an approach to the docking of metal-containing compounds based on Autodock^86^ (see SI for the further details and discussion). The developed protocol yields reasonable redocking results in terms of the prediction of the metal binding site, and of the position, orientation, and conformation of the ligands.

Blind molecular docking (the whole biomolecule structure is used without any bias towards specific binding sites) was performed using the approach to evaluate potential binding sites and affinities of Pt1^Y^ and Pt2^Y^ (Y = NR, O, S) to DNA (from a simulated *trans*-bound DNA interstrand adduct),^87^ of Au3^Y^ (Y = NEt, O, S, Se) to TrxR (PDB:2J3N),^88^ and of Ru4^Y^ (Y = NBn, S, Se) to CatB (PDB:3AI8).^89^

Pt^1Y^ and Pt^2Y^ (Y = NR, O, S) formed N7-guanine interstrand DNA adducts (SI Fig. 17, 18 and SI Table 12). The binding energies for Pt2^Y^ (Y = NBn, O, S) were lower than for Pt1^Y^ (Y = NMe, O, S), consistent with the higher cytotoxicity and higher degree of inhibition of DNA synthesis (Fig. 6) exhibited by Pt2^Y^ (NBn, O, S), arising from the additional π–π interactions between the benzyl group and the nucleobases (SI Fig. 18). Both chalcogen atoms in Pt2^O^ and Pt2^S^ are positioned towards a hydrogen bond donor area around the amine group of an adjacent adenine, which likely establishes a HB and stabilises the conformation.

Despite predicting binding sites for Au3^Y^ (Y = NEt, O, S, Se) not typically linked to TrxR inhibition (Sec498),^14^ a HB was observed Au3^O^ between the oxygen atom in the oxazolylidene ligand and the amine of Lys67 (3.01 Å, SI Fig. 18). This was not observed in Au3^S^ or Au3^Se^, consistent with the expected strength of the HB (SI Table 4). In contrast, a ChB was present between the carbonyl oxygen in Thr58 and the chalcogen atom in Au3^S^ or Au3^Se^ (3.60 Å and 3.39 Å, SI Fig. 18). These results highlight that HB and ChB interactions are likely to occur in (N,O)HC, and in (N,S)HC and (N,Se)HC complexes, respectively.

Docking studies of Ru4^Y^ (Y = NBn, S, Se) to CatB indicated that the complexes are likely to form adducts with residues Cys29 and His199 simultaneously, which are part of the catalytic pocket and active site of the protein.^90^ Other ruthenium complexes have previously been reported to interact with Cys29.^18^ For Ru4^Se^, two different poses binding Cys29:His199 were observed (SI Fig. 19), which could explain the higher cytotoxicity of Ru4^Se^ (Fig. 7).

## Conclusions

Unconventional chalcogen-containing azolylidene (N,Y)HC (Y = NR, O, S, Se) metal complexes based on (benz)oxazole, (benzo)thiazole and benzoselenazole were successfully synthesised, including the first reported transition metal selenazolylidene complexes. The electronic and steric properties of these unconventional, chalcogen-based carbene ligands were investigated. The ligands were found to be overall weaker donors with enhanced π-acceptor ability, which could prove useful in the synthesis of low-valent or main group complexes and potentially favouring catalytic reactions benefiting from electron-deficient metal centres. The cytotoxicity of the (N,Y)HC complexes was evaluated against ovarian cancer cells. Distinct trends in cytotoxicity emerged, depending on the metal centre. In the platinum complexes, substitution of the alkylated nitrogen in the azolylidene ligand for a chalcogen atoms decreased cytotoxicity following the trend (N,N)HC > (N,O)HC > (N,S)HC. For gold and ruthenium, the (N,Se)HC complexes, being the only reported examples of this class, were more cytotoxic than, in order, the respective (N,O)HC, (N,N)HC, and (N,S)HC analogues, suggesting a new avenue for putative metallocarbene anticancer drug candidates. In many cases, the complexes were able to overcome cisplatin-related resistance. Mechanistic and structural studies were performed *in vitro*, *in cellulo*, *in crystallo*, and *in silico*, revealing that incorporation of a chalcogen atom into the heterocyclic scaffold can modulate biological targets, activity and biomolecular interactions of the complexes. These findings highlight the potential of unconventional chalcogen azolylidene ligands as tuneable scaffolds to fine-tune the biological activity of metal complexes.

## Author contributions

J. R.-dG. conceived the idea and led the project. J. R.-dG., P. M. F. P, A. N. V. and L. E. K. F. conducted the synthesis and characterisation of the proligands and metal complexes. I. L. S. and K. G. carried out the biological experiments. F. F.-T. and R. S. collected and processed the single-crystal X-ray data. F. K. performed the cell fluorescence microscopy measurements and K. G. solved the protein crystal structures. All authors contributed to the discussion of the results and revision of the manuscript. P. J. D. directed and supervised the project.

## Conflicts of interest

There are no conflicts to declare.

## Supplementary Material

SC-OLF-D5SC05555E-s001

SC-OLF-D5SC05555E-s002

## Data Availability

The data supporting this article has been included in the supplementary information (SI). See DOI: https://doi.org/10.1039/d5sc05555e. CCDC 2130715, 2270445–2270450, 2271662, 2395164 and 2395165 contain the supplementary crystallographic data for this paper.^91*a*–*j*^ Protein crystallographic data for the adducts of HEWL with Ru4^S^ and Ru4^Se^ has been deposited at the PDB under accession numbers 9HTI and 9HTJ, respectively.^92*a*,*b*^ Additional data generated in this study is openly available at https://doi.org/10.5281/zenodo.16159145.
