# Supplementary material for: Unconventional chalcogen-containing azolylidene metal complexes as potential anticancer therapeutics
Source: Chem Sci. 2025 Dec 29;17(8):4296–306. doi: 10.1039/d5sc05555e (PMC12794336; doi:10.1039/d5sc05555e)
Supplement: SC-017-D5SC05555E-s001 [file SC-017-D5SC05555E-s001.pdf]

This journal is © The Royal Society of Chemistry 2025

Supplementary Information

for

Unconventional chalcogen-containing azolylidene metal complexes as potential anticancer therapeutics

Jan Romano-deGea[a*], Irina L. Sinenko[a], Peter M. F. Pânzar[a], Adriana Neves Vieira[a], Lindsey E. K. Frederiksen[a], Kseniya Glinkina[a], Farzaneh Fadaei-Tirani[a], Rosario Scopelliti[a], Fabien Kuttler[b], Kelvin Lau[c], Paul J. Dyson[a*]

[a] Institute of Chemical Sciences and Engineering, École Polytechnique Fédérale de Lausanne (EPFL), 1015 Lausanne, Switzerland.

[b] Biomolecular Screening Facility, École Polytechnique Fédérale de Lausanne (EPFL), 1015 Lausanne, Switzerland.

[c] Protein Production and Structure Core Facility, École Polytechnique Fédérale de Lausanne (EPFL), 1015 Lausanne, Switzerland.

[*] Corresponding authors

E-mail addresses: jan.romanodegea@epfl.ch ; paul.dyson@epfl.ch

Table of contents

1. Materials and methods 3
2. Synthesis and characterisation 4
   2.1. Azolium (N,Y)HC proligands $1^{Y}\cdot HX$-$4^{Y}\cdot HX$ (Y = NR, O, S, Se ; X = halide) 4
   2.2. (N,Y)HC metal complexes $Pt1^{Y}$, $Pt2^{Y}$, $Au3^{Y}$ and $Ru4^{Y}$ (Y = NR, O, S, Se) 9
   2.3. Single crystal X-ray diffraction analysis 18
   2.4. Other metal precursors and complexes 24
   General Procedure for the synthesis of NYHC−Selenium complexes ($Se1^{Y}$−$Se4^{Y}$) (Y = NR, O, S, Se) 26
   General procedure for the synthesis of Rh(NYHC)$(CO)_2$Cl ($Rh1^{Y}$−$Rh4^{Y}$) (Y = NR, O, S, Se) 28
   2.5. Partition coefficients 29
3. Computational details 31
4. *In vitro* studies 44
   4.1. Cell culture 44
   4.2. Cytotoxicity studies 44
   4.3. Protein inhibition assays 45
   4.4. EdU incorporation assay 46
5. Protein crystallography 50
6. Molecular docking 53
7. NMR spectra 65
Supplementary references 104

1. Materials and methods

All chemicals were either of reagent or analytical grade and used as purchased from commercial sources without additional purification. $PtCl_2$ and $HAuCl_4$ were purchased from Sigma Aldrich. $RuCl_3{\cdot}3H_2O$ was obtained from Precious Metals Online. Other reagents and metal precursors were prepared and purified according to procedures available in the literature with some modifications. Reactions were performed under an inert atmosphere (N_2) using Schlenk techniques with solvents dried using a solvent purification system (Innovative Technologies Inc.) or dried over molecular sieves (3 Å).

NMR spectra were acquired at 298 K (unless otherwise stated) on a Bruker Avance 400 MHz spectrometer. Chemical shifts (δ) and coupling constants (J) are reported in ppm and Hz, respectively. The following abbreviations were used to designate multiplicities: s = singlet, d = doublet, t = triplet, q = quartet, quint = quintuplet, sept = septuplet, hept = heptuplet, m = multiplet, and combinations thereof. ^{1}H and ^{13}C chemical shifts were referenced to $Si(CH_3)_4$ ($\delta(^{1}H,^{13}C)$ = 0 ppm) using the signals of the residual protons and of the ^{13}C of the deuterated solvent as secondary reference. ^{77}Se and ^{195}Pt chemical shifts were indirectly referenced with the ^{1}H signals of the residual protons of the deuterated solvent using the Ξ-scale (Ξ = 19.071513 MHz and 21.496784 MHz, respectively) with Me_2Se ($\delta(^{77}Se)$ = 0 ppm) or 1.2 M Na_2PtCl_6 in D_2O ($\delta(^{195}Pt)$ = 0 ppm) as secondary references.[4] NMR spectra were processed with MestreNova 14.2.1-27684 (Mestrelab Research S.L.) and TopSpin 4.2.0 (Bruker).

High resolution mass spectra (HRMS) were acquired by (nano)electrospray ionisation (ESI) using either a Thermo Orbitrap Elite instrument with an LTQ-Orbitrap analyser or a Waters XEVO G2-S QTOF instrument with a QTOF analyser. Elemental analysis was performed on a Thermo Scientific Flash 2000 Organic Elemental Analyser.

2. Synthesis and characterisation

2.1. Azolium (N,Y)HC proligands 1^{Y}·HX-4^{Y}·HX (Y = NR, O, S, Se ; X = halide)

1,3-Dimethyl-1H-imidazol-3-ium iodide (**1^{NMe}·HI**)[1]

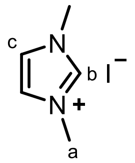

Imidazole (1 g, 1 eq., 14.69 mmol) and K_2CO_3 (3.04 g, 1.5 eq., 22.03 mmol) were suspended in acetonitrile (5 mL) and stirred at 90 °C for 1 h. Iodomethane (2.74 mL, 3 eq., 44.07 mmol) was added dropwise and the mixture was stirred at 90 °C for an additional 24 h. The suspension was filtered, washed with MeOH, acetone and DCM, and the solvent was removed under reduced pressure to yield a hygroscopic white solid (3.28 g, *quantitative*). ^{1}H NMR (400 MHz, $CDCl_3$): δ/ppm = 9.99 (s, 1H, H_b), 7.40 (d, *J* = 1.7 Hz, 2H, H_c), 4.10 (s, 6H, H_c) ^{13}C{^{1}H} NMR (101 MHz, $CDCl_3$): δ/ppm = 137.81 (C_b), 123.53 (C_c), 37.30 (C_a). HRMS (ESI/QTOF) m/z: [M – $I^–$]$^+$ Calcd for $C_5H_9N_2^+$ 97.0760; Found 97.0759.

3-Methyloxazol-3-ium iodide (**1^{O}·HI**)[2]

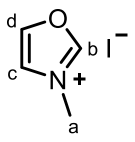

Oxazole (476 µL/500 mg, 1 eq., 7.24 mmol) and iodomethane (901 µL/2.06 g, 2 eq.) were stirred at 90 °C for 72 h. The suspension was filtered and washed with Et_2O and CH_2Cl_2 (2x1 mL) before drying under vacuum to yield a dark brown solid (748 mg, 49%). The product was stored in a flask under N_2 atmosphere. ^{1}H NMR (400 MHz, DMSO): δ/ppm = 10.22 – 10.17 (m, 1H, H_b), 8.76 (s, 1H, H_d), 8.25 (s, 1H, H_c), 3.93 (s, 3H, H_a). ^{13}C{^{1}H} NMR (101 MHz, DMSO): δ/ppm = 155.47 (C_b), 143.57 (C_d), 122.99 (C_c), 35.31 (C_a). HRMS (ESI/QTOF) m/z: [M – $I^–$]$^+$ Calcd for $C_4H_6NO^+$ 84.0444; Found 84.0447.

3-Methylthiazol-3-ium iodide (**1^{S}·HI**)[3]

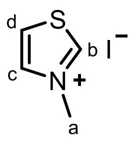

Thiazole (0.83 mL, 1 eq., 11.75 mmol) and iodomethane (0.73 mL, 1.2 eq., 14.09 mmol) were stirred at 90 °C for 18 h. The dark brown mixture was cooled to ambient temperature, dissolved in MeOH, and the solvent was removed under reduced pressure. The residue was triturated with diethyl ether and filtered to yield a cream solid (1.942 g, 75%). ^{1}H NMR (400 MHz, MeOD): δ/ppm = 8.39 (d, *J* = 3.7 Hz, 1H, H_c), 8.26 (d, *J* = 3.7 Hz, 1H, H_d), 4.29 (s, 3H, H_a). ^{13}C{^{1}H} NMR (101 MHz,

MeOD): δ/ppm = 160.61 (C_b), 139.54 (C_d), 127.13 (C_c), 42.53 (C_a). HRMS (nanochip-ESI/LTQ-Orbitrap) m/z: [M – I⁻]+ Calcd for C4H6NS+ 100.0215; Found 100.0216.

1,3-Dibenzyl-1H-imidazol-3-ium bromide (**2^{NBn}·HBr**)[4]

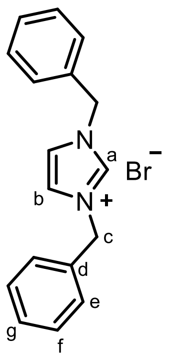

Molecular Weight: 329.24

Imidazole (1 g, 1 eq., 14.69 mmol) and K_2CO_3 (3.04 mg, 1.5 eq., 22.03 mmol) were suspended in acetonitrile (5 mL) and stirred at 90 °C for 1 h. Benzyl bromide (5.2 mL, 3 eq., 44.07 mmol) was added dropwise and the mixture was stirred at 90 °C for an additional 24 h. The suspension was filtered, washed with MeOH, acetone and DCM, and the solvent was removed under reduced pressure. The residue was suspended in Et_2O and filtered to yield a hygroscopic white solid (4.84 g, *quantitative*). ^{1}H NMR (400 MHz, $CDCl_3$): δ/ppm = 10.91 (d, *J* = 1.7 Hz, 1H, H_a), 7.50 – 7.44 (m, 4H, H_f), 7.41 – 7.32 (m, 6H, H_e + H_g), 7.17 (d, *J* = 1.7 Hz, 2H, H_b), 5.55 (s, 4H, H_c). ^{13}C{^{1}H} NMR (101 MHz, $CDCl_3$): δ/ppm = 136.99 (C_b), 132.50 (C_d), 129.39 (C_g), 129.29 (C_e), 128.86 (C_f), 121.56 (C_b), 53.29 (C_c). HRMS (ESI/QTOF) m/z: [M – I⁻]$^+$ Calcd for $C_{17}H_{17}N_2^+$ 249.1386; Found 249.1385.

3-Benzyloxazol-3-ium bromide (**2^O·HBr**)[5]

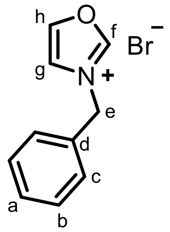

Oxazole (476 µL/500 mg, 1 eq., 7.24 mmol) and benzyl bromide (1.71 mL/2.48 g, 2 eq., 14.48 mmol) were stirred at 90 °C for 48 h. The mixture was filtered. The obtained solid was washed with Et_2O before drying under vacuum to yield cream-ochre crystals (1.12 g, 65%). ^{1}H NMR (400 MHz, $CDCl_3$): δ/ppm = 11.75 (s, 1H, H_f), 8.11 (s, 1H, H_h), 7.68 (s, 1H, H_g), 7.62 (dd, *J* = 6.7, 2.9 Hz, 2H, H_b), 7.49 – 7.41 (m, 3H, H_a + H_c), 6.03 (s, 2H, H_e). ^{1}H NMR (400 MHz, DMSO): δ/ppm = 10.47 (s, 1H, H_f), 8.82 (t, *J* = 1.2 Hz, 1H, H_h), 8.36 – 8.26 (m, 1H, H_g), 7.57 – 7.50 (m, 2H, H_b), 7.49 – 7.41 (m, 3H, H_a + H_c), 5.58 (s, 2H, H_e). ^{13}C{^{1}H} NMR (101 MHz, DMSO): δ/ppm = 155.43 (C_f), 144.37 (C_h), 132.50 (C_d), 129.24 (C_a), 129.05 (C_c), 129.03 (C_b), 121.54 (C_g), 51.53 (C_e). HRMS (ESI/QTOF) m/z: [M – Br⁻]$^+$ Calcd for $C_{10}H_{10}NO^+$ 160.0757; Found 160.0754.

3-Benzylthiazol-3-ium bromide (**2^S·HBr**)[5]

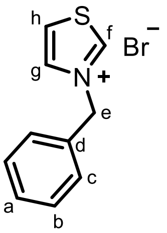

Thiazole (0.83 mL, 1 eq., 11.75 mmol) and benzyl bromide (1.4 mL, 1.2 eq., 14.09 mmol) were stirred at 90 °C for 18 h. The mixture was cooled to ambient temperature, dissolved in MeOH, and the solvent was removed under reduced pressure. The residue was triturated with Et_2O and filtered to yield a cream solid (3.00 g, 99%). ^{1}H NMR (400 MHz, MeOD): δ/ppm = 10.19 (s, 1H, H_f), 8.46 (d, *J* = 3.8 Hz, 1H, H_g), 8.29 (d, *J* = 3.8 Hz, 1H, H_h), 7.54 – 7.42 (m, 5H, H_a + H_b +H_c), 5.80 (s, 2H, H_e). $^{13}C\{^1H\}$ NMR (101 MHz, MeOD): δ/ppm = 160.04 (C_f), 138.32 (C_g), 134.21 (C_d), 130.92 (C_a), 130.67 (C_b), 130.11 (C_c), 127.92 (C_h), 59.65 (C_e). HRMS (nanochip-ESI/LTQ-Orbitrap) m/z: [M – Br^-]$^+$ Calcd for $C_{10}H_{10}NS^+$ 176.0528; Found 176.0533

1,3-Diethyl-1H-benzo[d]imidazol-3-ium iodide (**3^{NEt}·HI**)[6]

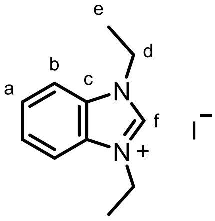

Benzimidazole (1 g, 1 eq., 8.46 mmol) and K_2CO_3 (1.75 mg, 1.5 eq., 12.69 mmol) were suspended in acetonitrile (5 mL) and stirred at 90 °C for 1 h. Iodoethane (2.04 mL, 3 eq., 25.39 mmol) was added dropwise and the mixture was stirred at 90 °C for an additional 24 h. The suspension was filtered, washed with MeOH, acetone and DCM, and the solvent was removed under reduced pressure. The residue was suspended in Et_2O and filtered to yield a hygroscopic white solid (2.55 g, *quantitative*). ^{1}H NMR (400 MHz, $CDCl_3$): δ/ppm = 11.18 (s, 1H, H_f), 7.79 – 7.64 (m, 4H, H_a + H_b), 4.69 (q, *J* = 7.3 Hz, 4H, H_d), 1.77 (t, *J* = 7.3 Hz, 6H, H_e). $^{13}C\{^1H\}$ NMR (101 MHz, $CDCl_3$): δ/ppm = 141.60 (C_f), 131.39 (C_c), 127.51 (C_a), 113.23 (C_b), 43.28 (C_d), 15.09 (C_e). HRMS (ESI/QTOF) m/z: [M – I^-]$^+$ Calcd for $C_{11}H_{15}N_2^+$ 175.1230; Found 175.1231.

3-Ethylbenzo[d]oxazol-3-ium iodide (**3°·HI**)

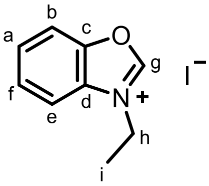

Benzoxazole (300 mg, 1 eq., 2.52 mmol) and iodoethane (405 µL/786 mg, 2 eq., 5.04 mmol) were stirred at 100 °C for 24 h. The product was obtained by filtration and washed with Et_2O, CH_2Cl_2 (2x1 mL), and more Et_2O before drying under vacuum to yield a brown powder (507 mg, 72%). The product was stored in a flask under N_2. ^{1}H NMR (400 MHz, $CDCl_3$): δ/ppm = 11.57 (s, 1H, H_g), 7.98 – 7.90 (m, 2H, H_a + H_f), 7.86 – 7.77 (m, 2H, H_b + H_e), 4.99 (dd, *J* = 7.3, 0.7 Hz, 2H, H_h), 1.88 (t, *J* = 7.4 Hz, 3H, H_i). $^{13}C\{^1H\}$ NMR (101 MHz,

$CDCl_3$): δ/ppm = 163.83 (C_g), 156.15 (C_c), 149.07 (C_d), 130.55 (C_a), 129.36 (C_b), 114.89 (C_f), 114.54 (C_e), 45.08 (C_h), 14.12 (C_i). HRMS (ESI/QTOF) m/z: [M – I^-]$^+$ Calcd for $C_9H_{10}NO^+$ 148.0757; Found 148.0762.

3-Ethylbenzo[d]thiazol-3-ium iodide (**3^S·HI**)[7]

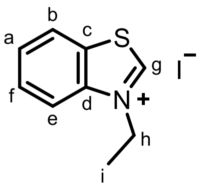

Benzothiazole (0.8 mL, 1 eq., 7.4 mmol) and iodoethane (0.71 mL, 1.2 eq., 8.88 mmol) were stirred at 90 °C for 18 h. The mixture was cooled to ambient temperature, dissolved in MeOH, and the solvent was removed under reduced pressure. The residue was triturated with Et_2O. The resulting red oil was frozen using liquid nitrogen and dried under vacuum, yielding a red-brown solid (2.04 g, 95%). ^{1}H NMR (400 MHz, MeOD): δ/ppm = 10.56 (s, 1H, H_g), 8.45 (d, J = 8.1 Hz, 1H, H_e), 8.39 (d, *J* = 8.6 Hz, 1H, H_b), 7.99 (ddd, *J* = 8.6, 7.2, 1.2 Hz, 1H, H_f), 7.89 (ddd, *J* = 8.3, 7.2, 1.1 Hz, 1H, H_a), 4.93 (q, *J* = 7.3 Hz, 2H, H_h), 1.75 (t, *J* = 7.3 Hz, 3H, H_i). ^{13}C{^{1}H} NMR (101 MHz, MeOD): δ/ppm = 163.91 (C_g), 141.78 (C_d), 133.05 (C_c), 131.30 (C_f), 130.15 (C_a), 126.13 (C_e), 118.13 (C_b), 49.99 (C_h), 14.52 (C_i). HRMS (nanochip-ESI/LTQ-Orbitrap) m/z: [M – I^-]$^+$ Calcd for $C_9H_{10}NS^+$ 164.0528; Found 164.0523.

3-Ethylbenzo[d][1,3]selenazol-3-ium iodide (**3^{Se}·HI**)

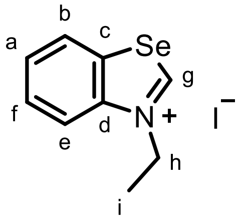

Benzoselenazole (333 mg, 1 eq., 1.83 mmol) and EtI (176 µL, 1.2 eq., 2.19 mmol) were stirred for 6 h at 90 °C. The mixture was cooled to room temperature, dissolved in MeOH, and the solvent was removed under reduced pressure. The residue was suspended in Et_2O and filtered to yield a bright orange solid (0.550 g, 89%). ^{1}H NMR (400 MHz, MeOD): δ/ppm = 11.21 (s, 1H), 8.47 (ddd, *J* = 8.2, 1.2, 0.6 Hz, 1H), 8.37 (dt, *J* = 8.6, 0.9 Hz, 1H), 7.93 (ddd, *J* = 8.5, 7.3, 1.2 Hz, 1H), 7.80 (ddd, *J* = 8.3, 7.2, 1.1 Hz, 1H), 4.90 (qd, *J* = 7.2, 0.6 Hz, 2H), 1.72 (t, *J* = 7.3 Hz, 3H). ^{13}C{^{1}H} NMR (101 MHz, MeOD): δ/ppm = 176.11 (C_g), 142.89 (C_d), 136.20 (C_c), 133.30 (C_i), 130.69 (C_l), 130.65 (C_k), 130.51 (C_f), 129.72 (C_a), 129.44 (C_j), 128.82 (C_e), 120.13 (C_b), 59.35 (C_h). ^{77}Se{^{1}H} NMR (76 MHz, MeOD): δ/ppm = 659.97. HRMS (ESI/QTOF) m/z: [M – I^-]$^+$ Calcd for $C_9H_{10}NSe^+$ 211.9973; Found 211.9974.

1,3-Dibenzyl-1H-benzo[d]imidazol-3-ium bromide (**4^{NBn}·HBr**)[8]

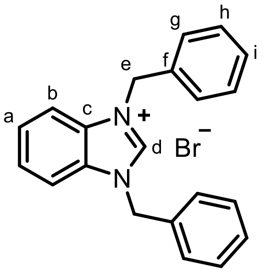

Benzimidazole (1 g, 1 eq., 8.46 mmol) and K_2CO_3 (1.75 mg, 1.5 eq., 12.69 mmol) were suspended in acetonitrile (5 mL) and stirred for 1 h at 90 °C. BnBr (3 mL, 3 eq., 25.39 mmol) was added dropwise and the mixture was stirred at 90 °C for an additional 24 h. The suspension was filtered, washed with MeOH, acetone and DCM, and the solvent was removed under reduced pressure. The residue was suspended in diethyl ether and filtered to yield a hygroscopic white solid (2.846 g, 89%). ^{1}H NMR (400 MHz, CDCl3): δ/ppm = 12.01 (s, 1H, H_d), 7.55 (m, 2H, H_b), 7.53 – 7.47 (m, 6H, $H_a + H_h$), 7.43 – 7.34 (m, 6H, $H_g + H_i$), 5.87 (s, 4H, H_e). ^{13}C{^{1}H} NMR (101 MHz, $CDCl_3$): δ/ppm = 143.39 (C_d), 129.63 (C_g), 128.48 (C_h), 113.87 (C_b), 51.74 (C_e). HRMS (ESI/QTOF) m/z: [M – Br$^-$]+ Calcd for $C_{21}H_{19}N_2^+$ 299.1543; Found 299.1551.

3-Benzylbenzo[d]thiazol-3-ium bromide (**4^{S}·HBr**)[9]

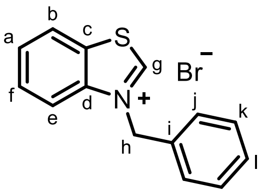

Benzothiazole (0.8 mL, 1 eq., 7.4 mmol) and benzyl bromide (1.05 mL, 1.2 eq., 8.88 mmol) were stirred at 90 °C for 18 h. The dark brown mixture was cooled to ambient temperature, dissolved in MeOH, and the solvent was removed under reduced pressure. The residue product was triturated with Et_2O and filtered to yield a cream solid (2.17 g, 96%). ^{1}H NMR (400 MHz, MeOD): δ/ppm = 10.52 (s, 1H, H_g), 8.47 – 8.40 (m, 1H, H_e), 8.35 – 8.28 (m, 1H, H_b), 7.92 (ddd, J = 8.5, 7.3, 1.4 Hz, 1H, H_f), 7.87 (ddd, J = 8.0, 7.2, 1.2 Hz, 1H, H_a), 7.57 – 7.39 (m, 5H, $H_j + H_k + H_l$), 6.12 (s, 2H, H_h). ^{13}C{^{1}H} NMR (101 MHz, MeOD): δ/ppm = 164.92 (C_g), 141.81 (C_d), 133.27 (C_c), 133.17 (C_i), 131.31 (C_l), 130.80 (C_f), 130.67 (C_k), 130.25 (C_a), 129.75 (C_j), 126.17 (C_e), 118.55 (C_b), 57.59 (C_h). HRMS (nanochip-ESI/LTQ-Orbitrap) m/z: [M – Br$^-$]$^+$ Calcd for $C_{14}H_{12}NS^+$ 226.0685; Found 226.0676.

3-Benzylbenzo[d][1,3]selenazol-3-ium bromide (**4^{Se}·HBr**)

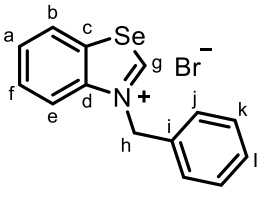

Benzoselenazole (333 mg, 1 eq., 1.83 mmol) and BzBr (260 µL, 1.2 eq., 2.19 mmol) were stirred for 3 h at 90 °C. The mixture was cooled to room temperature, dissolved in MeOH, and the solvent was removed under reduced pressure. The residue was

suspended in Et_2O and filtered to yield a cream-yellow solid (0.582 g, 90%). ^1H NMR (400 MHz, MeOD): δ/ppm = 11.19 (s, 1H, H_g), 8.50 – 8.43 (m, 1H, H_e), 8.34 – 8.27 (m, 1H, H_b), 7.85 (ddd, *J* = 8.6, 7.3, 1.3 Hz, 1H, H_f), 7.77 (ddd, *J* = 8.3, 7.2, 1.2 Hz, 1H, H_a), 7.50 – 7.41 (m, 5H, $H_j + H_k + H_l$), 6.08 (s, 2H, H_h). $^{13}C\{^1H\}$ NMR (101 MHz, MeOD): δ/ppm = 176.11 (C_g), 142.89 (C_d), 136.20 (C_c), 133.30 (C_i), 130.69 (C_l), 130.65 (C_k), 130.51 (C_f), 129.72 (C_a), 129.44 (C_j), 128.82 (C_e), 120.13 (C_b), 59.35 (C_h). $^{77}Se\{^1H\}$ NMR (76 MHz, MeOD): δ/ppm = 662.83. HRMS (ESI/QTOF) m/z: $[M – Br^-]^+$ Calcd for $C_{14}H_{12}NSe^+$ 274.0129; Found 274.0130.

2.2. (N,Y)HC metal complexes Pt1^Y, Pt2^Y, Au3^Y and Ru4^Y (Y = NR, O, S, Se)

(1,3-Dimethyl-1H-3λ⁴-imidazol-2-yl)diiodido(pyridine)platinum(II) (**Pt1^{NMe}**)[10]

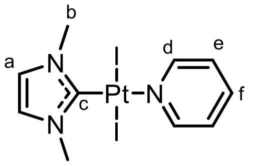

1,3-Dimethyl-1*H*-imidazol-1-ium iodide (98.6 mg, 1.1 eq., 0.44 mmol), $PtCl_2$ (106 mg, 1 eq., 0.4 mmol), K_2CO_3 (553 mg, 10 eq., 4 mmol) and NaI (600 mg, 10 eq., 4 mmol) were suspended in pyridine (2 mL). The mixture was sonicated for 1 h before stirring at 100 °C for 17 h. The solvent was removed under reduced pressure, and the residue was dissolved in CH_2Cl_2 and filtered, before removing the solvent under reduced pressure. The obtained solid was suspended in *n*-pentane, filtered, and washed with further *n*-pentane, yielding a yellow solid (108.8 mg, 44%). Intense yellow, X-ray quality, prism-shaped crystals were obtained by slow evaporation from a saturated $CHCl_3$ solution. ^1H NMR (400 MHz, $CDCl_3$): δ/ppm = 9.05 (dt, J = 5.0, 1.6 Hz, 2H, H_d), 7.77 – 7.68 (m, 1H, H_f), 7.36 – 7.29 (m, 2H, H_e), 6.83 (s, 2H, H_a), 3.96 (s, 6H, H_b). $^{13}C\{^1H\}$ NMR (101 MHz, $CDCl_3$): δ/ppm = 153.88 (C_d), 137.56 (C_f), 125.08 (C_e), 121.94 (C_a), 38.31 (C_b). $^{195}Pt\{^1H\}$ NMR (86 MHz, $CDCl_3$): δ/ppm = −4310. HRMS (nanochip-ESI/LTQ-Orbitrap) m/z: $[M – I^- + H_3N]^+$ Calcd for $C_{10}H_{16}IN_4Pt^+$ 514.0062; Found 514.0053.

(3-Methyl-1λ³-oxazol-2-yl)diiodido(pyridine)platinum(II) (**Pt1^O**)

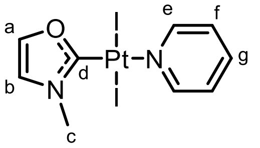

In a vial under an argon atmosphere, KOAc (27 mg, 2.2 eq., 0.27 mmol, dried at 150 °C), 3-methyloxazol-3-ium iodide (52 mg, 2 eq., 0.25 mmol), and (dimethylsulfoxide)diiodido(pyridine)platinum(II) (75 mg, 1 eq., 0.12 mmol) were dissolved in CH_2Cl_2 (20 mL, dry). The reaction was stirred at 25 °C for 24 h. The reaction was filtered to remove an unidentified brown precipitate, and the solvent was removed under reduced pressure. The residue was

recrystallised from CH_2Cl_2/*n*-pentane. The precipitate was isolated by filtration and washed with Et_2O followed by *n*-pentane, yielding a butterscotch-coloured powder (32 mg, 42%). Intense yellow, X-ray quality, prism-shaped crystals were obtained by slow diffusion of *n*-pentane into a saturated THF solution. ^1H NMR (400 MHz, $CDCl_3$): δ/ppm = 9.03 – 8.97 (m, 2H, H_e), 7.83 (d, *J* = 1.4 Hz, 1H, H_a), 7.76 (tt, *J* = 7.8, 1.7 Hz, 1H, H_g), 7.43 – 7.32 (m, 2H, H_f), 7.08 (d, *J* = 1.4 Hz, 1H, H_b), 4.01 (s, 3H, H_c). $^{13}C\{^1H\}$ NMR (101 MHz, $CDCl_3$): δ/ppm = 153.43 (C_e), 143.12 (C_a), 137.65 (C_g), 124.89 (C_f), 120.58 (C_b), 36.30 (C_c). $^{195}Pt\{^1H\}$ NMR (86 MHz, $CDCl_3$): δ/ppm = −4362. HRMS (ESI/QTOF) m/z: $[M + Na]^+$ Calcd for $C_9H_{10}I_2N_2NaOPt^+$ 633.8423; Found 633.8425. Anal. Calcd for $C_9H_{10}I_2N_2OPt$: C, 17.69; H, 1.65; N, 4.58. Found: C, 16.74; H, 1.76; N, 4.43.

(3-Methyl-3λ⁴-thiazol-2-yl)diiodido(pyridine)platinum(II) (**Pt1^S**)

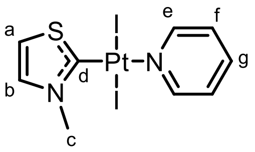

3-Methylthiazol-3-ium iodide (90.6 mg, 1 eq., 0.4 mmol), $PtCl_2$ (106 mg, 1 eq., 0.4 mmol), K_2CO_3 (551 mg, 10 eq., 3.99 mmol) and NaI (598 mg, 10 eq., 3.99 mmol) were suspended in pyridine (2 mL, dry). The mixture was sonicated for 1 h before stirring at 100 °C for 17 h. The solvent was removed under reduced pressure, and the residue was dissolved in CH_2Cl_2 and filtered, before concentrating the filtrate under reduced pressure. The residue was recrystallised from CH_2Cl_2/*n*-pentane. The resulting solid was obtained by filtration and washed with further *n*-pentane, yielding a mustard-coloured powder (99 mg, 40%). Intense yellow, X-ray quality, irregularly shaped crystals were grown by slow diffusion of *n*-pentane into a saturated CH_2Cl_2 solution. ^1H NMR (400 MHz, $CDCl_3$): δ/ppm = 9.04 – 8.97 (m, 2H, H_e), 7.74 (t, J = 7.7 Hz, 1H, H_g), 7.50 (d, J = 3.9 Hz, 1H, H_b), 7.43 (d, J = 3.9 Hz, 1H, H_a), 7.35 (t, J = 6.9 Hz, 2H, H_f), 4.30 (s, 3H, H_c). $^{13}C\{^1H\}$ NMR (101 MHz, $CDCl_3$): δ/ppm = 153.97 (C_e), 137.88 (C_g), 135.84 (C_b), 125.19 (C_f), 124.64 (C_a), 44.53 (C_c). $^{195}Pt\{^1H\}$ NMR (86 MHz, $CDCl_3$): δ/ppm = −4129. HRMS (ESI/QTOF) m/z: [M – I^- + MeCN + MeOH]+ Calcd for $C_{12}H_{17}IN_3OPtS^+$ 572.9779; Found 572.9781.

(1,3-Dibenzyl-1H-3λ⁴-imidazol-2-yl)diiodido(pyridine)platinum(II) (**Pt2^{NBn}**)[10]

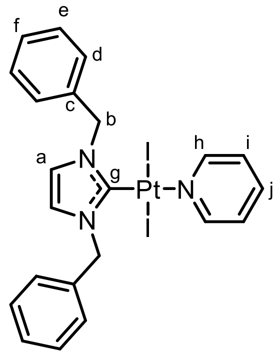

1,3-Dibenzyl-1*H*-imidazol-1-ium bromide (116.6 mg, 1.1 eq., 0.35 mmol), $PtCl_2$ (86 mg, 1 eq., 0.32 mmol), K_2CO_3 (445 mg, 10 eq., 3.22 mmol) and NaI (483 mg, 10 eq., 3.22 mmol) were suspended in pyridine (2 mL). The mixture was sonicated for 1 h before stirring at 100 °C for 17 h. The solvent was removed under reduced pressure, and the residue was dissolved in CH_2Cl_2 and filtered, before concentrating the filtrate under reduced pressure. The resulting solid was suspended in *n*-pentane, obtained by filtration through a nylon filter (pore size 0.45 µm), and washed with further *n*-pentane, yielding yellow crystals (200 mg, 80%). Pale-yellow, X-ray quality, prism-shaped crystals were grown by slow diffusion of *n*-pentane into a saturated CH_2Cl_2 solution. ^{1}H NMR (400 MHz, $CDCl_3$): δ/ppm = 9.06 (dt, J = 5.1, 1.6 Hz, 2H, H_h), 7.80 – 7.68 (m, 1H, H_j), 7.57 – 7.49 (m, 4H, H_e), 7.44 – 7.33 (m, 6H, $H_d + H_{fff}$), 7.37 – 7.26 (m, 2H, H_i), 6.58 (s, 2H, H_a), 5.76 (s, 3H, H_b). ^{13}C{^{1}H} NMR (101 MHz, $CDCl_3$): δ/ppm = 153.93 (C_h), 137.60 (C_j), 135.50 (C_c), 129.38 (C_e), 129.00 (C_d), 128.51 (C_f), 125.10 (C_i), 120.63 (C_a), 55.00 (C_b). ^{195}Pt{^{1}H} NMR (86 MHz, $CDCl_3$): δ/ppm = −4306. HRMS (nanochip-ESI/LTQ-Orbitrap) m/z: [M + Na]$^+$ Calcd for $C_{22}H_{21}I_2N_3NaPt^+$ 798.9365; Found 798.9372. Anal. Calcd for $C_{22}H_{21}I_2N_3Pt$: C, 34.04; H, 2.73; N, 5.41. Found: C, 33.56; H, 2.69; N, 5.37.

(3-Benzyl-1λ³-oxazol-2-yl)diiodido(pyridine)platinum(II) (**Pt2^{O}**)

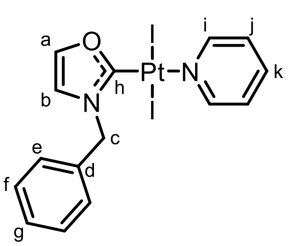

In a vial under an argon atmosphere, 3-benzyloxazol-3-ium bromide (59 mg, 2 eq., 0.25 mmol), (dimethylsulfoxide)diiodido(pyridine)platinum(II) (75 mg, 1 eq., 0.12 mmol), and KOAc (27 mg, 2.2 eq., 0.27 mmol dried at 150 °C) were dissolved in CH_2Cl_2 (5 mL, dry). The reaction was stirred at 25 °C for 4 h. The reaction was filtered and the solvent was removed under reduced pressure. CH_2Cl_2/*n*-pentane (3:5, 40 mL) was added to the mixture. The precipitate (bis(3-benzyl-3*H*-λ³-oxazol-2-yl)platinum(II) iodide) was removed by filtration. The product was recrystallised from the filtrate by adding additional *n*-pentane. The precipitate was obtained by filtration and washed with *n*-pentane, yielding a saffron-coloured powder (47 mg, 57%). ^{1}H NMR (400 MHz, $CDCl_3$): δ/ppm = 9.02 (d, *J* = 5.7 Hz, 2H, H_i), 7.79 – 7.68 (m, 2H, $H_a + H_k$), 7.58 – 7.51 (m, 2H, H_f), 7.45 – 7.32

(m, 5H, H_e + H_g + H_j), 6.82 (s, 1H, H_b), 5.73 (s, 2H, H_c). $^{13}C\{^1H\}$ NMR (101 MHz, $CDCl_3$): δ/ppm = 153.81 (C_i), 143.67 (C_a), 138.01 (C_k), 133.26 (C_d), 129.38 (C_f), 129.35 (C_e), 129.23 (C_g), 125.25 (C_j), 119.09 (C_b), 53.44 (C_c). $^{195}Pt\{^1H\}$ NMR (86 MHz, $CDCl_3$): δ/ppm = −4365. HRMS not found.

(3-Benzyl-3λ⁴-thiazol-2-yl)diiodido(pyridine)platinum(II) (**Pt2^{S}**)

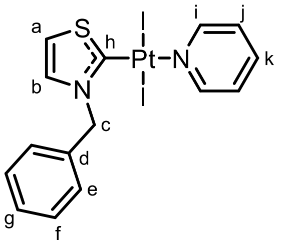

3-Benzylthiazol-3-ium iodide (90.9 mg, 1 eq., 0.36 mmol), $PtCl_2$ (94.4 mg, 1 eq., 0.36 mmol), K_2CO_3 (490.6 mg, 10 eq., 3.55 mmol) and NaI (532.1 mg, 10 eq., 3.55 mmol) were suspended in pyridine (2 mL, dry). The mixture was sonicated for 1 h before stirring at 100 °C for 17 h. The solvent was removed under reduced pressure, and the resulting solid was dissolved in CH_2Cl_2 and filtered, , before removing the solvent under reduced pressure. The residue was recrystallised from CH_2Cl_2/*n*-pentane. The precipitate was obtained by filtration through a nylon filter (pore size 0.45 µm) and washed with further *n*-pentane. A small amount of excess free pyridine was removed by vigorous heating under vacuum, yielding a dark brown powder (49 mg, 20%) ^1H NMR (400 MHz, $CDCl_3$): δ/ppm = 9.05 – 8.98 (m, 2H, H_i), 7.78 – 7.66 (m, 1H, H_k), 7.59 – 7.52 (m, 2H, H_f), 7.46 – 7.38 (m, 3H, H_a + H_e), 7.38 – 7.30 (m, 4H, H_b + H_g + H_j), 6.06 (s, 2H, H_c). $^{13}C\{^1H\}$ NMR (101 MHz, $CDCl_3$): δ/ppm = 154.01 (C_i), 137.89 (C_k), 134.35 (C_d), 134.29 (C_b), 129.42 (C_f), 129.33 (C_e), 129.13 (C_a), 125.20 (C_j), 124.87 (C_g), 61.39 (C_c). $^{195}Pt\{^1H\}$ NMR (86 MHz, $CDCl_3$): δ/ppm = −4129. HRMS (ESI/QTOF) m/z: $[M - I^-]^+$ Calcd for $C_{15}H_{14}IN_2PtS^+$ 575.9565; Found 575.9583.

Iodido(1,3-Diethyl-1H-3λ⁴-benzo[d]imidazol-2-yl)gold(I) (**Au3^{NEt}**)[11]

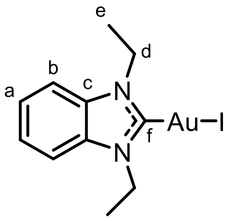

In a vial protected from light, 1,3-diethyl-1*H*-benzo[*d*]imidazol-3-ium iodide (223 mg, 1.2 eq., 0.74 mmol), K_2CO_3 (102 mg, 1.2 eq., 0.74 mmol), and $Au(SMe_2)Cl$ (181 mg, 1 eq., 0.62 mmol) were dissolved in acetone (15 mL, degassed) and stirred in the dark for 24 h at 60 °C. The solvent was removed under reduced pressure. The resulting solid was recrystallised from CH_2Cl_2/*n*-pentane, filtered, and washed with additional *n*-pentane and diethyl ether, yielding a light-grey solid (187 mg, 60%). Colourless, X-ray quality, prism-shaped crystals were grown by slow diffusion of *n*-pentane into a saturated CH_2Cl_2 solution. ^1H NMR (400 MHz, $CDCl_3$): δ/ppm = 7.54 – 7.40 (m, 4H, H_a + H_b),

4.55 (m, J = 7.3, 5.2 Hz, 4H, H_d), 1.55 (t, J = 7.3 Hz, 6H, H_e). $^{13}C\{^1H\}$ NMR (101 MHz, $CDCl_3$): δ/ppm = 132.81 (C_c), 124.64 (C_a), 111.58 (C_b), 43.71 (C_d), 15.66 (C_e). HRMS (nanochip-ESI/LTQ-Orbitrap) m/z: [M + Na]$^+$ Calcd for $C_{11}H_{14}AuIN_2Na^+$ 520.9760; Found 520.9750. Anal. Calcd for $C_{11}H_{14}AuIN_2$: C, 26.52; H, 2.83; N, 5.62. Found: C, 26.88; H, 2.88; N, 5.70.

Iodido(3-Ethyl-3H-1λ^3-benzo[d]oxazol-2-yl)gold(I) (**Au3^O**)

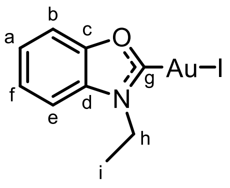

In a vial under an argon atmosphere and protected from light, 3-ethylbenzo[*d*]oxazol-3-ium iodide (85 mg, 1.2 eq., 0.31 mmol), KOAc (30 mg, 1.2 eq., 0.31 mmol, dried at 150 °C), and $Au(SMe_2)Cl$ (75 mg, 1 eq., 0.25 mmol) were dissolved in acetone (12 mL, dry and degassed) and stirred in the dark for 24 h at 60 °C. The solvent was removed under reduced pressure and the residue was dissolved in CH_2Cl_2. The mixture was filtered through celite and the solvent was removed under reduced pressure. The residue was recrystallised from CH_2Cl_2/*n*-pentane, filtered, and washed with additional *n*-pentane to yield a brown powder (62 mg, 52%). Intense yellow, X-ray quality, prism-shaped crystals were obtained by slow diffusion of *n*-pentane into a saturated THF solution. ^1H NMR (400 MHz, $CDCl_3$): δ/ppm = 7.78 – 7.71 (m, 1H, H_b), 7.64 – 7.51 (m, 3H, $H_a + H_f + H_e$), 4.65 (q, *J* = 7.3 Hz, 2H, H_h), 1.63 (t, *J* = 7.3 Hz, 3H, H_i). $^{13}C\{^1H\}$ NMR (101 MHz, $CDCl_3$): δ/ppm = 205.98 (C_g), 151.22 (C_c), 129.10 (C_d), 127.30 (C_f), 126.85 (C_a), 113.12 (C_b), 112.32 (C_e), 43.11 (C_h), 14.98 (C_i). HRMS (APCI/QTOF) m/z: [M + H]$^+$ Calcd for $C_9H_{10}AuINO^+$ 471.9467; Found 471.9477.

Iodido(3-Ethyl-3λ^4-benzo[d]thiazol-2-yl)gold(I) (**Au3^S**)

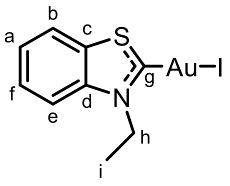

In a vial protected from light, 3-ethylbenzo[*d*]thiazol-3-ium iodide (66 mg, 1.2 eq., 0.31 mmol), K_2CO_3 (38 mg, 1.2 eq., 0.31 mmol), and $Au(SMe_2)Cl$ (75 mg, 1 eq., 0.25 mmol) were dissolved in acetone (12 mL, dry and degassed) and stirred in the dark for 24 h at 60 °C. The solvent was removed under reduced pressure and the residue was dissolved in CH_2Cl_2. The mixture was filtered through celite and the solvent was removed under reduced pressure. The residue was recrystallised from CH_2Cl_2/*n*-pentane, filtered, and washed with additional *n*-pentane to yield a bright yellow powder (37 mg, 30%). ^1H NMR (400 MHz, $CDCl_3$): δ/ppm = 7.94 (d, *J* = 7.8 Hz, 1H, H_e), 7.79 (d, *J* = 8.2 Hz, 1H, H_b), 7.69 (t, *J* = 7.9 Hz, 1H, H_a), 7.64 (t, *J* = 7.5 Hz, 1H, H_f), 4.88 (q, *J* = 7.3 Hz, 2H,

H_h), 1.66 (t, *J* = 7.3 Hz, 3H, H_i). $^{13}C\{^1H\}$ NMR (101 MHz, $CDCl_3$): δ/ppm = 211.04 (C_g), 141.95 (C_d), 133.39 (C_c), 128.30 (C_f), 126.68 (C_a), 123.24 (C_e), 115.05 (C_b), 50.58 (C_h), 14.98 (C_i). HRMS (ESI/QTOF) m/z: [M + Na]$^+$ Calcd for $C_9H_9AuINNaS^+$ 509.9058; Found 509.9065.

Iodido(3-Ethyl-3λ⁴-benzo[d]selenazol-2-yl)gold(I) (**Au3^{Se}**)

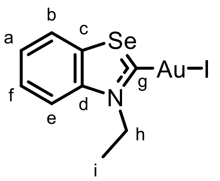

In a vial protected from light, 3-ethylbenzo[*d*][1,3]selenazol-3-ium iodide (66 mg, 1.2 eq., 0.31 mmol), K_2CO_3 (38 mg, 1.2 eq., 0.31 mmol), and $Au(SMe_2)Cl$ (75 mg, 1 eq., 0.25 mmol) were dissolved in acetone (12 mL, dry and degassed) and stirred in the dark for 24 h at 60 °C. The solvent was removed under reduced pressure and the residue was dissolved in CH_2Cl_2. The mixture was filtered through celite and the solvent was removed under reduced pressure. The residue was recrystallised from CH_2Cl_2/*n*-pentane, filtered, and washed with additional *n*-pentane to yield a dark yellow powder (43 mg, 26%). ^1H NMR (400 MHz, $CDCl_3$): δ/ppm = 8.12 – 8.02 (m, 1H, H_e), 7.73 – 7.65 (m, 1H, H_b), 7.36 – 7.28 (m, 1H, H_a), 7.18 – 7.09 (m, 1H, H_f), 3.87 – 3.73 (m, 2H, H_h), 1.22 – 1.06 (m, 3H, H_i). $^{13}C\{^1H\}$ NMR (101 MHz, $CDCl_3$): δ/ppm = 190.85 (C_g), 162.67 (C_d), 139.38 (C_c), 132.40 (C_f), 129.98 (C_a), 129.72 (C_e), 128.97 (C_b), 41.09 (C_h), 13.21 (C_i). $^{77}Se\{^1H\}$ NMR (76 MHz, $CDCl_3$): δ/ppm = 402.42. HRMS (nanochip-ESI/LTQ-Orbitrap) m/z: [M – I$^-$ + MeCN]$^+$ Calcd for $C_{11}H_{12}AuN_2Se^+$ 448.9826; Found 448.9958.

Dichlorido(1,3-dibenzyl-1H-3λ⁴-benzo[d]imidazol-2-yl)(η⁶-p-cymene)ruthenium(II) (**Ru4^{NBn}**)[12]

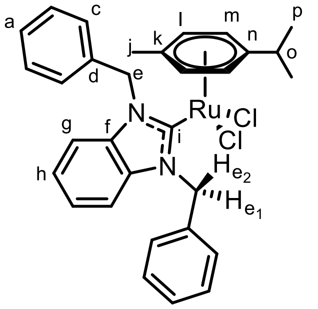

1,3-Dibenzyl-1*H*-benzo[*d*]imidazol-3-ium bromide (310 mg, 4 eq., 0.82 mmol) and silver(I) oxide (114 mg, 2.4 eq., 0.49 mmol) were dissolved in CH_2Cl_2 (18 mL, degassed). The reaction was stirred in the dark at 25 °C for 16 h, before filtering the reaction into a flask containing [Ru(η^6-*p*-cymene)Cl_2]$_2$ (125.5 mg, 1 eq., 0.205 mmol) dissolved in CH_2Cl_2 (9 mL, degassed), with a yellowish precipitate forming instantaneously. The reaction was stirred at 25 °C for 24 h before filtering over celite, and the solvent was removed under reduced pressure. The product was recrystallised from CH_2Cl_2/*n*-pentane (1:1.5), cooled to -20 °C, isolated by filtration and washed with *n*-pentane to yield an orange

powder (96 mg, 39%). Orange, X-ray quality, needle-shaped crystals were grown by slow diffusion of *n*-pentane into a saturated CH_2Cl_2 solution. ^{1}H NMR (400 MHz, $CDCl_3$): δ/ppm = 7.36 (t, *J* = 7.4 Hz, 4H, H_b), 7.29 (t, *J* = 7.3 Hz, 2H, H_a), 7.12 (d, *J* = 7.5 Hz, 4H, H_c), 7.10 – 7.05 (m, 2H, H_h), 7.05 – 7.00 (m, 2H, H_g), 6.56 (d, *J* = 17.0 Hz, 2H, H_{e1}), 5.84 (d, *J* = 17.2 Hz, 2H, H_{e2}), 5.35 (d, *J* = 5.8 Hz, 2H, H_l), 5.06 (d, *J* = 5.8 Hz, 2H, H_m), 2.73 (h, *J* = 6.8 Hz, 1H, H_o), 1.89 (s, 3H, H_j), 1.17 (d, *J* = 6.9 Hz, 6H, H_p). $^{13}C\{^1H\}$ NMR (101 MHz, $CDCl_3$): δ/ppm = 191.86 (C_i), 137.64 (C_d), 135.72 (C_f), 129.08 (C_b), 127.64 (C_a), 126.10 (C_c), 123.28 (C_h), 111.81 (C_g), 108.19 (C_n), 97.42 (C_k), 85.63 (C_l), 84.78 (C_m), 53.15 (C_e), 30.78 (C_o), 22.63 (C_p), 18.45 (C_j). HRMS (ESI/QTOF) m/z: $[M - Cl^-]^+$ Calcd for $C_{31}H_{32}ClN_2Ru^+$ 569.1292; Found 569.1311.

Dichlorido(3-benzyl-3λ⁴-benzo[d]thiazol-2-yl)(η⁶-p-cymene)ruthenium(II) (**Ru4^{S}**)

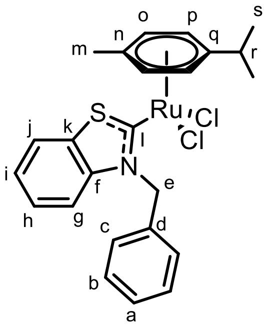

3-Benzylbenzo[*d*]thiazole-3-ium bromide (31.8 mg, 2 eq., 0.10 mmol), silver(I) oxide (22.1 mg, 2 eq., 0.10 mmol), and [Ru(η^6-*p*-cymene)Cl_2]$_2$ (34.2 mg, 1 eq., 0.06 mmol) were dissolved in CH_2Cl_2 (2 mL, dry and degassed) under a N_2 atmosphere. The reaction was stirred in the dark at 25 °C for 28 h before filtering over celite, and the filtrate was concentrated under reduced pressure. The oily residue was dissolved in a minimum volume of DCM and purified by column chromatography over SiO_2 using 1:25 MeOH/CH_2Cl_2 as eluant. The first red band was collected and concentrated under reduced pressure to yield a red powder (28 mg, 51%). Intense orange, X-ray quality, plate-shaped crystals were grown by slow diffusion of Et_2O into a saturated THF solution. ^{1}H NMR (400 MHz, $CDCl_3$): δ/ppm = 7.83 – 7.76 (m, 1H, H_g), 7.45 – 7.28 (m, 6H, $H_a + H_b + H_h + H_i + H_j$), 7.09 (d, *J* = 7.0 Hz, 2H, H_c), 6.36 (s, 2H, H_e), 5.28 (d, *J* = 5.9 Hz, 2H, H_o), 5.14 (d, *J* = 5.9 Hz, 2H, H_p), 2.83 (hept, *J* = 7.5 Hz, 1H, H_r), 2.10 (s, 3H, H_m), 1.22 (d, *J* = 6.9 Hz, 6H, H_s). $^{13}C\{^1H\}$ NMR (101 MHz, $CDCl_3$): δ/ppm = 144.80 (C_f), 136.58 (C_d), 136.35 (C_k), 129.49 (C_b), 128.11 (C_a), 126.46 (C_h), 125.49 (C_c), 124.96 (C_i), 121.58 (C_g), 114.84 (C_j), 106.34 (C_q), 101.07 (C_n), 86.69 (C_o), 86.22 (C_p), 56.81 (C_e), 30.78 (C_r), 22.38 (C_s), 18.30 (C_m). HRMS (ESI/QTOF) m/z: $[M + Cl^-]^+$ Calcd for $C_{24}H_{25}ClNRuS^+$ 496.0434; Found 496.0433.

Dichlorido(3-benzyl-3λ⁴-benzo[d][1,3]selenazol-2-yl)(η⁶-p-cymene)ruthenium(II) (**Ru4^{Se}**)

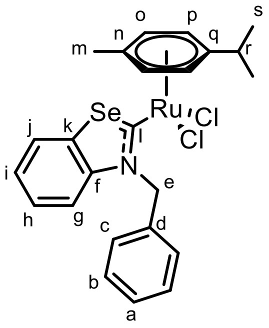

3-Benzylbenzo[*d*][1,3]selenazol-3-ium bromide (115 mg, 2 eq., 0.33 mmol), silver(I) oxide (60 mg, 1.5 eq., 0.24 mmol), and [Ru(η^6^-*p*-cymene)Cl_2]$_2$ (100 mg, 1 eq., 0.16 mmol) were dissolved in CH_2Cl_2 (10 mL, dry and degassed). The reaction was stirred in the dark at 25 °C for 24 h before filtering over celite, and the solvent was removed under reduced pressure. The residue was dissolved in CH_2Cl_2 (5 mL) before adding *n*-pentane (60 mL), leaving a red-brown film on the flask. The mixture was filtered. The desired compound was then recrystallised by addition of a further 40 mL of *n*-pentane to the filtrate. The precipitate was isolated by filtration and washed with additional *n*-pentane to yield an orange powder (37 mg, 20%). Intense orange, X-ray quality, plate-shaped crystals were grown by slow diffusion of *n*-pentane into a saturated THF solution. ^{1}H NMR (400 MHz, $CDCl_3$): δ/ppm = 7.86 (d, J = 7.3 Hz, 1H, H_g), 7.45 – 7.27 (m, 6H, $H_a + H_b + H_h + H_i + H_j$), 7.11 (d, J = 6.9 Hz, 2H, H_c), 6.37 (s, 2H, H_e), 5.28 (d, J = 5.9 Hz, 2H, H_o), 5.14 (d, J = 5.8 Hz, 2H, H_p), 2.80 (hept, 1H, H_r), 2.10 (s, 3H, H_m), 1.21 (d, J = 6.9 Hz, 6H, H_s). $^{13}C\{^{1}H\}$ NMR (101 MHz, $CDCl_3$): δ/ppm = 146.25 (C_f), 136.85 (C_d), 136.69 (C_k), 129.50 (C_b), 128.07 (C_a), 126.10 (C_h), 125.52 (C_c), 125.06 (C_i), 124.85 (C_g), 116.75 (C_j), 106.59 (C_q), 101.43 (C_n), 88.69 (C_o), 86.36 (C_p), 58.95 (C_e), 30.75 (C_r), 22.39 (C_s), 18.31 (C_m). HRMS (nanochip-ESI/LTQ-Orbitrap) m/z: [M + Cl^-]$^+$ Calcd for $C_{24}H_{25}ClNRuSe^+$ 543.9879; Found 543.9893.

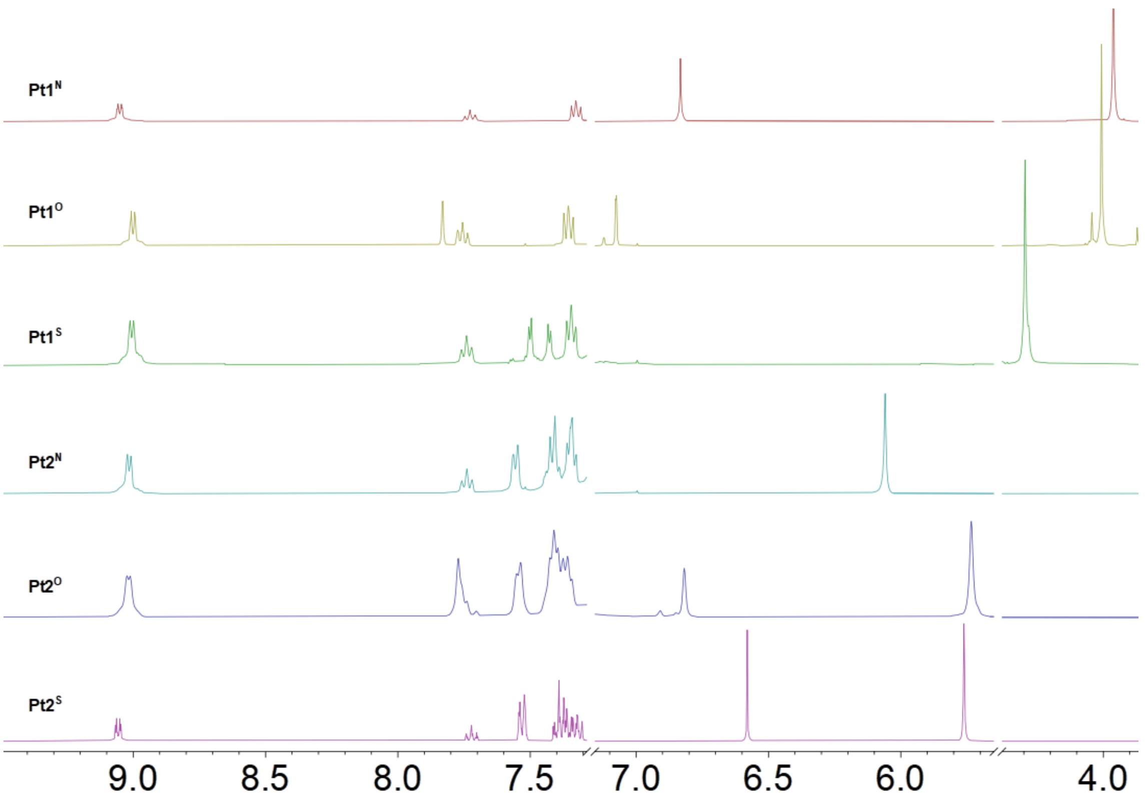

Supplementary Figure 1. ^{1}H NMR spectra of $\mathbf{Pt1^{Y}}$ and $\mathbf{Pt2^{Y}}$ (Y = NR, O, S).

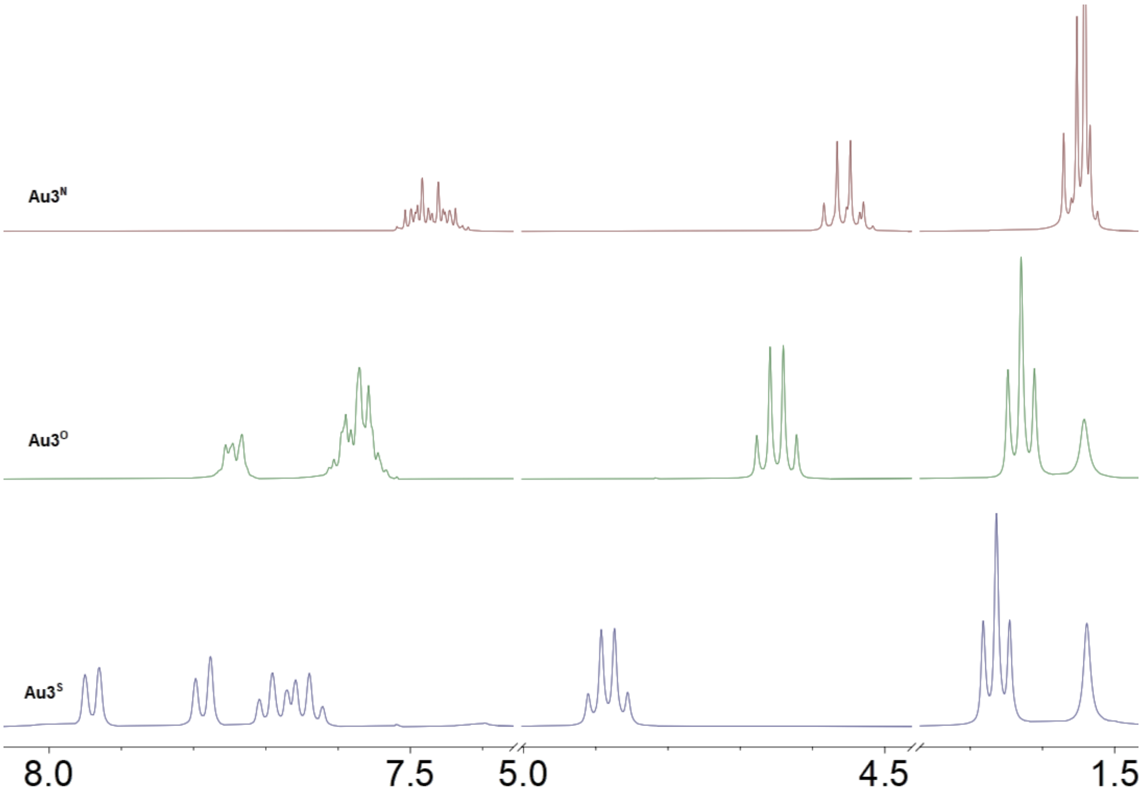

Supplementary Figure 2. ^{1}H NMR spectra of $\mathbf{Au3^{Y}}$ (Y = NEt, O, S).

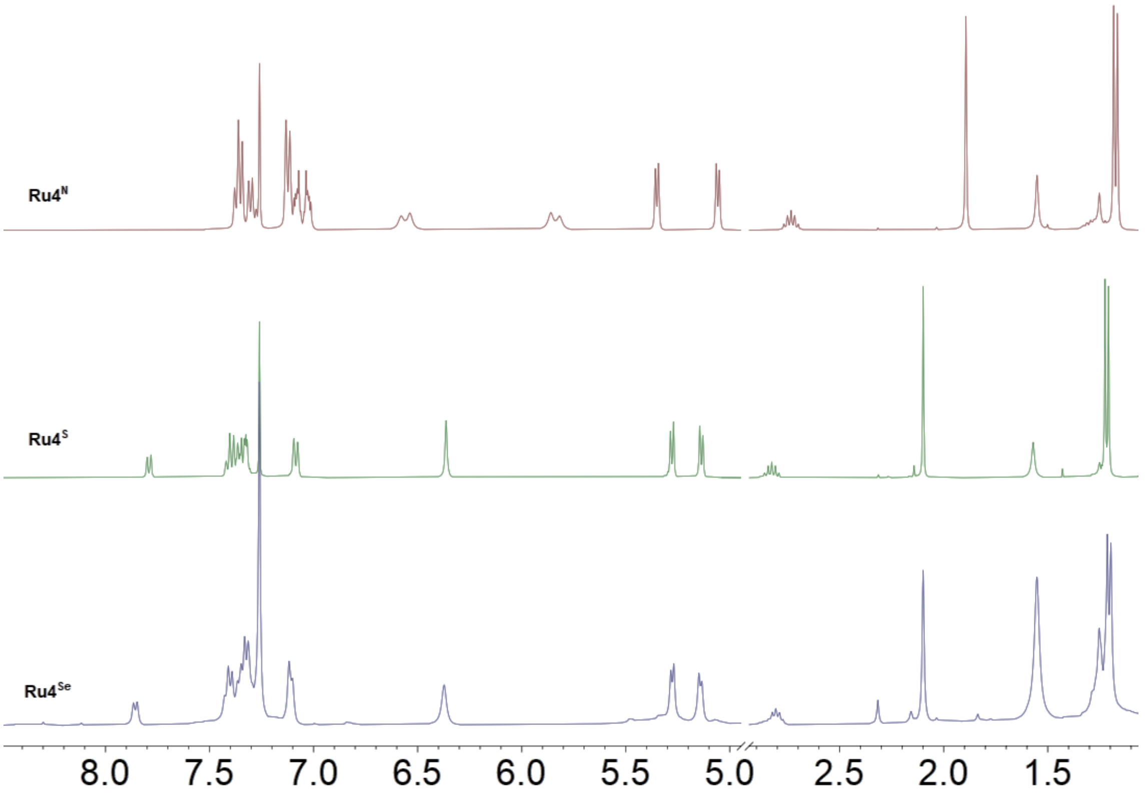

Supplementary Figure 3. ^{1}H NMR spectra of **Ru4^{Y}** (Y = NBn, S, Se).

2.3. Single crystal X-ray diffraction analysis

Crystals of **Pt1^{NMe}**, **Pt1^{O}**, **Pt1^{S}**, **Pt2^{NBn}**, **Au3^{NEt}**, **Au3^{O}**, **Au3^{S}**, **Ru4^{NBn}**, **Ru4^{S}**, and **Ru4^{Se}** were mounted on a XtaLAB Synergy R, DW system, HyPix-Arc 150 diffractometer or on a SuperNova, Dual, Cu at home/near, AtlasS2 diffractometer. The crystals were kept at a steady T = 140.00(10) K or T = 103.00(10) K during data collection. The structures were solved with the **ShelXT** solution program using dual methods and by using **Olex2** 1.5 as the graphical interface.[13,14] The models were refined with **ShelXL** 2018/3 (Sheldrick, 2015) using full matrix least squares minimisation on $\boldsymbol{F^2}$.[15]

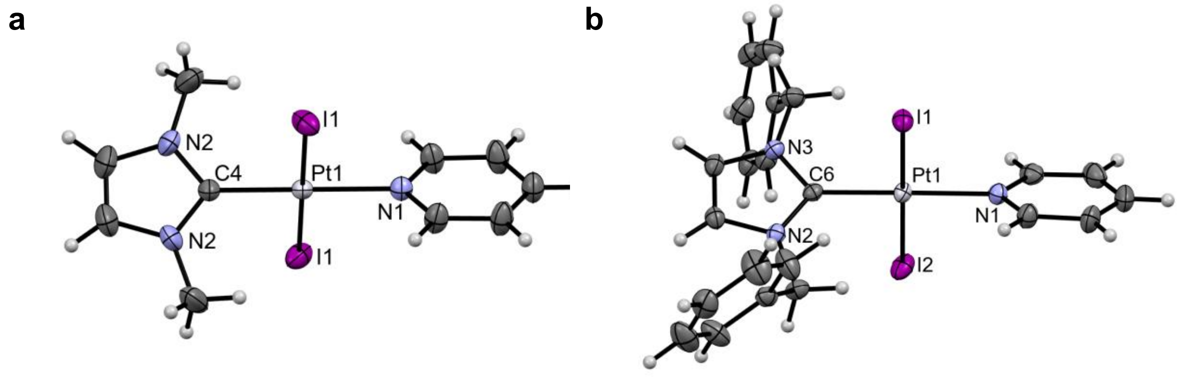

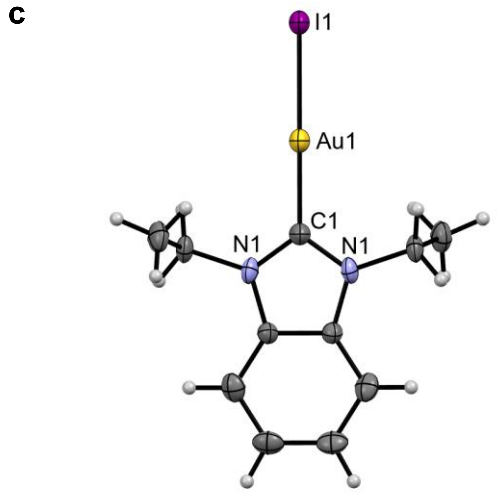

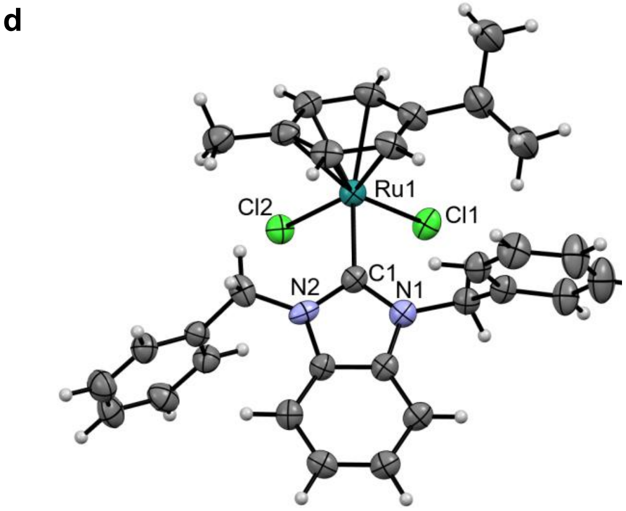

Supplementary Figure 4. Single crystal XRD structures of **Pt1^{NMe}** (**a**), **Pt2^{NBn}** (**b**), **Au3^{NEt}** (**c**), and **Ru4^{NBn}** (**d**). Thermal ellipsoids are drawn with a 50% probability. Hydrogen atoms, solvates and disorder have been omitted for clarity.

Supplementary Table 1. Crystallographic data for **Pt1^{NMe}**, **Pt1^{O}**, **Pt1^{S}** and **Pt2^{NBn}**.

**Compound**	**Pt1^{NMe}**	**Pt1^{O}**	**Pt1^{S}**	**Pt2^{NBn}**
Formula	$C_{10}H_{13}I_2N_3Pt$	$C_9H_{10}N_2OI_2Pt$	$C_9H_{10}I_2N_2PtS$	$C_{22}H_{21}I_2N_3Pt$
$D_{calc.}$/ g cm^{-3}	2.717	3.011	2.909	2.272
μ/mm^{-1}	13.224	55.266	14.231	8.914
Formula Weight	624.12	611.08	627.14	776.31
Colour	clear intense yellow	colourless	clear intense yellow	clear pale yellow
Shape	prism-shaped	irregular-shaped	irregular-shaped	plate-shaped
Size/mm^3	0.32×0.28×0.15	0.08×0.07×0.05	0.13×0.12×0.05	0.22×0.07×0.02
T/K	139.99(10)	140.00(10)	140.00(10)	139.99(10)
Crystal System	hexagonal	monoclinic	hexagonal	triclinic
Flack Parameter	0.006(2)		0.003(3)	
Hooft Parameter	0.0097(19)		0.003(3)	
Space Group	$P6_522$	$P2_1/c$	$P6_122$	*P*-1
a/Å	9.25931(7)	8.8706(3)	9.2044(2)	8.3972(4)
b/Å	9.25931(7)	15.3280(7)	9.2044(2)	11.7055(5)
c/Å	30.8237(4)	19.8300(10)	29.2718(6)	12.1100(6)
a/°	90	90	90	93.480(4)
b/°	90	90.089(4)	90	98.668(4)
g/°	120	90	120	104.169(4)

$V/Å^3$	2288.61(4)	2696.3(2)	2147.69(10)	1135.01(10)
Z	6	8	6	2
Z'	0.5	2	0.5	1
Wavelength/Å	0.71073	1.54184	0.71073	0.71073
Radiation type	Mo $K_α$	Cu $K_α$	Mo $K_α$	Mo $K_α$
$Q_{min}/°$	2.540	3.645	2.555	2.356
$Q_{max}/°$	40.858	74.951	36.429	40.802
Measured Refl's.	165378	21502	74558	30293
Indep't Refl's	4701	5212	3423	11552
Refl's I≥2*s*(I)	4242	3379	3045	7713
R_{int}	0.0684	0.0826	0.0617	0.0339
Parameters	77	252	85	253
Restraints	0	283	0	0
Largest Peak/e $Å^{-3}$	1.592	7.408	1.475	1.120
Deepest Hole/e $Å^{-3}$	-1.143	-2.558	-0.781	-1.433
GooF	1.101	1.041	1.141	0.986
wR_2 (all data)	0.0450	0.2986	0.0488	0.0651
wR_2	0.0441	0.2619	0.0474	0.0582
R_1 (all data)	0.0335	0.1336	0.0370	0.0661
R_1	0.0269	0.0985	0.0282	0.0329
CCDC number	**2270446**	**2395164**	**2270445**	**2270447**

Supplementary Table 2. Crystallographic data for **Au3^{NEt}**, **Au3^{O}**, **Au3^{S}**, **Ru4^{NBn}**, **Ru4^{S}** and **Ru4^{Se}**.

**Compound**	**Au3^{NMe} (X = I)**	**Au3^{O} (X = I)**	**Au3^{S} (X = Br)**	**Ru4^{NBn}**	**Ru4^{S}**	**Ru4^{Se}**
Formula	$C_{11}H_{14}AuIN_2$	C_9H_9AuINO	$C_9H_9AuBrNS$	$C_{32}H_{34}Cl_4N_2Ru$	$C_{28}H_{33}Cl_2NORuS$	$C_{28}H_{33}Cl_2NORuSe$
$D_{calc.}$/ g cm^{-3}	2.599	2.934	2.738	1.495	1.501	1.612
μ/mm^{-1}	40.512	48.340	17.667	7.535	0.887	8.280
Formula Weight	498.11	471.04	440.11	689.48	603.58	650.48
Colour	clear pale colourless	colourless	clear light yellow	orange	clear intense orange	clear intense orange
Shape	prism-shaped	needle-shaped	prism-shaped	needle-shaped	plate-shaped	plate-shaped
Size/mm^3	0.19×0.08×0.06	0.21×0.01×0.01	0.22×0.20×0.10	0.28×0.07×0.05	0.58×0.21×0.04	0.18×0.10×0.04
T/K	103(4)	139.99(10)	140.00(10)	139.99(10)	139.99(10)	140.00(10)
Crystal System	monoclinic	orthorhombic	monoclinic	monoclinic	triclinic	triclinic
Flack Parameter		0.029(13)				
Space Group	$C2/c$	$Pna2_1$	$C2/c$	$P2_1/n$	$P\bar{1}$	$P\bar{1}$
a/Å	10.6281(3)	6.92293(16)	16.4314(4)	15.0757(5)	9.75755(16)	9.7647(2)
b/Å	16.7383(5)	28.2837(4)	9.4384(2)	12.3164(2)	10.13025(18)	10.0920(2)
c/Å	7.1700(3)	16.3408(2)	13.8055(3)	18.0889(7)	15.2002(3)	15.3151(3)
a/°	90	90	90	90	107.3376(16)	107.2390(18)
b/°	93.494(3)	90	94.094(2)	114.216(5)	98.0674(15)	98.9646(18)
g/°	90	90	90	90	106.1798(15)	105.768(2)
V/Å^3	1273.14(8)	3199.63(10)	2135.56(9)	3063.2(2)	1335.90(4)	1340.55(5)

Z	4	12	8	4	2	2
Z'	0.5	3	1	1	1	1
Wavelength/Å	1.54184	1.54184	0.71073	1.54184	0.71073	1.54184
Radiation type	Cu K_α	Cu K_α	Mo K_α	Cu K_α	Mo K_α	Cu K_α
Q_{min}/°	4.936	3.123	2.485	3.232	2.193	3.126
Q_{max}/°	74.926	74.815	41.024	75.607	30.508	75.321
Measured Refl's.	11353	25185	24582	57987	29275	16526
Indep't Refl's	1266	6178	5634	6163	8147	5254
Refl's I≥2*s*(I)	1168	5224	4154	4515	7595	4860
R_{int}	0.0960	0.0580	0.0358	0.1118	0.0199	0.0215
Parameters	71	355	119	355	310	356
Restraints	0	294	0	0	39	156
Largest Peak/e Å^{-3}	2.959	7.702	1.703	2.240	1.201	0.614
Deepest Hole/e Å^{-3}	-2.766	-5.737	-1.028	-0.950	-1.207	-0.701
GooF	1.057	1.136	1.020	1.042	1.054	1.063
wR_2 (all data)	0.1072	0.1725	0.0656	0.1899	0.0964	0.0756
wR_2	0.1056	0.1661	0.0606	0.1694	0.0949	0.0744
R_1 (all data)	0.0422	0.0864	0.0523	0.0951	0.0374	0.0300
R_1	0.0398	0.0730	0.0297	0.0673	0.0346	0.0276
CCDC number	**2270450**	**2395165**	**2270448**	**2270449**	**2271662**	**2130715**

2.4. Other metal precursors and complexes

(Dimethylsulfoxide)dichlorido(pyridine)platinum(II)[16]

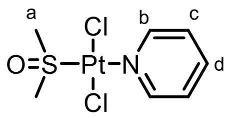

Potassium tetrachloroplatinate(II) (1g, 1 eq., 2.40 mmol) and dimethyl sulfoxide (171 µL, 1 eq, 2.40 mmol) were dissolved in H_2O (20 mL) and stirred at 25 °C for 16 h. The yellow solution was filtered to remove trace amounts of *cis*-[$PtCl_2(Me_2SO)_2$]. Pyridine (195 µL, 1 eq., 2.40 mmol) was added to the filtrate, causing instantaneous formation of a pale-yellow precipitate. The reaction was stirred at 25 °C for 30 min and the precipitate was isolated by filtration and washed with H_2O and Et_2O, yielding a pale-yellow powder (920 mg, 90%). ^1H NMR (400 MHz, $CDCl_3$): δ/ppm = 8.82 – 8.71 (m, 2H, H_b), 7.89 (tt, J = 7.7, 1.7 Hz, 1H, H_d), 7.45 (t, J = 6.9 Hz, 2H, H_c), 3.46 (s, 6H, H_a). $^{13}C\{^1H\}$ NMR (101 MHz, $CDCl_3$): δ/ppm = 152.20 (C_b), 139.69 (C_d), 125.70 (C_c), 44.38 (C_a). $^{195}Pt\{^1H\}$ NMR (86 MHz, $CDCl_3$): δ/ppm = -3020.12. HRMS (ESI/QTOF) m/z: [M + Na]$^+$ Calcd for $C_7H_{11}Cl_2NNaOPtS^+$ 444.9479; Found 444.9496. Anal. Calcd for $C_7H_{11}Cl_2NOPtS$: C, 19.87; H, 2.62; N, 3.31; S, 7.58. Found: C, 19.98; H, 2.67; N, 3.33; S, 7.84.

(Dimethylsulfoxide)diiodido(pyridine)platinum(II)

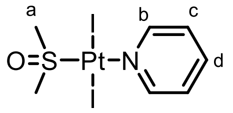

(Dimethylsulfoxide)dichlorido(pyridine)platinum(II) (300 mg, 1 eq., 0.71 mmol) was dissolved in acetone (30 mL) and cooled to -78 °C. NaI (319 mg, 3 eq., 2.13 mmol) was added and the mixture was stirred at -78 °C for 30 min. The reaction was allowed to warm to 0 °C, changing colour from pale-yellow to yellow and forming a coral-coloured precipitate. The reaction was stirred at 0 °C for a further 10 min. Upon warming to ambient temperature, the precipitate was isolated by filtration and washed with H_2O and Et_2O, yielding a coral-coloured powder (355 mg, 83%). ^1H NMR (400 MHz, $CDCl_3$): δ/ppm = 8.79 – 8.70 (m, 2H, H_b), 7.79 (tt, J = 7.7, 1.6 Hz, 1H, H_d), 7.46 – 7.35 (m, 2H, H_c), 3.87 (s, 6H, H_a). $^{13}C\{^1H\}$ NMR (101 MHz, $CDCl_3$): δ/ppm = 153.81 (C_b), 139.10 (C_d), 125.66 (C_c), 51.24 (C_a). $^{195}Pt\{^1H\}$ NMR (86 MHz, $CDCl_3$): δ/ppm = -4326.82. HRMS (ESI/QTOF) m/z: [M + H]$^+$ Calcd for $C_7H_{12}I_2NOPtS^+$ 606.8371; Found 606.8395. Anal. Calcd for $C_7H_{11}I_2NOPtS$: C, 13.87; H, 1.83; N, 2.31; S, 5.29. Found: C, 14.04; H, 1.79; N, 2.30; S, 5.97.

Chlorido(dimethylsulfide)gold(I) (**AuDMSCl**)[17]

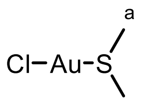

Hydrogen tetrachloroaurate(III) (1 g, 1 eq., 2.54 mmol) was dissolved in EtOH (24 mL, degassed), before adding a solution of dimethylsulfide (560 µL, 3 eq., 7.62 mmol) in EtOH (20 mL. degassed). The mixture was stirred in the dark at ambient temperature for 3 h, and subsequently cooled to -20 °C. The precipitate was obtained by filtration followed by washing with cold EtOH and cold Et_2O. The product was dried under vacuum, yielding a white powder (655 mg, 88%). ^{1}H NMR (400 MHz, $CDCl_3$): δ/ppm = 2.76 (s, 6H, H_a). $^{13}C\{^1H\}$ NMR (101 MHz, $CDCl_3$): δ/ppm = 25.03 (C_a). HRMS (ESI/QTOF) m/z: $[M - Cl^- + MeCN]^+$ Calcd for $C_4H_9AuNS^+$ 300.0116; Found 300.0126.

bis(η⁶-p-cymene)di-µ-chlorodichlorodiruthenium (**[Ru(η⁶-p-cymene)Cl₂]₂**)[18]

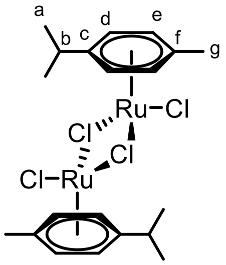

A solution of ruthenium (III) chloride hydrate (3 g, 1 eq., 11.47 mmol based on $RuCl_3 \cdot 3H_2O$) and α-phellandrene (17.9 mL, 10 eq., 115 mmol) in EtOH (150 mL, degassed) was stirred in a closed vessel at 115 °C for 20 h. The mixture was concentrated to half of its volume under reduced pressure and subsequently cooled to -20 °C. The precipitate was obtained by filtration and washed with cold Et_2O, yielding a dark red powder (3.19 g, quantitative). ^{1}H NMR (400 MHz, $CDCl_3$): δ/ppm = 5.47 (d, J = 5.9 Hz, 4H, H_e), 5.33 (d, J = 5.9 Hz, 4H, H_d), 2.92 (hept, J = 6.9 Hz, 2H, H_b), 2.16 (s, 6H, H_g), 1.28 (d, J = 6.9 Hz, 12H, H_a). $^{13}C\{^1H\}$ NMR (101 MHz, $CDCl_3$): δ/ppm = 101.38 (C_c), 96.90 (C_f), 81.45 (C_e), 80.68 (C_d), 30.77 (C_b), 22.30 (C_a), 19.08 (C_g). Anal. Calcd for $C_{20}H_{28}Ru_2Cl_4$: C, 39.23; H, 4.61. Found: C, 39.39; H, 4.57.

Dichlorido(η6-p-cymene)(dimethylsulfoxide)ruthenium[19]

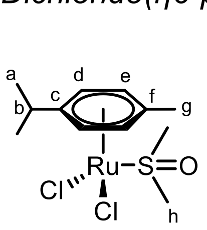

Bis(η⁶-*p*-cymene)di-*µ*-chlorodichlorodiruthenium (300 mg, 1 eq., 0.49 mmol) and dimethyl sulfoxide (70 µL, 2 eq., 0.98 mmol) where dissolved in CH_2Cl_2 and stirred at 40 °C for 4h. The resulting solution was concentrated under reduced pressure and the residue was recrystallised from CH_2Cl_2/*n*-pentane. The precipitate was isolated by filtration and washed with *n*-pentane to yield an orange powder (361 mg, 96%). ^{1}H NMR (400 MHz, DMSO): δ/ppm = 5.82 (d, *J* = 6.1 Hz, 2H, H_d), 5.77

(d, J = 6.3 Hz, 2H, H_e), 2.83 (hept, J = 6.9 Hz, 1H) 2.54 (s, 6H, H_h), 2.09 (s, 3H, H_c), 1.19 (d, J = 6.9 Hz, 6H, H_c). ^{13}C NMR (101 MHz, DMSO): δ/ppm = 106.36 (C_c), 100.07 (C_f), 86.35 (C_e), 85.50 (C_d), 40.43 (C_h), 29.96 (C_b), 21.49 (C_a), 17.86 (C_g).

Bromido(3-ethyl-3λ⁴-benzo[d]thiazol-2-yl)gold(I) (**Au3^{S}** (X = Br))

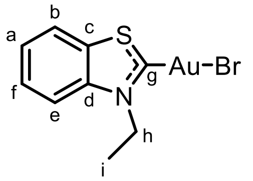

In a vial protected from light, 3-ethylbenzo[*d*]thiazol-3-ium bromide (66 mg, 1 eq., 0.27 mmol), K_2CO_3 (38 mg, 1 eq., 0.27 mmol), and Au(SMe_2)Cl (80 mg, 1 eq., 0.27 mmol) were dissolved in acetone (12 mL, dry and degassed) and stirred in the dark for 24 h at 60 °C, resulting in a white-purple mixture. The solvent was removed under reduced pressure and the residue was dissolved in CH_2Cl_2. The mixture was filtered through celite and the solvent was removed under reduced pressure. The oily residue was washed with Et_2O, which was then decanted. The resulting solid was recrystallised from CH_2Cl_2/*n*-pentane, filtered, and washed with additional *n*-pentane to yield a pale-yellow powder (36 mg, 30%). Pale-yellow, X-ray quality, prism-shaped crystals were grown by slow diffusion of *n*-pentane into a saturated CH_2Cl_2 solution. ^{1}H NMR (400 MHz, $CDCl_3$): δ/ppm = 7.93 (d, J = 7.8 Hz, 1H, H_e), 7.78 (d, J = 8.2 Hz, 1H, H_b), 7.68 (t, J = 7.6 Hz, 1H, H_f), 7.63 (t, J = 7.5 Hz, 1H, H_a), 4.87 (q, J = 7.3 Hz, 2H, H_h), 1.65 (t, J = 7.3 Hz, 3H, H_i). ^{13}C{^{1}H} NMR (101 MHz, $CDCl_3$): δ/ppm = 141.93 (C_d), 133.77 (C_c), 128.27 (C_f), 126.62 (C_a), 123.19 (C_e), 115.04 (C_b), 50.93 (C_h), 14.95 (C_i). HRMS (ESI/QTOF) m/z: [M + CH_3CN – Br^-]$^+$ Calcd for $C_{11}H_{12}AuN_2S^+$ 401.0381; Found 401.0389.

General Procedure for the synthesis of NYHC−Selenium complexes (Se1^{Y}−Se4^{Y}) (Y = NR, O, S, Se)

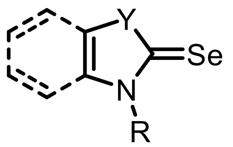

The corresponding azolium salts **1^{Y}·HX–4^{Y}·HX** (Y = NR, O, S, Se ; X = halide) (0.17 mmol, 1.0 eq.), selenium (20 mg, 0.25 mmol, 1.5 eq.) and Na^{t}BuO (19.5 mg, 0.20 mmol, 1.2 eq.), or KOAc (19.6 mg, 0.20 mmol, 1.2 eq.) for **Se1^{O}**, **Se2^{O}** and **Se3^{O}**, in THF (1 mL) were stirred at room temperature under an inert atmosphere for 16 h. The solution was filtered and the solvent was removed under reduced pressure. The residue was analysed without further purification.

1,3-dimethyl-1,3-dihydro-2H-imidazole-2-selenone (**Se1^{NMe}**). ^{77}Se{^{1}H} NMR (76 MHz, Chloroform-*d*): δ/ppm = −3. HRMS (ESI/QTOF) m/z: [M + H]$^{+}$ Calcd for $C_5H_9N_2Se^+$ 176.9925; Found 176.9930.

3-methyloxazole-2(3H)-selenone (**Se1^{O}**). ^{77}Se{^{1}H} NMR (76 MHz, Chloroform-*d*): δ/ppm = *not found*. HRMS (ESI/QTOF) m/z: *not found*.

3-methylthiazole-2(3H)-selenone (**Se1^{S}**). ^{77}Se{^{1}H} NMR (76 MHz, Chloroform-*d*): δ/ppm = 331. HRMS (ESI/QTOF) m/z: [M + H]$^{+}$ Calcd for $C_4H_6NSSe^+$ 179.9381; Found 179.9387.

1,3-dibenzyl-1,3-dihydro-2H-imidazole-2-selenone (**Se2^{NBn}**). ^{77}Se{^{1}H} NMR (76 MHz, Chloroform-*d*): δ/ppm = 3. HRMS (ESI/QTOF) m/z: [M + H]$^{+}$ Calcd for $C_{17}H_{17}N_2Se^+$ 329.0551; Found 329.0556.

3-benzyloxazole-2(3H)-selenone (**Se2^{O}**). ^{77}Se{^{1}H} NMR (76 MHz, Chloroform-*d*): δ/ppm = *not found*. HRMS (ESI/QTOF) m/z: [M + H]$^{+}$ Calcd for $C_{10}H_{10}NOSe^+$ 239.9922; Found 239.9922.

3-benzylthiazole-2(3H)-selenone (**Se2^{S}**). ^{77}Se{^{1}H} NMR (76 MHz, Chloroform-*d*): δ/ppm = 320. HRMS (ESI/QTOF) m/z: [M + H]$^{+}$ Calcd for $C_{10}H_{10}NSSe^+$ 255.9694; Found 255.9700.

1,3-diethyl-1,3-dihydro-2H-benzo[d]imidazole-2-selenone (**Se3^{NEt}**). ^{77}Se{^{1}H} NMR (76 MHz, Chloroform-*d*): δ/ppm = 45. HRMS (ESI/QTOF) m/z: [M + H]$^{+}$ Calcd for $C_{11}H_{15}N_2Se^+$ 255.0395; Found 255.0393.

3-ethylbenzo[d]oxazole-2(3H)-selenone (**Se3^{O}**). ^{77}Se{^{1}H} NMR (76 MHz, Chloroform-*d*): δ/ppm = 152. HRMS (ESI/QTOF) m/z: [M + H]$^{+}$ Calcd for $C_9H_{10}NOSe^+$ 227.9922; Found 227.9925.

3-ethylbenzo[o]thiazole-2(3H)-selenone (**Se3^{S}**). ^{77}Se{^{1}H} NMR (76 MHz, Chloroform-*d*): δ/ppm = 402. HRMS (ESI/QTOF) m/z: [M + H]$^{+}$ Calcd for $C_9H_{10}NSSe^+$ 243.9694; Found 243.9697.

3-ethylbenzo[o]selenazole-2(3H)-selenone (**Se3^{Se}**). ^{77}Se{^{1}H} NMR (76 MHz, Chloroform-*d*): δ/ppm = 668 (benzoselenazole), 510 (C=Se). HRMS (ESI/QTOF) m/z: *not found*.

1,3-dibenzyl-1,3-dihydro-2H-benzo[d]imidazole-2-selenone (**Se4^{NBn}**). ^{77}Se{^{1}H} NMR (76 MHz, Chloroform-*d*): δ/ppm = 67. HRMS (ESI/QTOF) m/z: $[M + H]^+$ Calcd for $C_{21}H_{19}N_2Se^+$ 379.0708; Found 379.0708.

3-benzylbenzo[d]thiazole-2(3H)-selenone (**Se4^{S}**). ^{77}Se{^{1}H} NMR (76 MHz, Chloroform-*d*): δ/ppm = 415. HRMS (ESI/QTOF) m/z: $[M + H]^+$ Calcd for $C_{14}H_{12}NSSe^+$ 305.9850; Found 305.9849.

3-benzylbenzo[d][1,3]selenazole-2(3H)-selenone (**Se4^{Se}**). ^{77}Se{^{1}H} NMR (76 MHz, Chloroform-*d*): δ/ppm = 705 (benzoselenazole), 517 (C=Se). HRMS (ESI/QTOF) m/z: $[M + H]^+$ Calcd for $C_{14}H_{12}NSe_2^+$ 353.9295; Found 353.9300.

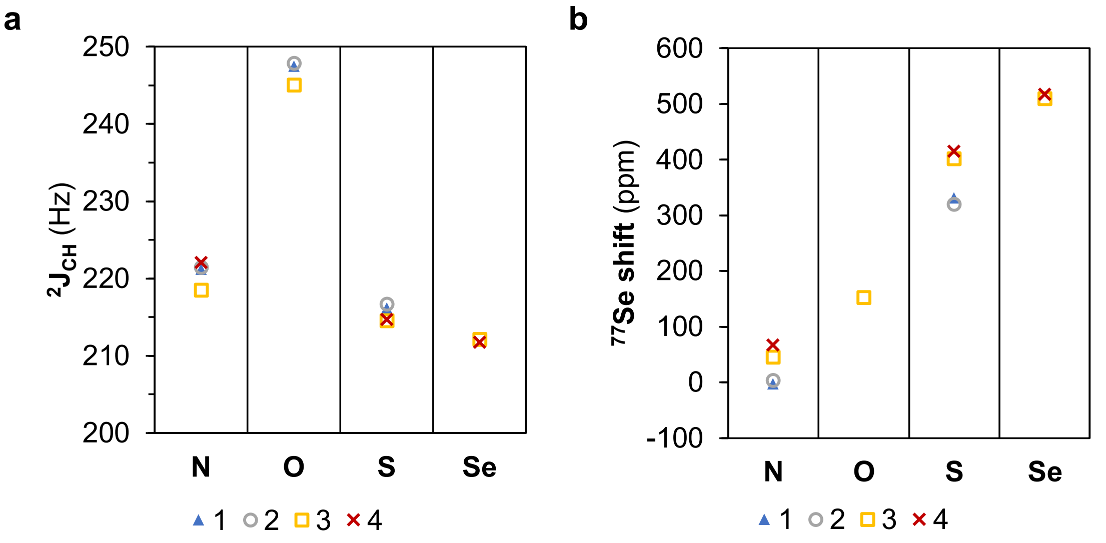

Supplementary Figure 5. $^{1}J_{HC}$ values of azolium proligands **1^{Y}·HX–4^{Y}·HX** (**a**) and ^{77}Se NMR chemical shift of selenium adducts **Se1^{Y}–Se4^{Y}** (Y = NR, O, S, Se) (**b**). The σ-donor strength was extracted from the one bond coupling constant, $^{1}J_{CH}$, of the carbene carbon atom of **1^{Y}·HX–4^{Y}·HX** (Y = NR, O, S or Se; X = halide) in d_6-DMSO.[20,21] The extent of σ-donation of the ligands is slightly lower in (N,O)HCs than in classical (N,N)HCs ($^{1}J_{HC}$ = 246.8 ± 1.5 Hz vs $^{1}J_{HC}$ = 220.8 ± 1.6 Hz), whereas (N,S)HCs ($^{1}J_{HC}$ = 215.5 ± 1.5 Hz) and (N,Se)HCs ($^{1}J_{HC}$ = 211.7 Hz) are slightly stronger σ-donors. The π-backbonding properties of **Se1^{Y}–Se4^{Y}** (Y = NR, O, S Se) were assessed using a method based on the ^{77}Se NMR chemical shift, $δ_{Se}$.[22] Based on the magnitude of $δ_{Se}$, the π-accepting properties of the carbene ligands increases according to the following sequence N (28 ± 34 ppm) < O (152 ppm) < S (367 ± 48 ppm) < Se (514 ppm).

General procedure for the synthesis of Rh(NYHC)(CO)$_2$Cl (Rh1^{Y}–Rh4^{Y}) (Y = NR, O, S, Se)

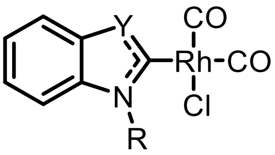

The corresponding azolium proligand **1^{Y}·HX-4^{Y}·HX** (Y = NR, O, S, Se ; X = halide) (0.17 mmol, 1.0 eq.), $[Rh_2(CO)_4Cl_2]$ (32.9 mg, 0.20 mmol, 0.6 eq.) and $Na^{t}BuO$ (19.5 mg, 0.20 mmol, 1.2 eq.), or KOAc (19.6 mg, 0.20 mmol, 1.2 eq.) for **$Rh1^{O}$**, **$Rh2^{O}$** and **$Rh3^{O}$**, in THF (1 mL) were refluxed under an inert atmosphere for 12 h. The solvent was removed under reduced pressure and the residue was analysed without further purification. TEP was from using the formula $TEP = 0.8001[\overline{v_{CO}}(Rh)] + 420$, where $\overline{v_{CO}}(Rh)$ is the average CO bond stretch.

Supplementary Table 3. Symmetric, asymmetric and average CO bond IR stretching bands of **$Rh1^{Y}$–$Rh4^{Y}$** (Y = NR, O, S, Se) and calculated TEP values for carbenes **1^{Y}–4^{Y}** (Y = NR, O, S, Se).

	**v**$_{CO}$ (sym.) (cm^{-1})	**v**$_{CO}$ (asym.) (cm^{-1})	**Avg. v**$_{CO}$ (cm^{-1})	**TEP** (cm^{-1})	
**$Rh1^{NMe}$**	2074	2003	2039	**1^{N}**	2051.0
**$Rh1^{O}$**	2085	2002	2044	**1^{O}**	2055.0
**$Rh1^{S}$**	2087	2014	2051	**1^{S}**	2060.6
**$Rh2^{NBn}$**	2082	2007	2045	**2^{N}**	2055.8
**$Rh2^{O}$**	2085	2009	2047	**2^{O}**	2057.8
**$Rh2^{S}$**	2086	2012	2049	**2^{S}**	2059.4
**$Rh3^{NEt}$**	2063	2019	2041	**3^{N}**	2053.0
**$Rh3^{O}$**	2079	2026	2053	**3°**	2062.2
**$Rh3^{S}$**	2084	2022	2053	**3^{S}**	2062.6
**$Rh3^{Se}$**	2089	2021	2055	**3^{Se}**	2064.2
**$Rh4^{NBn}$**	2083	2002	2043	**4^{N}**	2054.2
**$Rh4^{S}$**	2083	2032	2058	**4^{S}**	2066.2
**$Rh4^{Se}$**	2097	2022	2060	**4^{Se}**	2067.8

2.5. Partition coefficients

The *n*-octanol-water partition coefficient ($logP_{OW}$) was determined using the Shake-flask method[23] with quantification using inductively coupled plasma mass spectrometry (ICP-MS) on a Perkin Elmer NexIon 350 D. The partition coefficient was calculated using the equation: $P_{ow} = [M]_{org_f} / \left([M]_{org_i} - [M]_{org_f}\right)$, where $[M]_{org_i}$ and $[M]_{org_f}$ are the concentrations of metal in the organic phase before

and after mixing with the aqueous phase, respectively. Measurements were performed in duplicates.

3. Computational details

DFT calculations were performed using the ORCA quantum chemistry package 5.0.2[24] Geometry optimisation and subsequent calculations were performed at the B3LYP level of theory using the theory using the def2-TZVPP basis set with SARC and ZORA relativistic corrections.[25–27] The initial geometries of the carbenes **1^Y^–4^Y^** (Y = NR. O, S, Se) and proligands **1^Y·HX-4^Y·HX** (Y = NR. O, S, Se ; X = halide) were generated from the single crystal X-ray diffraction structures of the corresponding complexes and, for those missing, by replacing the corresponding atoms in other optimised structure. The initial geometries of the complexes **$Pt1^Y$**, **$Pt2^Y$**, **$Au3^Y$** and **$Ru4^Y$** (Y = NR, O, S, Se) were generated from the experimentally determined structures. All the optimised geometries were verified as minima by the absence of imaginary frequencies. The results were analysed using Multiwfn 3.7 and representations were generated using Avogadro 1.2.0.[28] TEP values were obtained from the ESP energy values of local minima (V_{min}), (3,+3) critical points (CPs), of the electrostatic potential (ESP) surface of the σ-shaped lone pair of the carbene carbon atom of **1^Y–4^Y** using the equation $TEP_{comp} = 0.5939V_{min} + 2101.3\ cm^{-1}$.[29] Lone pair energies and σ-hole energies were obtained from the ESP energy values of the local minima (V_{min}) (3,+3 CPs) and local maxima (V_{max}) of the electron localisation function (ELF) and ESP surfaces of **1^Y·HX-4^Y·HX** (Y = NR. O, S, Se ; X = halide), respectively. These energies correlate to the strength of hydrogen and chalcogen bonds.[30,31] Log*P* values were calculated using a previously developed approach.[32] AIM charge at the metal and electron occupancy in the M–C_{NHC} bond were estimated from the integration of the electron density in the metal and M–C_{NHC} bond basins calculated from the AIM and ELF functions, respectively. Binding energies (E_b) were calculated according to the equation $E_b = E_{complex} - E_{NHC} - E_{metal}$, where $E_{complex}$ is the total energy of the metal complexes **$Pt1^Y$**, **$Pt2^Y$**, **$Au3^Y$** and **$Ru4^Y$** (Y = NR, O, S, Se), E_{NHC} is the total energies of the free carbene **1^Y-4^Y** (Y = NR. O, S, Se), and E_{metal} is the total energy the remaining metal fragment, i.e. $PtPyrI_2$, AuI, $RuCymCl_2$.

Supplementary Table 4. Energy of the ESP surface at the local minima corresponding to the carbene lone pair (V_{min}), calculated TEP (TEP_{comp}), aromaticity descriptor HOMA, and energy of the ESP surface at the lone pair(s) and σ-hole(s) of **1^Y–4^Y** (Y = NR, O, S, Se).

	V_{min} (kcal/mol)	TEP_{comp} (cm^{-1})	HOMA	Lone pair (kcal/mol)		σ-hole (kcal/mol)	
**1**^{NMe}	-80.71	2053.4	0.784				
**1**^{O}	-71.97	2058.6	0.087	490.6			
**1**^{S}	-70.80	2059.3	0.655	393.3		122.5	128.1
**1**^{Se}	-68.54	2060.6	-	219.7	219.1	124.6	128.8
**2**^{NBn}	-77.29	2055.4	0.771				
**2**^{O}	-71.18	2059.0	0.081	483.0			
**2**^{S}	-69.87	2059.8	0.653	384.8		121.8	116.1
**2**^{S}	-67.79	2061.0	-	208.0	207.4	122.7	118.4
**3**^{NEt}	-76.51	2055.9	0.800				
**3**^{O}	-69.70	2059.9	0.154	475.5			
**3**^{S}	-69.21	2060.2	0.653	361.4	362.9	104.5	118.9
**3**^{Se}	-67.67	2061.1	-	192.4	192.0	115.3	119.6
**4**^{NBn}	-73.28	2057.8	0.791				
**4**^{O}	-68.41	2060.7	0.153	475.4			
**4**^{S}	-67.89	2061.0	0.652	358.5	359.4	106.7	116.7
**4**^{Se}	-66.31	2061.9	-	185.2	185.8	116.4	111.6

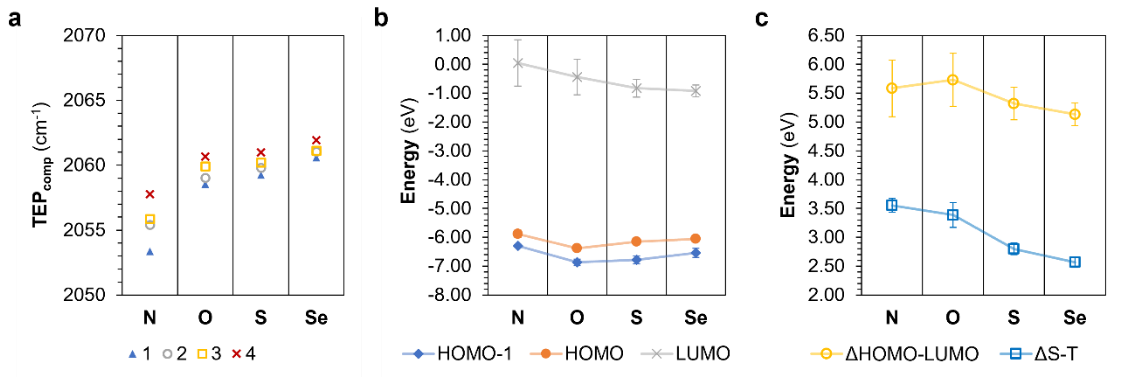

Supplementary Figure 6. Computational TEP (**a**) and HOMO-1, HOMO, and LUMO energy levels (**b**), and HOMO-LUMO ($\Delta_{HOMO-LUMO}$) and single-triplet gap (Δ_{S-T}) (**b**) of **1**^{Y}–**4**^{Y} (Y = NR, O, S, Se) calculated at the B3LYP/def2-TZVPP level. See Supplementary Table S4 for the corresponding energy levels. The HOMO and LUMO energy of **1**^{Y}–**4**^{Y} (Y = NR, O, S, Se) correlated well with the experimentally measured σ-donating and π-accepting character of the ligands.[33] The HOMOs of the (N,Y)HCs are σ-symmetric, with the singlet lone pair localized on the carbene carbon atom and in the plane of the heterocycle, whereas the LUMOs π-type orbitals localized at the carbene carbon atom. The HOMO-LUMO gap (Δ_{gap}) of the (N,O)HCs is slightly larger in energy than the (N,N)HCs and decreases with the incorporation of the heavier chalcogens. The same trend was observed for the HOMO-1 energy and, therefore, for the π-

donating ability of the ligands. Additionally, the singlet-triplet energy gap also decreases with the incorporation of the chalcogen atom, however the singlet state is still favoured.

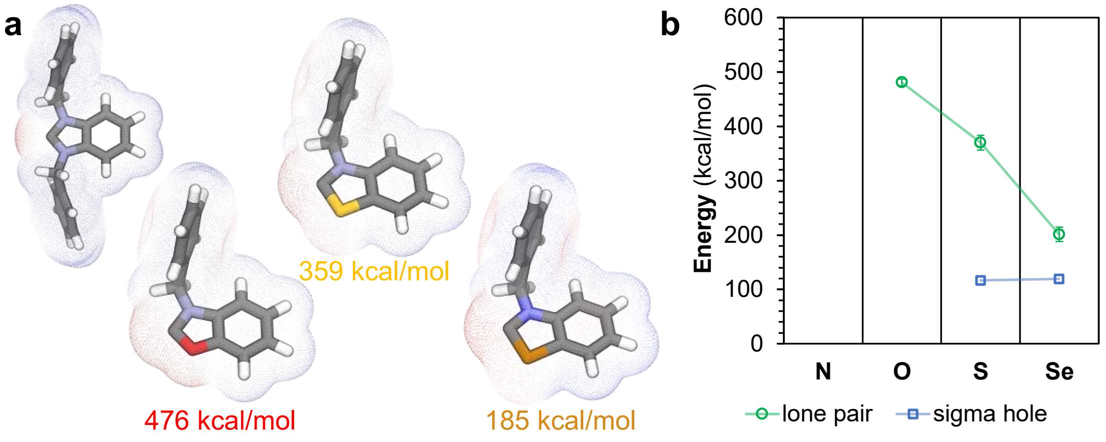

Supplementary Figure 7. Representation of the electrostatic surface potential map of **4**^{Y} (Y = NBn, O, S, Se) (**a**), and calculated energies of the lone pair and sigma holes of **1**^{Y}–**4**^{Y} (Y = NR, O, S, Se) (**b**) calculated at the B3LYP/def2-TZVPP level.

Supplementary Table 5. Energy levels of the π-donor (HOMO-1), σ-donor (HOMO) and π-acceptor (lowest unoccupied orbital with the appropriate symmetry) orbitals, HOMO-LUMO gap (Δ_{Gap}), and singlet-triplet gap (Δ_{S-T}) of **1**^{Y}–**4**^{Y} (Y = NR. O, S, Se) calculated at the B3LYP/def2-TZVPP level.

	**π-donor** (eV)	**σ-donor** (eV)	**π-acceptor** (eV)	**π-acceptor orbital**[a]	**Δ**$_{Gap}$ (eV)	**Δ**$_{S-T}$ (kcal/mol)
**1**^{NMe}	−6.242	−5.726	0.591	LUMO	6.317	83.7
**1**^{O}	−6.973	−6.343	0.073	LUMO	6.416	78.5
**1**^{S}	−6.926	−6.122	−0.432	LUMO	5.690	66.4
**1**^{Se}	−6.693	−6.042	−0.671	LUMO	5.371	60.6
**2**^{NBn}	−6.307	−5.852	0.880	LUMO+5	5.359	84.7
**2**^{O}	−6.961	−6.257	0.118	LUMO+2	5.536	84.9
**2**^{S}	−6.890	−6.135	−0.744	LUMO	5.391	66.7
**2**^{Se}	−6.653	−6.048	−0.834	LUMO	5.214	61.3
**3**^{NEt}	−6.28	−5.904	−0.634	LUMO	5.270	79.7
**3**^{O}	−6.757	−6.432	−0.976	LUMO	5.456	74.2
**3**^{S}	−6.662	−6.146	−1.068	LUMO	5.078	62.7
**3**^{Se}	−6.400	−6.045	−1.085	LUMO	4.960	57.5
**4**^{NBn}	−6.351	−6.044	−0.654	LUMO	5.390	79.5
**4**^{O}	−6.777	−6.484	−0.972	LUMO	5.512	74.6

**4^{S}**	−6.668	−6.197	−1.078	LUMO	5.119	62.4
**4^{Se}**	−6.415	−6.094	−1.11	LUMO	4.984	57.4

[a] Due to required orbital symmetry.

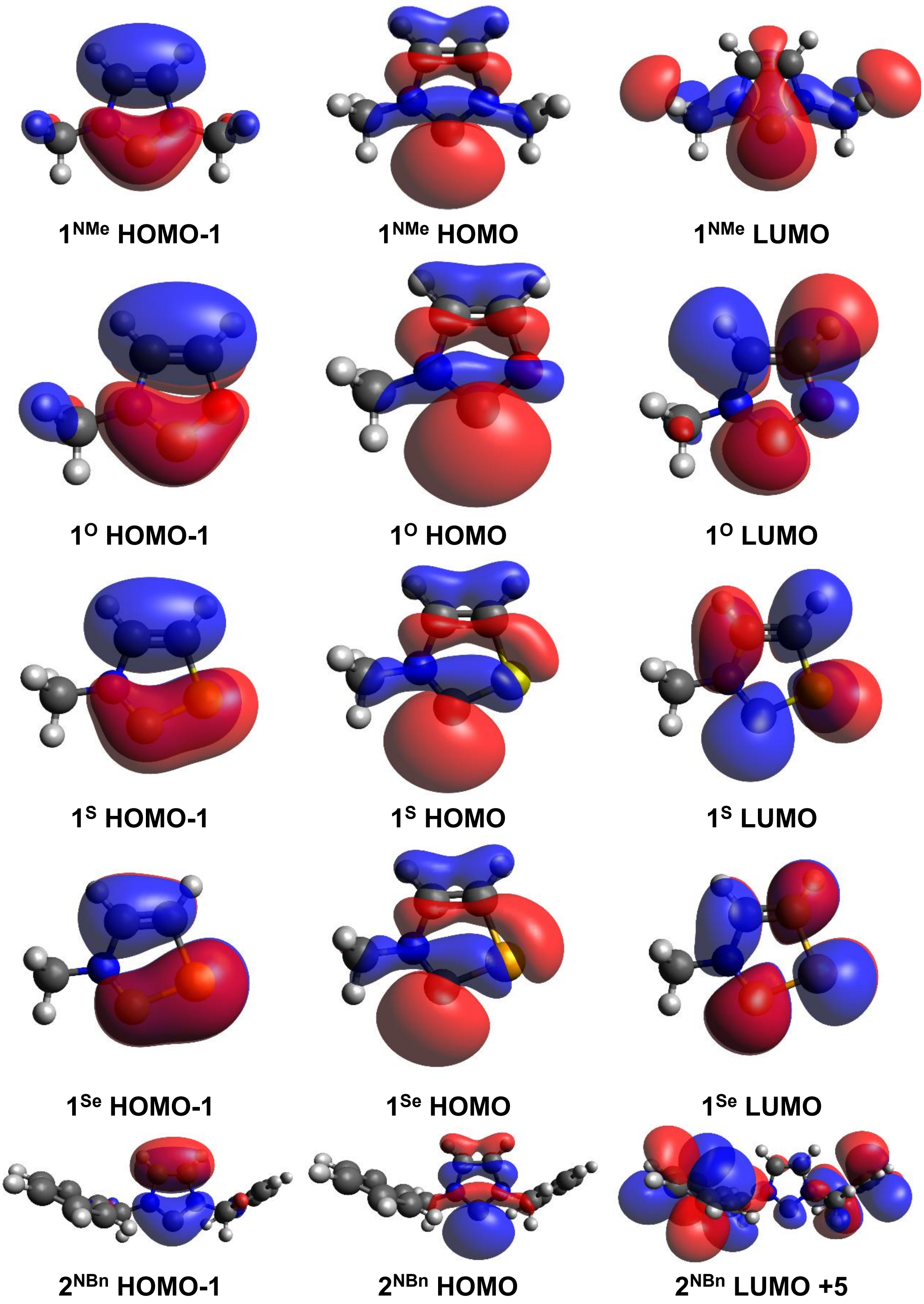

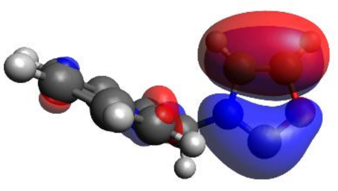

2^{O} HOMO-1

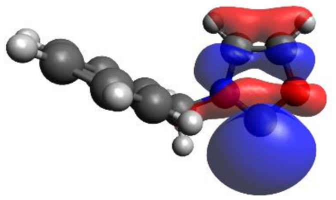

2^{O} HOMO

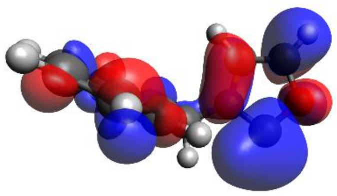

2^{O} LUMO +2

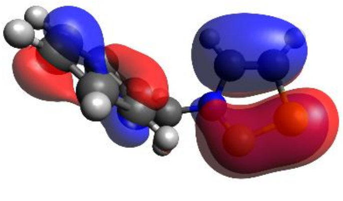

2^{S} HOMO-1

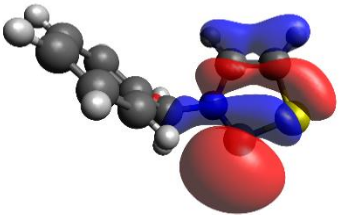

2^{S} HOMO

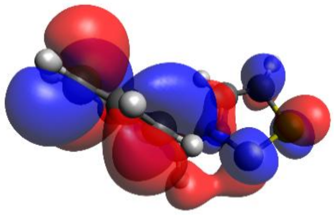

2^{S} LUMO

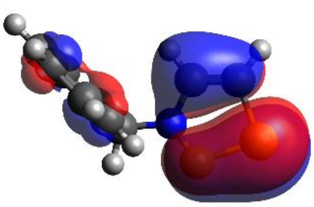

2^{Se} HOMO-1

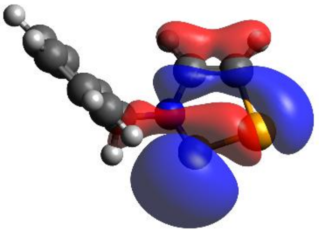

2^{Se} HOMO

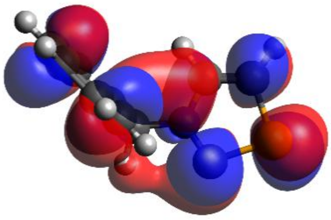

2^{Se} LUMO

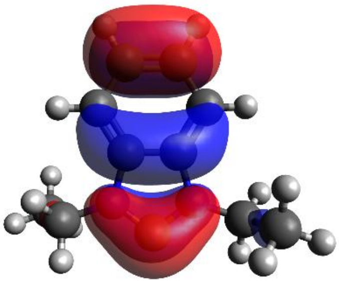

3^{NEt} HOMO-1

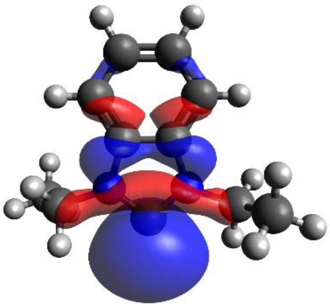

3^{NEt} HOMO

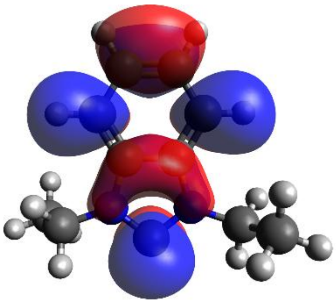

3^{NEt} LUMO

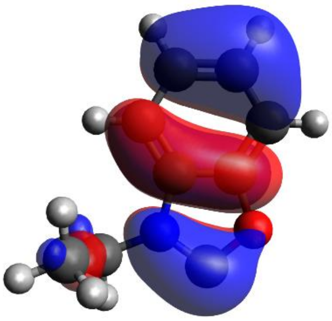

3° HOMO-1

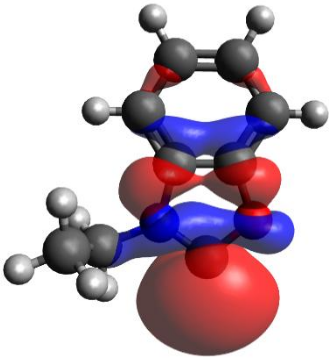

3° HOMO

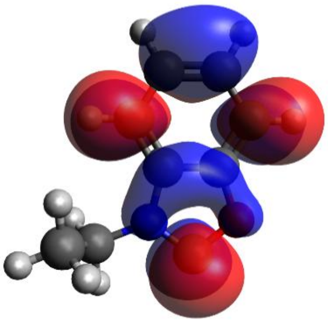

3° LUMO

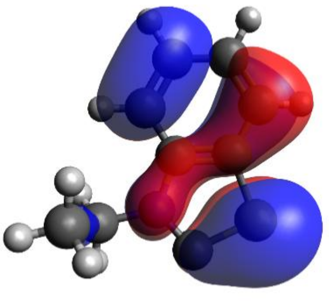

3^{S} HOMO-1

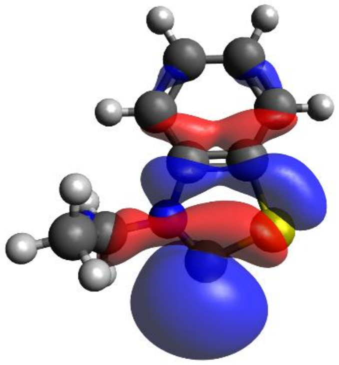

3^{S} HOMO

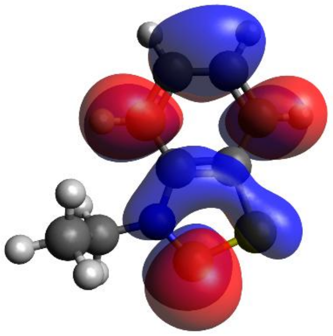

3^{S} LUMO

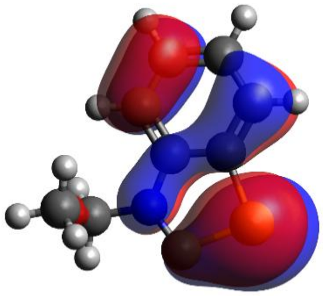

3^{Se} HOMO-1

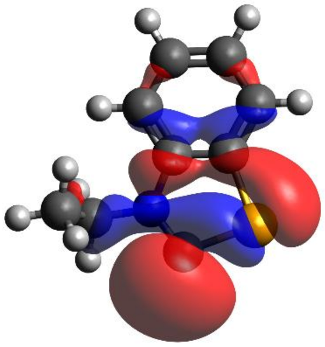

3^{Se} HOMO

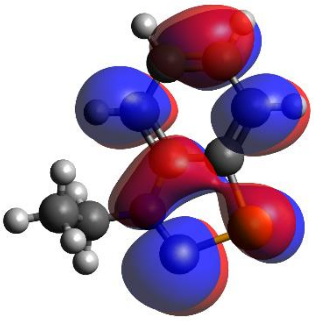

3^{Se} LUMO

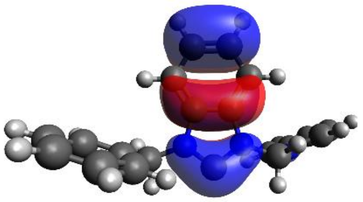

4^{NBn} HOMO-1

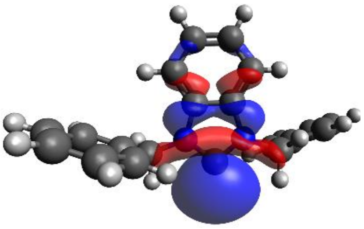

4^{NBn} HOMO

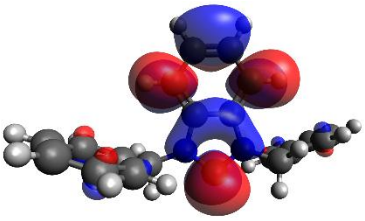

4^{NBn} LUMO

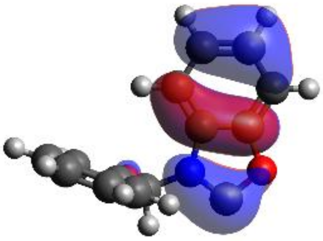

4° HOMO-1

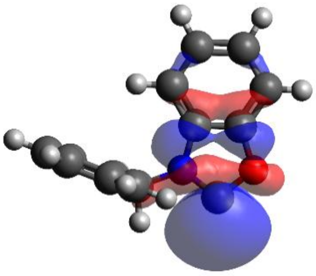

4° HOMO

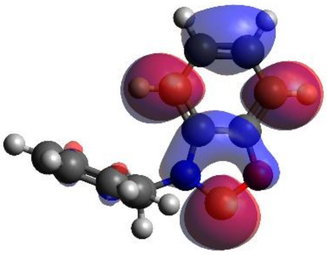

4° LUMO

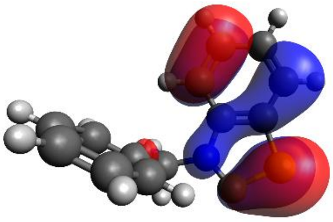

4^{S} HOMO-1

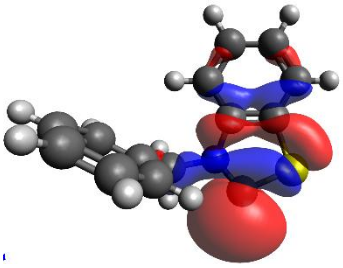

4^{S} HOMO

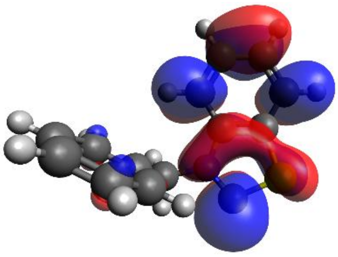

4^{S} LUMO

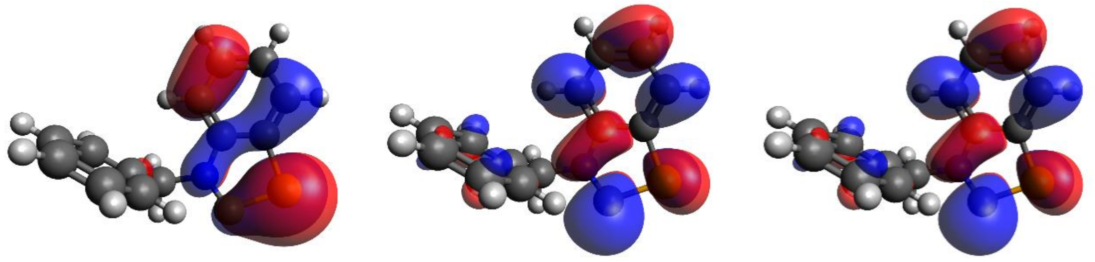

Supplementary Figure 8. Graphical representation of the π-donor (HOMO-1), σ-donor (HOMO) and π-acceptor (lowest unoccupied orbital with the appropriate symmetry) frontier orbitals of **1**^{Y}–**4**^{Y} (Y = NR, O, S, Se). See Supplementary Table S5 for the corresponding energy levels.

Supplementary Table 6. Calculated M–C_{NYHC} bond lengths, electron occupancy of the M–C_{NYHC} bond calculated from the ELF, charge at the metal atom from the electron density donation from the carbene to the metal estimated by AIM analysis, and calculated log*P* of complexes **Pt1**^{Y}, **Pt2**^{Y}, **Au3**^{Y} and **Ru4**^{Y} (Y = NR, O, S, Se).

	**M–C_{NYHC}** (calc.)	**M–C_{NYHC} occupancy** (e^-)	**AIM charge** (e^-)	**log*P*** (calc.)
**Pt1**^{NMe}	1.971	2.249	0.263	2.14
**Pt1**^{O}	1.947*	2.328	0.287	1.85
**Pt1**^{S}	1.957	2.231	0.278	1.83
**Pt1**^{Se}	1.949	2.237	0.280	1.65
**Pt2**^{NBn}	1.979	2.240	0.259	3.85
**Pt2**^{O}	1.949	2.322	0.290	2.68
**Pt2**^{S}	1.962	2.224	0.277	2.67
**Pt2**^{Se}	1.954	2.229	0.276	2.49
**Au3**^{NEt}	2.021	2.308	0.046	1.94
**Au3**^{O}	2.003*	2.376	0.088	1.39
**Au3**^{S}	2.011	2.281	0.073	1.43
**Au3**^{Se}	2.007	2.292	0.074	1.23
**Ru4**^{NBn}	2.098	2.274	0.916	4.63
**Ru4**^{O}	2.023	2.353	0.929	3.61
**Ru4**^{S}	2.048	2.247	0.920	3.70
**Ru4**^{Se}	2.042	2.246	0.921	3.55

* The experimental M–C_{NYHC} bond length in the (N,O)HCs is significantly longer than the predicted values, which could be due to the higher disorder of the crystal structures of **Pt1**^{O} and **Au3**^{O}. However, the trend in the electron occupancy hints at a weaker bond between the (N,O)HCs ligands and the metal, which would result in a longer bond length. The electron

occupancy in the M-C_{NYHC} bond could be indicative of the bond order. The charge at the metal atom calculated by AIM analysis indicates the enhanced electron donation of the (N,Y)HCs to the metal centres.[34].

Supplementary Table 7. HOMO and LUMO energy levels, HOMO-LUMO gap (Δ_{Gap}), and M–NHC binding energy of complexes **Pt1**^{Y}, **Pt2**^{Y}, **Au3**^{Y} and **Ru4**^{Y} (Y = NR, O, S, Se).

	**HOMO** (eV)	**LUMO** (eV)	Δ_{Gap} (eV)	**Binding energy** (eV)
**Pt1**^{NMe}	−5.340	−1.638	3.70	−3.21
**Pt1**^{O}	−5.394	−1.730	3.66	−3.07
**Pt1**^{S}	−5.422	−1.745	3.68	−3.02
**Pt1**^{Se}	−5.455	−1.768	3.68	−3.05
**Pt2**^{NBn}	−5.391	−1.658	3.73	−3.21
**Pt2**^{O}	−5.424	−1.717	3.71	−3.09
**Pt2**^{S}	−5.432	−1.732	3.70	−3.02
**Pt2**^{Se}	-5.447	-1.751	3.70	−3.05
**Au3**^{NEt}	−5.643	−1.662	3.98	−2.82
**Au3**^{O}	−5.760	−2.041	3.72	−2.65
**Au3**^{S}	−5.735	−2.189	3.55	−2.64
**Au3**^{Se}	−5.745	-2.212	3.53	−2.66
**Ru4**^{NBn}	−5.371	−1.430	3.94	−1.51
**Ru4**^{O}	−5.287	−1.630	3.66	−1.34
**Ru4**^{S}	−5.328	−1.472	3.86	−1.31
**Ru4**^{Se}	−5.371	−1.530	3.84	−1.37

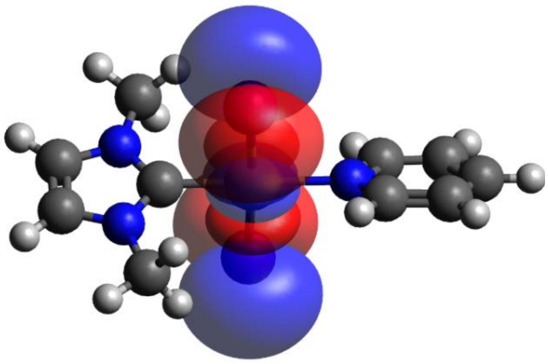

Pt1^{NMe} **HOMO**

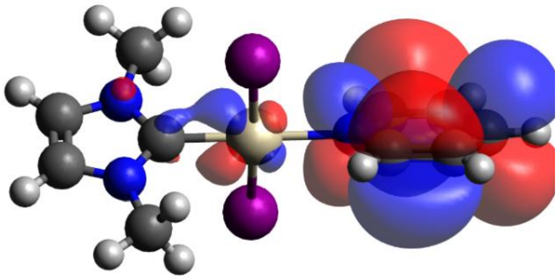

Pt1^{NMe} **LUMO**

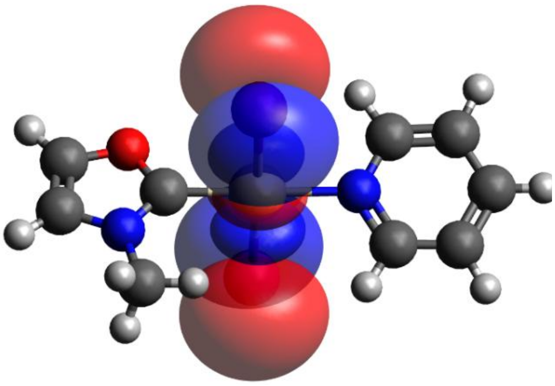

Pt1^{O} HOMO

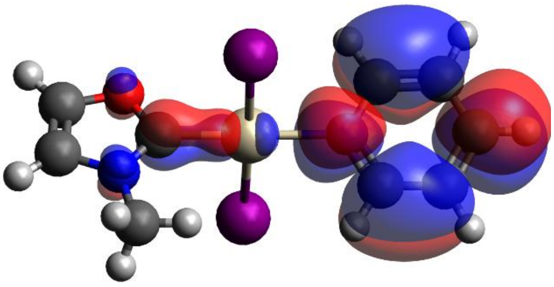

Pt1^{O} LUMO

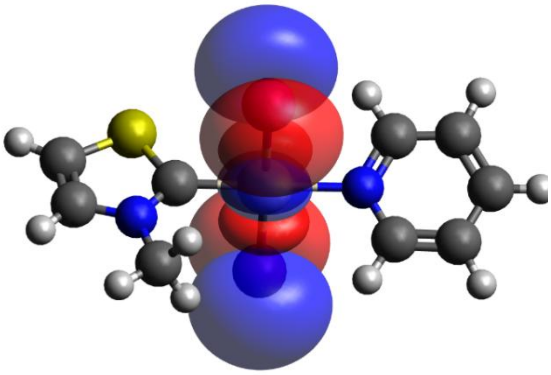

Pt1^{S} HOMO

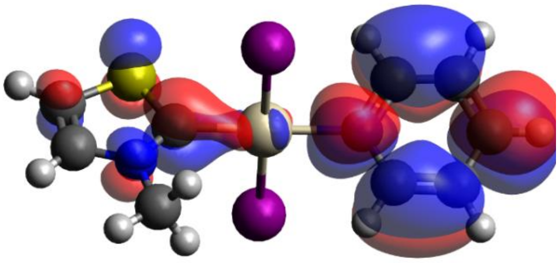

Pt1^{S} LUMO

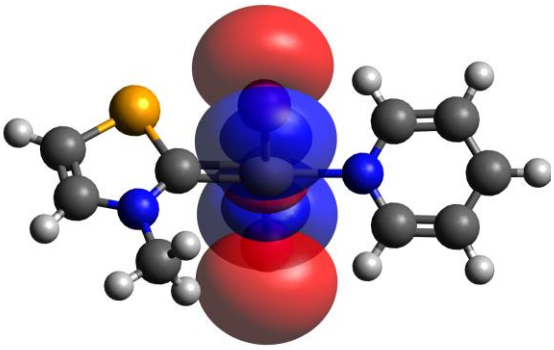

Pt1^{Se} HOMO

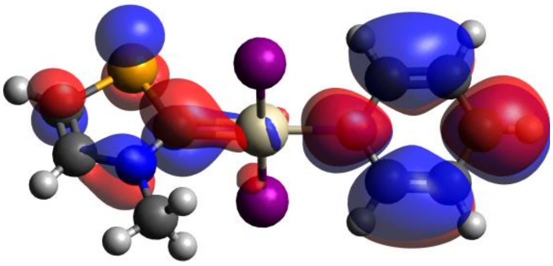

Pt1^{Se} LUMO

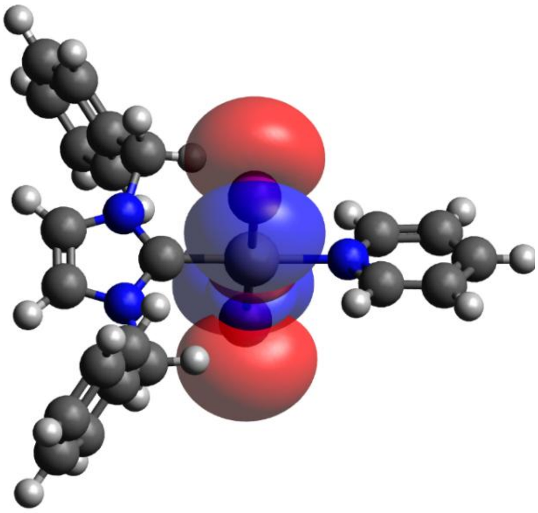

Pt2^{NBn} HOMO

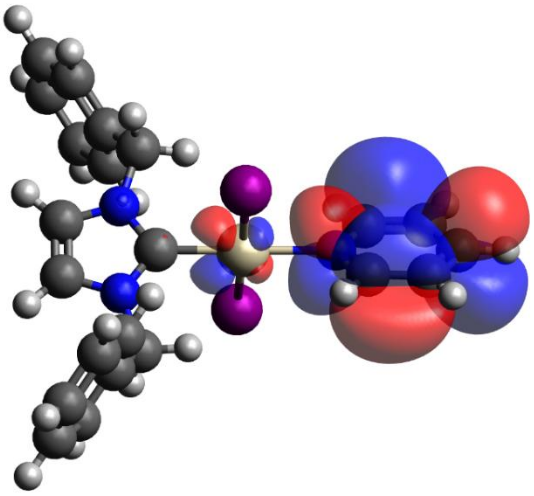

Pt2^{NBn} LUMO

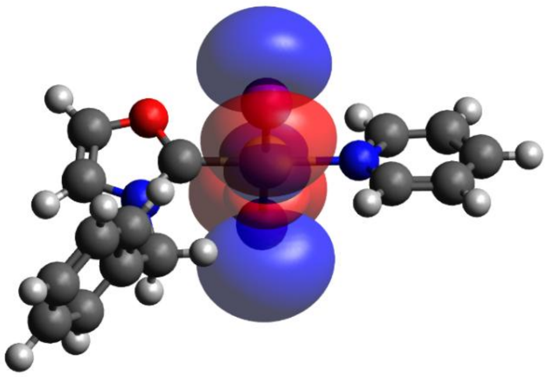

Pt2^{O} HOMO

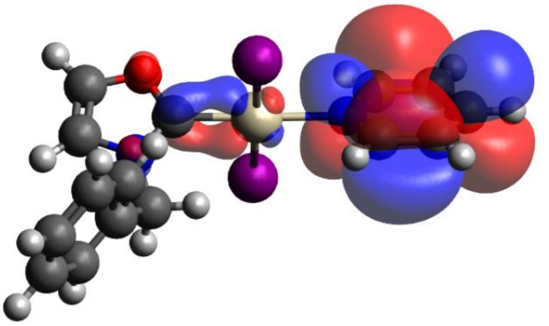

Pt2^{O} LUMO

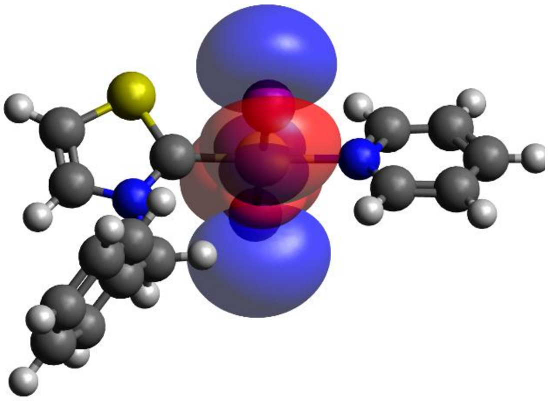

Pt2^{S} HOMO

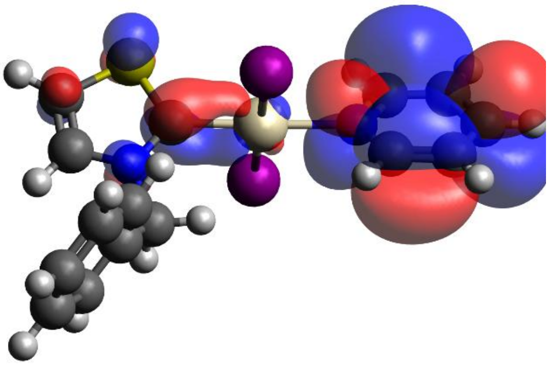

Pt2^{S} LUMO

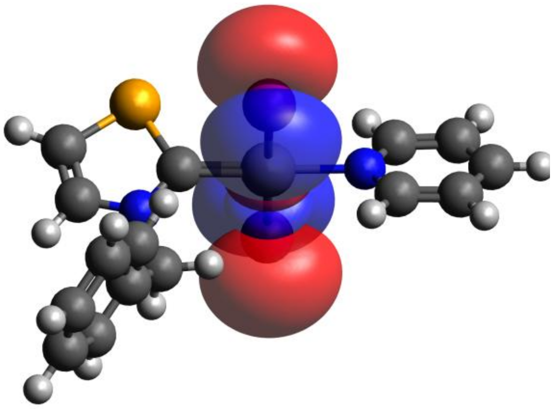

Pt2^{Se} HOMO

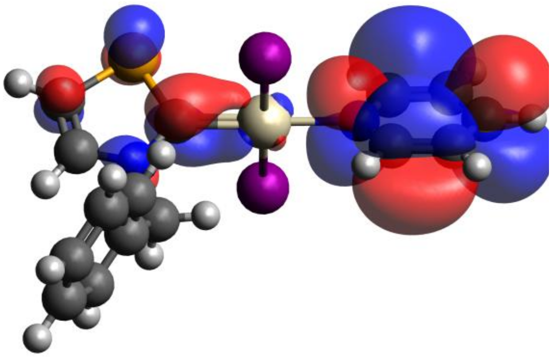

Pt2^{Se} LUMO

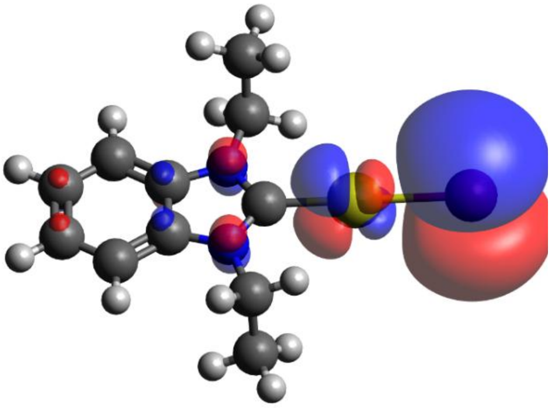

Au3^{NEt} HOMO

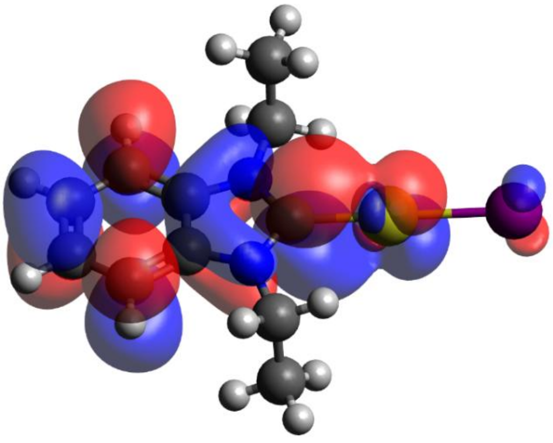

Au3^{NEt} LUMO

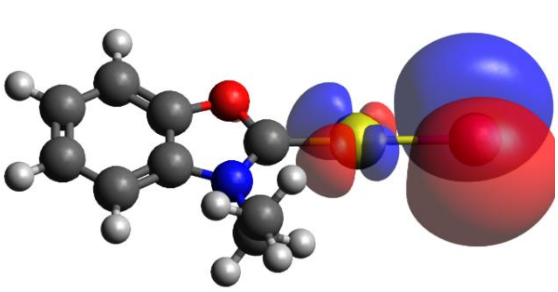

Au3^{O} HOMO

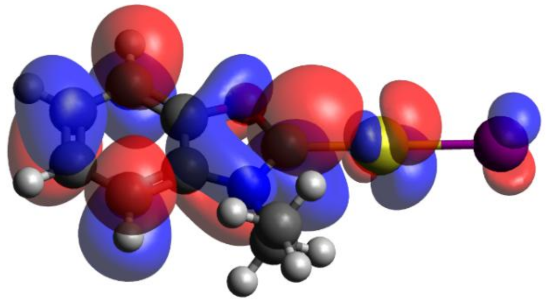

Au3^{O} LUMO

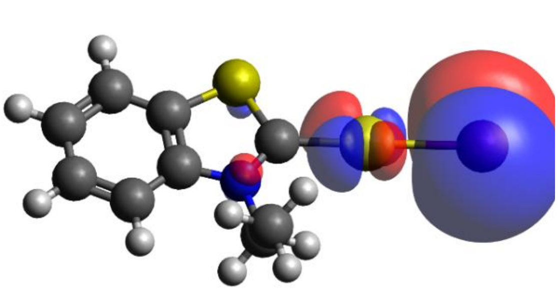

Au3^{S} HOMO

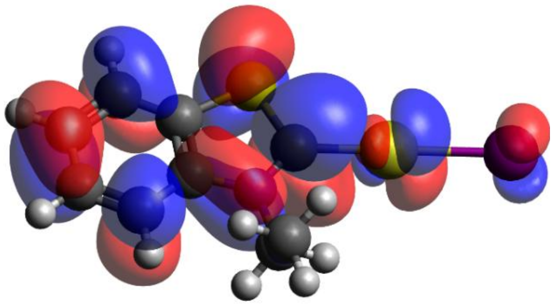

Au3^{S} LUMO

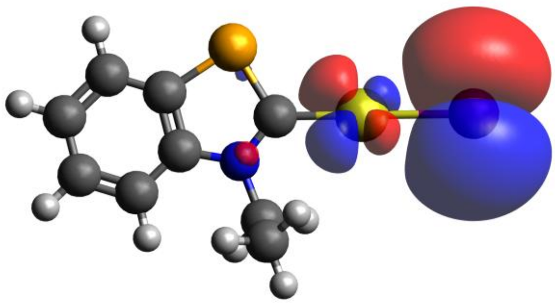

Au3^{Se} HOMO

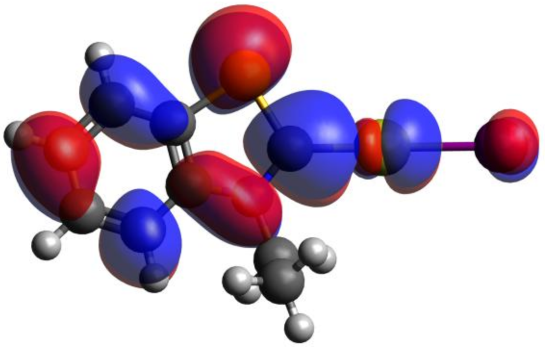

Au3^{Se} LUMO

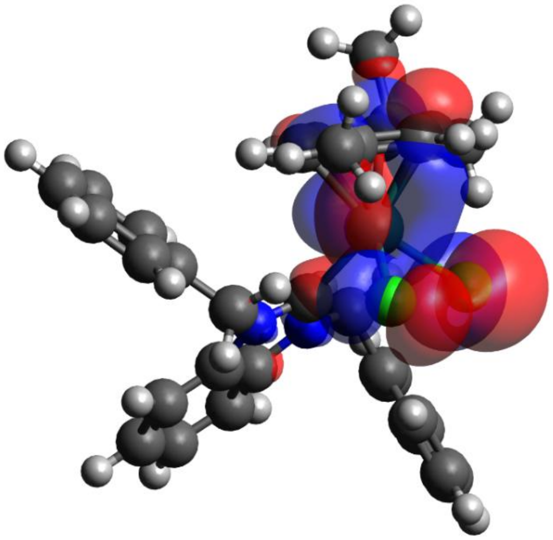

Ru4^{NBn} HOMO

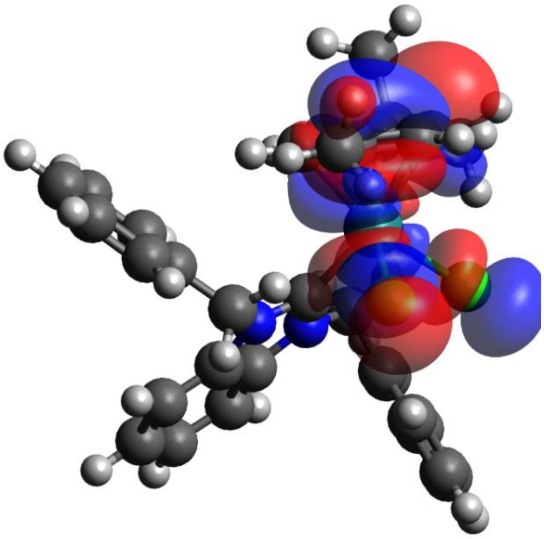

Ru4^{NBn} LUMO

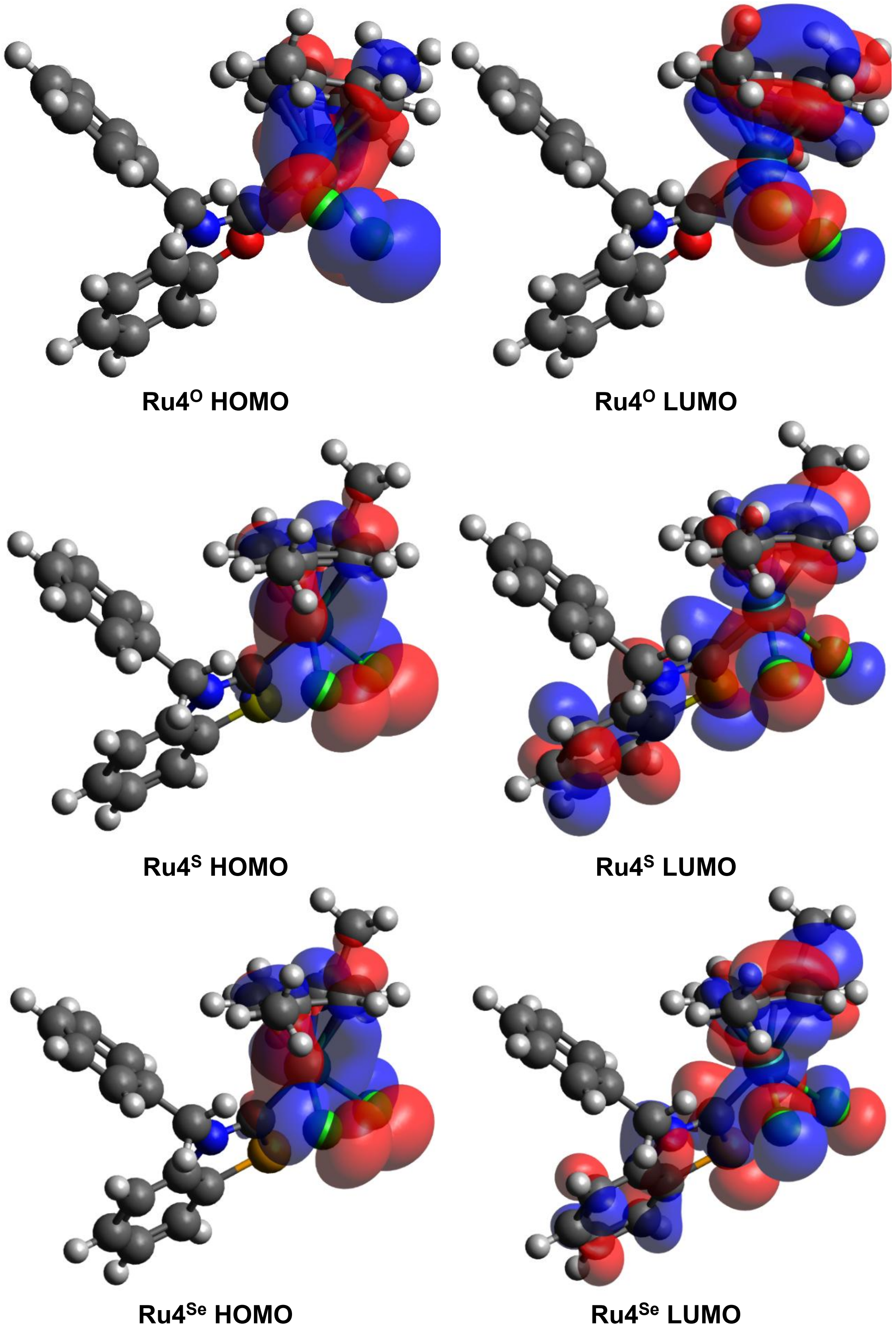

Supplementary Figure 9. Graphical representation of the frontier orbitals of complexes **Pt1^{Y}**, **Pt2^{Y}**, **Au3^{Y}** and **Ru4^{Y}** (Y = NR, O, S, Se). See Supplementary Table S6 for the corresponding energy levels.

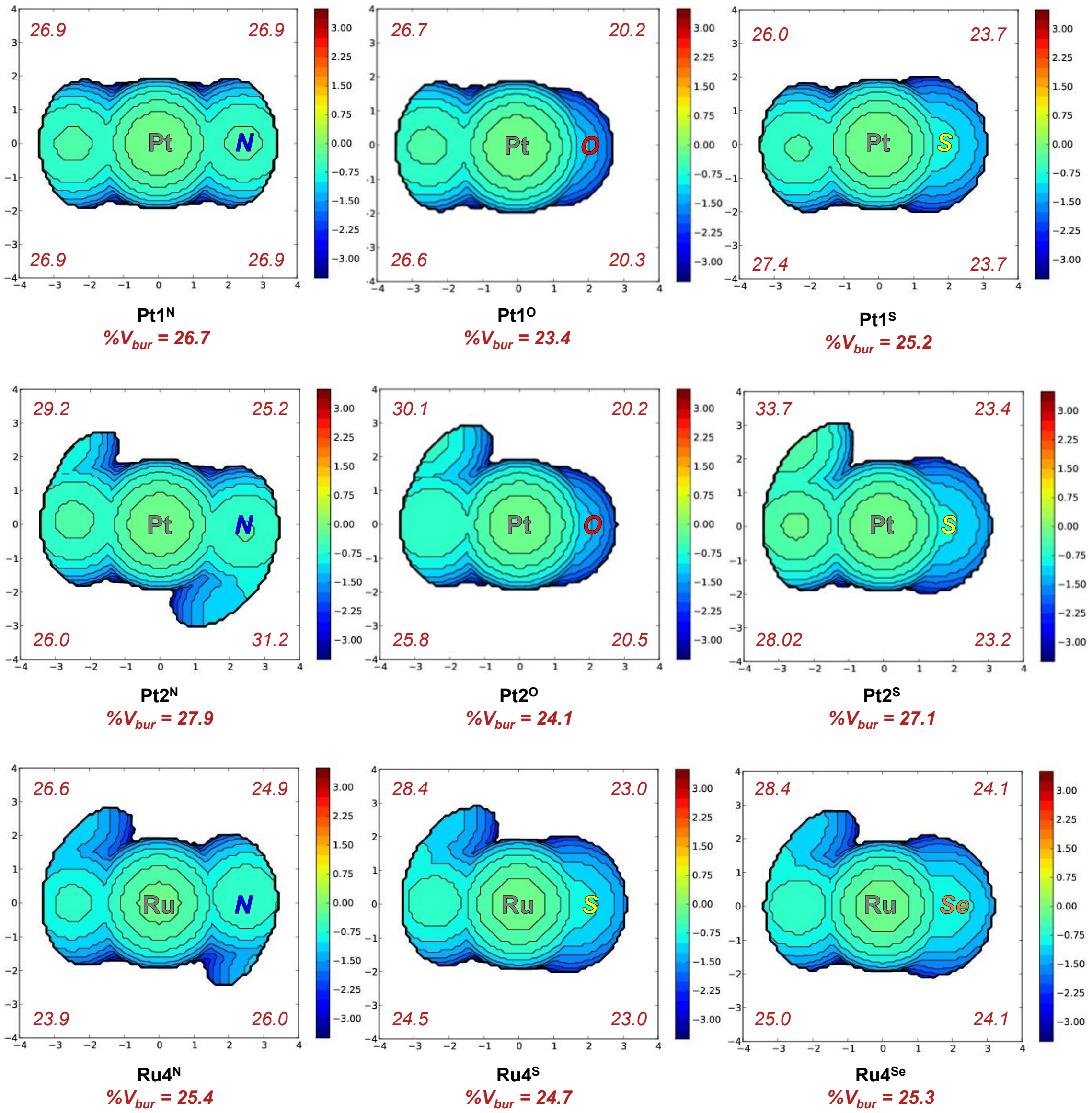

Supplementary Figure 10. Steric map of **Pt1^{Y}**, **Pt2^{Y}** and **Ru4^{Y}** (Y = NR, O, S, Se) showing total and per quadrant percentage of buried volume (%V_{bur}).

4. *In vitro* studies

4.1. Cell culture

The human ovarian carcinoma cell line, A2780, and its cisplatin-resistant counterpart, A2780cis, were procured from the European Collection of Cell Cultures (ECACC, UK) and were maintained in RPMI-1640 GlutaMAX (ThermoFisher, USA) base medium supplemented with 10% Foetal Bovine Serum (FBS; Sigma, Germany) and 1% of Penicillin-Streptomycin (ThermoFisher, USA). Additionally, A2780cis cell line was cultured in the presence of 1 μM cisplatin every three passages to maintain resistance to this drug. The human embryonic kidney cell line, HEK293T, and HeLa cell line were obtained from American Type Culture Collection (ATCC, USA) and were cultured in DMEM GlutaMAX (ThermoFisher, USA) base medium, supplemented with 10% FBS and 1% of Penicillin-Streptomycin. All cell lines were maintained in a humidified incubator at37 °C and 5% CO_2.

4.2. Cytotoxicity studies

100 μl of cell suspension at concentration of $7{\cdot}10^4$ cells/mL (A2780), $1.4{\cdot}10^5$ cells/mL (A2780cis and HEK293T) was dispensed into flat bottom 96-well plates and allowed to attach overnight. On the next day, 10 mM stock solutions of the tested compounds were prepared in DMSO, then diluted with media to the final concentration range of 0 - 100 μM and applied to the cells; 0.1% DMSO final solution and 10 μM final concentration gambogic acid were used as negative and positive controls, respectively. After 72 h incubation in a humidified incubator under 5% CO_2 at 37 °C, PrestoBlue™ cell viability reagent (Cat. No. A13262, Invitrogen, USA) was added to cultures at a 1:10 ratio according to the manufacturer instructions, and samples were incubated for 1.5 h. Fluorescence at 560/590 nm was detected with Tecan Infinite 500 microplate reader (Tecan, Switzerland). Three wells were assessed for each concentration of the tested compounds and three independent experiments were performed. Non-linear regression and IC_{50} value calculation were performed in GraphPad Prism 9 using the build-in function “Dose response- Inhibition”.

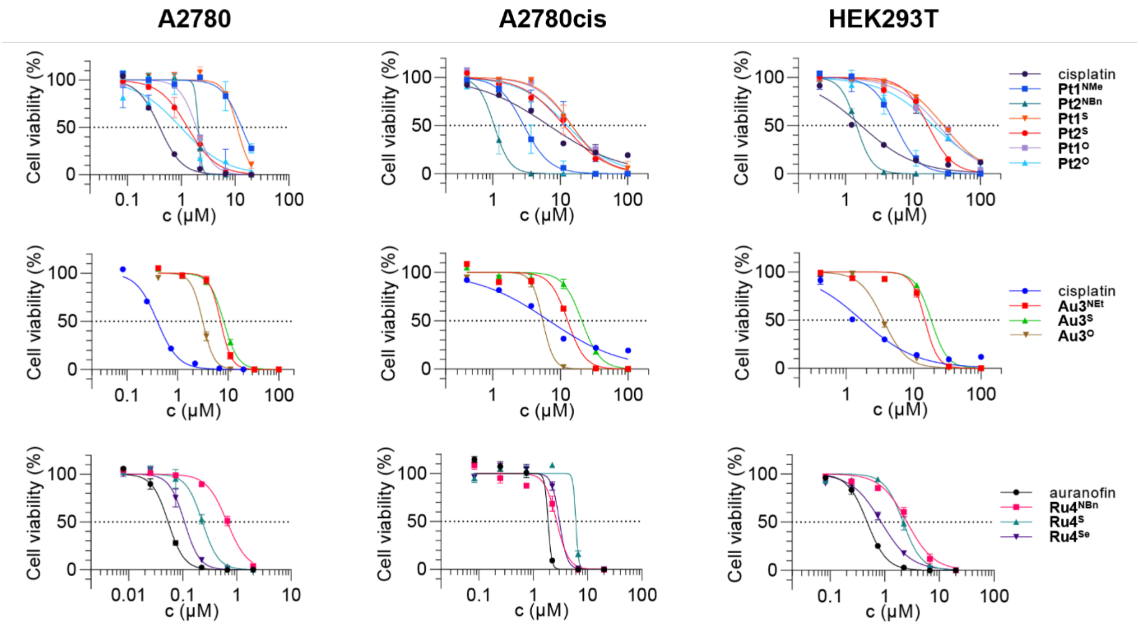

Supplementary Figure 11. Dose-response curves of **Pt1**^{Y}, **Pt2**^{Y}, **Au3**^{Y} and **Ru4**^{Y} (Y = NR, O, S, Se) after 72 h of incubation.

Supplementary Table 8. Cytotoxicity of azolium (N,Y)HC proligands **1**^{Y}**·HX-4**^{Y}**·HX** (Y = NR, O, S or Se; X = halide) against ovarian cancer (A2780 and A2780cis), and non-tumorigenic healthy kidney (HEK293T) cells after 72 h of incubation.

Compound	IC$_{50}$ (µM) after 72 h		
	**A2780**	**A2780cis**	**HEK293T**
**1**^{NMe}**·HI**	> 100	> 100	> 100
**1**^{O}**·HI**	11.8	39.8 ± 2.6	59.1 ± 16.6
**1**^{S}**·HI**	> 100	> 100	> 100
**2**^{NBn}**·HBr**	37.4	44.1	51.1 ± 12.4
**2**^{O}**·HBr**	> 100	> 100	> 100
**2**^{S}**·HBr**	58.6	73.5	> 100
**3**^{NEt}**·HI**	> 100	> 100	> 100
**3**^{O}**·HI**	> 100	> 100	> 100
**3**^{S}**·HI**	> 100	> 100	> 100
**3**^{Se}**·HI**	> 100	> 100	> 100
**4**^{NBn}**·HBr**	15.2 ± 12.1	24.4 ± 11.7	35.2 ± 26.3
**4**^{S}**·HBr**	34.7	37.9 ± 1.7	36.8 ± 19.3
**4**^{Se}**·HBr**	13.5 ± 5.9	28.4 ± 8.0	20.5 ± 14.2

4.3. Protein inhibition assays

A fluorometric assay kit was used to determine Cathepsin B activity (Cat. No. ab65300, Abcam, USA). Briefly, A2780 cells were seeded into 6-well plates at a density of $1.2 \cdot 10^6$ cells/mL and allowed to adhere overnight. Subsequently, they were treated with either 5 μM **Au3**^{Y} (Y = NEt, O, S, Se) or 50 μM **Ru4**^{Y} (Y = NBn, S, Se). After 72 h incubation the cell lysates were collected, and the protein concentration was determined by Bradford assay (Abcam). Then, the reactions were set up according to the manufacturer protocol, including the treated and untreated samples, as well as negative and background controls. Fluorescence was detected at Ex/Em 400 nm/505 nm with Tecan Infinite 500 microplate reader (Tecan, Switzerland). Three independent experiments were performed.

A colorimetric assay kit was used to determine the activity of Thioredoxin Reductase (TrxR) (Cat. No. ab83463, Abcam, USA). Briefly, A2780 cells were seeded into 6 well plates at a concentration $2 \cdot 10^5$ cells per well. The next day the cells were treated with the tested compounds at 1 μM **Au3**^{Y} (Y = NEt, O, S, Se) or 10 μM **Ru4**^{Y} (Y = NBn, S, Se) concentration. An equal volume of DMSO was used as a control. After 72 h incubation, the cells were washed 2 times with PBS and homogenized in 150 μL of TrxR assay buffer (ab83463, Abcam, UK) supplemented with protease inhibitor cocktail (MedChemExpress, USA) on ice. The lysates were incubated in ice for 20 min, then centrifuged at 10,000 g for 15 min at 4 °C. The supernatants were transferred to a new tube and stored at -80 °C. The concentration of the lysates was measured by Bradford reagent (Abcam) and equimolar dilutions of the lysates were prepared. The TrxR activity assay was performed according to the manufacturer protocol. The absorbance at 412 nm was detected using Tecan Spark (Tecan, Switzerland) plate reader at 25 °C. The data was analysed using Excel and GraphPad Prism 10. Three independent experiments were performed.

4.4. EdU incorporation assay

EdU incorporation assay was performed using a Click-iT EdU Imaging kit (C10086, Thermo Fisher, Switzerland) according to the manufacturer's protocol. HeLa cells were seeded into a black 96-well PhenoPlate with optically clear flat-bottom (Revity 6055302) at a concentration of 4000 cells per well. After 24 h, the cells were treated with the tested compounds **Pt1**^{Y} and **Pt2**^{Y} (Y = NR, O, S),

cisplatin and transplatin (between 0.3 and 60 μM, concentrations below the IC_{50} of the compounds in HeLA cells). Gemcitabine (1 and 3 μM) and an equal volume of DMSO were used as positive and negative control, respectively. Each condition was tested in triplicate. The assay was performed after an incubation of 24 h. The cells were additionally stained by Hoechst 33342 diluted in PBS with 3% BSA. The fluorescent signal was detected with the widefield INCell 2200 microscope (GE health care, USA) using a 10X/0.45 objective. The DAPI channel was used for Hoechst 33342 emitting blue (λ_{ex} = 390/ 18 nm, λ_{em} = 432.5/ 48 nm), and the FITC channel for Edu-AlexaFluor-488 (λ_{ex} = 475/ 28 nm, λ_{em} = 511.5/23 nm) emitting in green. For each well, 9 fields of view were captured. Image analysis was performed using CellProfiler (v4.2.5), CellProfiler Analyst (v3.0.4) and FIJI (v1.54k) software. Images were first pre-processed (correction for flat-field illumination using a Gaussian-based smoothing filter and rescaled for segmentation). All cells were segmented using the minimum cross-entropy thresholding method on an intermediate image composed of the addition of 2 channels imaged. For all cells, features of size, shape, and fluorescence intensities were extracted. Cells were then either classified directly as live or dead based on a combination of their size and EdU staining intensity or classified automatically as live or dead using a machine learning-based random forest classifier model in a multiparametric approach. The readout used is the average percentage of live or dead cells in each condition and the values are normalised to gemcitabine at 3 μM and DMSO (*untreated*) with the corresponding volume at 3 μM.

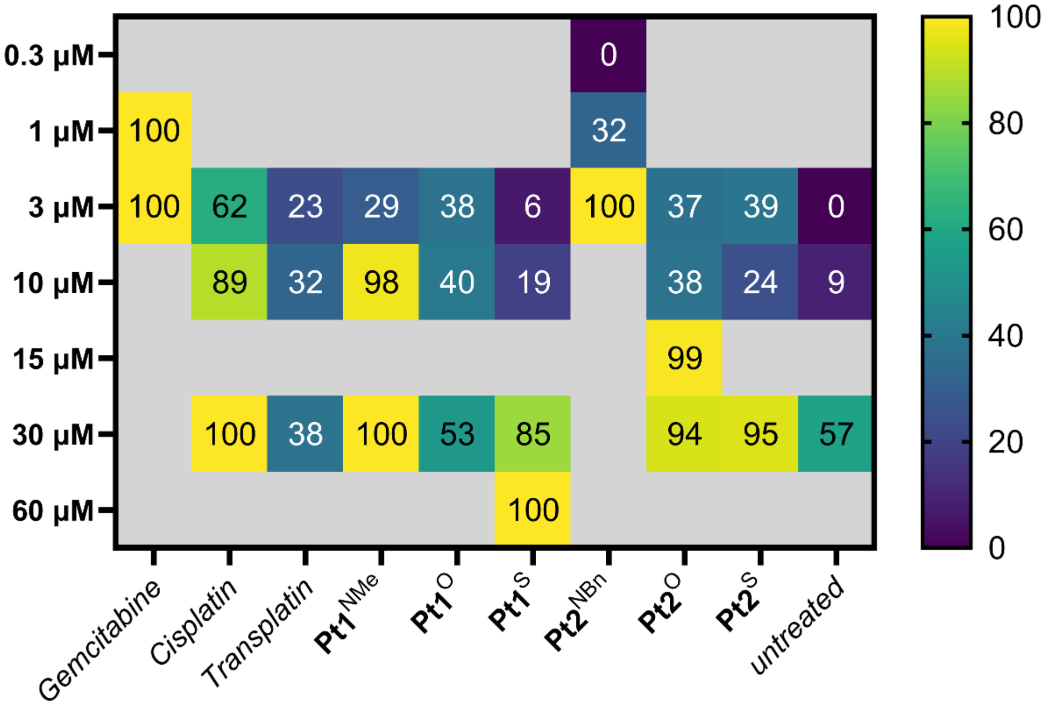

Supplementary Figure 12. EdU incorporation inhibition in HeLa cells treated with **Pt1**^{Y} and **Pt2**^{Y} (Y = NR, O, S). The observed EdU incorporation in the untreated sample is linked to the toxicity of high concentrations of DMSO to HeLa cells.

4.5. Qualitative SAR study

Qualitative SAR analysis was performed to identify major chemical, physical, structural and electronic properties of the ligand and/or complexes modulating the cytotoxicity of **Pt1**^{Y}, **Pt2**^{Y}, **Au3**^{Y} and **Ru4**^{Y} (Y = NR, O, S, Se). A two-tailed parametric Spearman correlation test (α = 0.2) was conducted between the cytotoxicity values of each cell line and 37 experimental and computational descriptors, which were normalised by centring the data to zero mean.

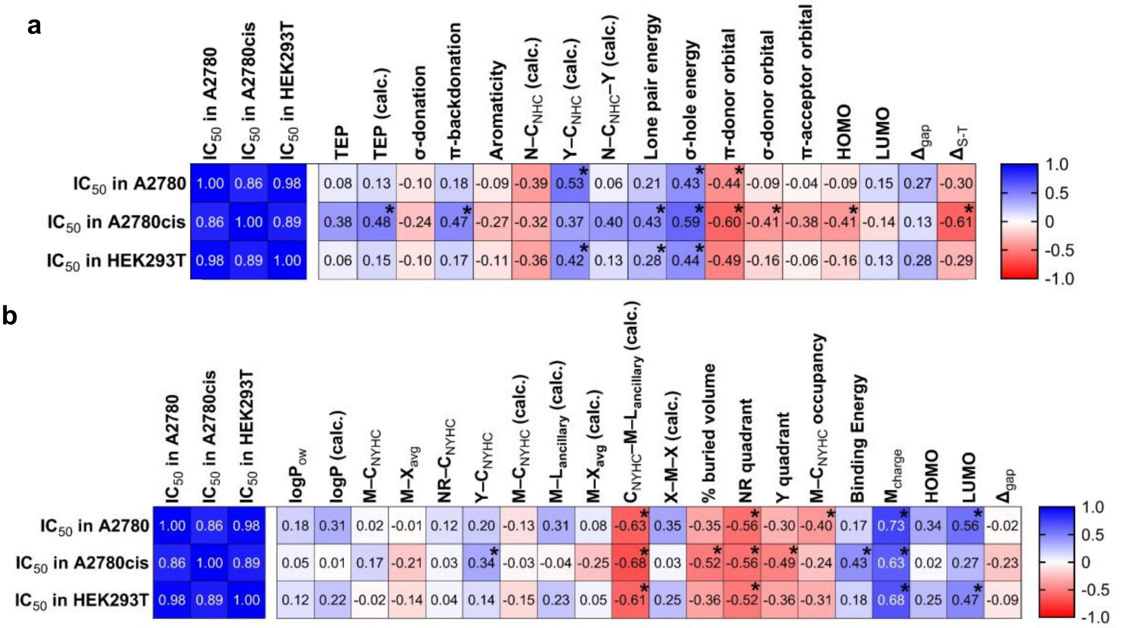

Supplementary Figure 13. Spearman correlation matrix between the IC_{50} values of metal complexes **Pt1^Y**, **Pt2^Y**, **Au3^Y** and **Ru4^Y** (Y = NR, O, S, Se) against A2780, A2780cis, and HEK293T cells and 37 experimental and computational descriptors of the azolylidene ligands **1^Y**–**4^Y** (Y = NR. O, S, Se) (**a**) or metal complexes **Pt1^Y**, **Pt2^Y**, **Au3^Y** and **Ru4^Y** (Y = NR, O, S, Se) (**b**). * Statistically significant contributions.

5. Protein crystallography

Hen egg white lysozyme (HEWL) crystals were obtained from a solution of HEWL (25 mg/mL) in Tris-HCl buffer (20 mM, pH = 8.0). Crystals of the adducts of **Ru4^{S}** and **Ru4^{Se}** with HEWL suitable for X-ray diffraction were obtained by soaking the performed protein crystals of HEWL with a solution containing the complexes in DMSO (50 mM) in a crystallization solution containing 0.8 M NaCl and 0.1 M NaOAc at pH = 4.6 for about 5–10 second.[36] The resulting crystals, **HEWL@Ru4^{Y}** (Y = S, Se), were analysed at the European Synchrotron Radiation Facility (ESRF) using a wavelength of 0.96546 Å, resulting in structures with a 1.17 and 1.37 Å resolution.

Supplementary Table 9. Crystal data, collection, phasing and refinement of HEWL@**Ru4^{Y}** (Y = S, Se) adducts.

	**HEWL@Ru4^{S}** *native*	**HEWL@Ru4^{Se}** *native*
	**Data collection**	
Wavelength (Å)	0. 96546	0.96546
Space group	P 43 21 2	P 43 21 2
a, b, c (Å)	79.19, 79.19, 37.15	78.26, 78.26, 37.16
α, β, γ (°)	90, 90, 90	90, 90, 90
Resolution (Å)*	19.21 – 1.49 (1.51 - 1.49)	18.74 – 1.17 (1.18 – 1.17)
R_{meas}*	0.078 (1.597)	0.038 (1.307)
R_{merge}*	0.074 (1.484)	0.033 (1.140)
Mean I/σI*	11.2 (0.8)	18.0 (1.2)
Completeness (%)*	99.5 (99.9)	98.8 (99.2)
Multiplicity*	8.4 (7.2)	4.3 (4.1)
CC1/2*	0.999 (0.488)	0.999 (0.557)
	**Refinement**	
Resolution (Å)	19.21 – 1.49	18.74 – 1.17
Total reflections	166978 (6987)	167116 (7839)
Total unique	19816 (966)	39289 (1926)
R_{work}#	0.1563 (0.2936)	0.1456 (0.3223)
R_{free}#	0.1871 (0.3521)	0.1824 (0.3486)

Number of non-hydrogen atoms	1170	1267
macromolecules	1049	1073
ligands	33	47
solvent	88	147
Protein residues	129	129
RMS deviations (bonds)%	0.009	0.144
RMS deviations (angles)%	1.005	4.23
Ramachandran favoured (%)%	98.43	98.43
Ramachandran allowed (%)%	1.57	1.57
Ramachandran outliers (%)%	0.00	0.00
Rotatmer outliers (%)%	0.00	0.00
Clashscore%	3.30	2.30
Average B-factor#	26.82	22.57
macromolecules	25.92	20.43
ligands	36.71	31.88
solvent	34.54	35.15
Molprobity Score%	1.12	1.11
**PDB**	**9HTJ**	**9HTI**

#as reported by phenix.refine
*as reported by autoPROC
%as reported by Molprobity

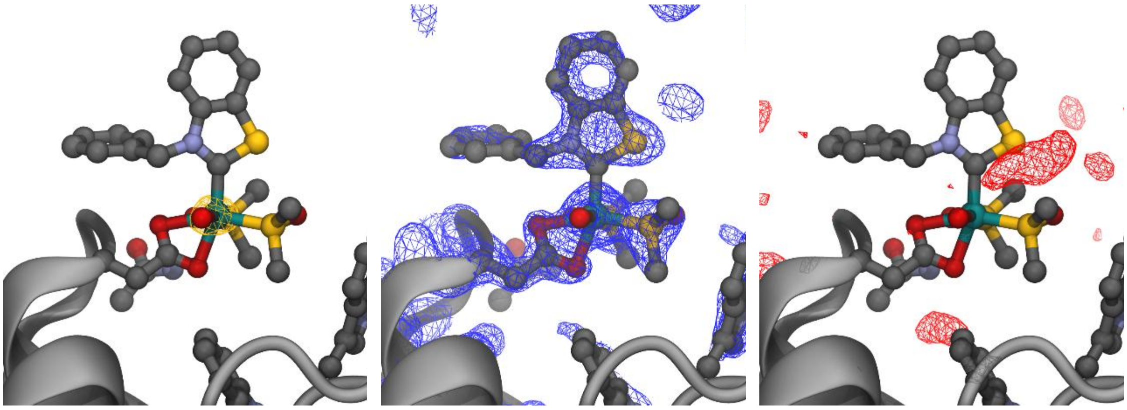

Supplementary Figure 14. Protein crystallography electron density maps of the adduct formed in the reaction of HEWL with **4Ru^{S}**. The anomalous difference map (yellow), the refined electron

density (2Fo-Fc) map (blue), and the unmodeled electron density (Fo-Fc) map (red) are contoured at 4σ, 1.5σ, and 3σ, respectively.

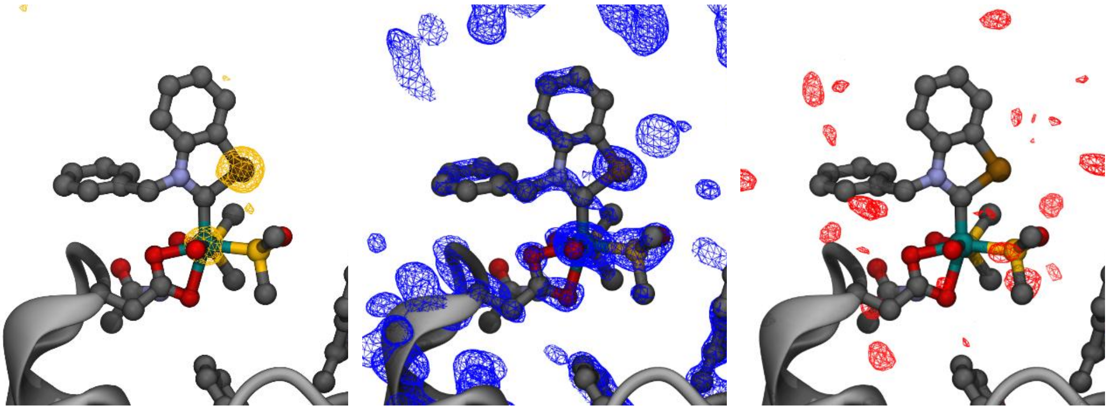

Supplementary Figure 15. Protein crystallography electron density maps of the adduct formed in the reaction of HEWL with **$4Ru^{Se}$**. The anomalous difference map (yellow), the refined electron density (2Fo-Fc) map (blue), and the unmodeled electron density (Fo-Fc) map (red) are contoured at 4σ, 1.5σ, and 3σ, respectively.

6. Molecular docking

The developed metal docking protocol is based on Autodock, an open-source multi-platform docking software.[37] It relies on the reported docking procedure for zinc ions in Zn-containing proteins and pseudopotential for covalent docking in Autodock.[38–41] The labile ligand(s) of the metal complexes were replaced with a donor dummy-atom set at 2 Å from the metal centre (shorter than a metal-nucleophile bond) in order to emulate the presence of a lone pair (LP). The metal atom parameters were modified to donor type in order to enhance the directionality of the coordination (serving as a two-point axis to force the correct bond angle between the nucleophile in the biomolecule and the metal complex). Specific atom pairwise interactions between the metal atom or the LP dummy-atom and typically coordinating atoms (N, O, S) were introduced. The pairwise interactions were defined by an equilibrium distance, R_{eq}, and a potential well between the two atoms, ε. R_{eq} was obtained from available crystallographic data, and ε was obtained from the proportion of the frequency of the metal centre binding each coordinating atom (Supplementary Figure 15).[42] Finally, clashes between hydrogen atoms in the biomolecules and the metal atom were removed. The method was validated by redocking crystallographic structures of biomolecule-metal adducts containing platinum, gold or ruthenium carbene, or *trans*-Pt complexes (Supplementary Figure 16 and Supplementary Table 11). The model scored reasonable results in terms of the prediction of the metal binding site and the position, orientation, and conformation of the ligands (RMSD of 2.9 and 4.7 Å, respectively). Overall, the developed docking protocol is able to suggest potential binding sites and conformations for the developed (N,Y)HC metal complexes. Furthermore, the approach appears to consider small structural variations between the docked complexes when proposing solutions, such as differentiating the possible interactions of oxazolylidenes and thiazolylidenes ligands with amino acids. However, the developed docking protocol appears to underscore solutions in disorganised or highly solvent exposed areas of the biomolecules and is biased toward binding sites with more surface information where the docked metal compounds can establish more complex interactions. Additionally, in complexes with ligands containing a limited number of atoms or functional groups capable of establishing classical interactions with biomolecules

(HB or π-interactions), the docking appears mostly guided by the geometry of the metal centre and the shape of the molecule, rather than by chemical interactions, which leads to a higher number of solutions.

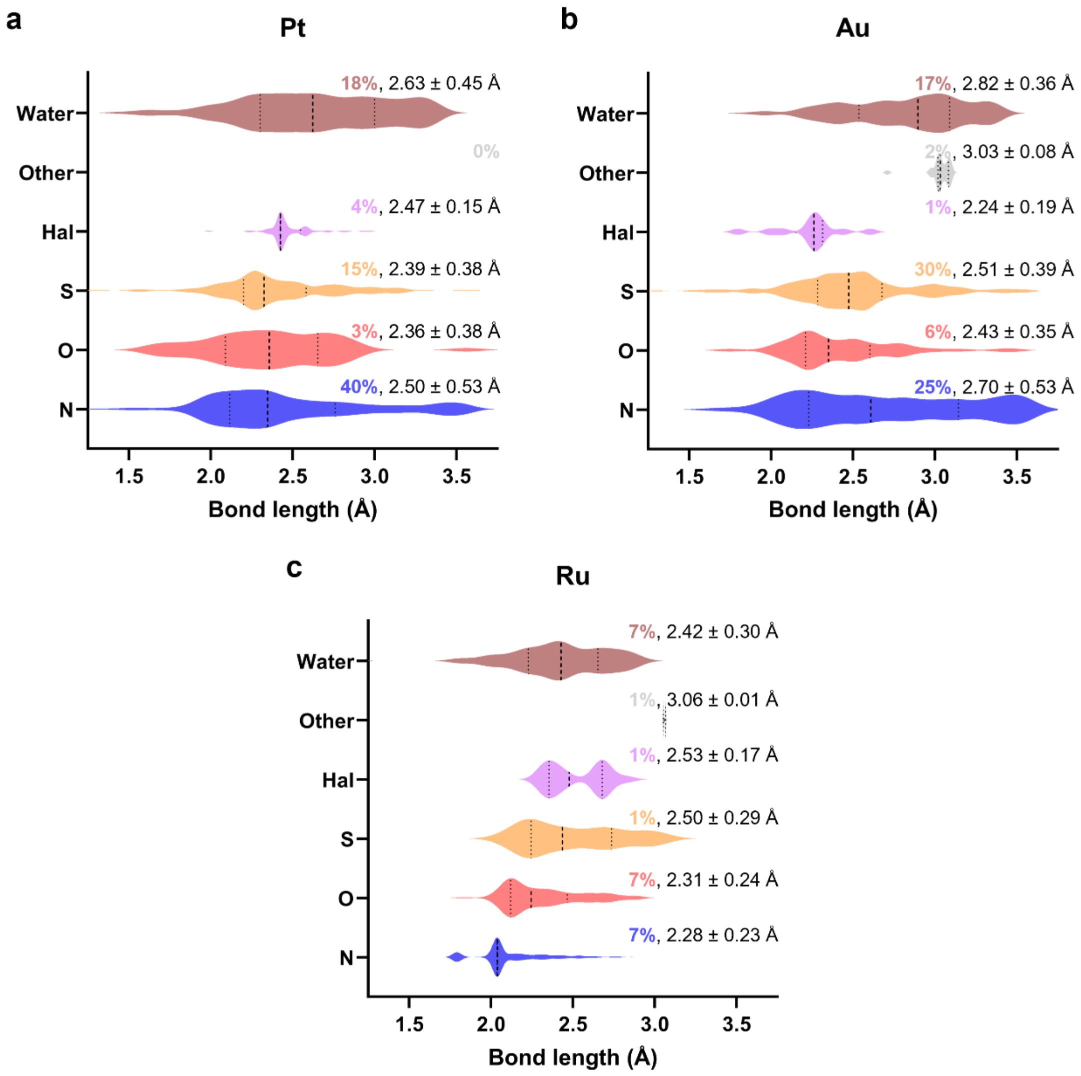

Supplementary Figure 16. Metal-biomolecule frequency of binding atom and bond lengths (N, O, S, water, halides and other) for Pt, Au and Ru. Extracted from the MESPEUS database.[42] Percentages do not add up to 100% as atoms bound to metals that are not part of the biomolecule, water molecules or halides are not taken into consideration.

Supplementary Table 10. Results for the validation of the docking protocol.

Metal	PDB	Lowest energy		Biggest cluster	
		Pose energy (kcal/mol)	RMSD	Pose energy (kcal/mol)	RMSD
Pt	3CO3	−8.71	0.44 (0.20)	−8.71 (−8.58)	0.44 (0.20)
	4QGZ:1	−6.65	2.51 (2.20)	−6.65 (−6.62)	2.51 (2.20)

	4QGZ:2	−4.70	2.63 (2.07)	−4.70 (−4.63)	2.63 (2.07)
	4ZEE:1	−6.37	5.88 (6.38)	−6.37 (−6.30)	5.88 (6.38)
	4ZEE:2	−3.59	6.56 (5.53)	−3.32 (−3.30)	3.38 (2.13)
**Au**	5JVX:1	−7.46	3.34 (1.31)	−7.46 (−7.32)	3.34 (1.31)
	5JVX:2	−5.07	3.20 (5.02)	−4.74 (−4.51)	0.42 (0.22)
	5JVX:3	−6.13	7.88 (7.51)	−6.13 (−6.05)	7.88 (7.51)
	5JVX:4	−5.36	7.02 (6.03)	−5.36 (−5.00)	8.49 (6.03)
	7R1P*	−7.13	n.a. (0.62)	−5.87 (−5.67)	n.a. (0.10)
**Ru**	1T3P	−7.75	1.61 (0.51)	−7.75 (−7.67)	1.61 (0.51)
	6BO1	−7.49	5.76 (1.69)	−7.49 (−7.47)	5.76 (1.69)
	6BO2	−9.57	0.39 (0.22)	−9.57 (−9.56)	0.39 (0.22)
	6WGO	−7.13	9.88 (3.59)	−5.87 (−5.67)	8.62 (1.08)
	8C39	−7.63	1.81 (0.57)	−7.18 (−7.48)	8.32 (0.97)

*The ligands were not modelled in this PDB structure.

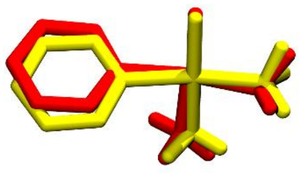

Pt:3CO3

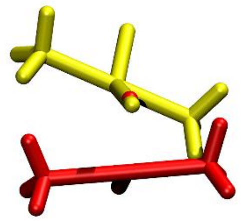

Pt:4QGZ:1

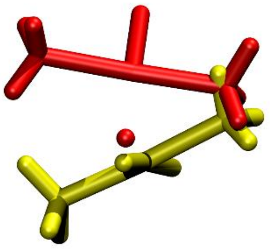

Pt:4QGZ:2

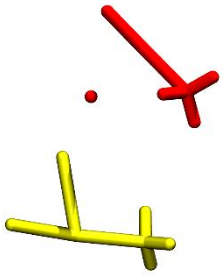

Pt:4ZEE:1

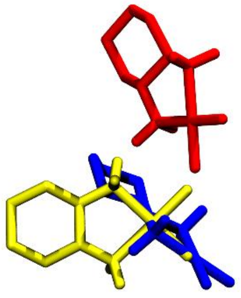

Pt:4ZEE:2

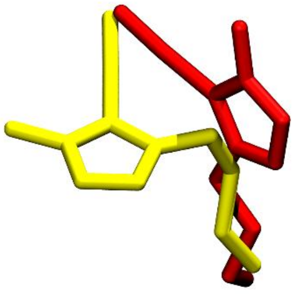

Au:5JVX:1

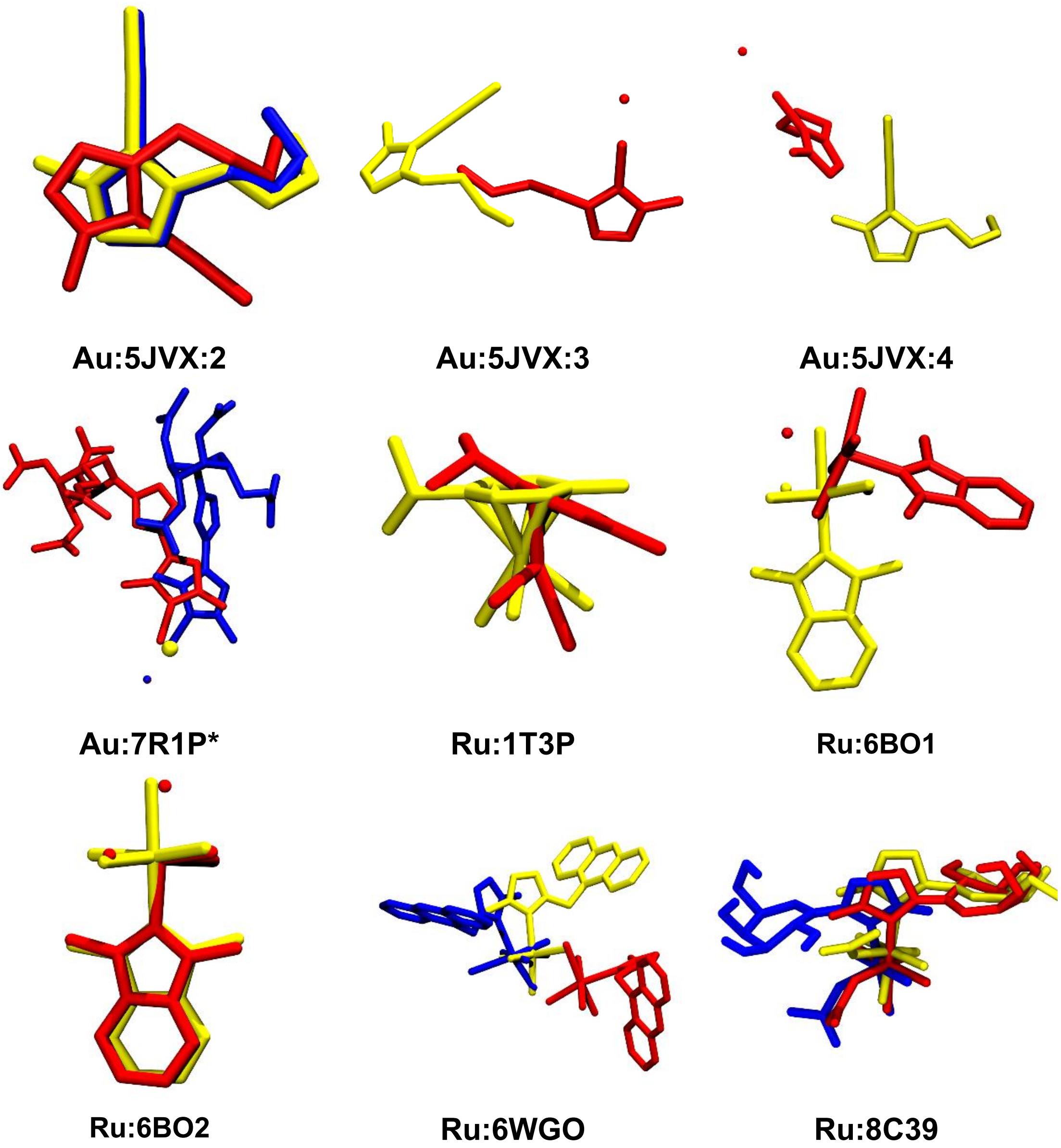

Supplementary Figure 17. Redocking poses for the validation of the docking protocol. Reference (yellow), lowest energy (red), and bigger cluster (blue, if different than the lowest energy pose). *The ligands were not modelled in this PDB structure.

Molecular docking was performed using AutoGrid 4.2 and AutoDock 4.2. Solvent molecules, cocrystallised ligands, and cofactors were removed from the biomolecule structure. If needed, hydrogen atoms were added to the biomolecule, and non-polar hydrogen atoms were merged. Afterwards, Gasteiger charges were calculated. A grid box centred around the crystallographic binding site (54 × 54 × 54 points, 20 Å) with a resolution of 0.375 Å (which is roughly a quarter of the length of a carbon-carbon single bond) was created. The ligand structure was prepared by replacing the labile ligand(s) for a dummy-atom (LP)

with null charge set at a distance of 2.1 Å from the metal atom. The free torsion tree was assigned, and the metal atom type was added with null charge. Optimised AutoDock force fields and pairwise interactions (see below) were used to calculate the energies and the best conformation was optimised by applying a Lamarckian Genetic Algorithm (LGA). With an initial population size of 350 and several evaluations of 50000000, the GA was allowed to run up to 27000 generations to find one best individual. Each experiment was set to 50 GA runs. The gene mutation and crossover rate were fixed at 2% and 80%, respectively. Clustering was performed to analyse the convergence of the simulations. The docking files for the docking of the studied complexes were prepared using AutoDock Tools using the protocol presented in the main text and the details above. The blind docking was performed for **Pt1^{Y}**, **Pt2^{Y}**, **Au3^{Y}** and **Ru4^{Y}** (Y = NR, O, S, Se) with a previously reported calculated trans-bound DNA interstrand adduct, 2J3N and 3AI8, respectively. A grid box encompassing the whole biomolecule (blind docking) with a resolution of 0.375 Å was created. The ligand structure was prepared by replacing the labile ligand(s) for a dummy-atom (LP) with null charge set at a distance of 2.1 Å from the metal atom. The free torsion tree was assigned, and the metal atom type was added with null charge. The optimised AutoDock force fields and pairwise interactions were used. The docking was performed as described above. The resulting poses were visualised with Visual Molecular Dynamics (VMD, University of Illinois) and Discovery Studio Visualizer (BIOVIA, Dassault Systèmes).

Supplementary Table 11. Summary of the docking results of **Pt1^{Y}** and **Pt2^{Y}** (Y = NR, O, S) to a simulated *trans*-bound interstrand DNA structure.[43]

Complex	**Lowest binding energy** (kcal/mol)	**Average binding energy** (kcal/mol)	Poses in cluster
**Pt1^{NMe}**	−13.77	−13.67	43
	−13.70	−13.66	7
**Pt1^{O}**	−13.71	−13.66	37
	−13.45	−13.38	13
**Pt1^{S}**	−14.03	−13.96	33
	−13.88	−13.83	17
**Pt2^{NBn}**	−18.16	−17.64	50

**Pt2^{O}**	−16.91	−16.55	50
**Pt2^{S}**	−17.21	−16.93	50

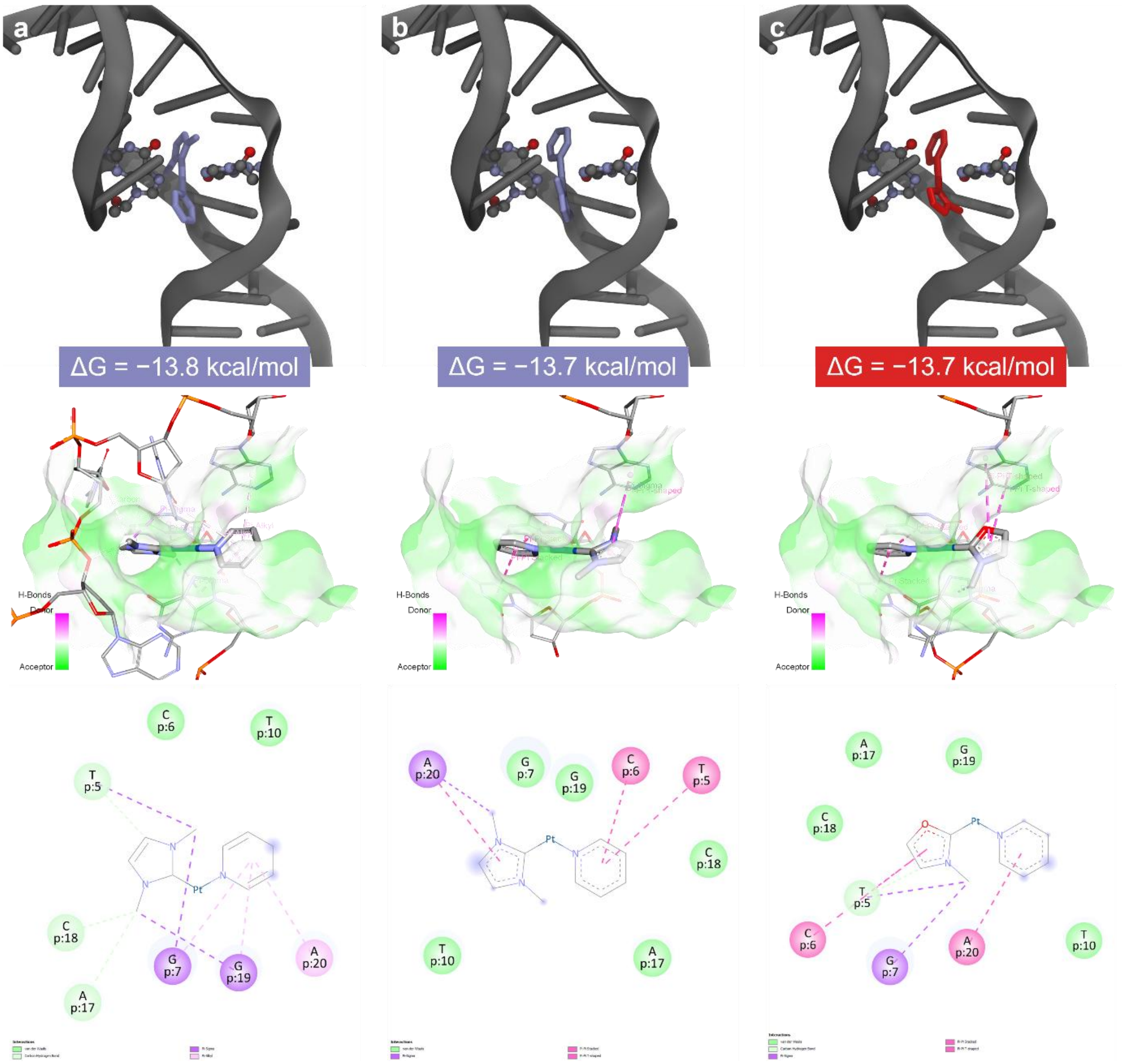

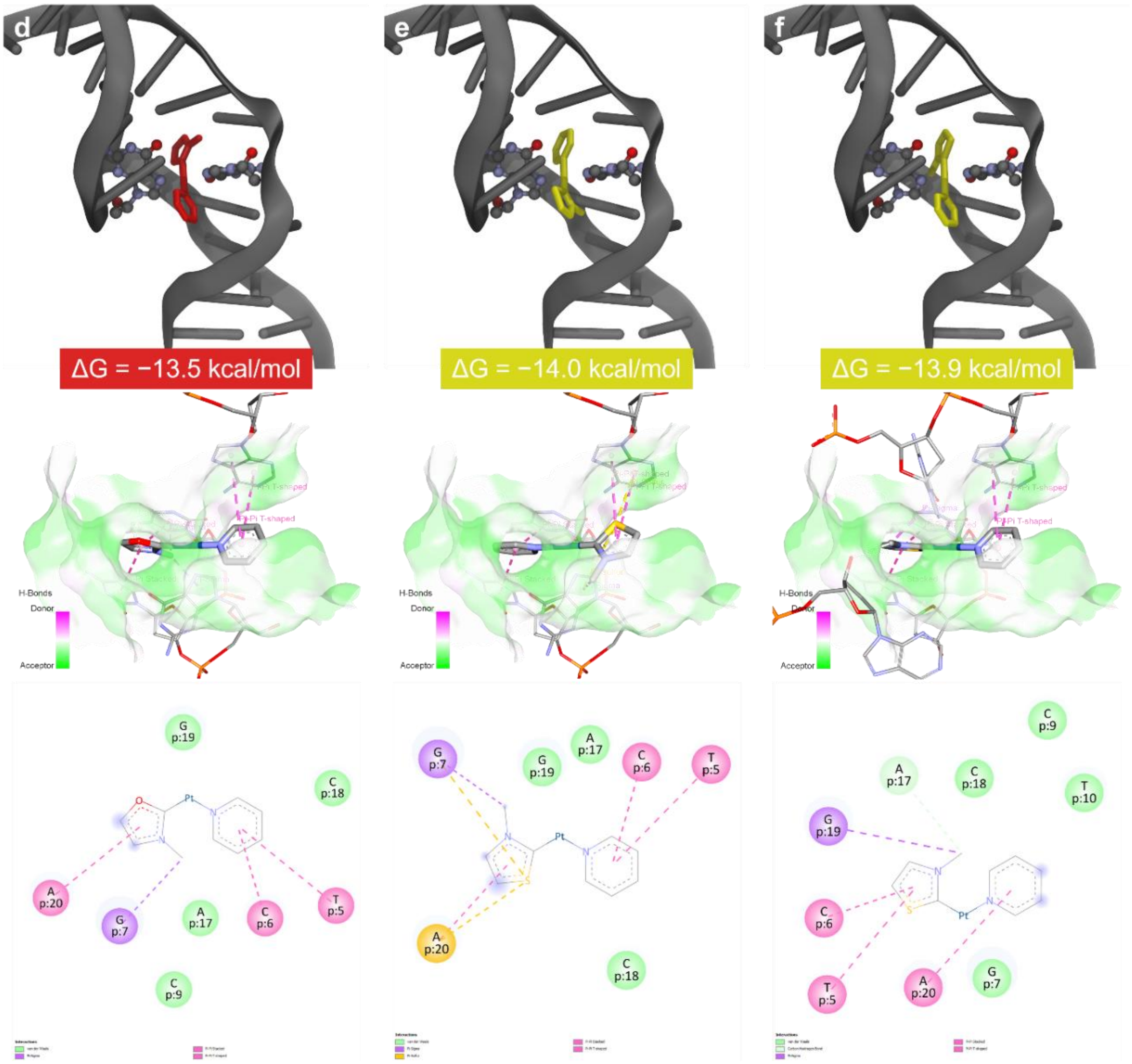

Supplementary Figure 18. Docking of **Pt1^{Y}** (Y = NMe (**a** and **b**), O (**c** and **d**), S (**e** and **f**)) to a simulated *trans*-bound interstrand DNA structure, showing the whole structure, the binding pocket with a H-bond coloured (donor and acceptor in pink and green, respectively) solvent accessible surface, and interaction map (pink, π-π stacking; pink, π-σ interaction; green, van de Waals; light green, carbon hydrogen bond; yellow, π-sulfur interaction). Two clusters with very close binding energies were obtained for each compound: **Pt1^{NMe}** (**a** and **b**), **Pt1^{O}** (**c** and **d**), and **Pt1^{S}** (**e** and **f**). Each conformation presents the pyridine and (N,Y)HC ligands in opposite positions. The structures are stabilised mainly by π-π and π-σ interactions, as well as π-sulfur in **Pt1^{S}**.

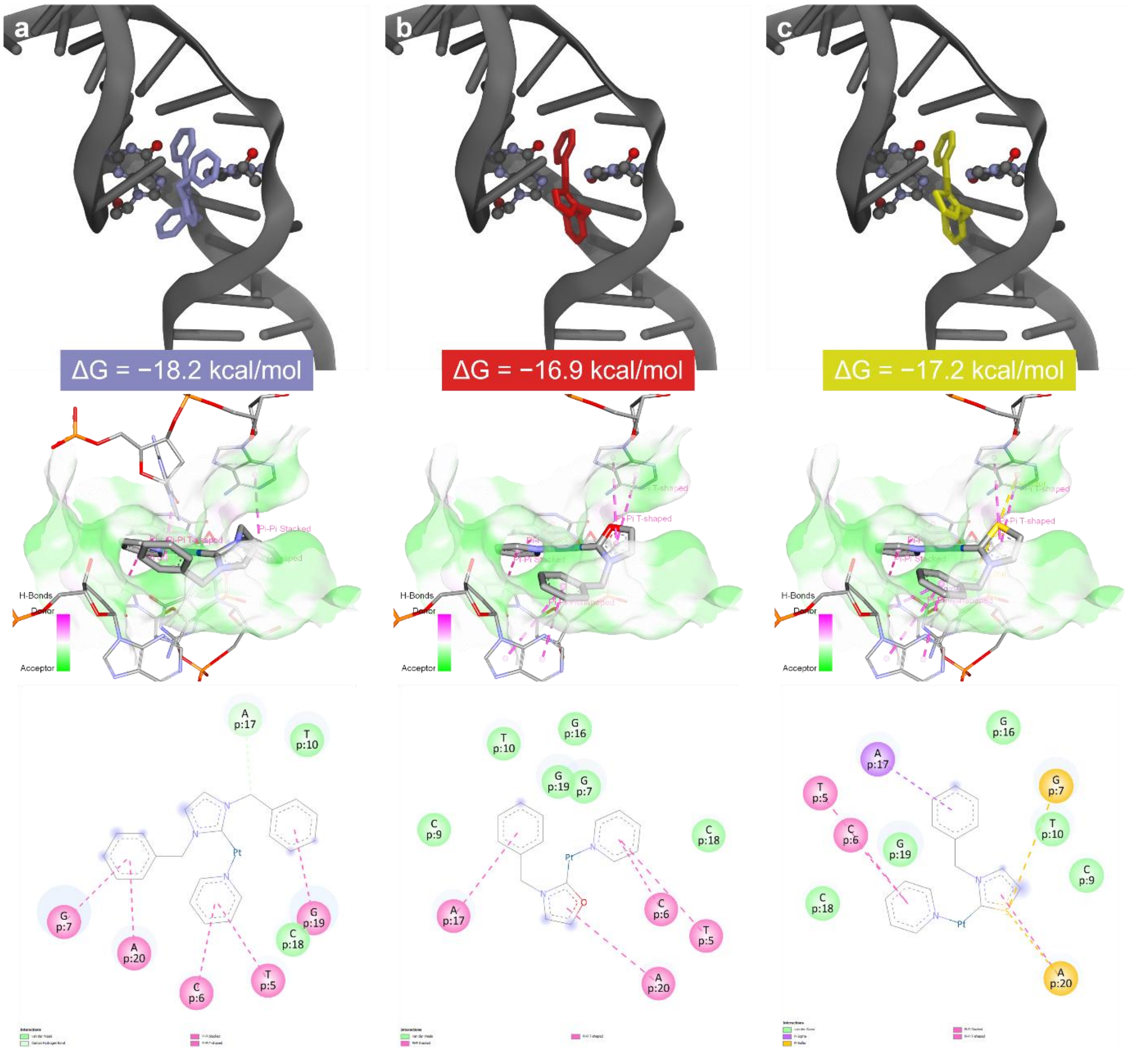

Supplementary Figure 19. Docking of **Pt2**^{Y} (Y = NBn (**a**), O (**b**), S (**c**), and Se (**d**)) to a simulated *trans*-bound interstrand DNA structure, showing the whole structure, the binding pocket with a H-bond coloured (donor and acceptor in pink and green, respectively) solvent accessible surface, and interaction map (pink, π-π stacking; pink, π-σ interaction; green, van de Waals; light green, carbon hydrogen bond; yellow, π-sulfur interaction). A single cluster is obtained for each structure, likely due to the higher number of π-π interactions occurring with the benzyl "wingtip". Both chalcogen atoms in **Pt2**^{O} and **Pt2**^{S} are positioned towards a hydrogen bond donor area of the solvent accessible surface which likely modulates the orientation of the (N,Y)HC ligand and the benzyl group. Due to the rigidity of the biomolecule in the docking, the nucleobase and the chalcogen atoms are too distant to be considered a classical hydrogen bond. However, in a dynamic system, it most likely would establish a HB interaction.

Supplementary Table 12. Summary of the docking results of **Au3^{Y}** to TrxR (PDB:2J3N).

Complex	Residue	Binding energy (kcal/mol)	Poses in cluster
**Au3^{NEt}**	Cys296	−9.03	17
	Cys189	−8.43	11
	Cys365	−7.91	6
	His439	−7.70	5
	Arg416	−7.30	3
**Au3^{O}**	Cys205	−9.32	18
	Cys475	−8.67	7
	Cys296	−8.50	6
	Cys365	−8.31	6
	Cys189	−7.65	3
**Au3^{S}**	Cys296	−8.79	10
	Cys365	−8.39	8
	Cys189	−8.16	3
	Cys296	−8.02	9
	His439	−7.85	4
**Au3^{Se}**	Cys296	−8.92	16
	Cys296	−8.80	1
	Cys365	−8.46	9
	Cys189	−8.23	4
	Cys296	−7.98	6

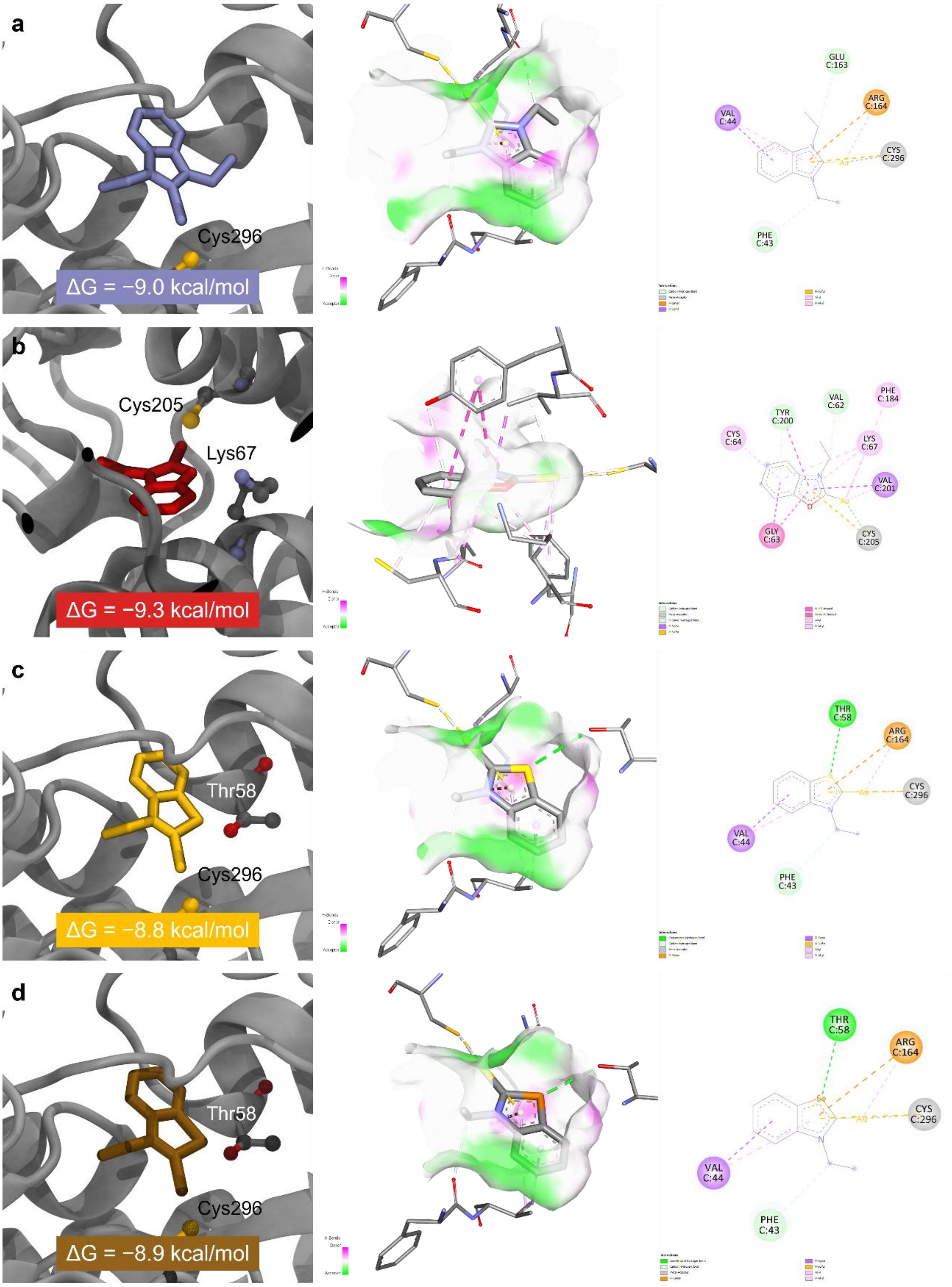

Supplementary Figure 20. Docking of **Au3^{NEt}** (**a**), **Au3^{O}** (**b**), and **Au3^{S}** (**c**) to TrxR, showing the interacting residues, the binding pocket with a H-bond coloured (donor and acceptor in pink and green, respectively) solvent accessible surface, and interaction map (pink, π-π stacking; purple, π-σ interaction; light pink, π-alkyl interaction; orange, π-cation interaction; bright green, hydrogen bond; light green, carbon hydrogen bond; yellow, π-sulfur interaction). **Au3^{O}** appears to form a HB (3.0 Å) with the amine in Lys67, whilst **Au3^{S}** and **Au3^{Se}** might form a ChB (3.6 Å) with the carbonyl of Thr58.

Supplementary Table 13. Summary of the docking results of and **Ru4^{Y}** to catB (PDB:3AI8).

Complex	Residue	**Binding energy** (kcal/mol)	Poses in cluster
**Ru4^{NBn}**	Cys29:His199	−10.47	4
	His111	−9.79	3
	His145	−9.01	4
	His111	−8.94	4
	Cys29	−8.59	4
**Ru4^{S}**	Cys29:His199	−10.30	8
	His145	−9.79	16
	His111	−9.32	3
	His111	−9.17	10
	Tyr94	−7.50	3
**Ru4^{Se}**	Cys29:His199	−10.36	17
	Cys29:His199	−9.69	7
	His111	−9.14	9
	Cys29	−8.61	4
	His145	−8.48	3

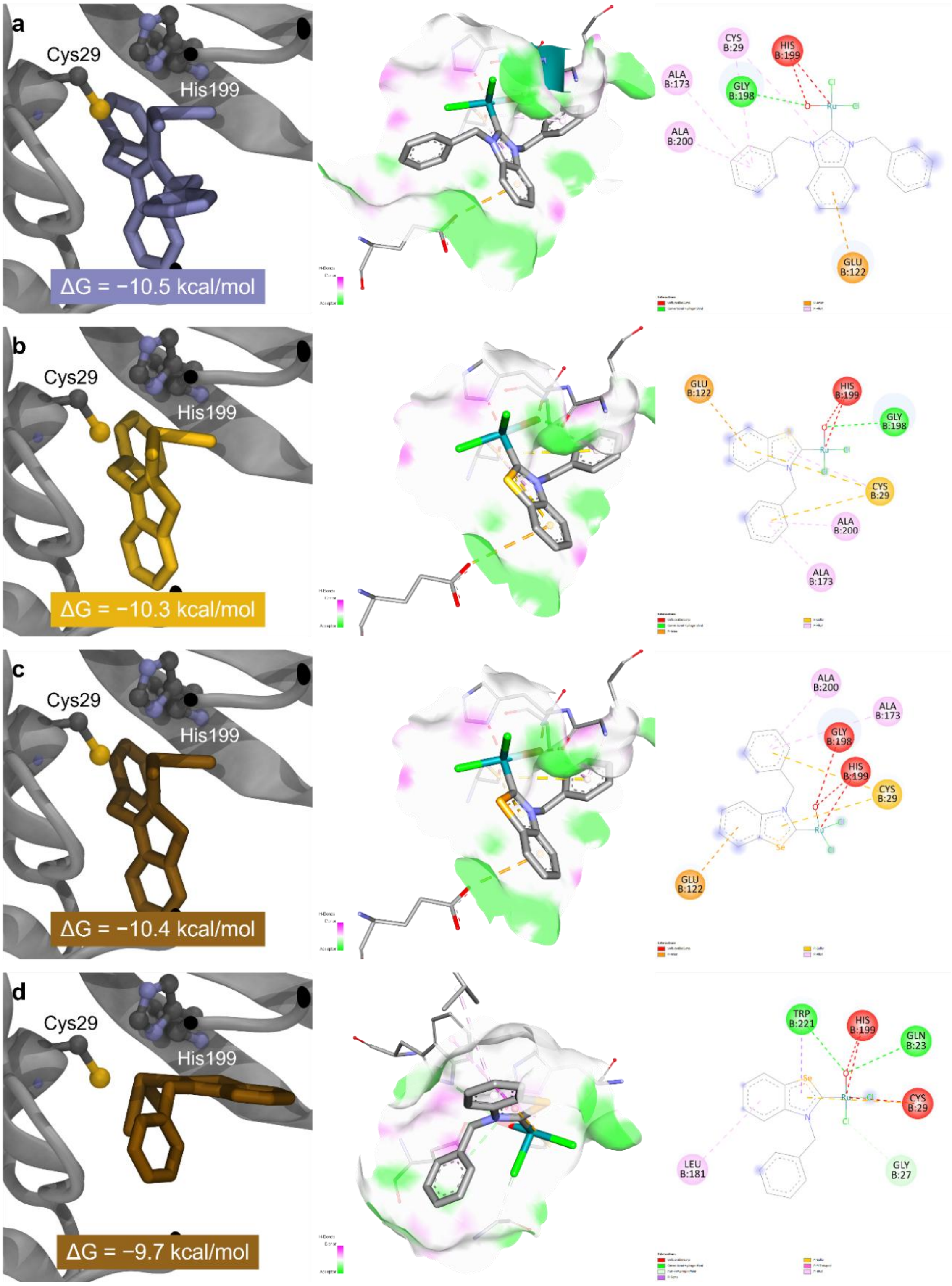

Supplementary Figure 21. Docking of **Ru4^{NBn}** (**a**), **Ru4^{S}** (**b**), and **Ru4^{Se}** (**c** and **d**) to CatB, showing the interacting residues, the binding pocket with a H-bond coloured (donor and acceptor in pink and green, respectively) solvent accessible surface and interaction map (light pink, π-alkyl interaction; orange, π-anion interaction; bright green, hydrogen bond; yellow, π-sulfur interaction; red, unfavourable bump). In **d**, the selenium atom is positioned towards a donor area of the surface in the proximity of a carbonyl, which could indicate the presence of a ChB interaction.

7. NMR spectra

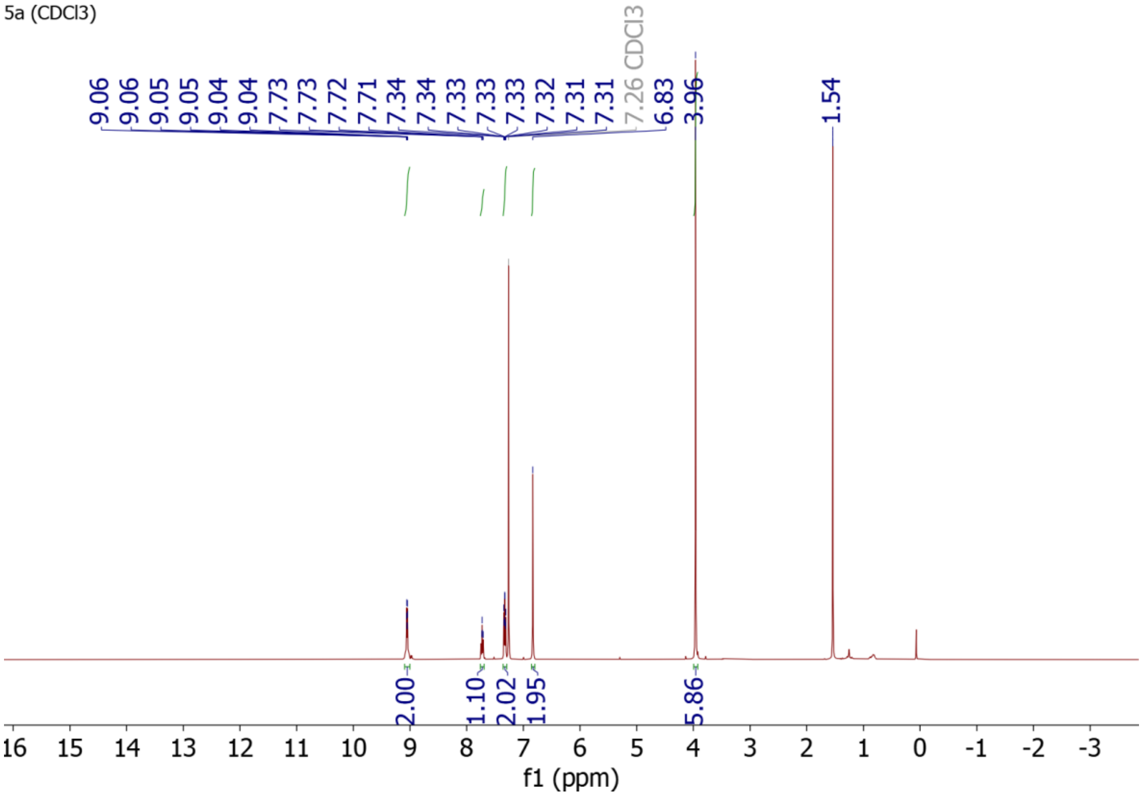

Supplementary Figure 22. ^{1}H NMR spectrum of **Pt1^{N}**.

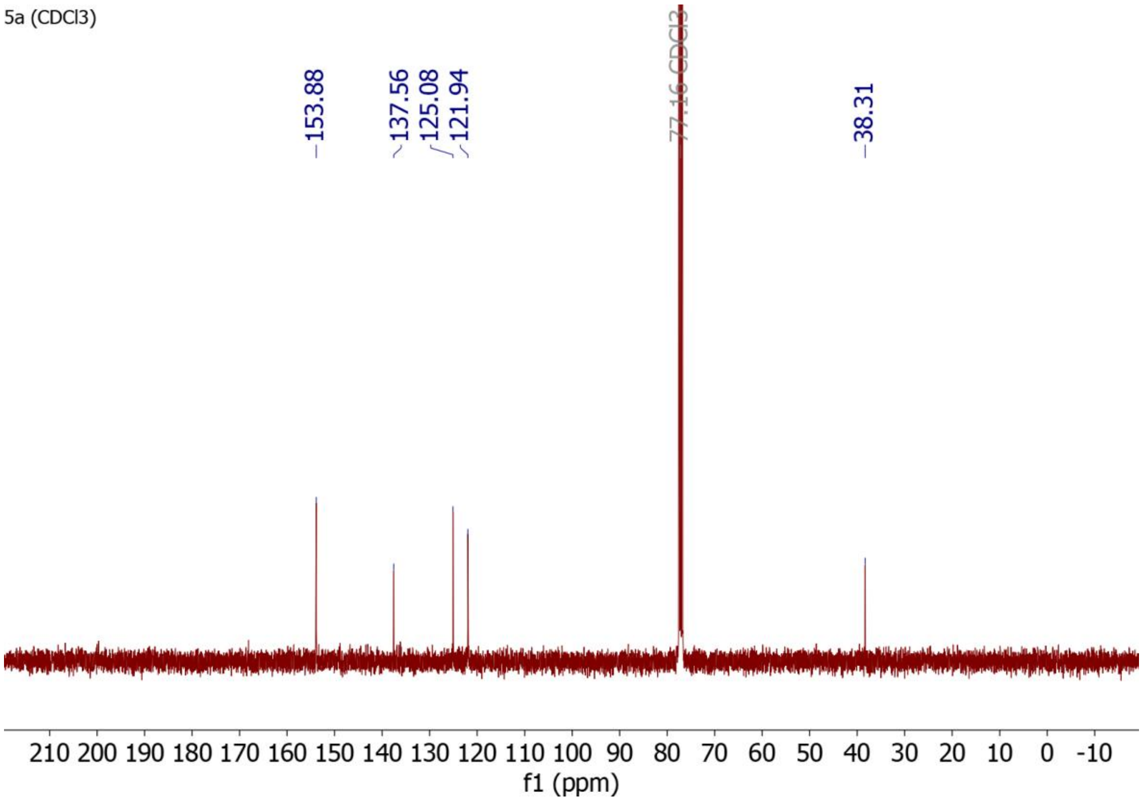

Supplementary Figure 23. ^{13}C NMR spectrum of **Pt1^{NMe}**.

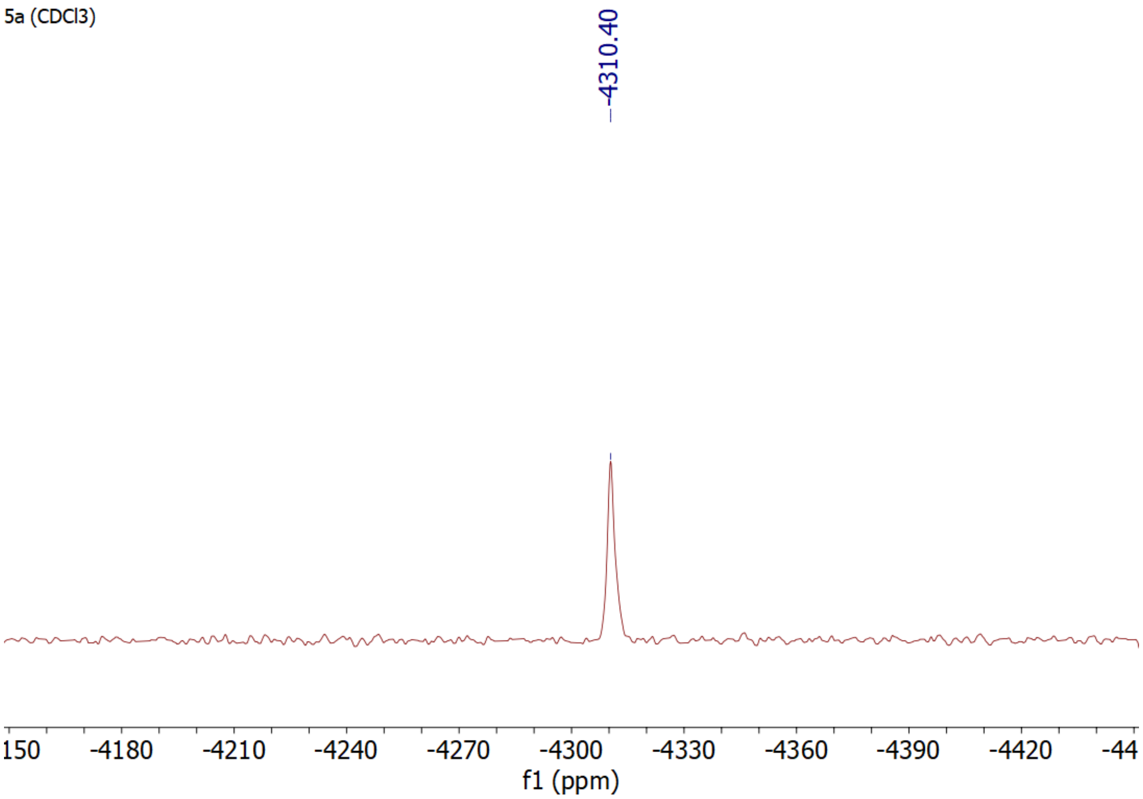

Supplementary Figure 24. ^{195}Pt NMR spectrum of **Pt1^{NMe}**.

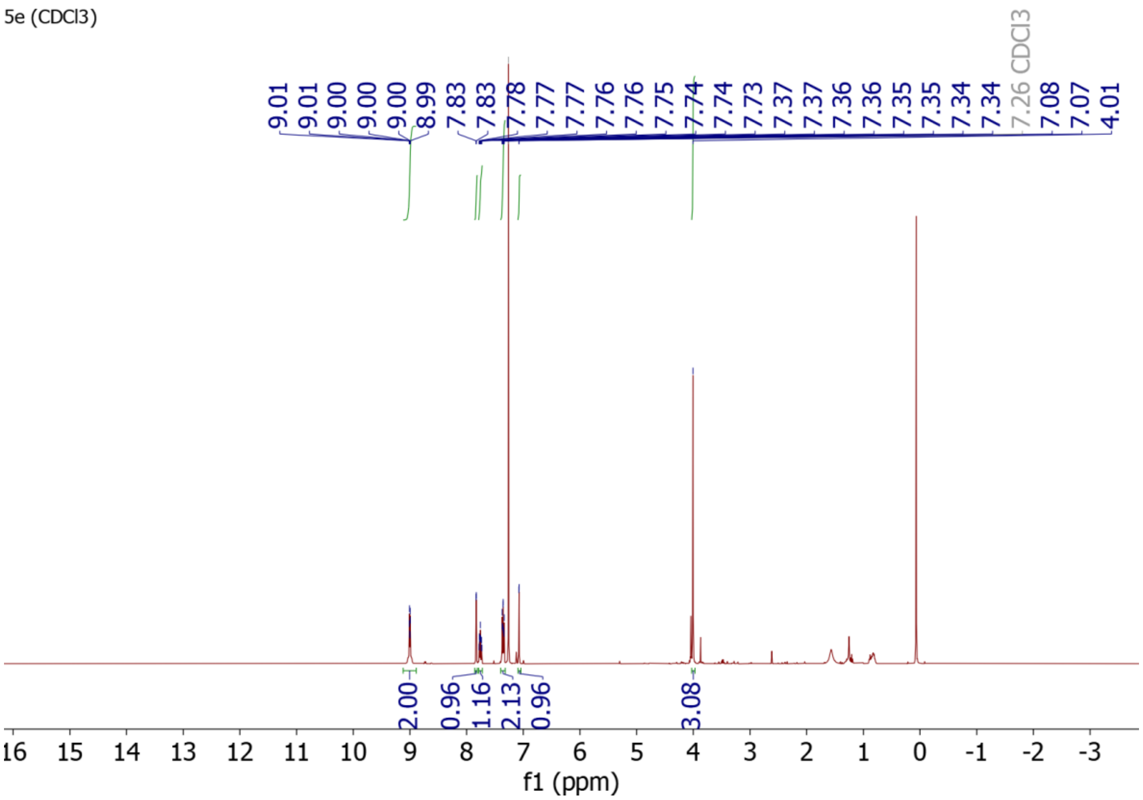

Supplementary Figure 25. ^{1}H NMR spectrum of **Pt1^{O}**.

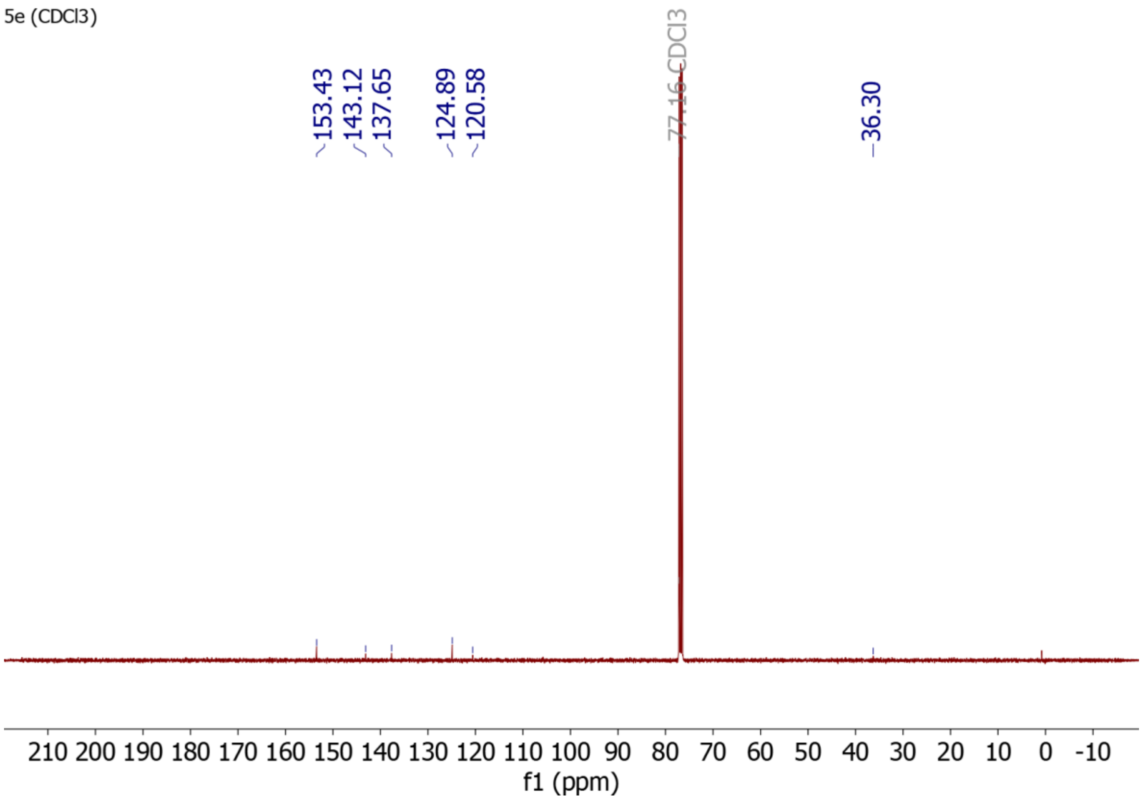

Supplementary Figure 26. ^{13}C NMR spectrum of **Pt1^{O}**.

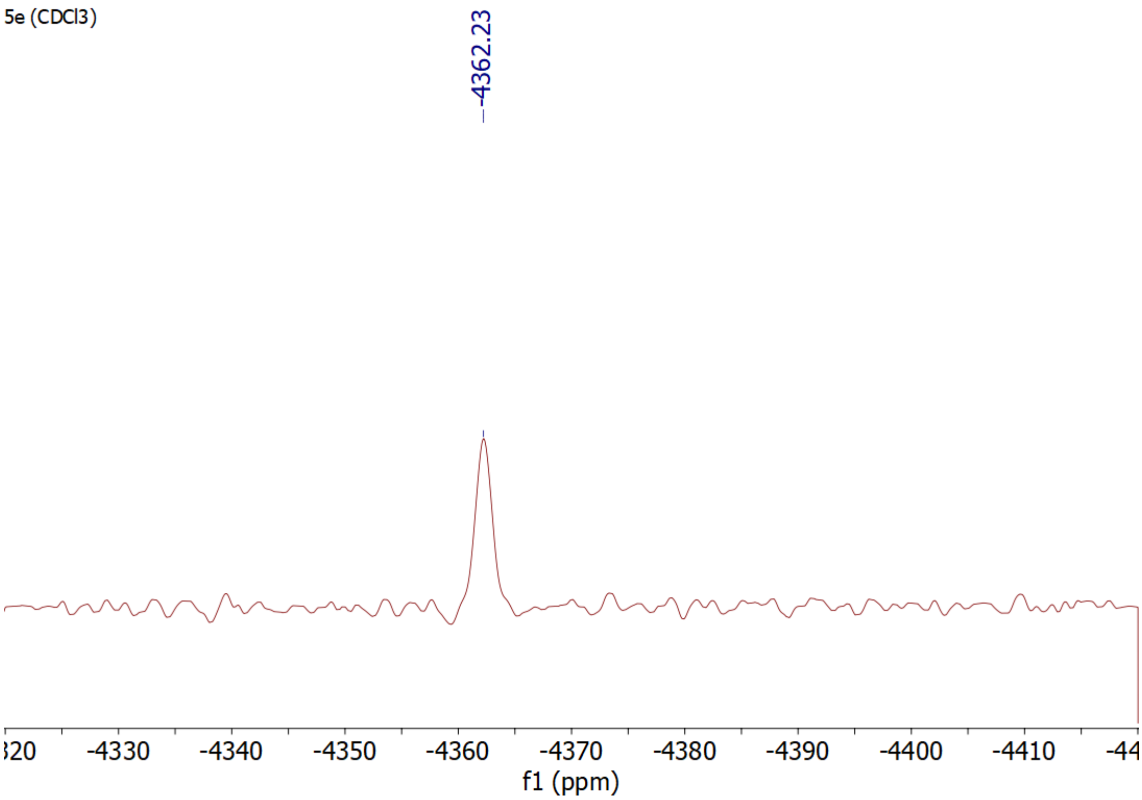

Supplementary Figure 27. ^{195}Pt NMR spectrum of **Pt1^{O}**.

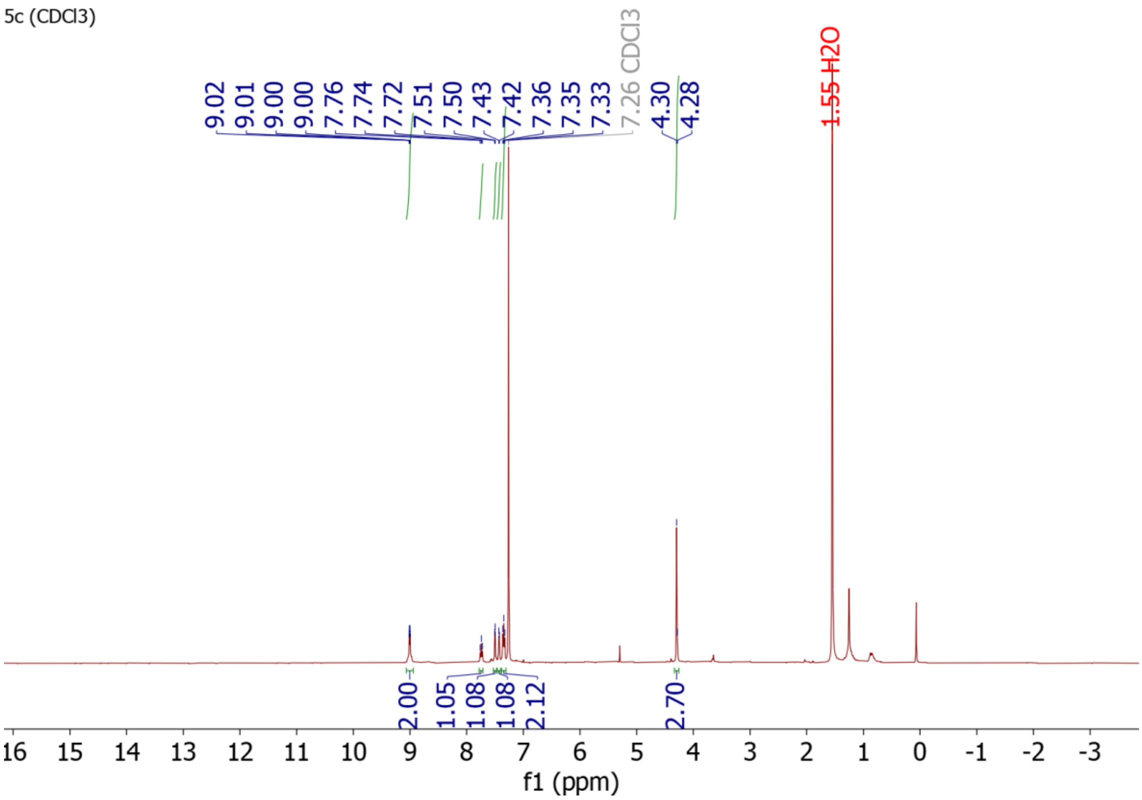

Supplementary Figure 28. ^{1}H NMR spectrum of **Pt1^{S}**.

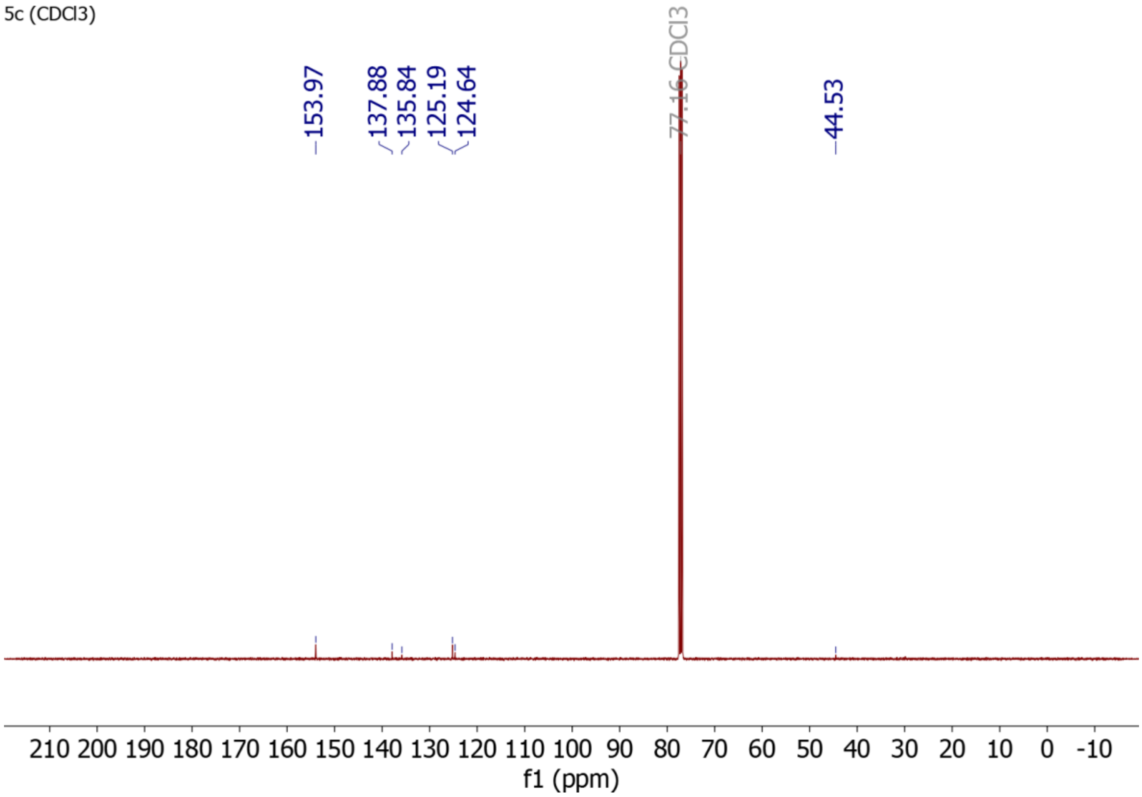

Supplementary Figure 29. ^{13}C NMR spectrum of **Pt1^{S}**.

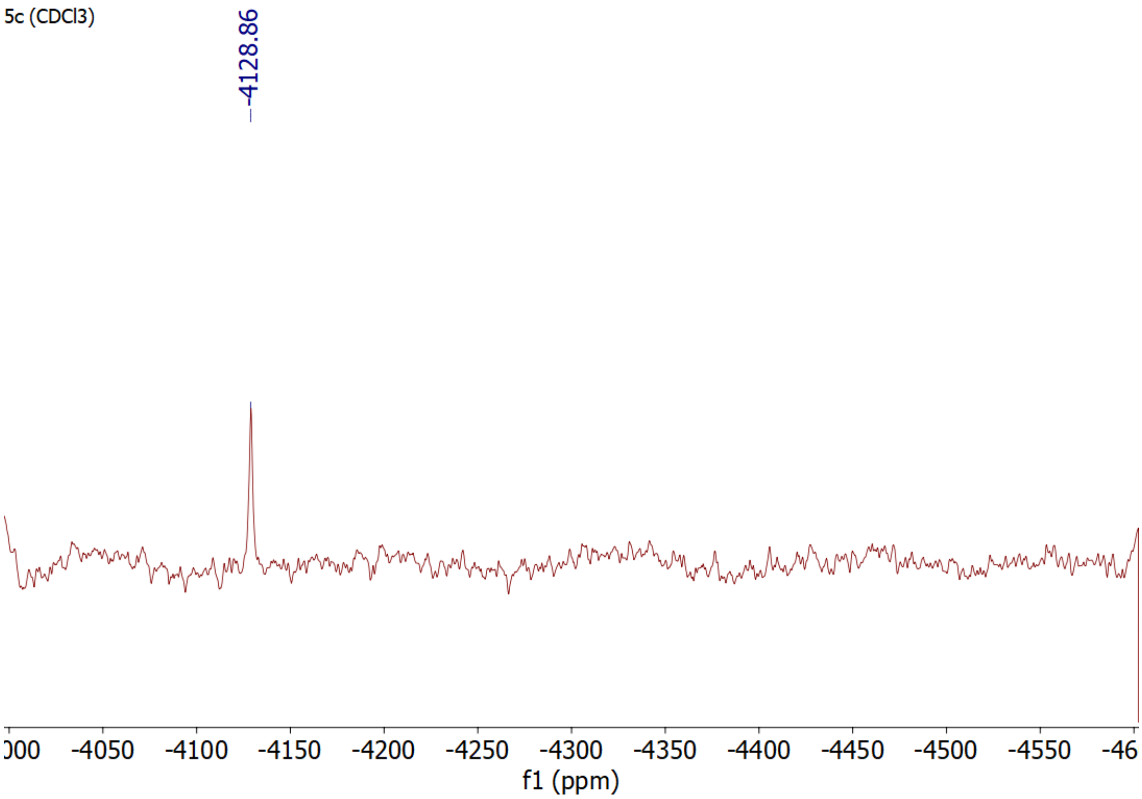

Supplementary Figure 30. ^{195}Pt NMR spectrum of **Pt1^{S}**.

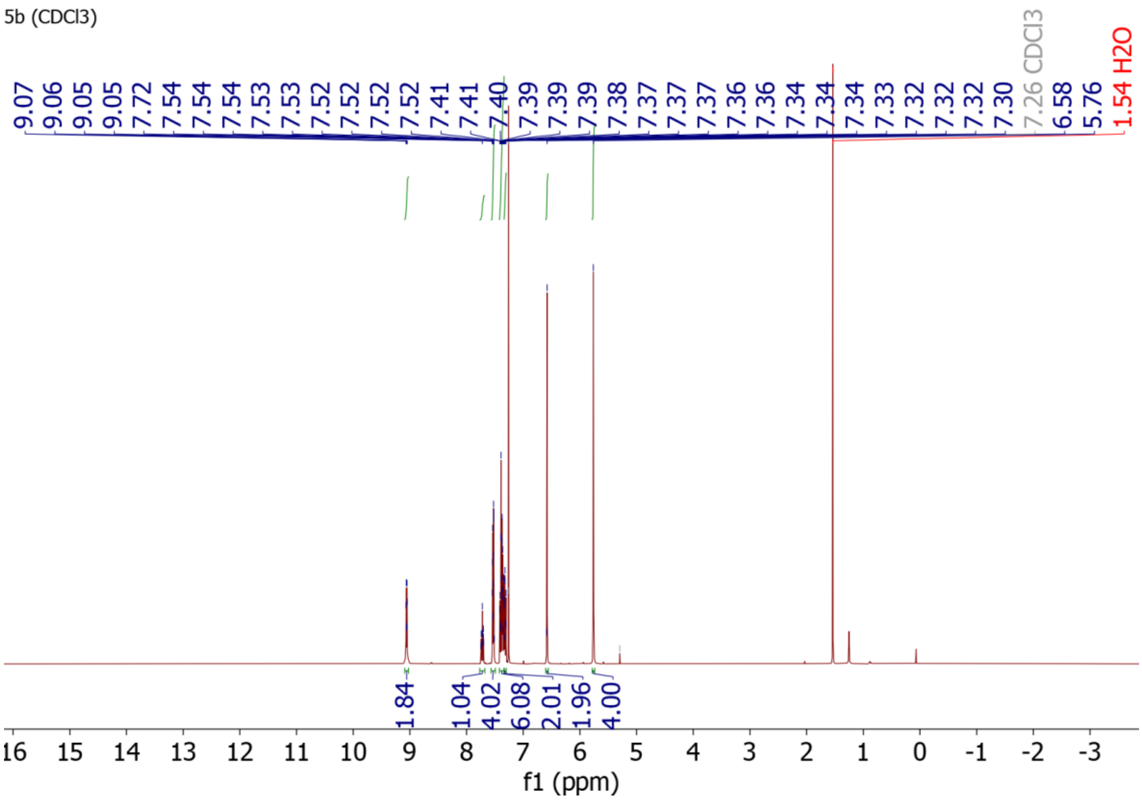

Supplementary Figure 31. ^{1}H NMR spectrum of **Pt2^{NBn}**.

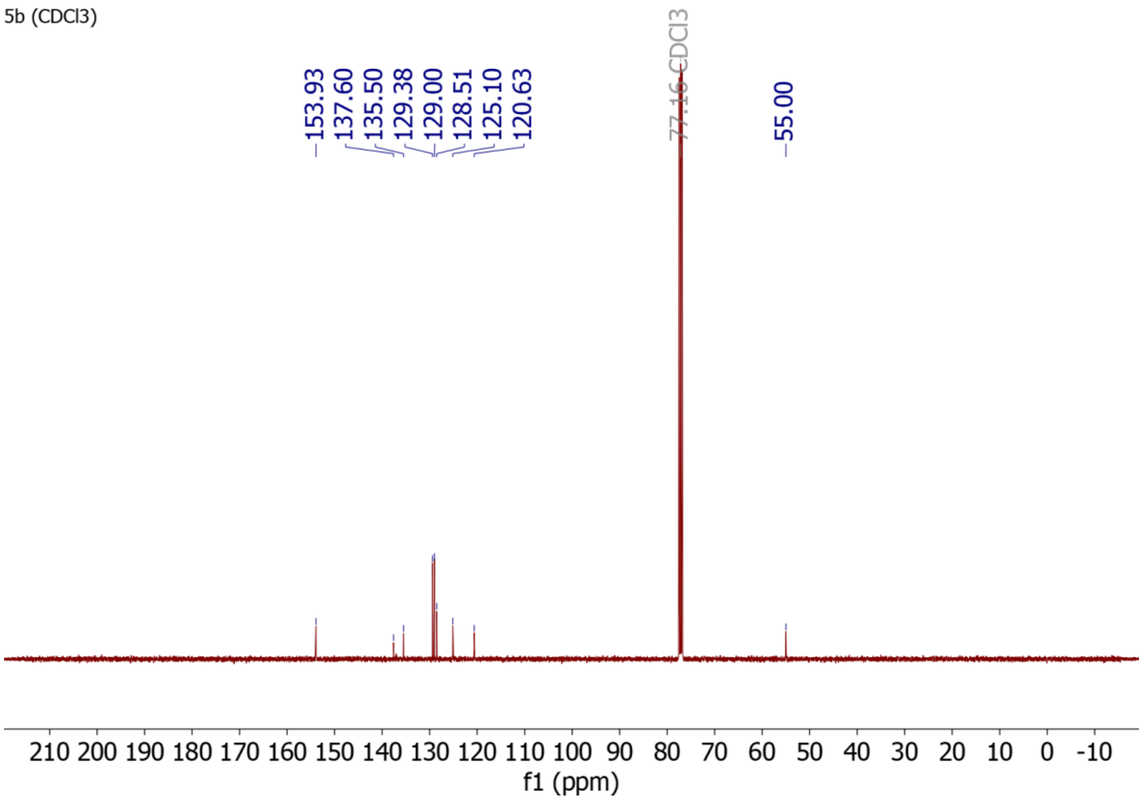

Supplementary Figure 32. ^{13}C NMR spectrum of **Pt2^{NBn}**.

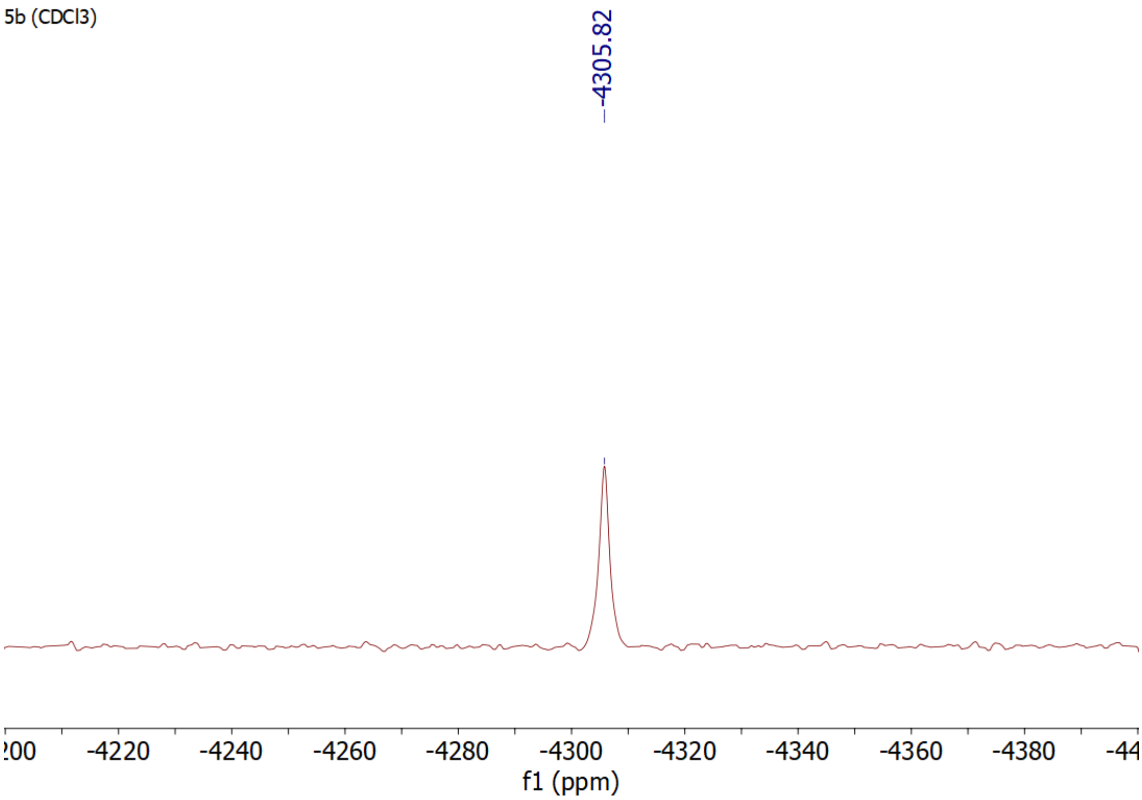

Supplementary Figure 33. ^{195}Pt NMR spectrum of **Pt2^{NBn}**.

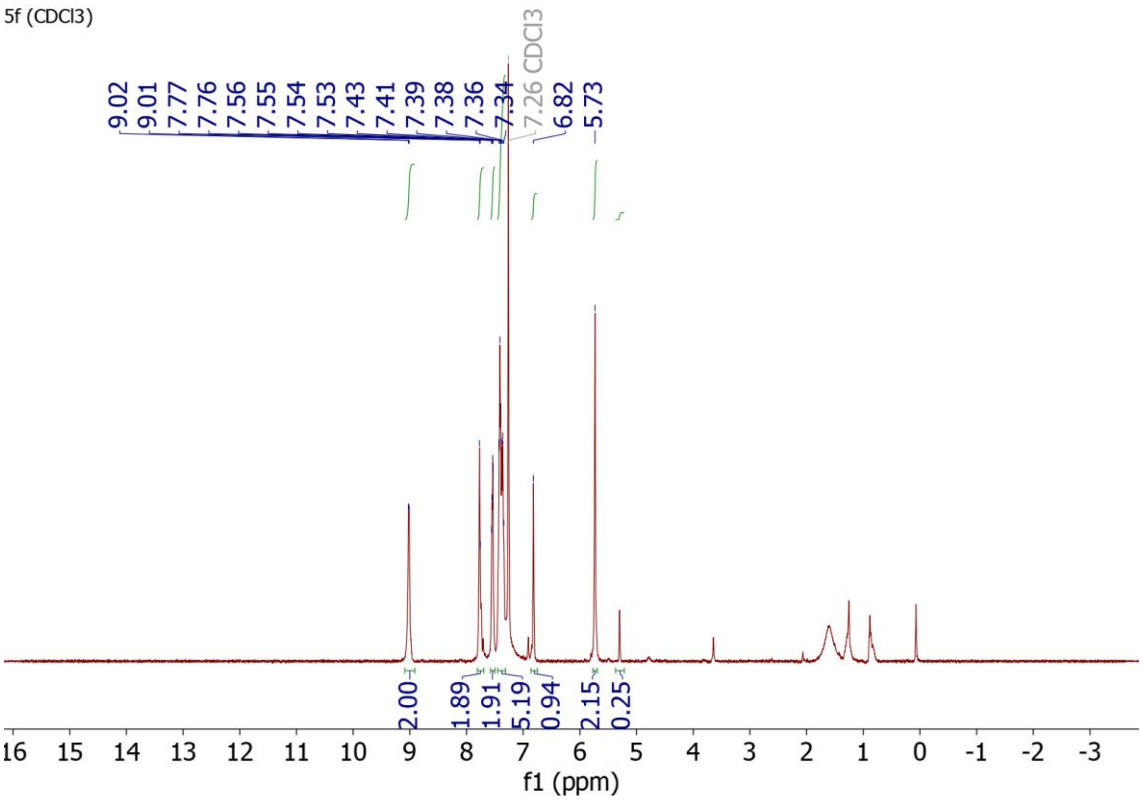

Supplementary Figure 34. ^{1}H NMR spectrum of **Pt2^{O}**.

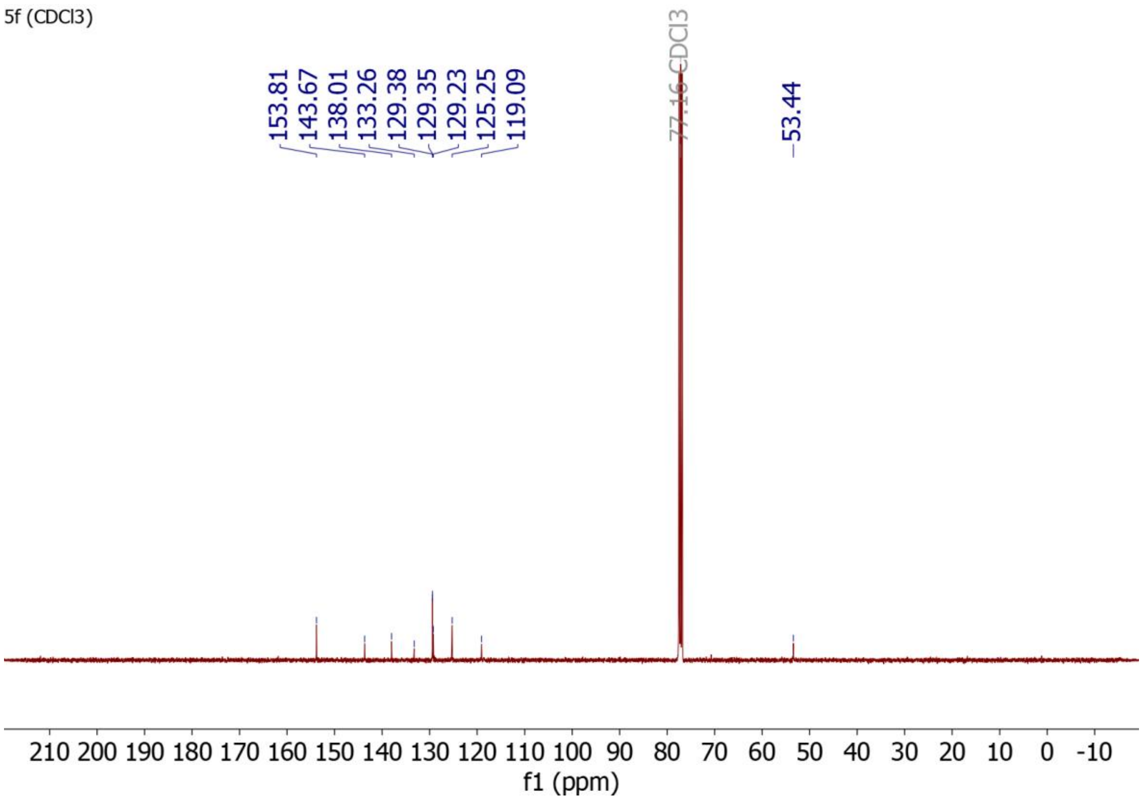

Supplementary Figure 35. ^{13}C NMR spectrum of **Pt2^{O}**.

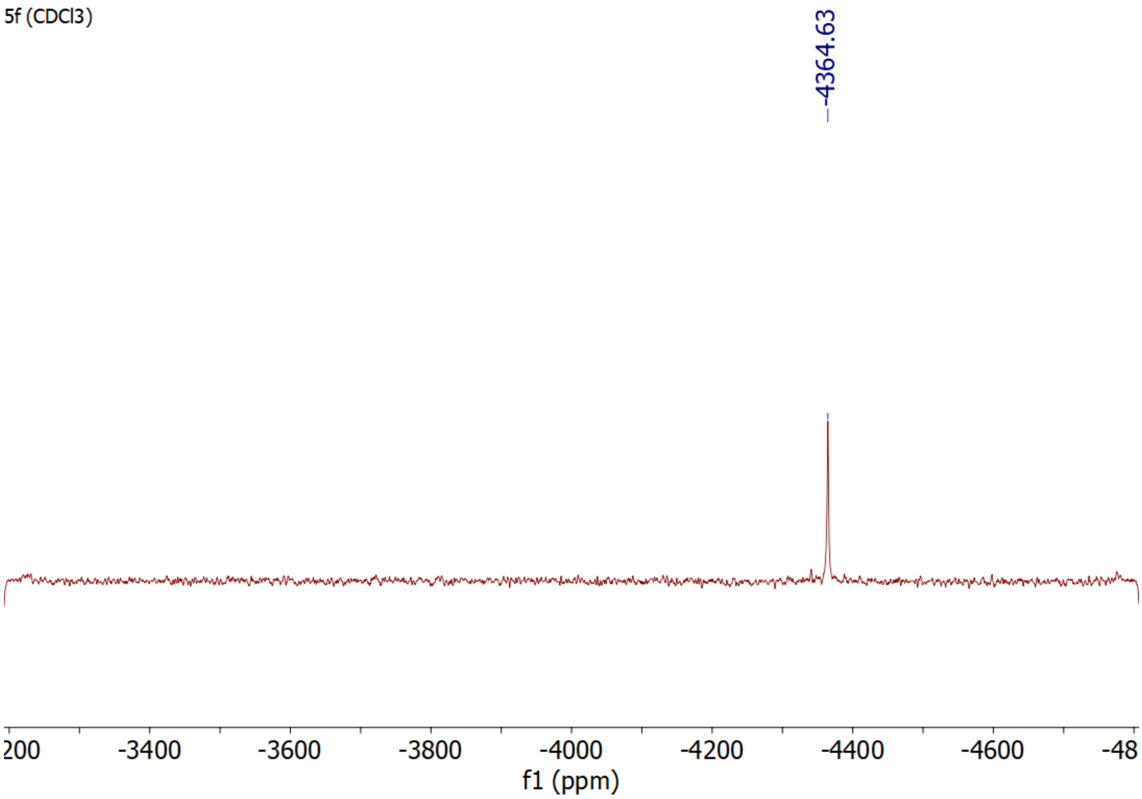

Supplementary Figure 36. ^{195}Pt NMR spectrum of **Pt2^{O}**.

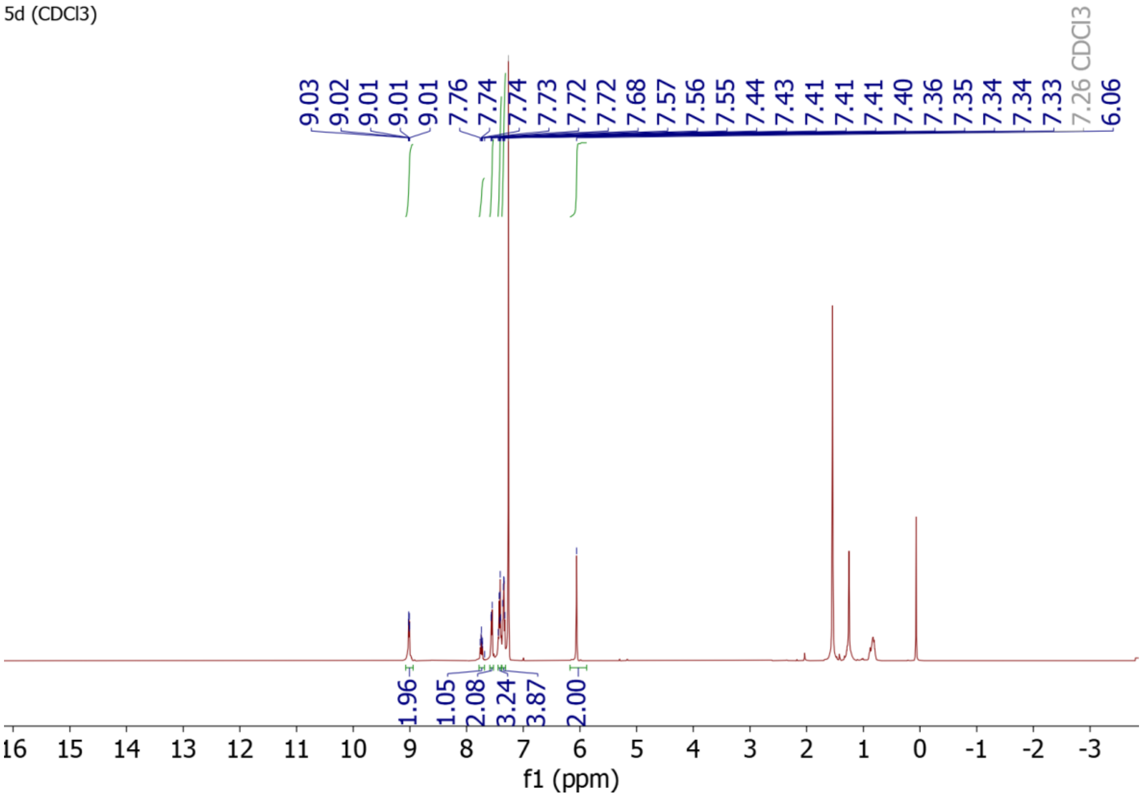

Supplementary Figure 37. ^{1}H NMR spectrum of **Pt2^{S}**.

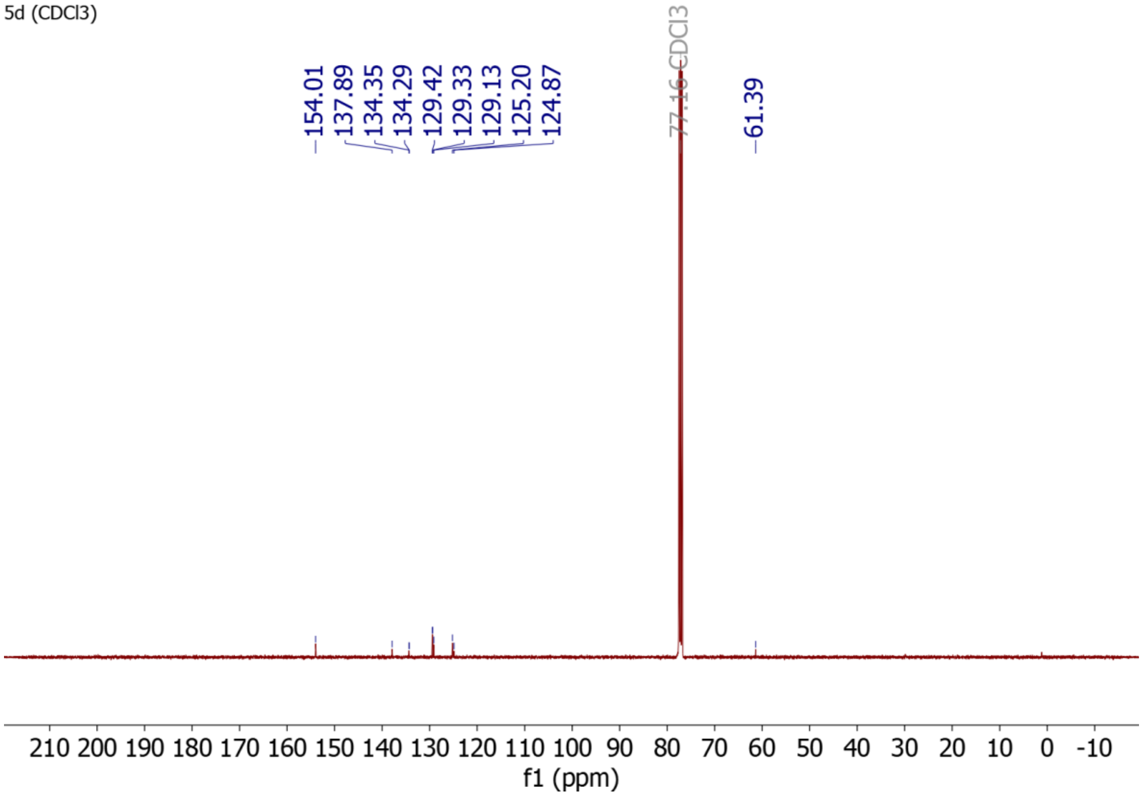

Supplementary Figure 38. ^{13}C NMR spectrum of **Pt2^{S}**.

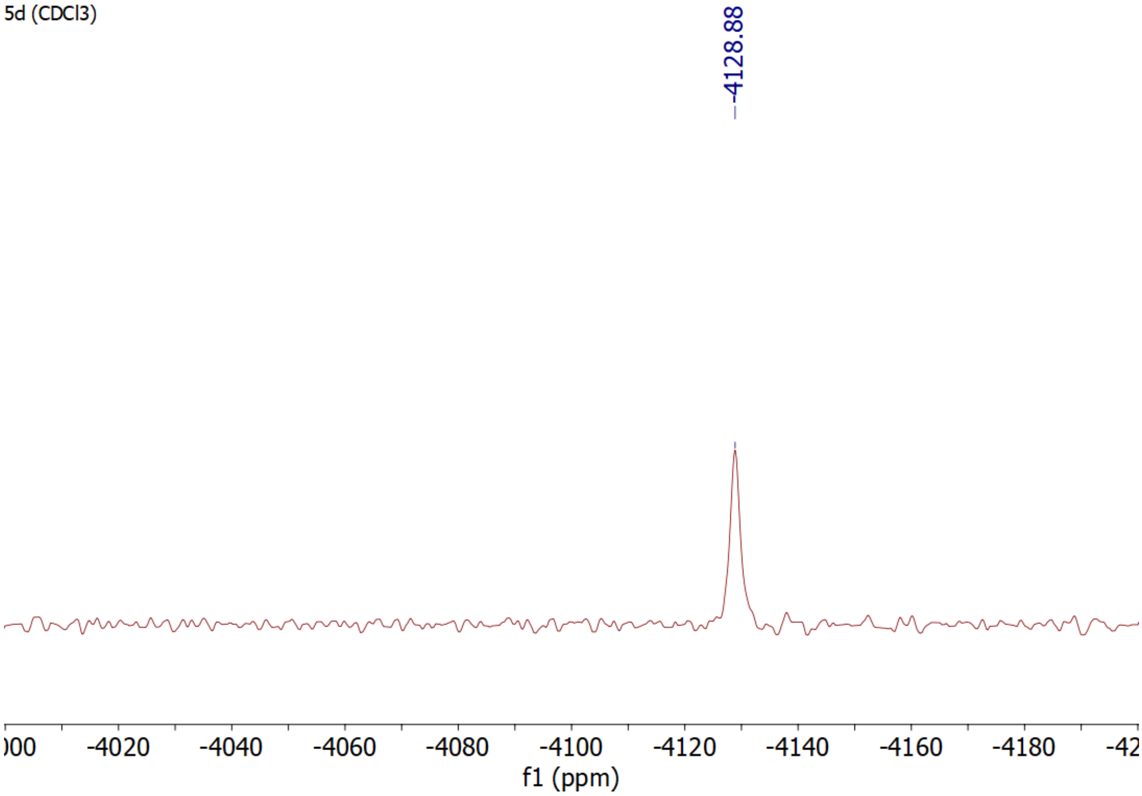

Supplementary Figure 39. ^{195}Pt NMR spectrum of **Pt2^{S}**.

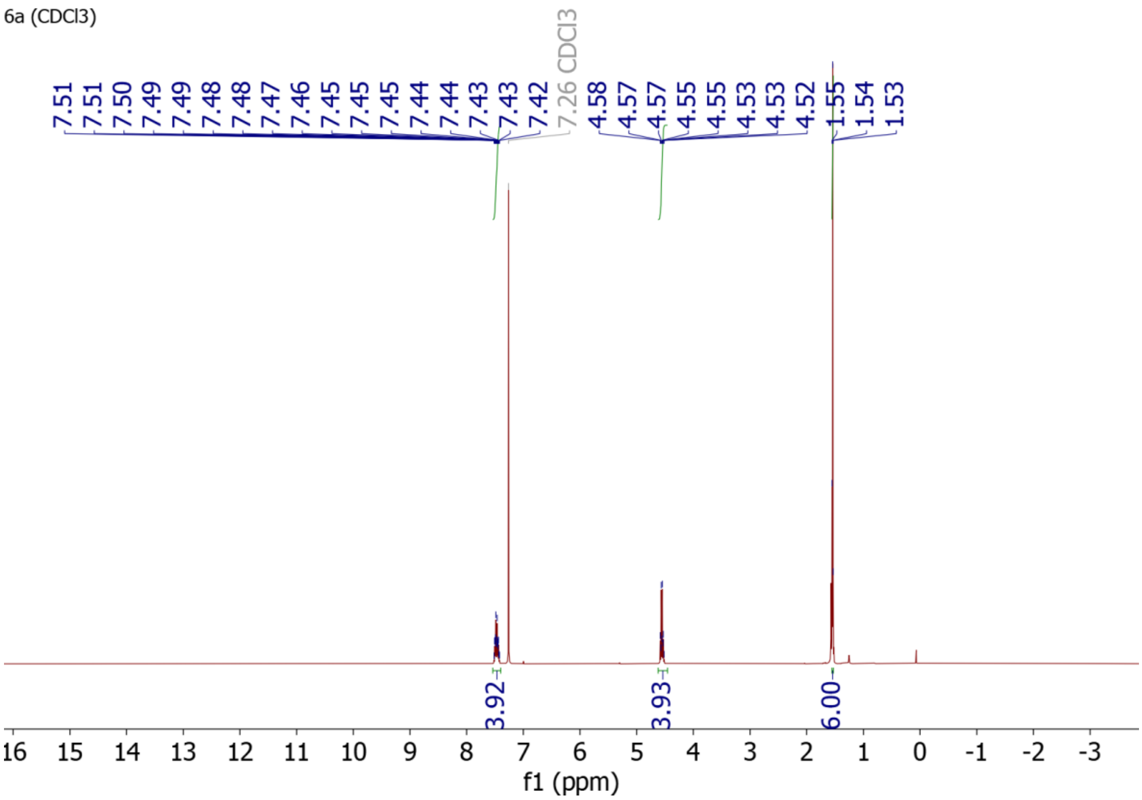

Supplementary Figure 40. ^{1}H NMR spectrum of **Au3^{NEt}**.

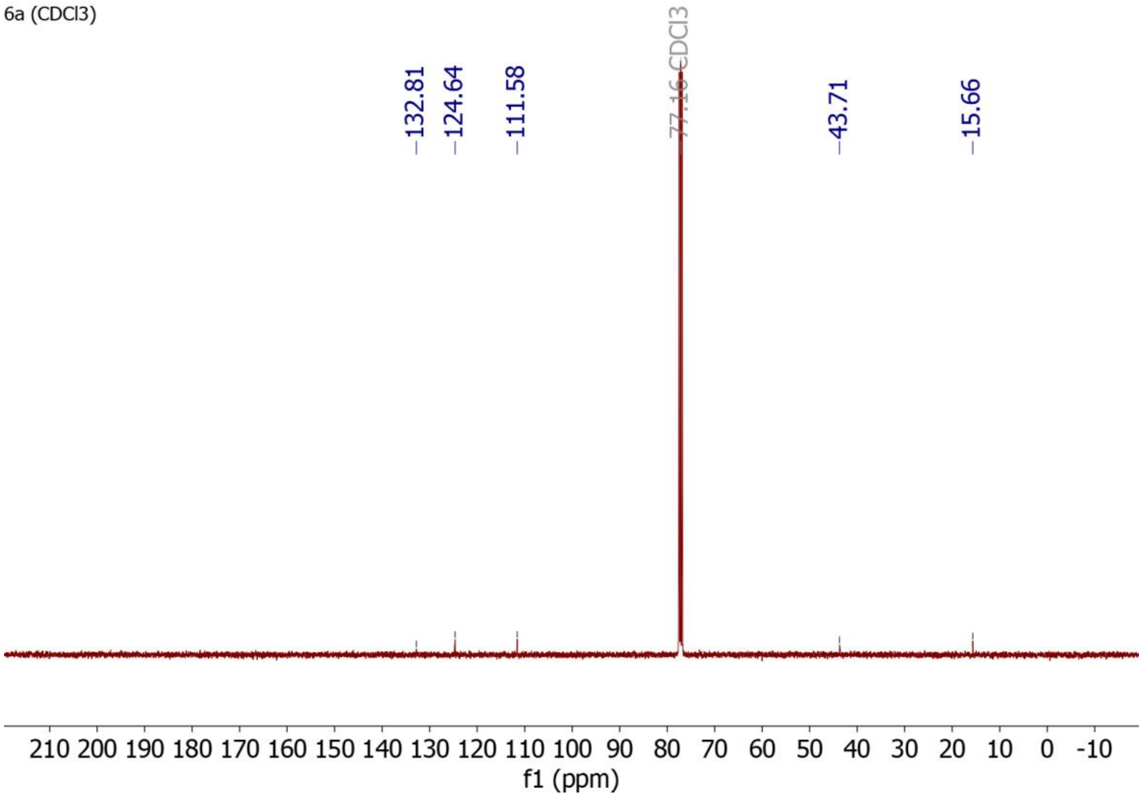

Supplementary Figure 41. ^{13}C NMR spectrum of **Au3^{NEt}**.

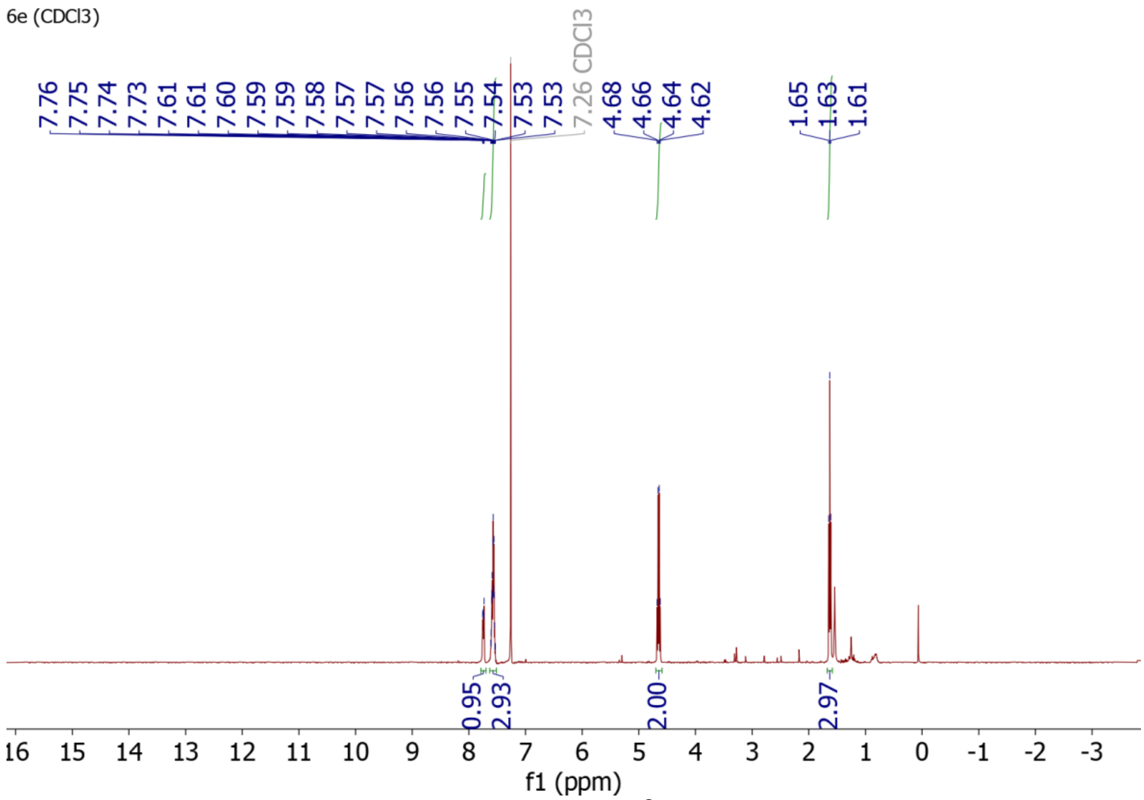

Supplementary Figure 42. ^{1}H NMR spectrum of **Au3^{O}**.

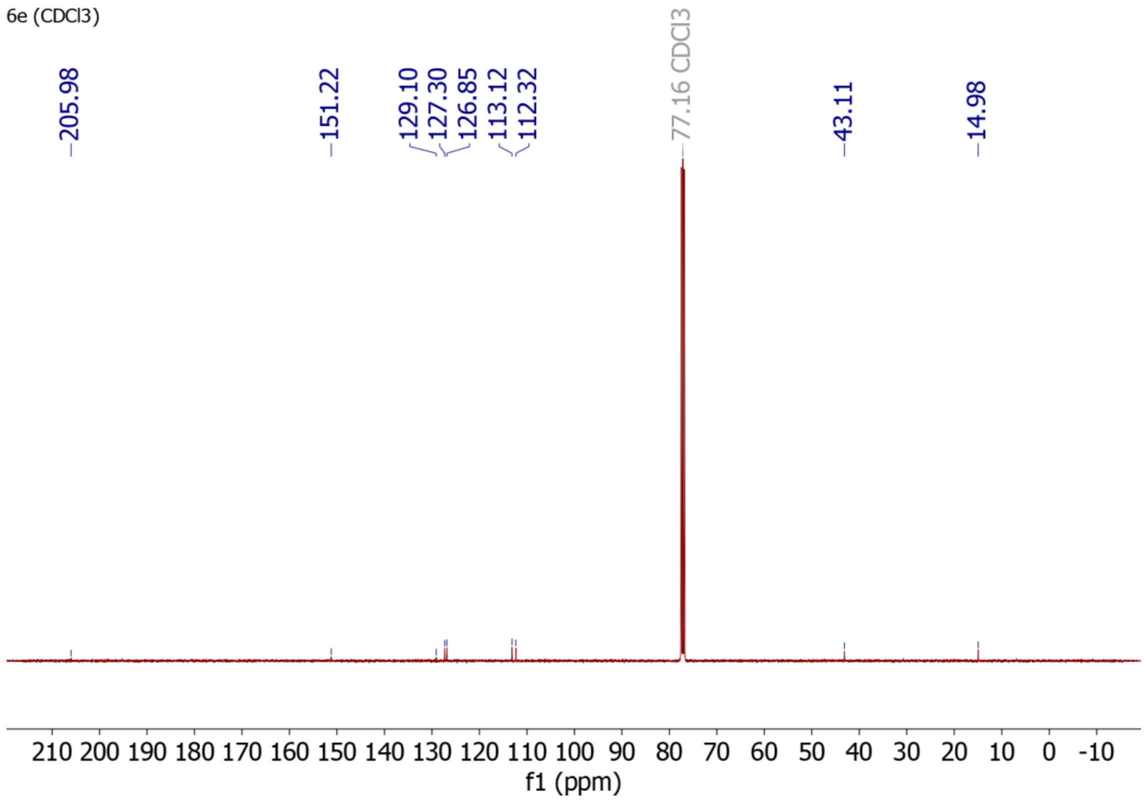

Supplementary Figure 43. ^{13}C NMR spectrum of **Au3^{O}**.

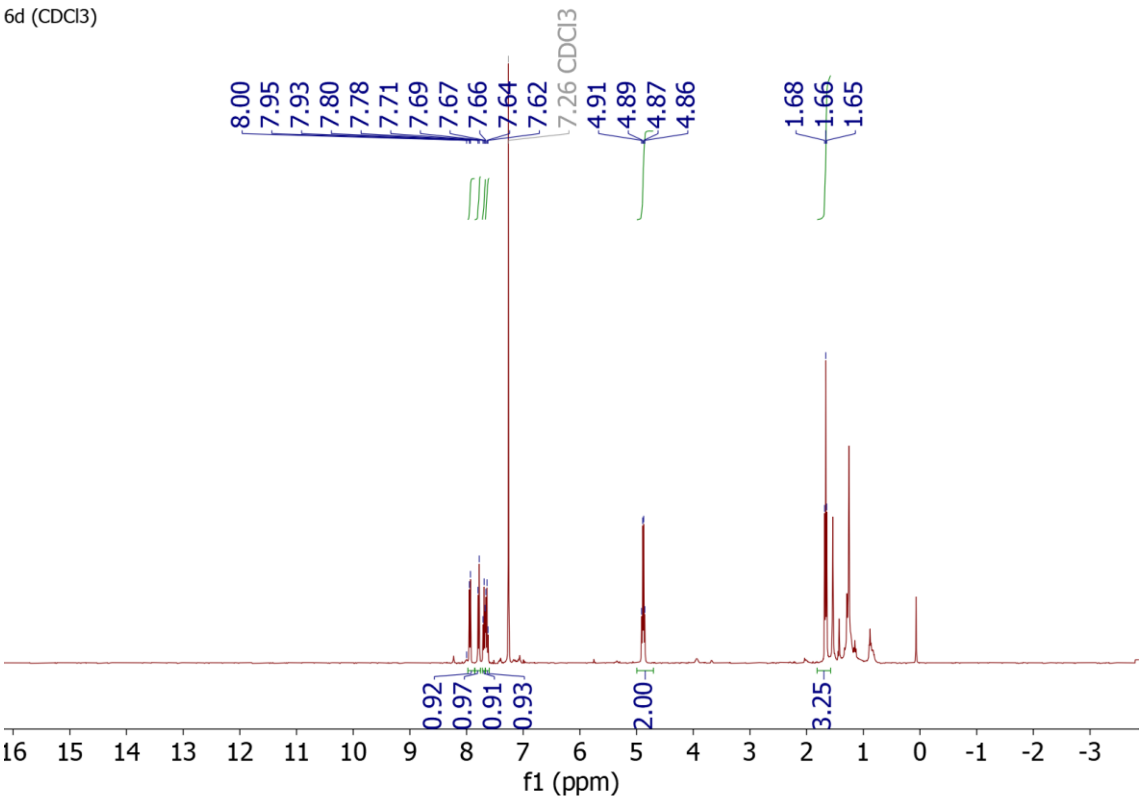

Supplementary Figure 44. ^{1}H NMR spectrum of **Au3^{S}**.

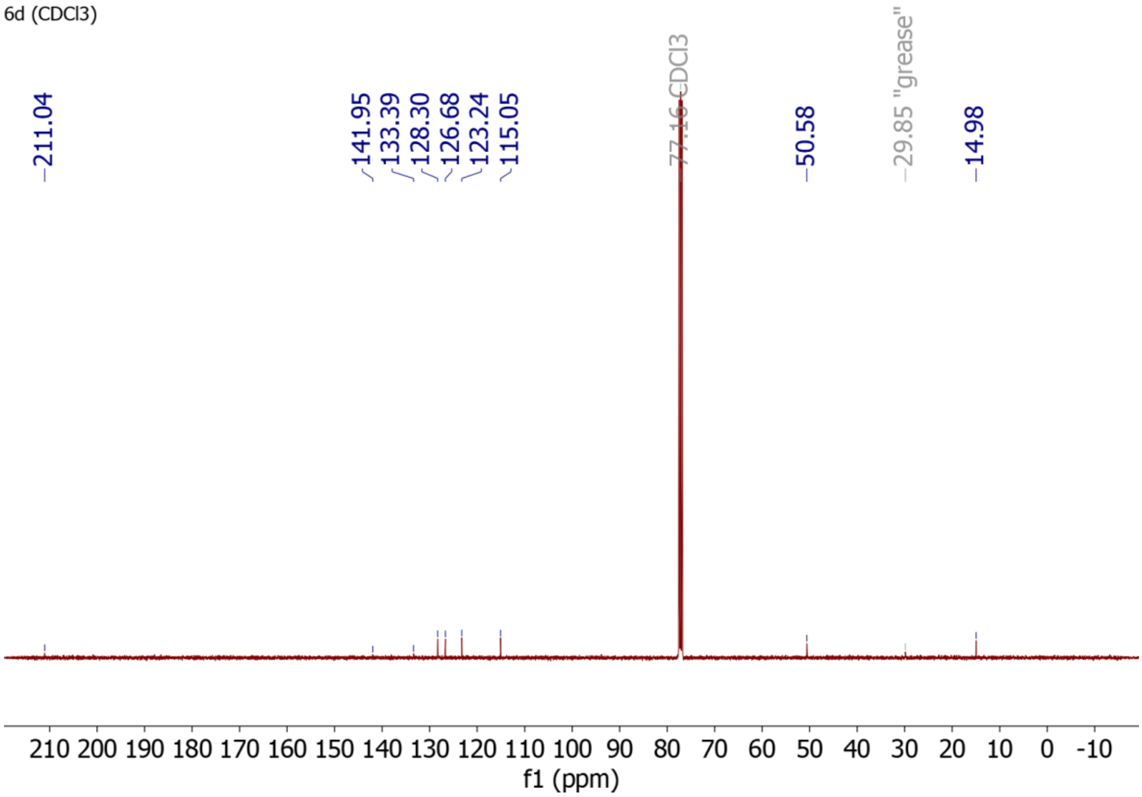

Supplementary Figure 45. ^{13}C NMR spectrum of **Au3^{S}**.

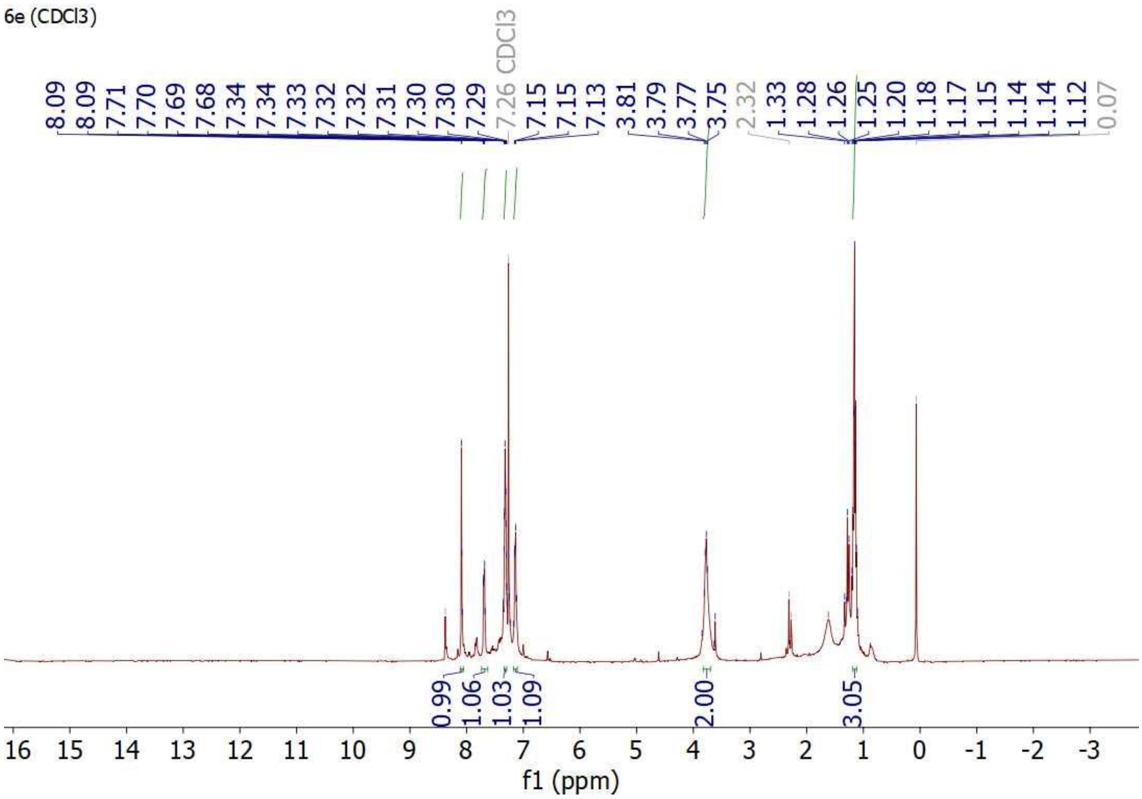

Supplementary Figure 46. ^{1}H NMR spectrum of **Au3^{Se}**.

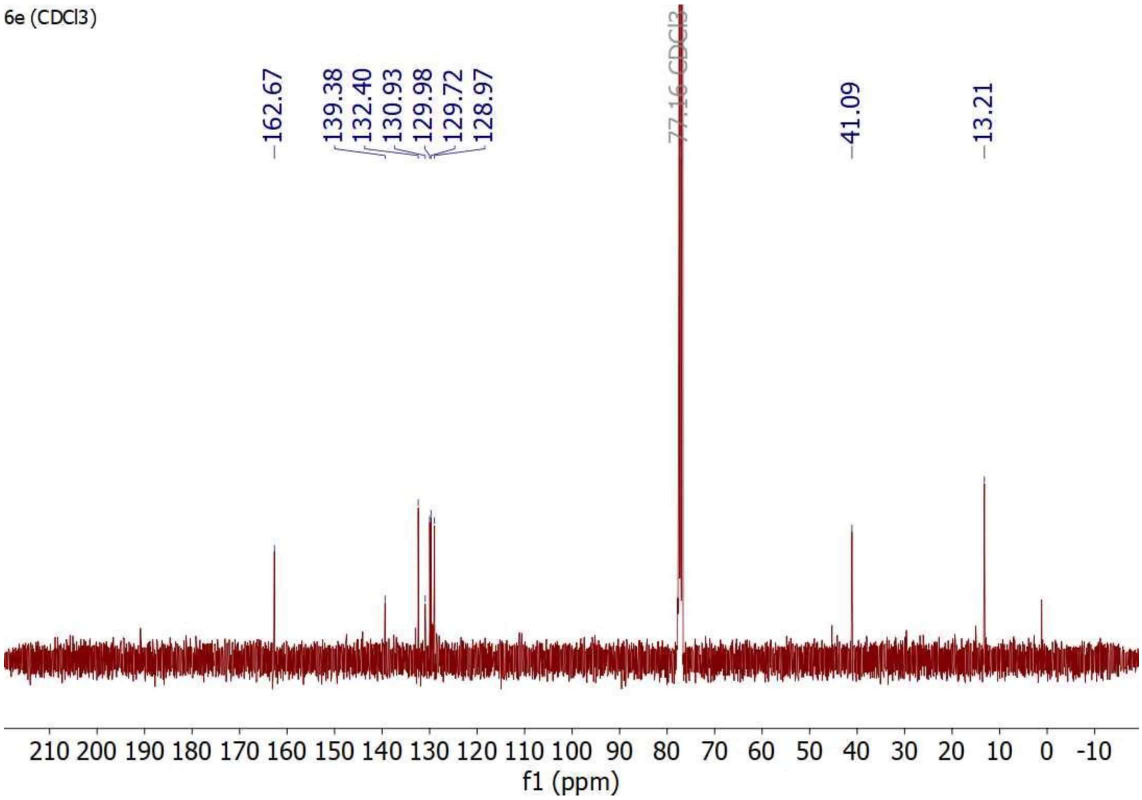

Supplementary Figure 47. ^{13}C NMR spectrum of **Au3^{Se}**.

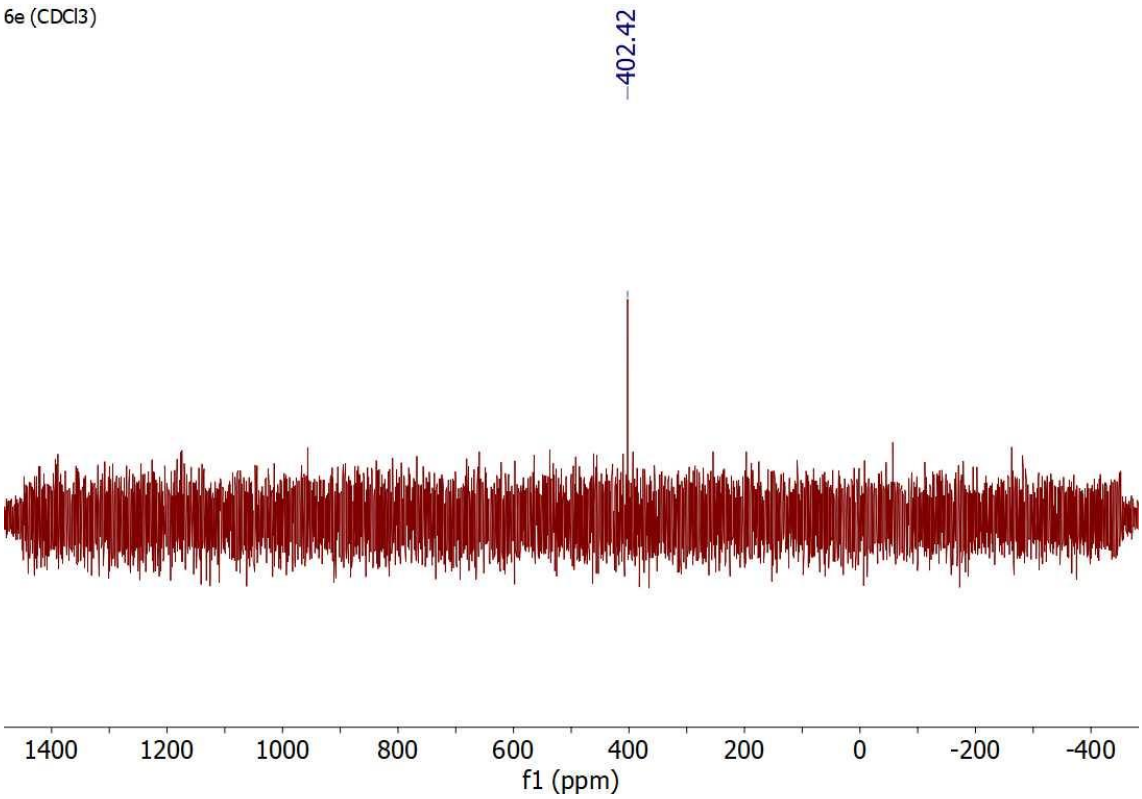

Supplementary Figure 48. ^{77}Se NMR spectrum of **Au3^{Se}**.

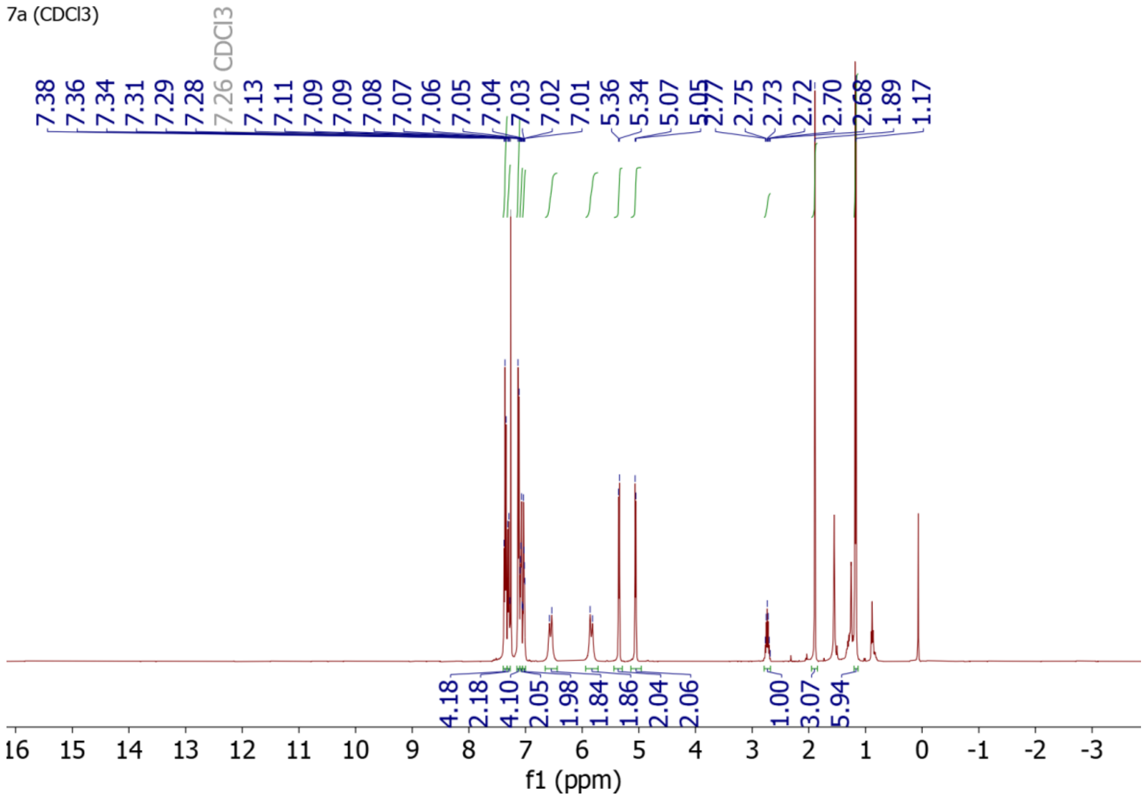

Supplementary Figure 49. ^{1}H NMR spectrum of **Ru4^{NBn}**.

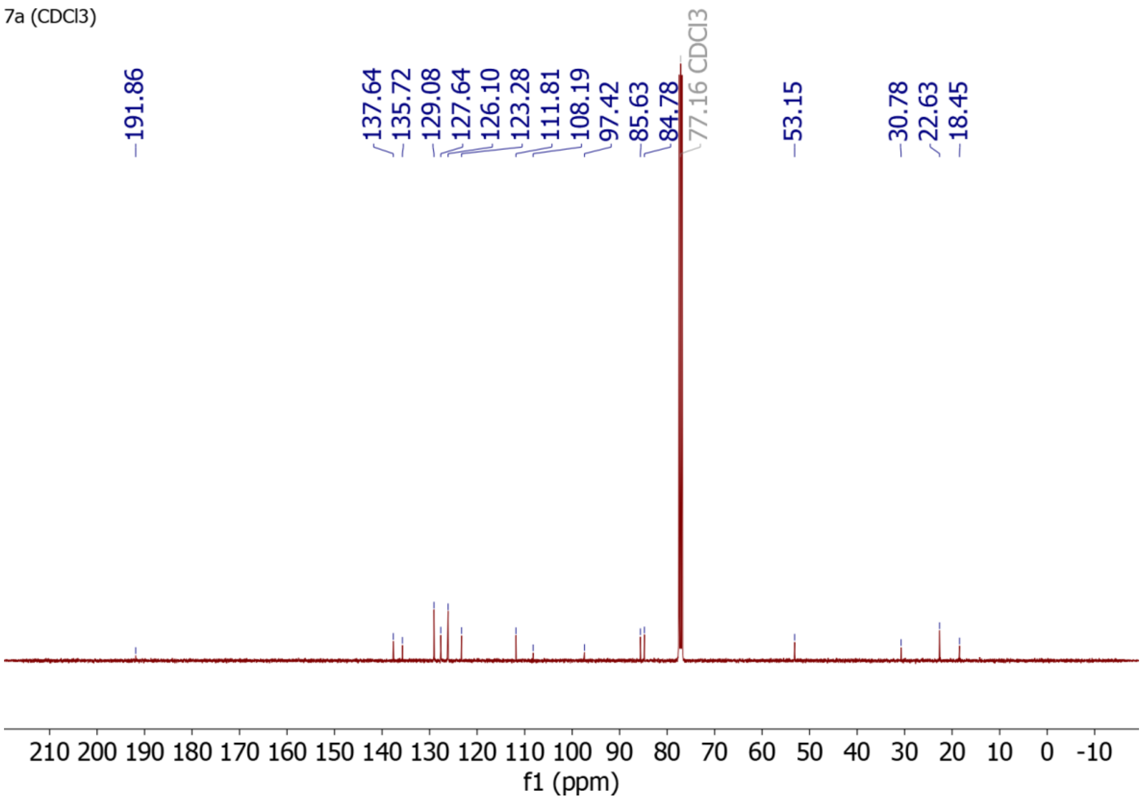

Supplementary Figure 50. ^{13}C NMR spectrum of **Ru4^{NBn}**.

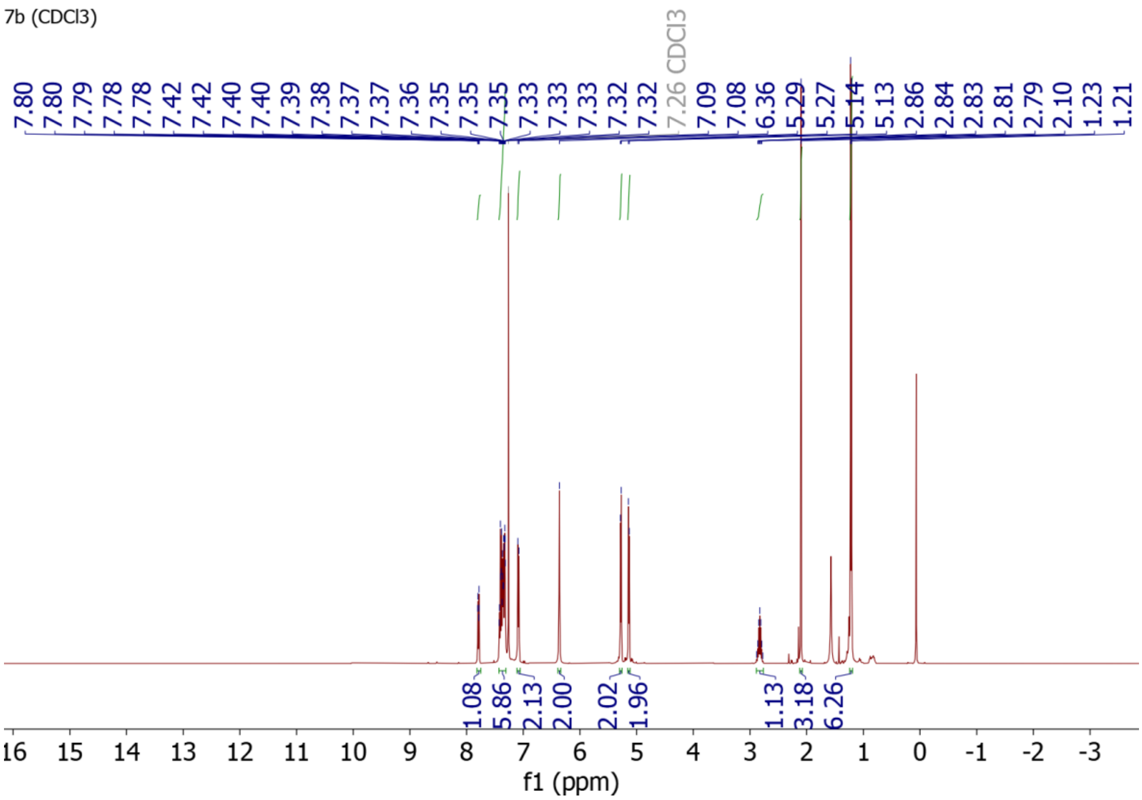

Supplementary Figure 51. ^{1}H NMR spectrum of **Ru4^{S}**.

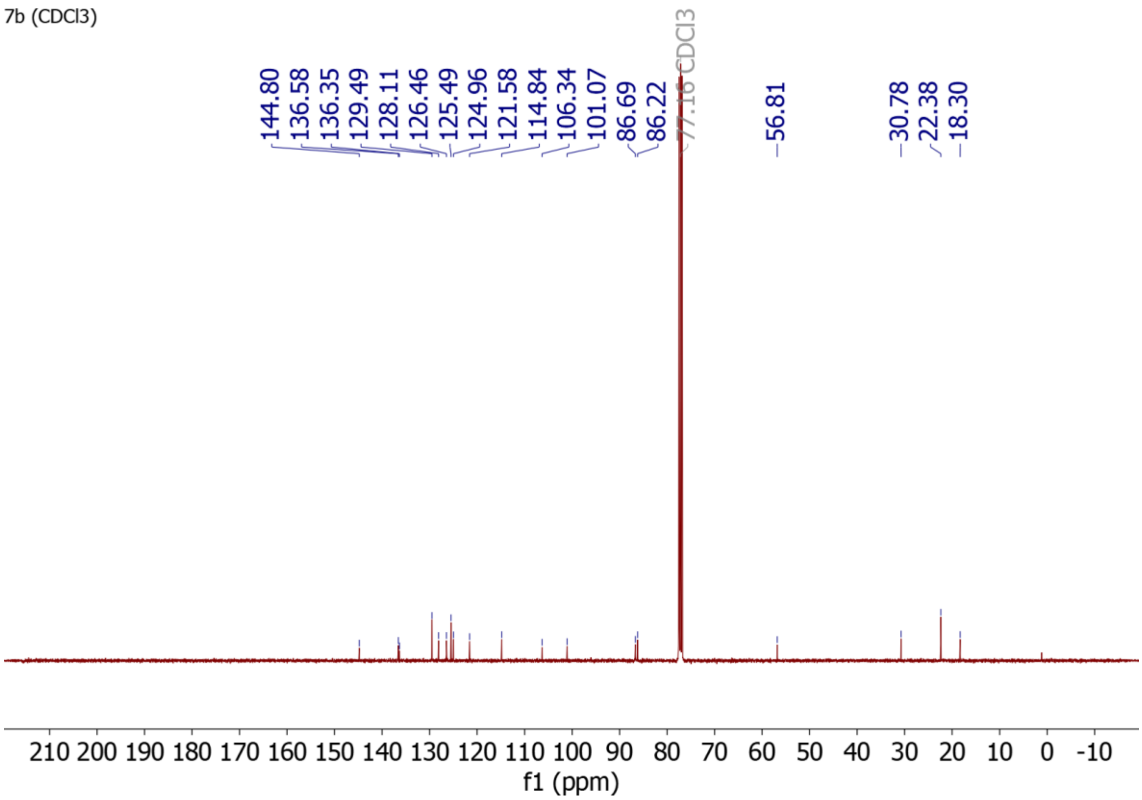

Supplementary Figure 52. ^{13}C NMR spectrum of **Ru4^{S}**.

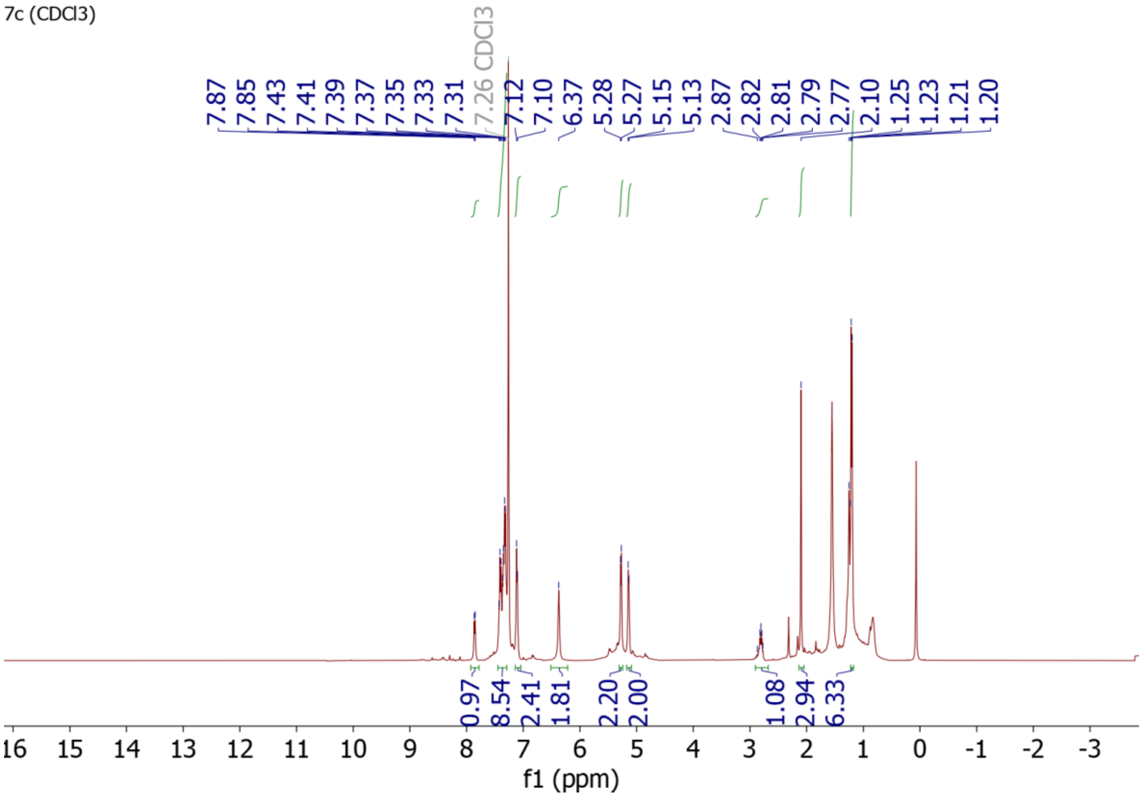

Supplementary Figure 53. ^{1}H NMR spectrum of **Ru4^{Se}**.

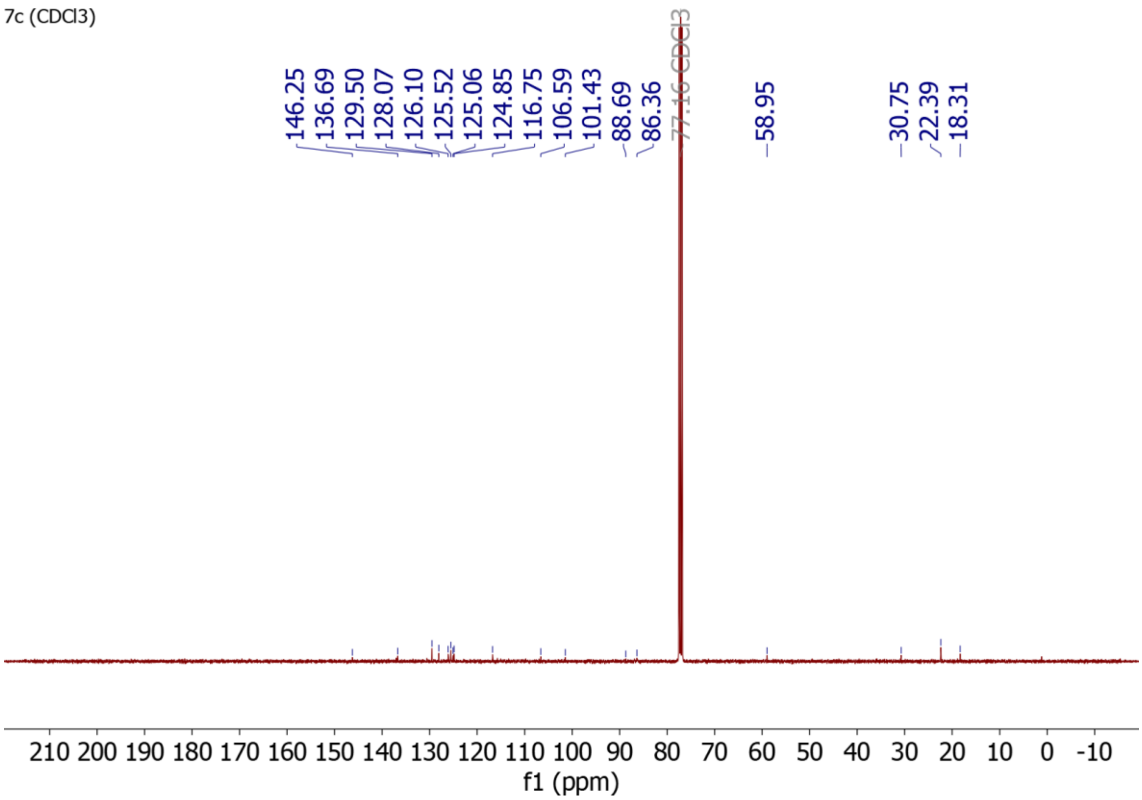

Supplementary Figure 54. ^{13}C NMR spectrum of **Ru4^{Se}**.

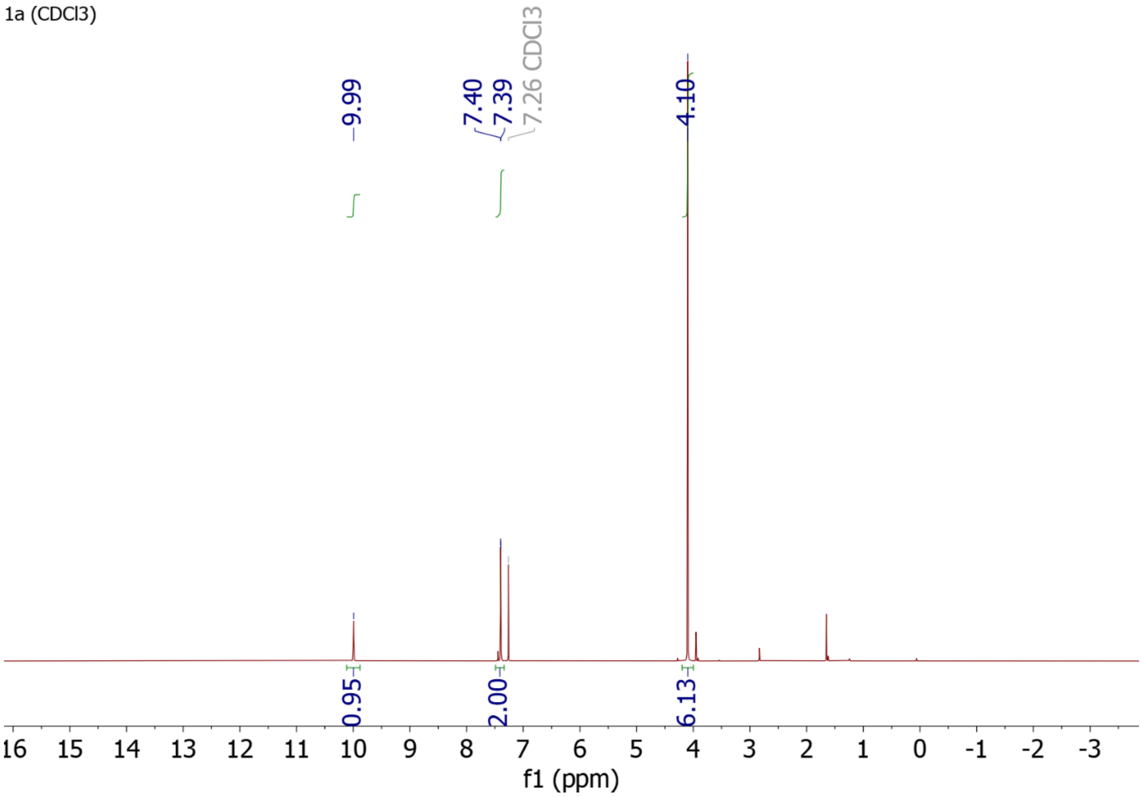

Supplementary Figure 55. ^{1}H NMR spectrum of **1^{NMe}·HI**.

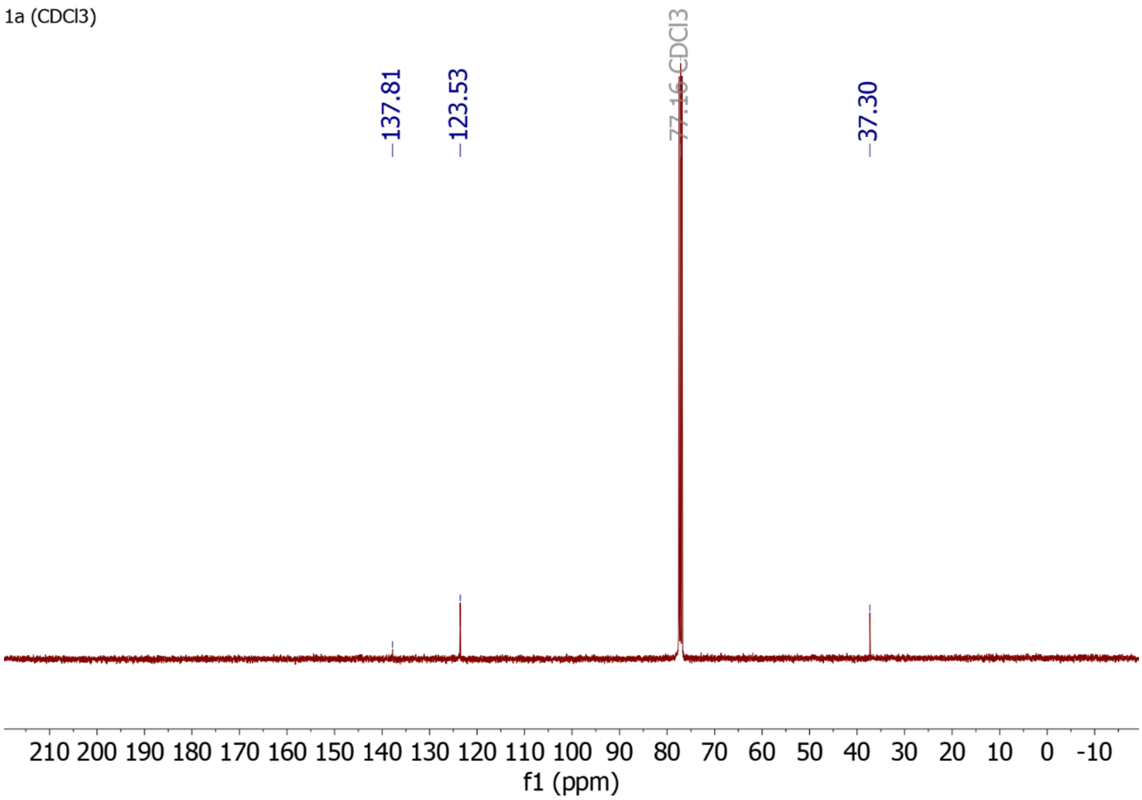

Supplementary Figure 56. ^{13}C NMR spectrum of $\mathbf{1^{NMe}}$**·HI**.

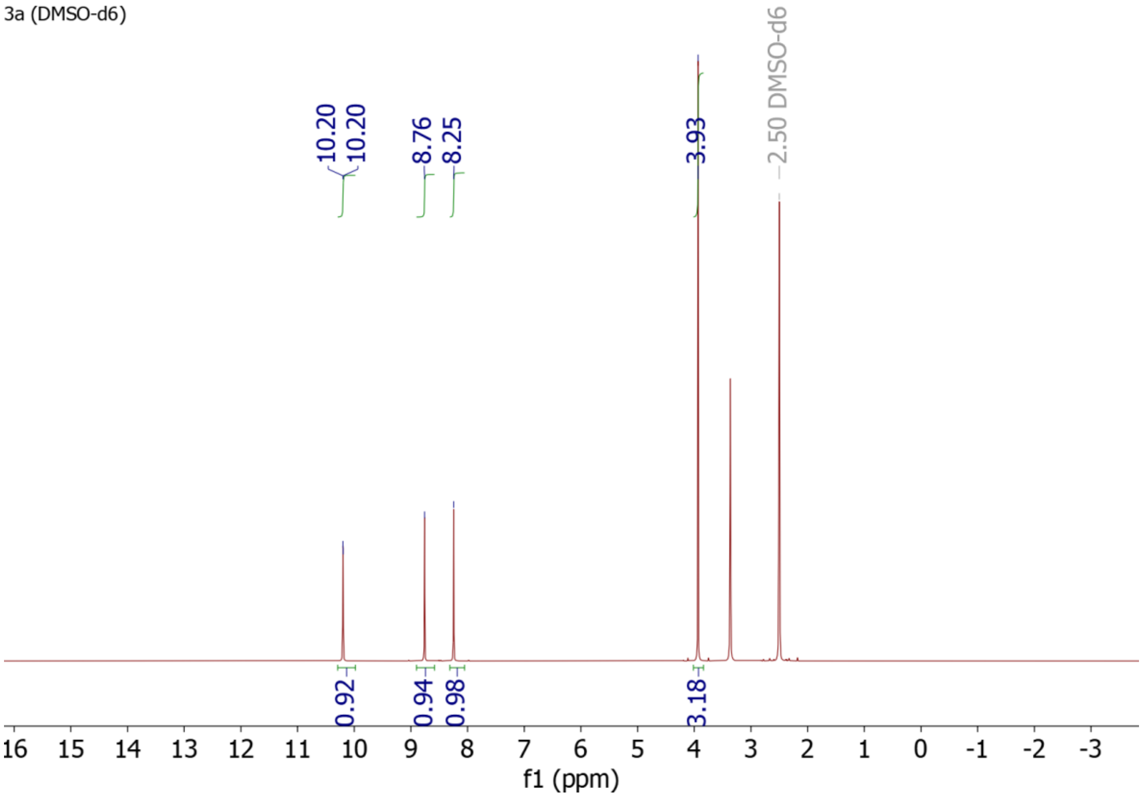

Supplementary Figure 57. ^{1}H NMR spectrum of $\mathbf{1^{O}}$**·HI**.

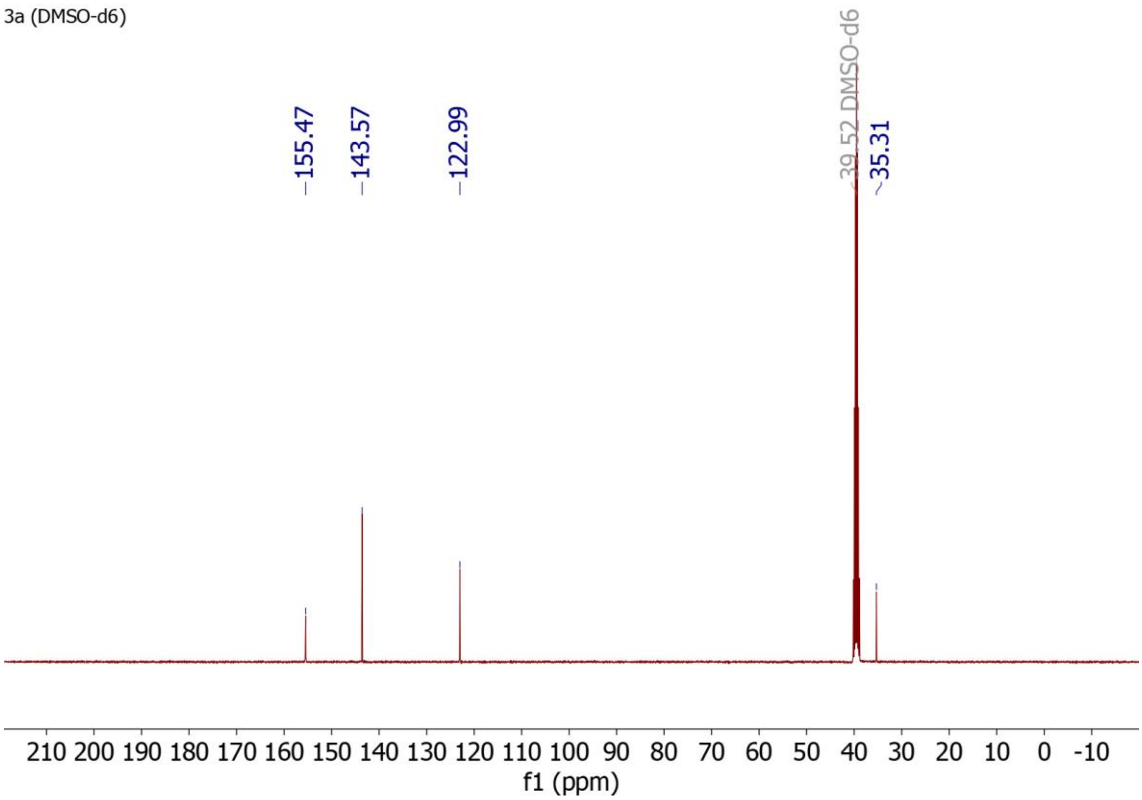

Supplementary Figure 58. ^{13}C NMR spectrum of **1^{O}·HI**.

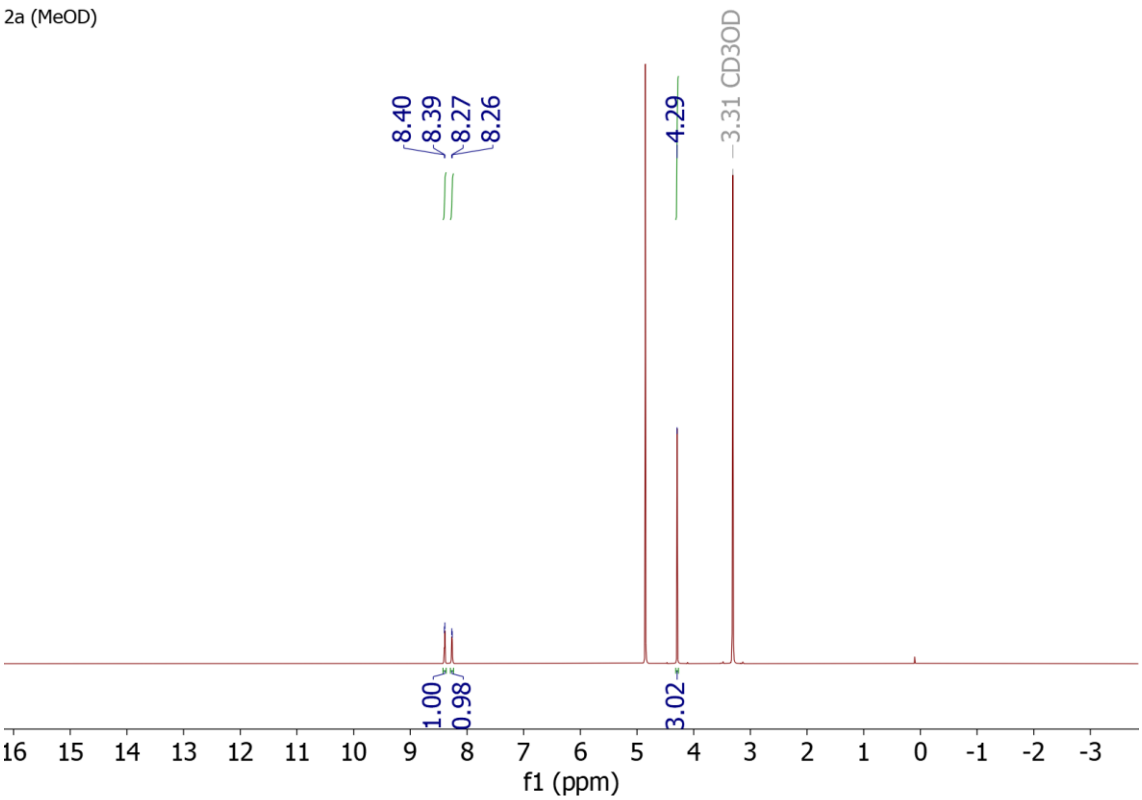

Supplementary Figure 59. ^{1}H NMR spectrum of **1^{S}·HI**.

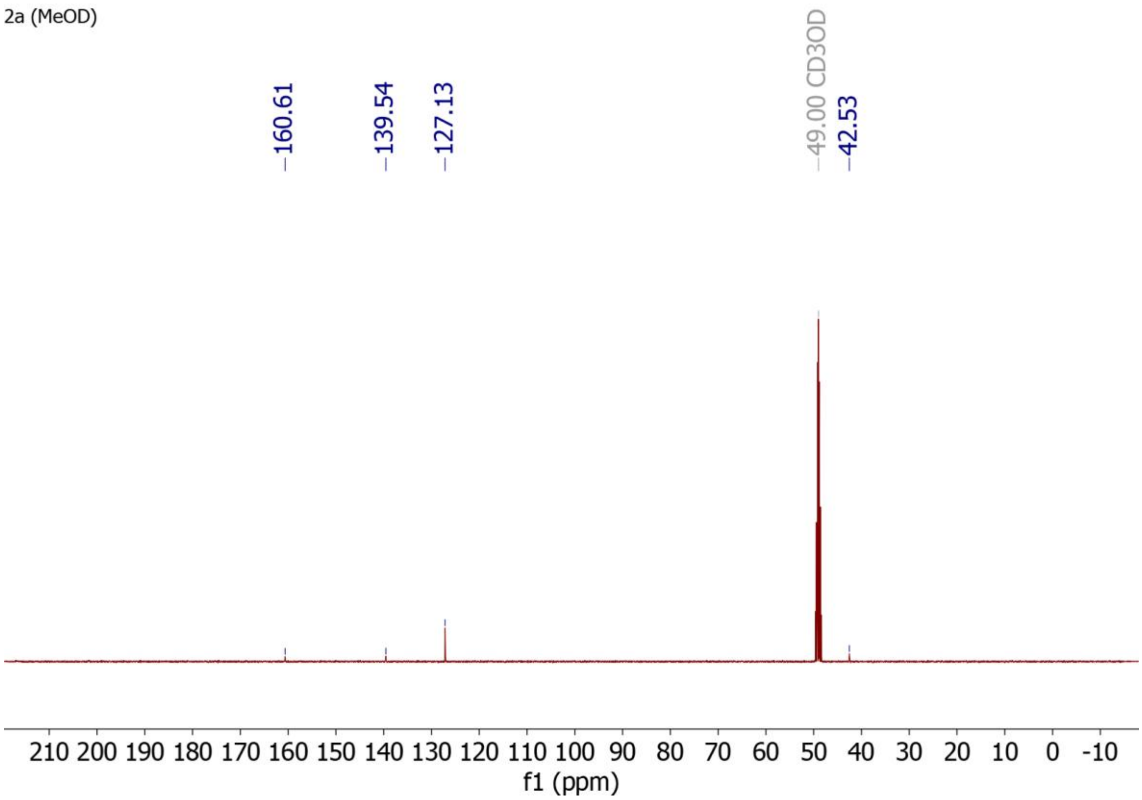

Supplementary Figure 60. ^{13}C NMR spectrum of **1^{S}·HI**.

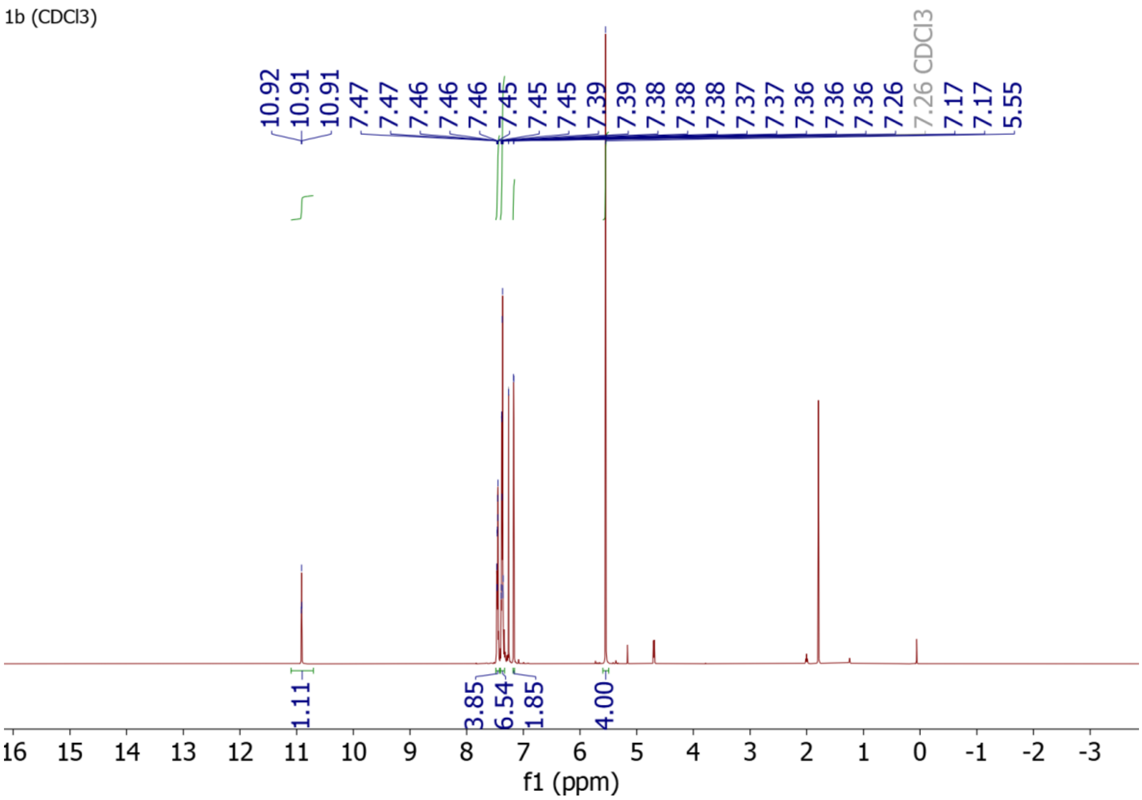

Supplementary Figure 61. ^{1}H NMR spectrum of **2^{NBn}·HBr**.

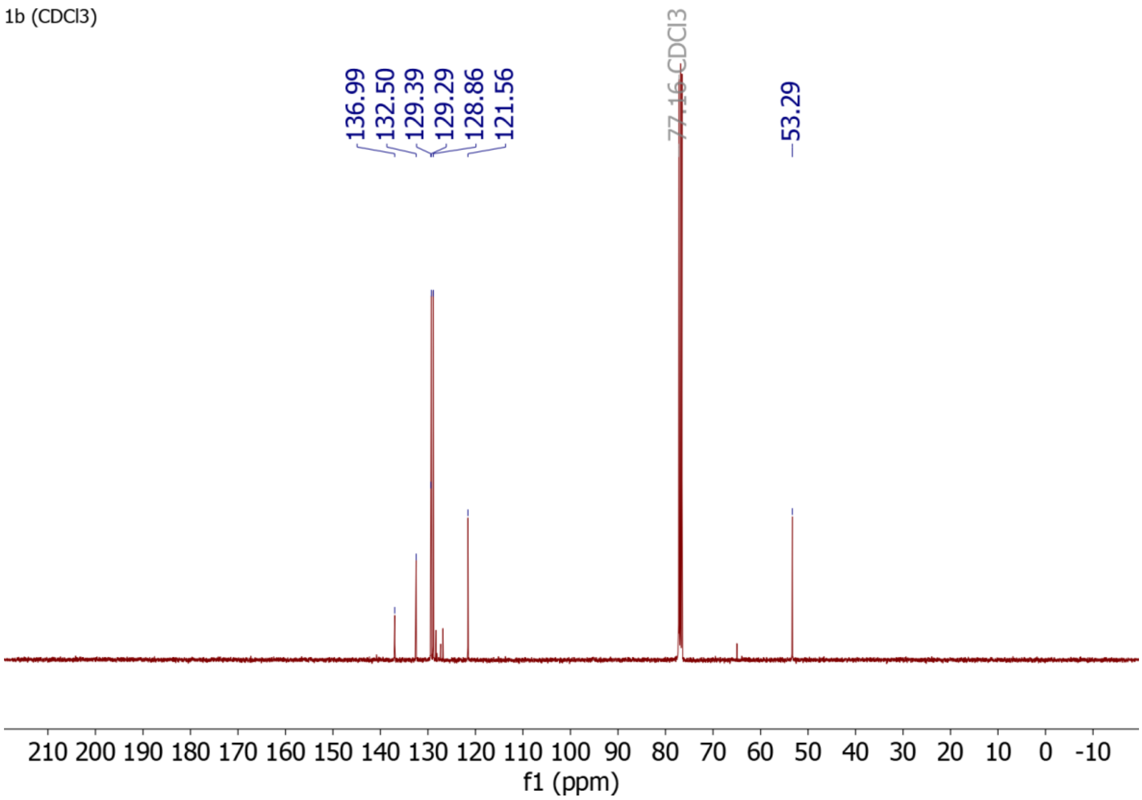

Supplementary Figure 62. ^{13}C NMR spectrum of $\mathbf{2^{NBn}}$**·HBr**.

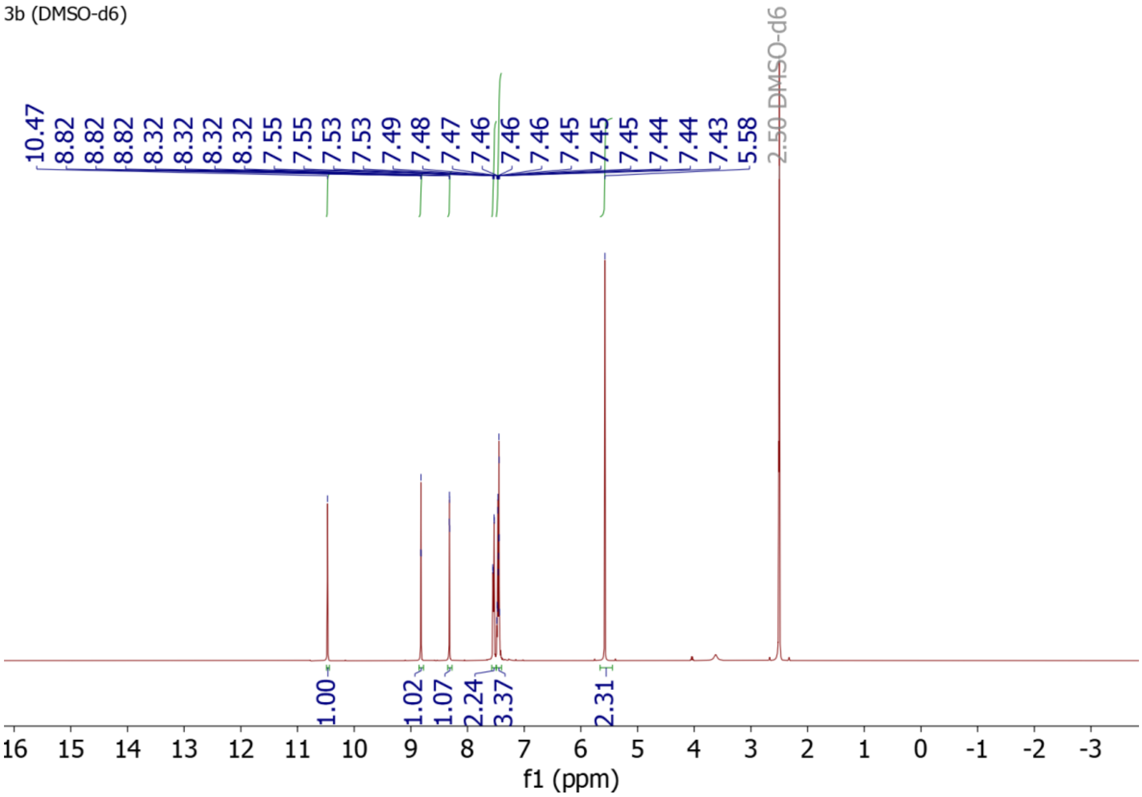

Supplementary Figure 63. ^{1}H NMR spectrum of $\mathbf{2^{O}}$**·HBr**.

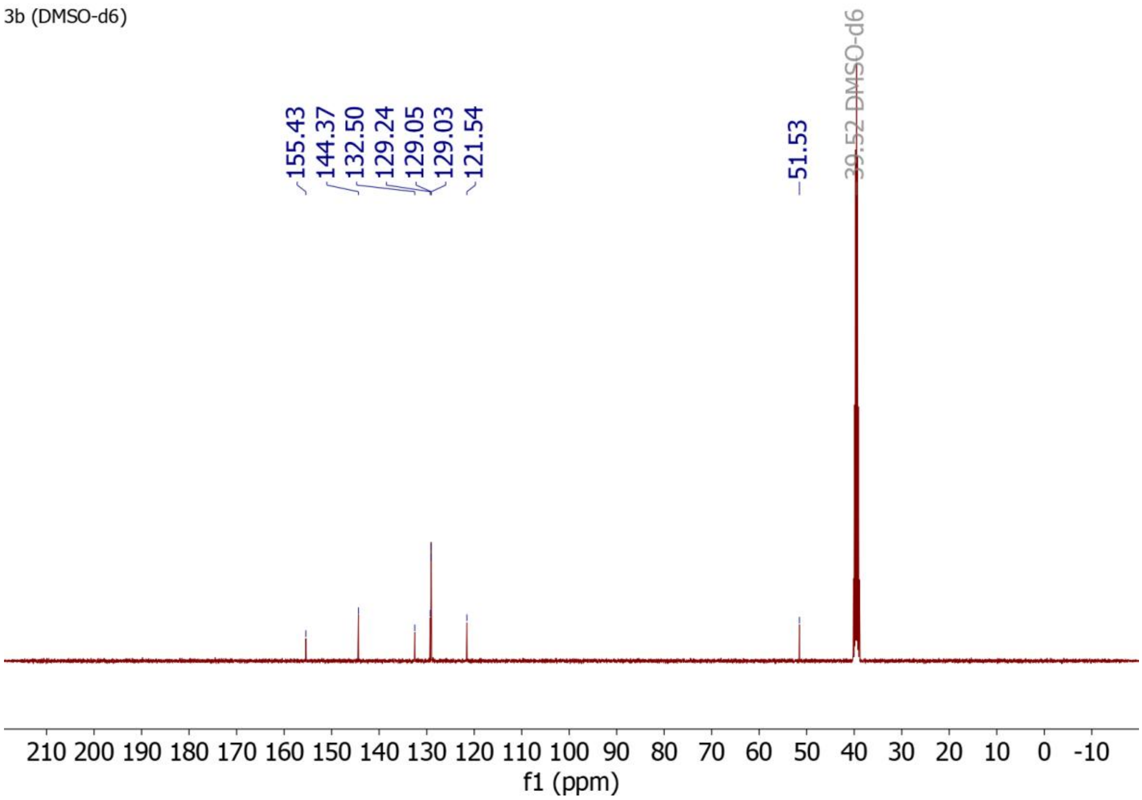

Supplementary Figure 64. ^{13}C NMR spectrum of **2^{O}·HBr**.

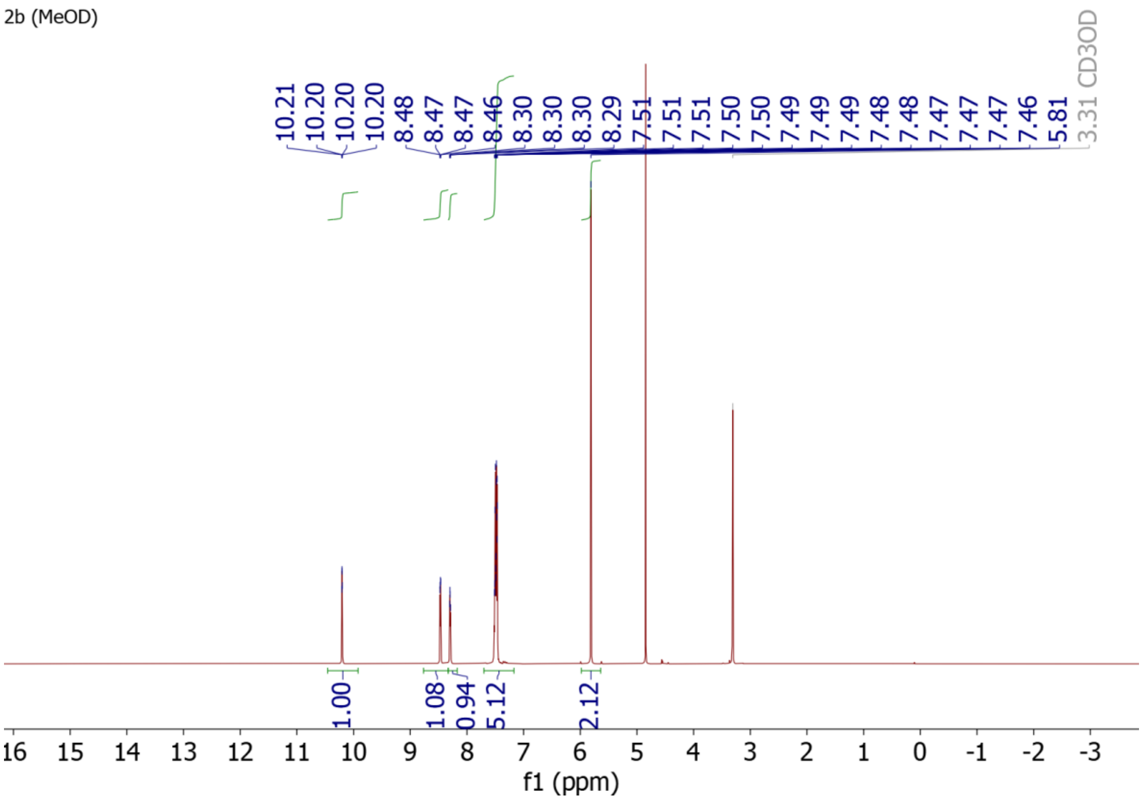

Supplementary Figure 65. ^{1}H NMR spectrum of **2^{S}·HBr**.

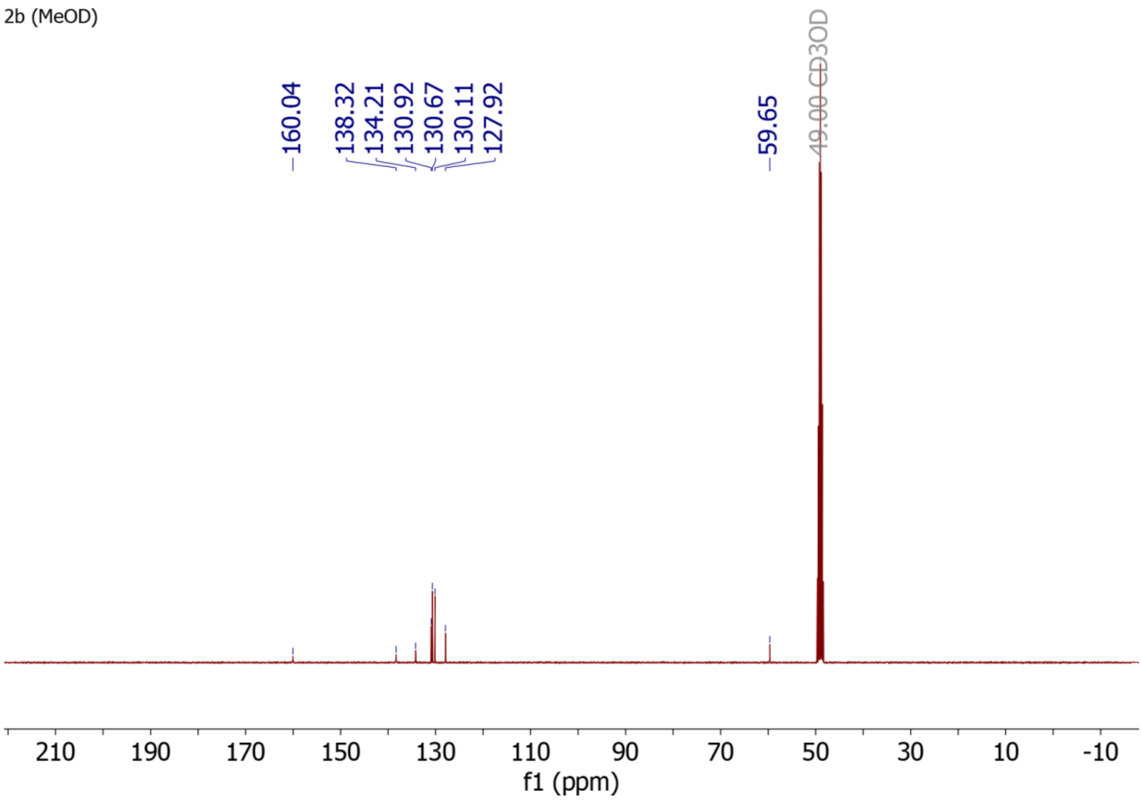

Supplementary Figure 66. ^{13}C NMR spectrum of **2^{S}·HBr**.

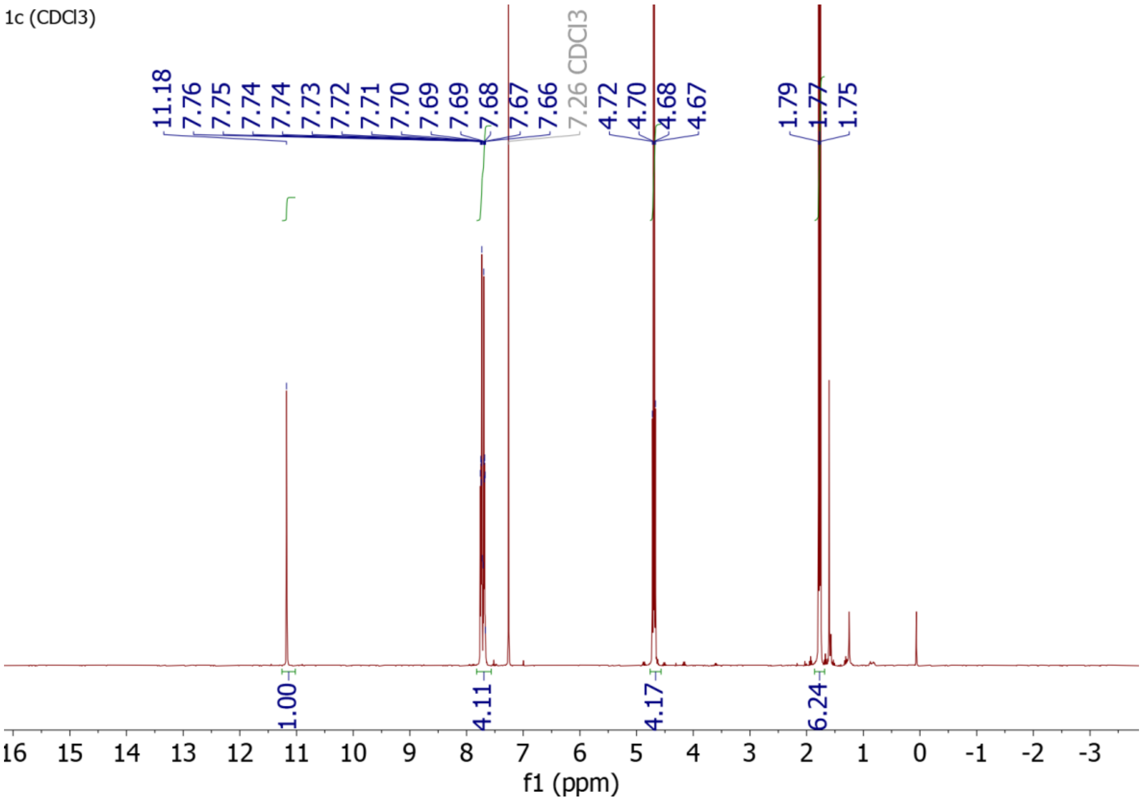

Supplementary Figure 67. ^{1}H NMR spectrum of **3^{NEt}·HI**.

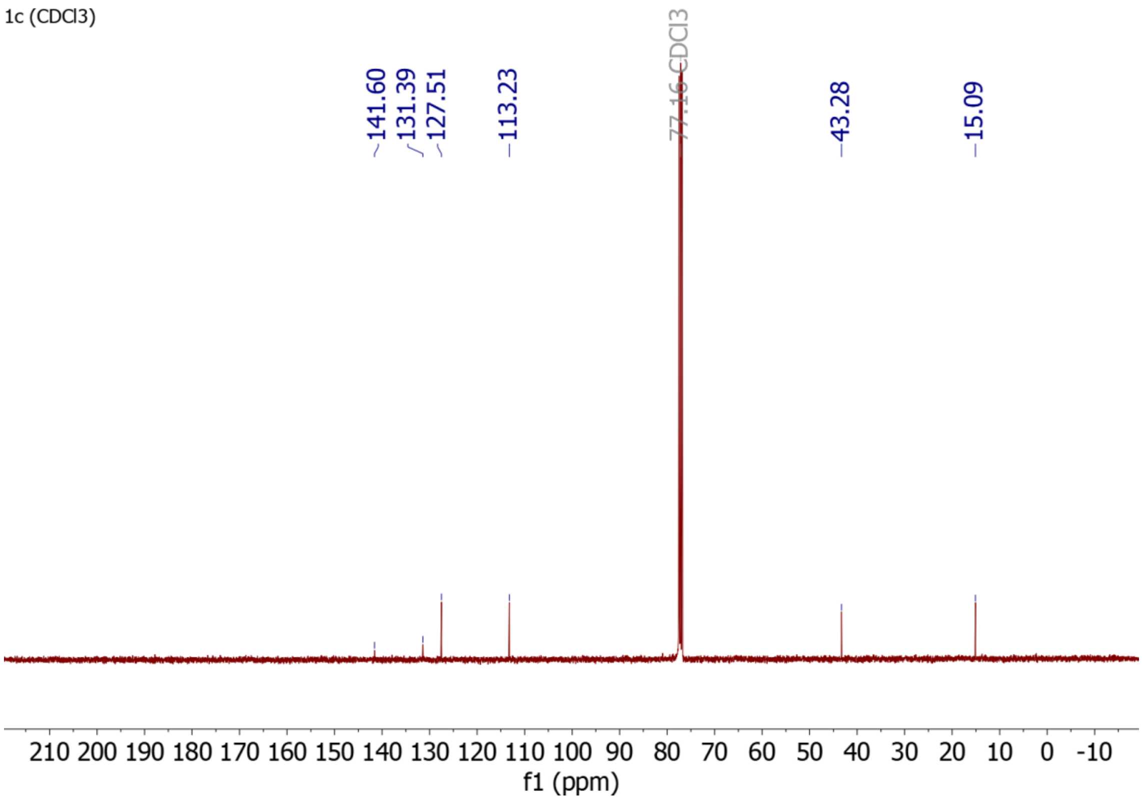

Supplementary Figure 68. ^{13}C NMR spectrum of $\mathbf{3^{NEt}{\cdot}HI}$.

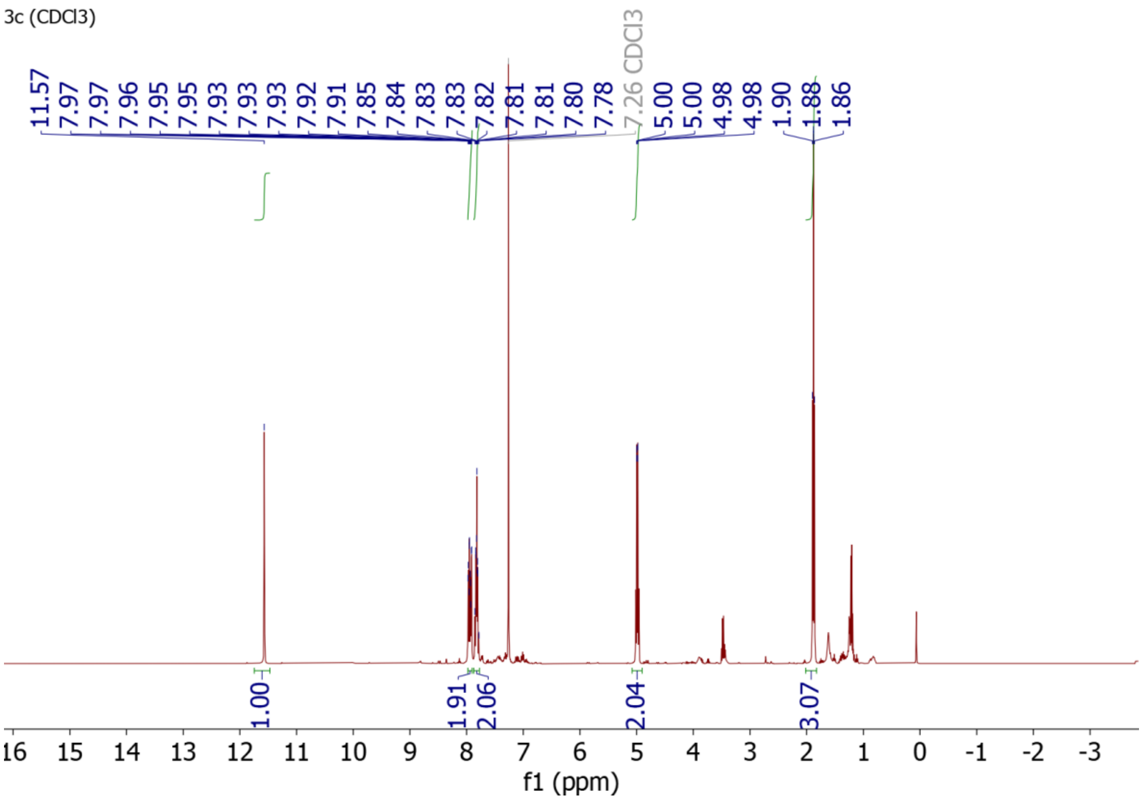

Supplementary Figure 69. ^{1}H NMR spectrum of $\mathbf{3^{O}{\cdot}HI}$.

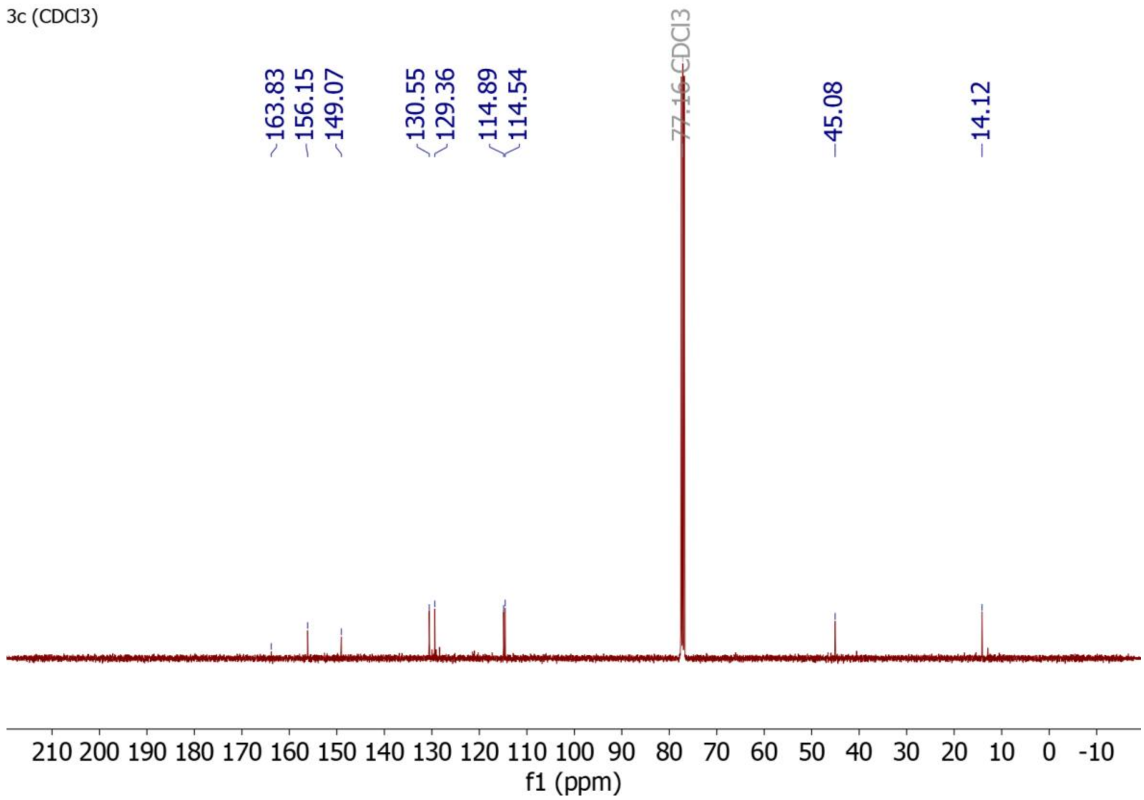

Supplementary Figure 70. ^{13}C NMR spectrum of **3°·HI**.

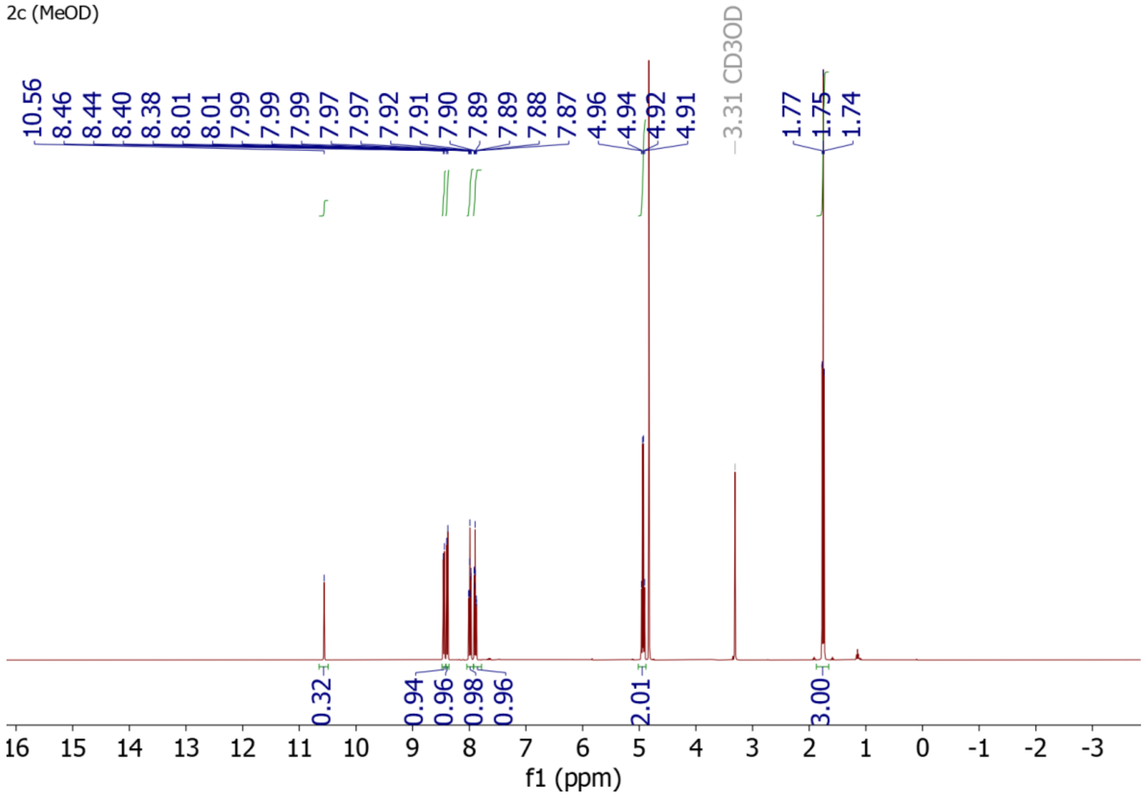

Supplementary Figure 71. ^{1}H NMR spectrum of **3^{S}·HI**.

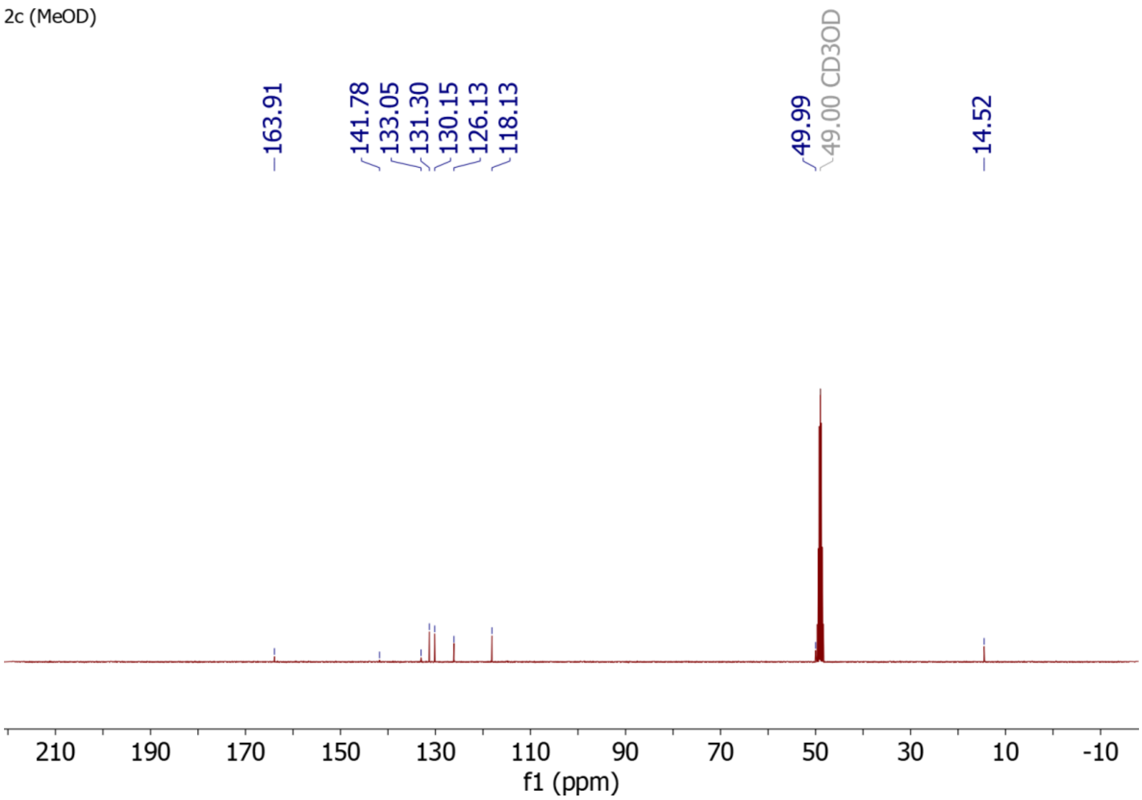

Supplementary Figure 72. ^{13}C NMR spectrum of **3^{S}·HI**.

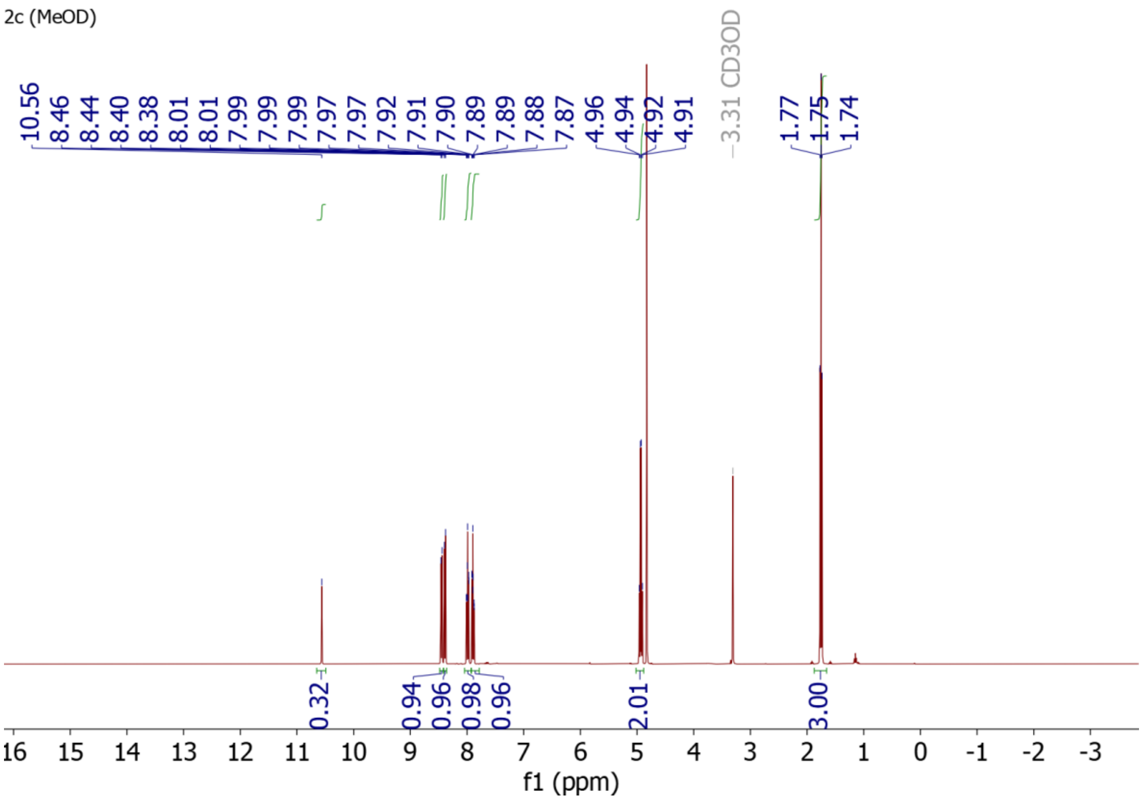

Supplementary Figure 73. ^{1}H NMR spectrum of **3^{S}·HBr**.

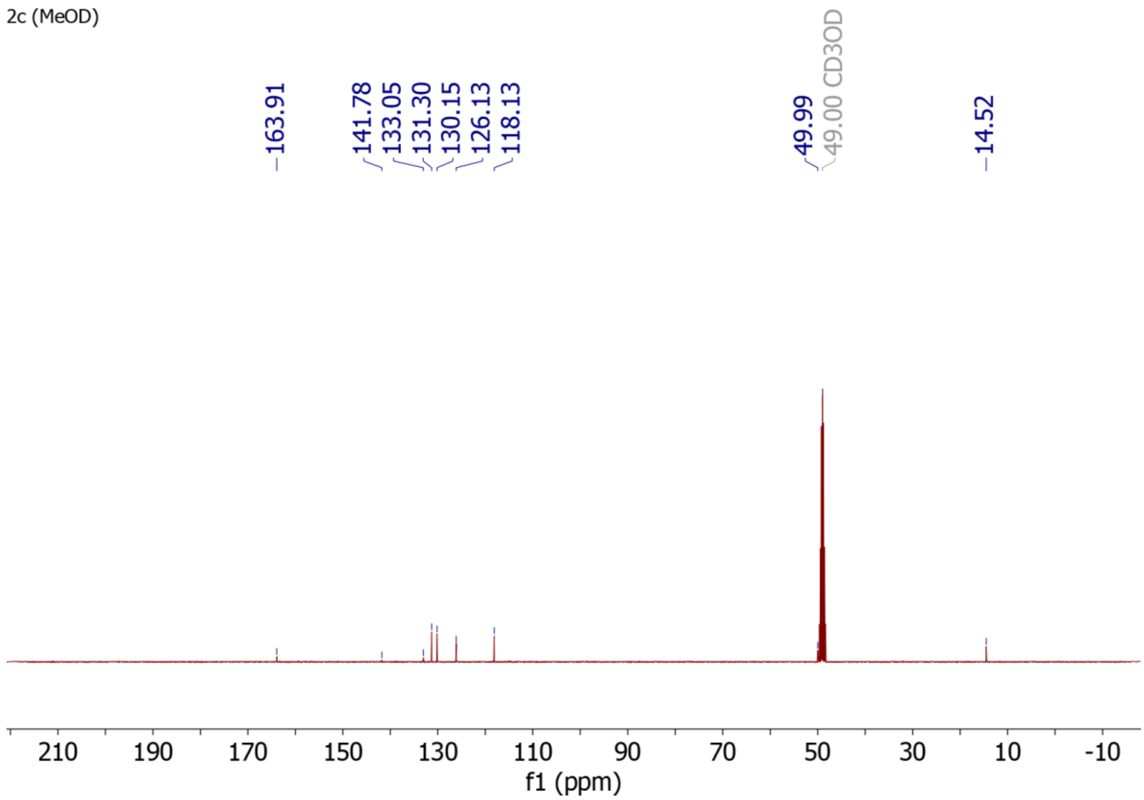

Supplementary Figure 74. ^{13}C NMR spectrum of **3^{S}·HBr**.

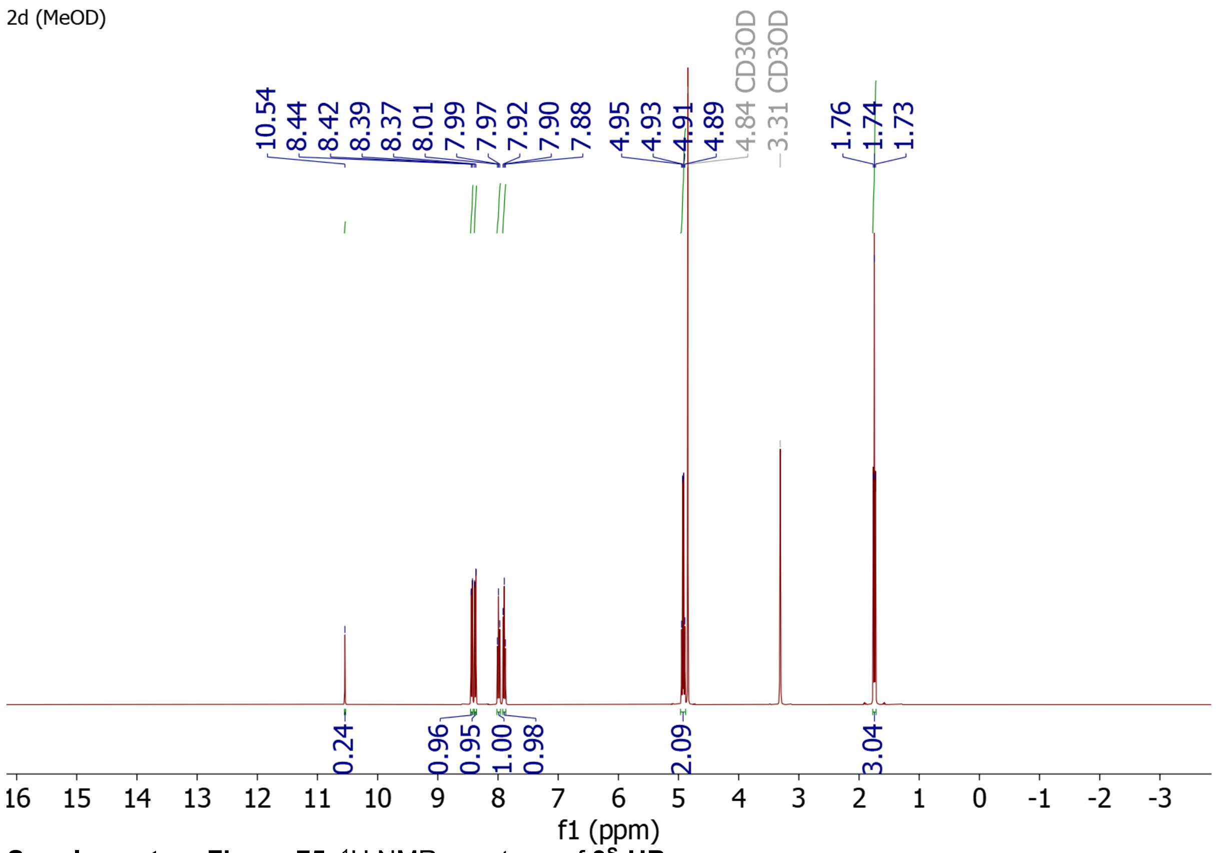

Supplementary Figure 75. ^{1}H NMR spectrum of **3^{S}·HBr**.

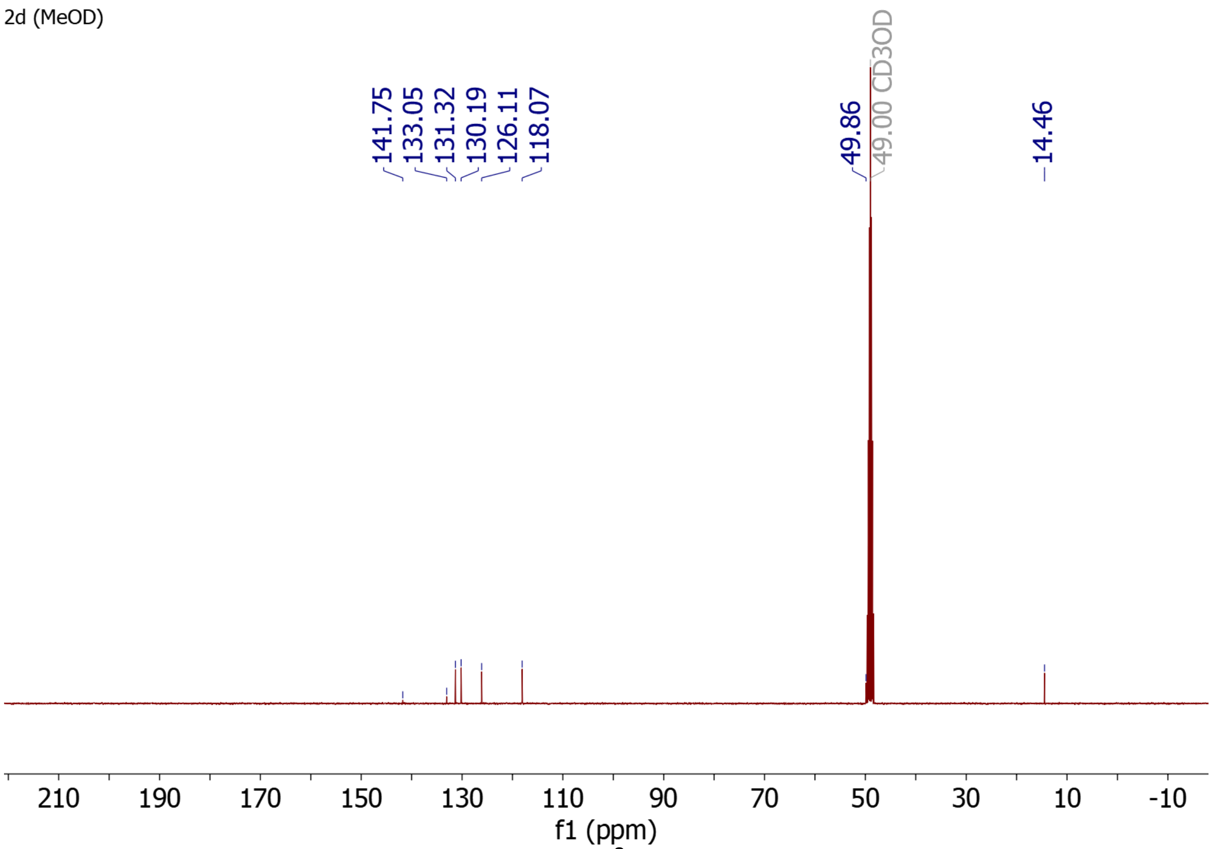

Supplementary Figure 76. ^{13}C NMR spectrum of $\mathbf{3^{S}{\cdot}HBr}$.

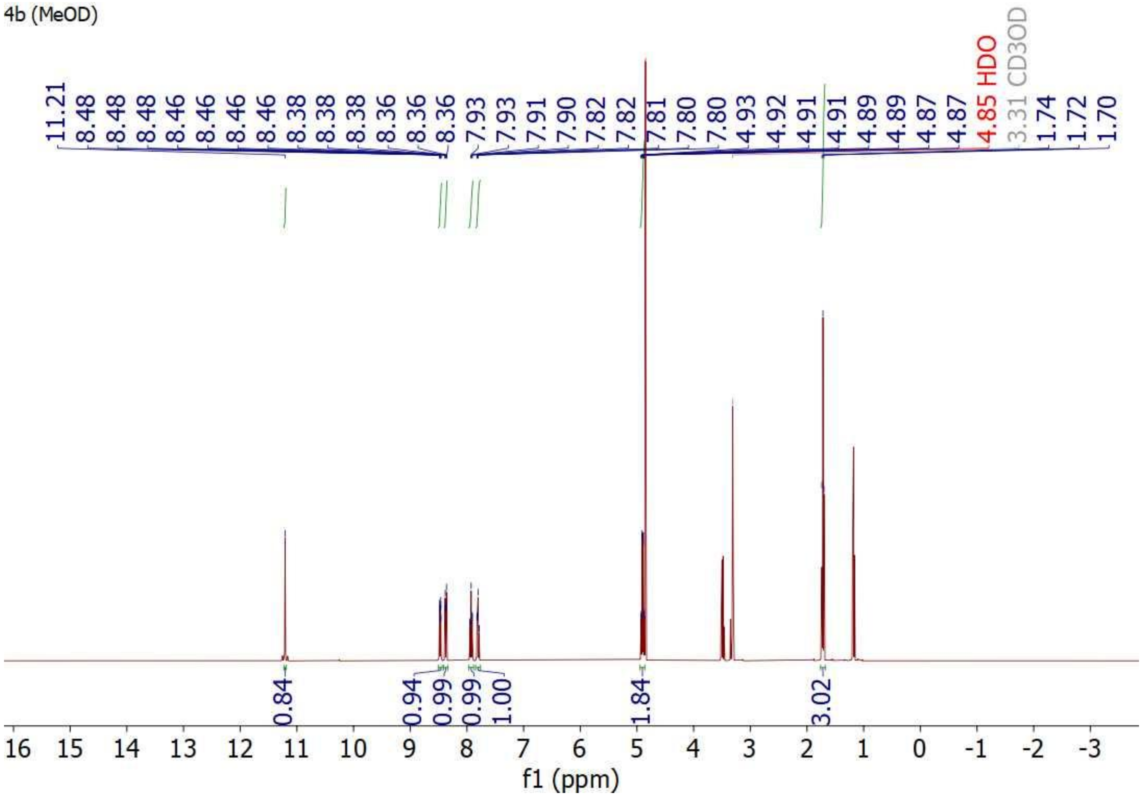

Supplementary Figure 77. ^{1}H NMR spectrum of $\mathbf{3^{Se}{\cdot}HI}$.

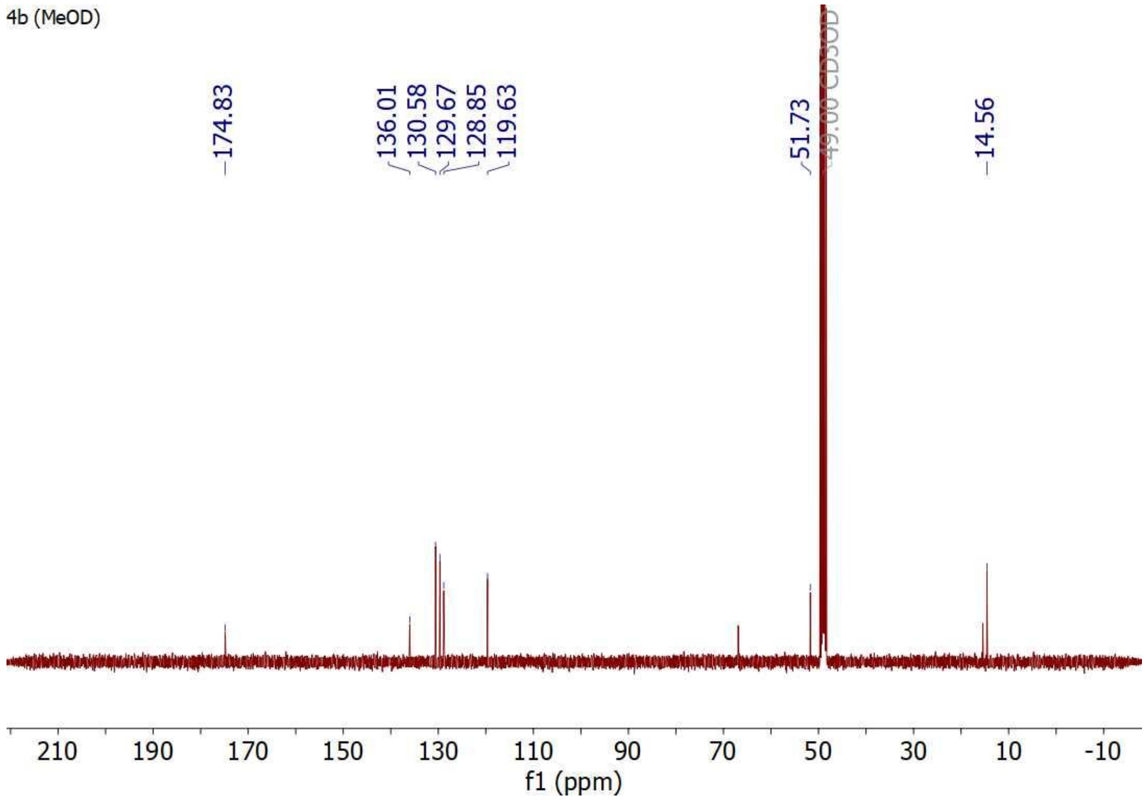

Supplementary Figure 78. ^{13}C NMR spectrum of $\mathbf{3^{Se}\cdot HI}$.

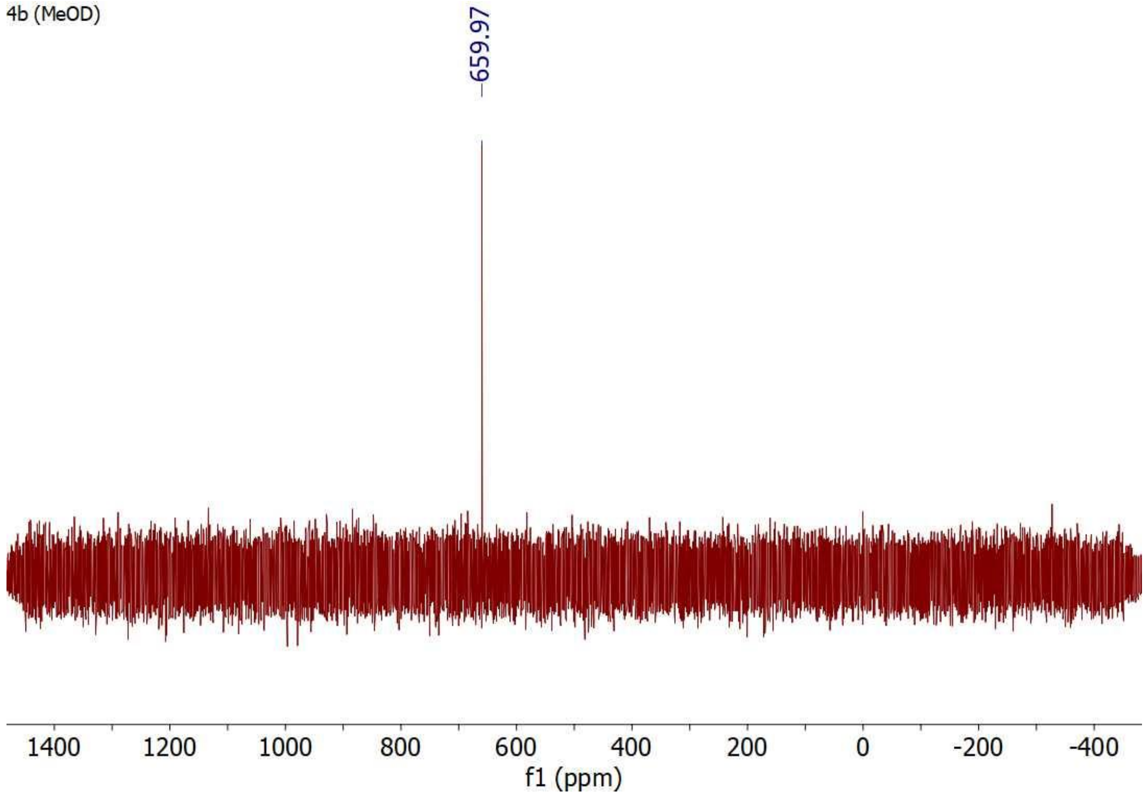

Supplementary Figure 79. ^{77}Se NMR spectrum of $\mathbf{3^{Se}\cdot HI}$.

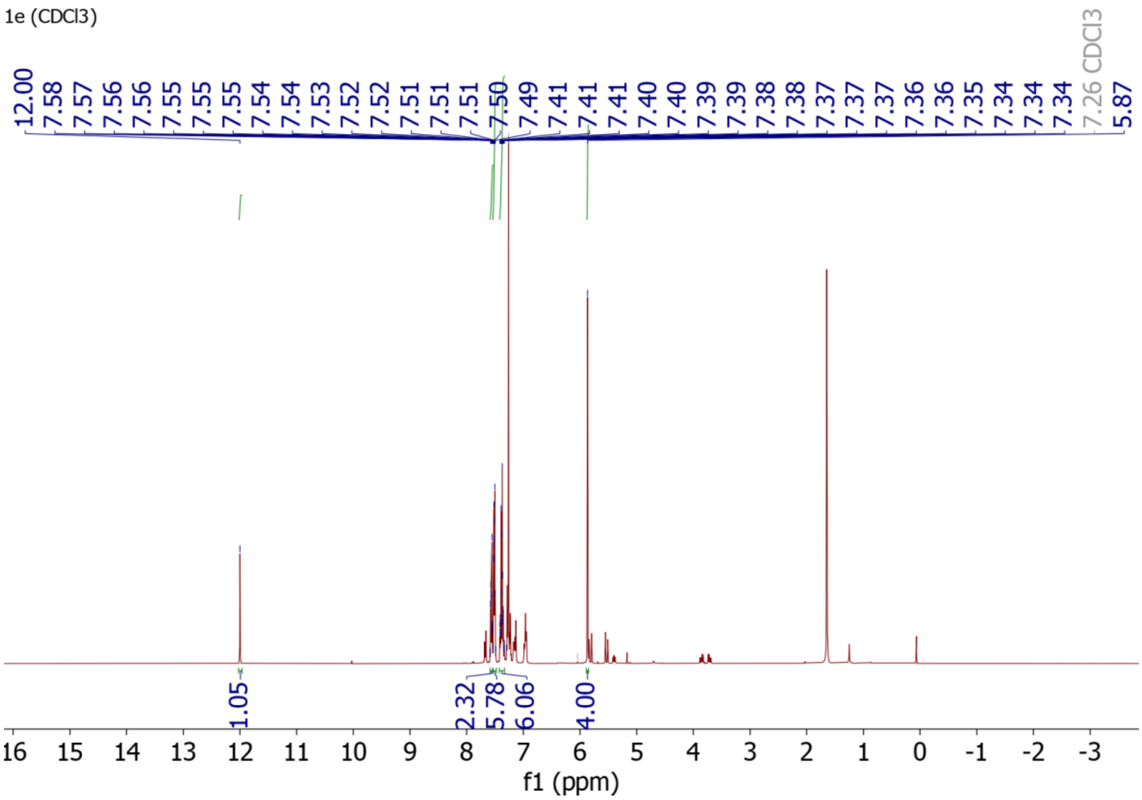

Supplementary Figure 80. ^{1}H NMR spectrum of **4^{NBn}·HBr**.

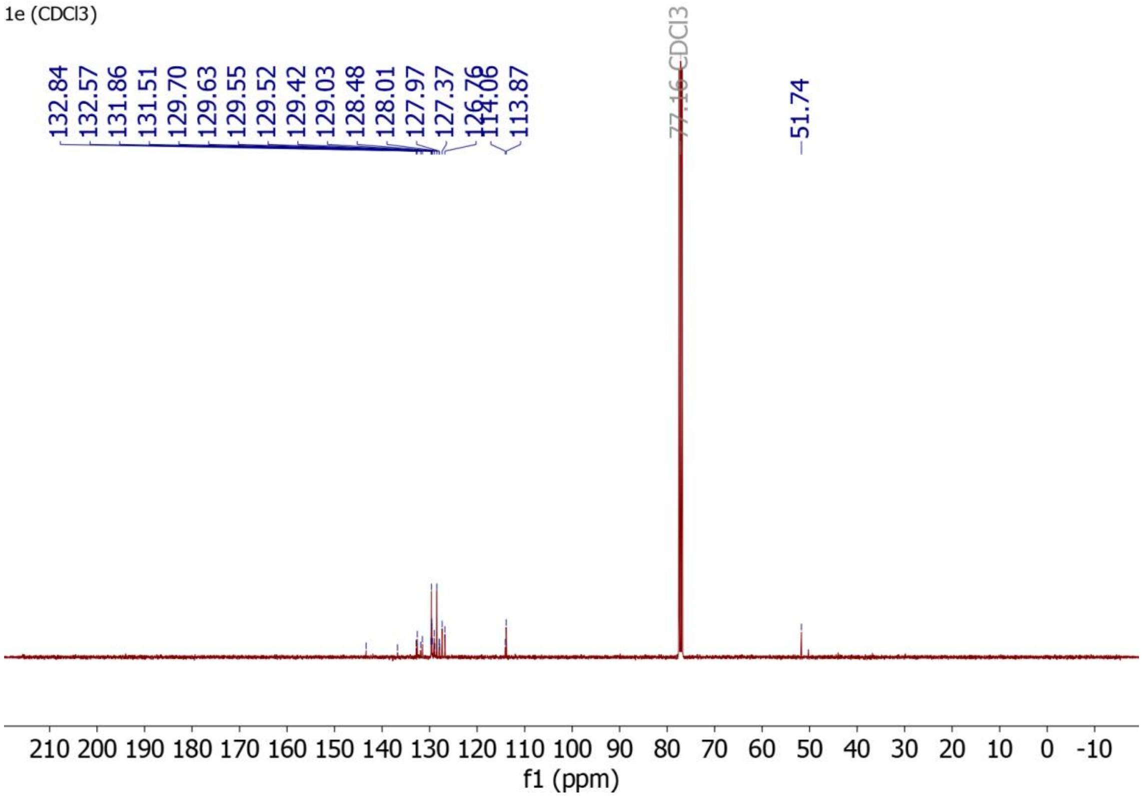

Supplementary Figure 81. ^{13}C NMR spectrum of **4^{NBn}·HBr**.

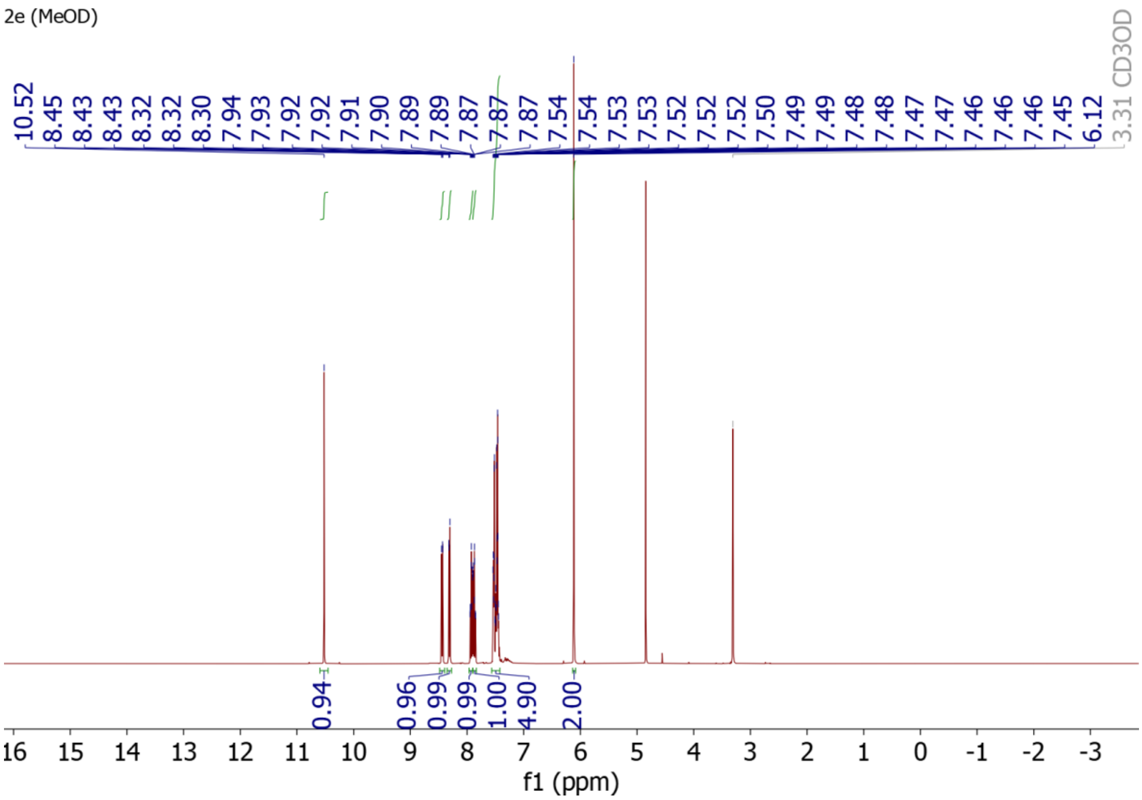

Supplementary Figure 82. ^{1}H NMR spectrum of $\mathbf{4^{S}}$**·HBr**.

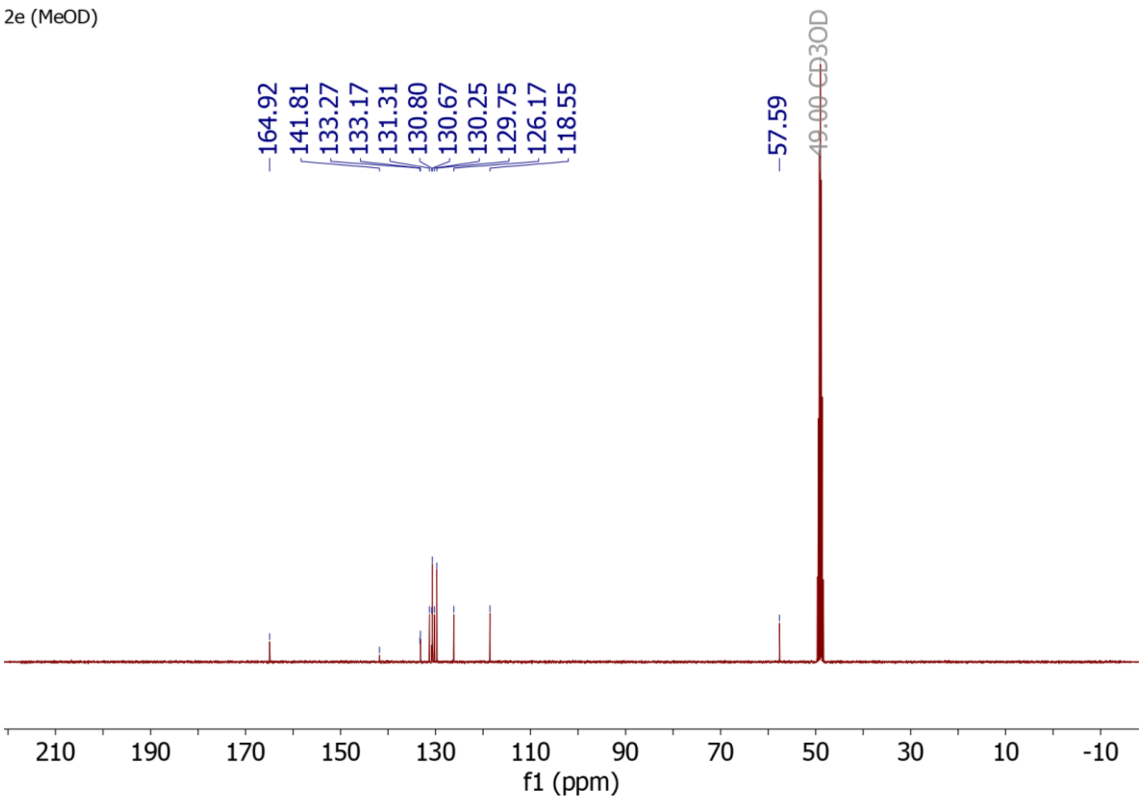

Supplementary Figure 83. ^{13}C NMR spectrum of $\mathbf{4^{S}}$**·HBr**.

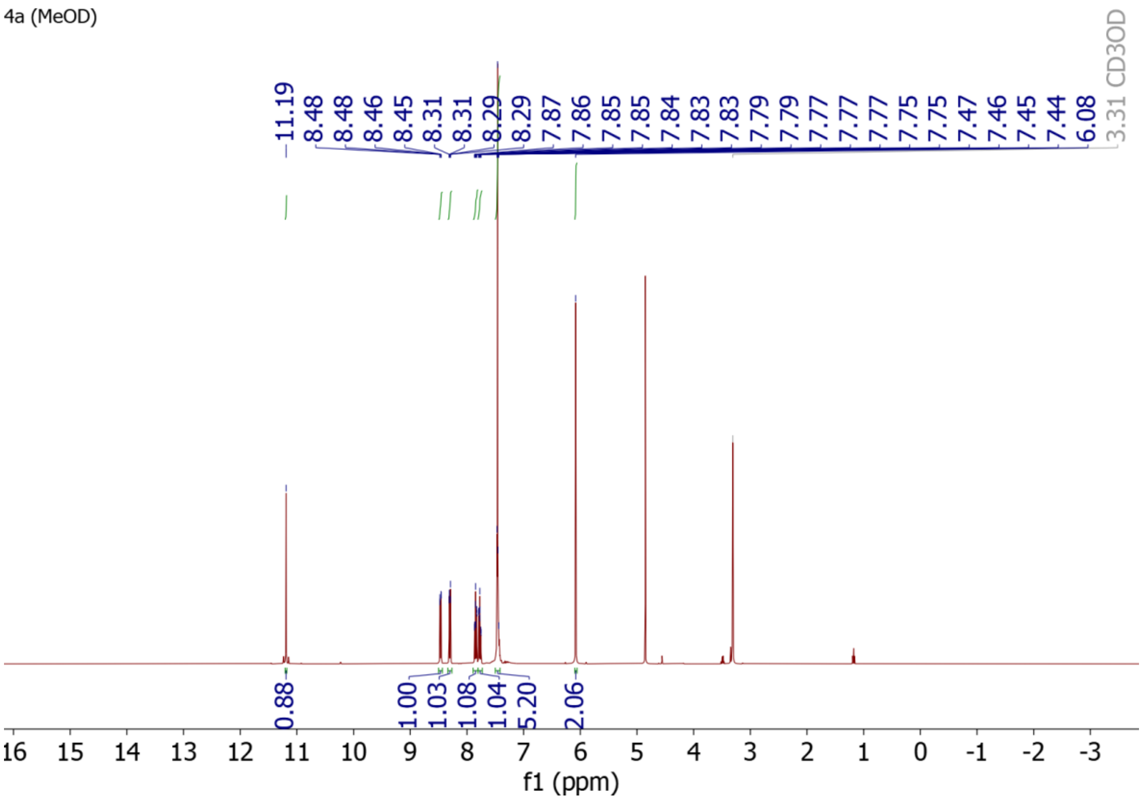

Supplementary Figure 84. ^{1}H NMR spectrum of $\mathbf{4^{Se}{\cdot}HBr}$.

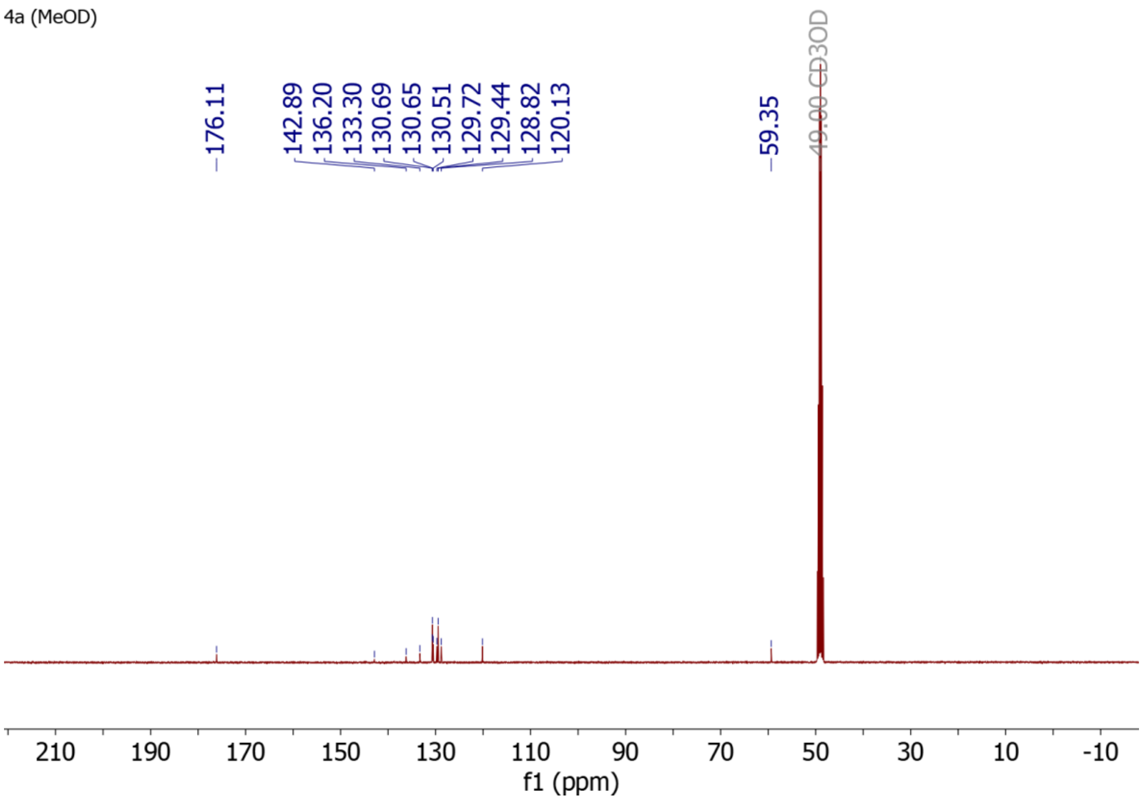

Supplementary Figure 85. ^{13}C NMR spectrum of $\mathbf{4^{Se}{\cdot}HBr}$.

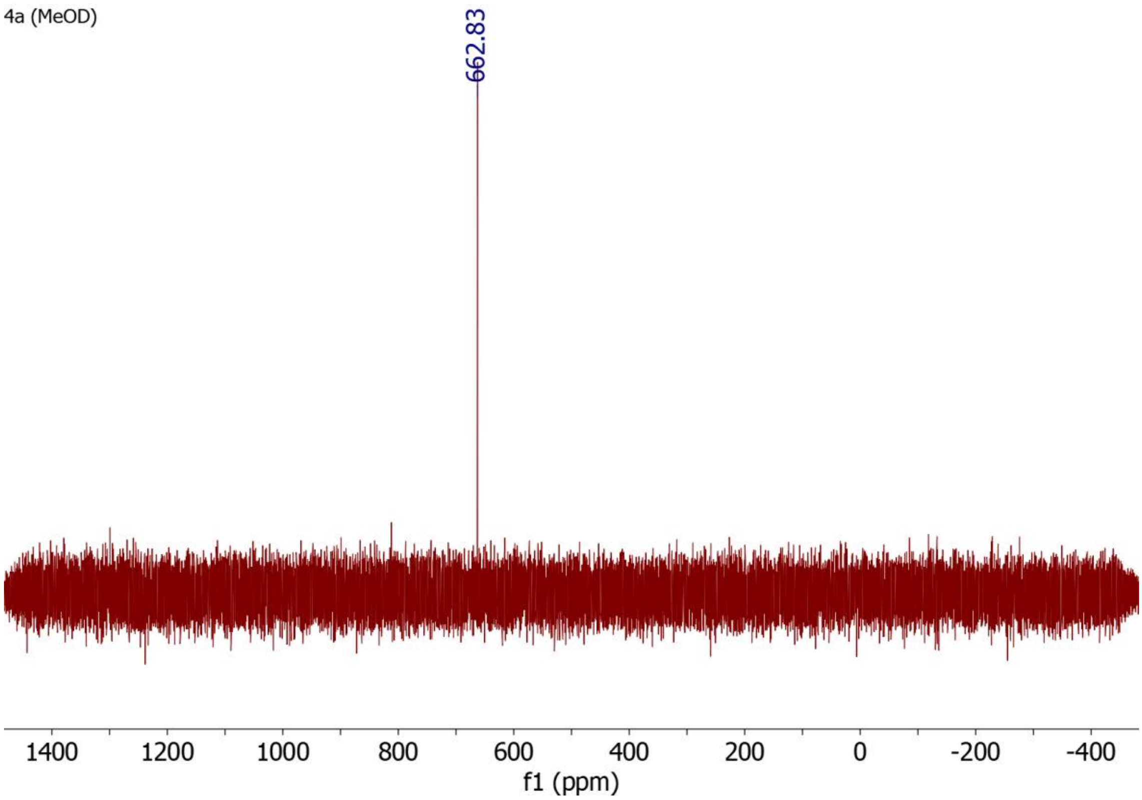

Supplementary Figure 86. ^{77}Se NMR spectrum of **4^{Se}·HBr**.

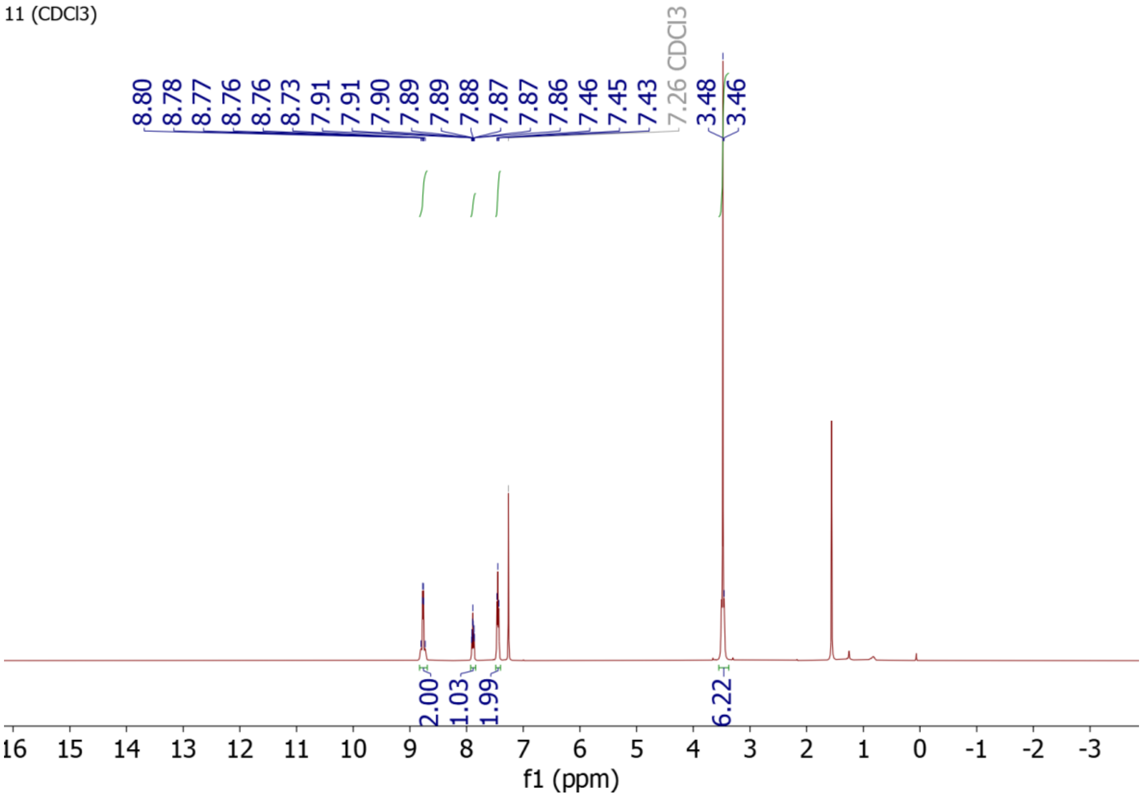

Supplementary Figure 87. ^{1}H NMR spectrum of (dimethylsulfoxide)dichlorido(pyridine)platinum(**II**).

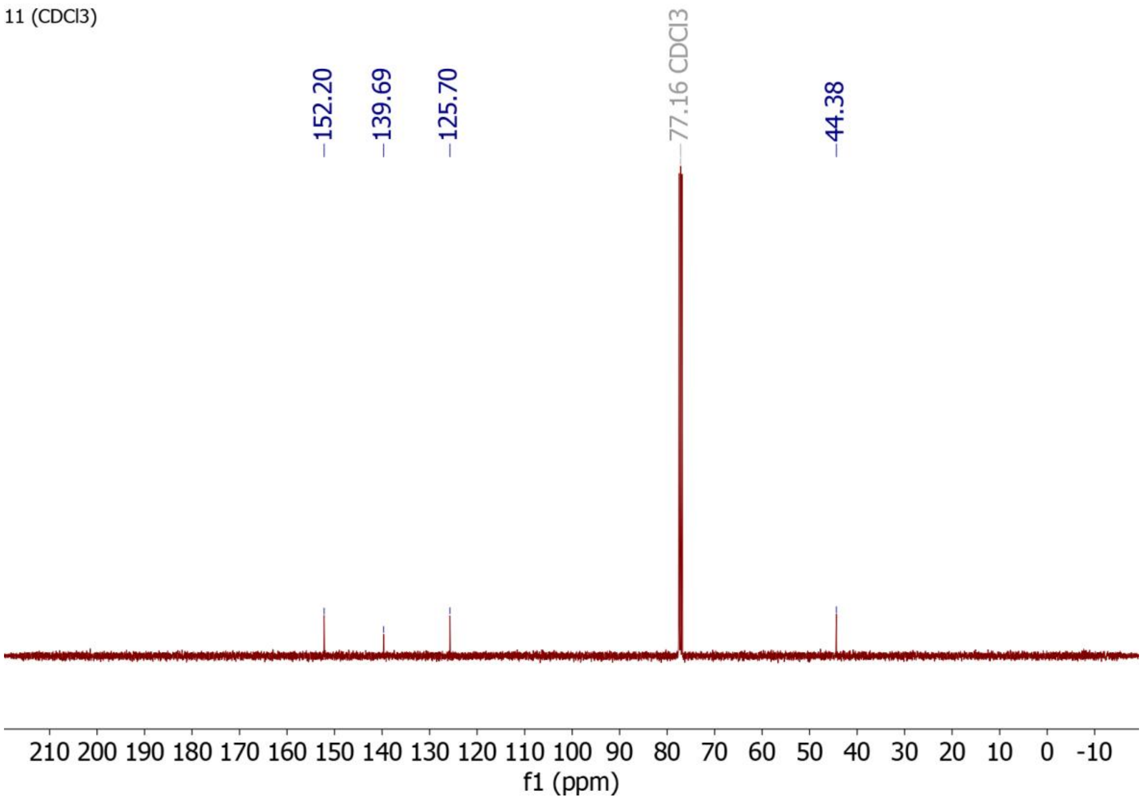

Supplementary Figure 88. ^{13}C NMR spectrum of (dimethylsulfoxide)dichlorido(pyridine)platinum(II).

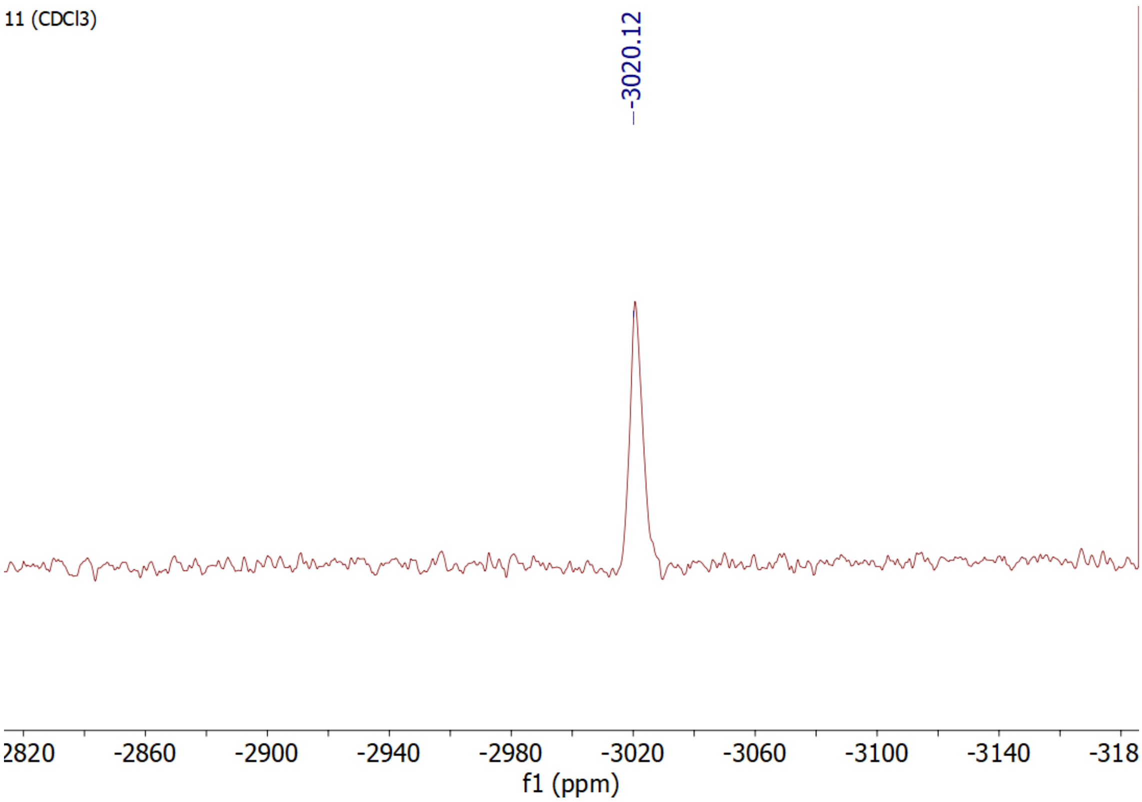

Supplementary Figure 89. ^{195}Pt NMR spectrum of (dimethylsulfoxide)dichlorido(pyridine)platinum(II).

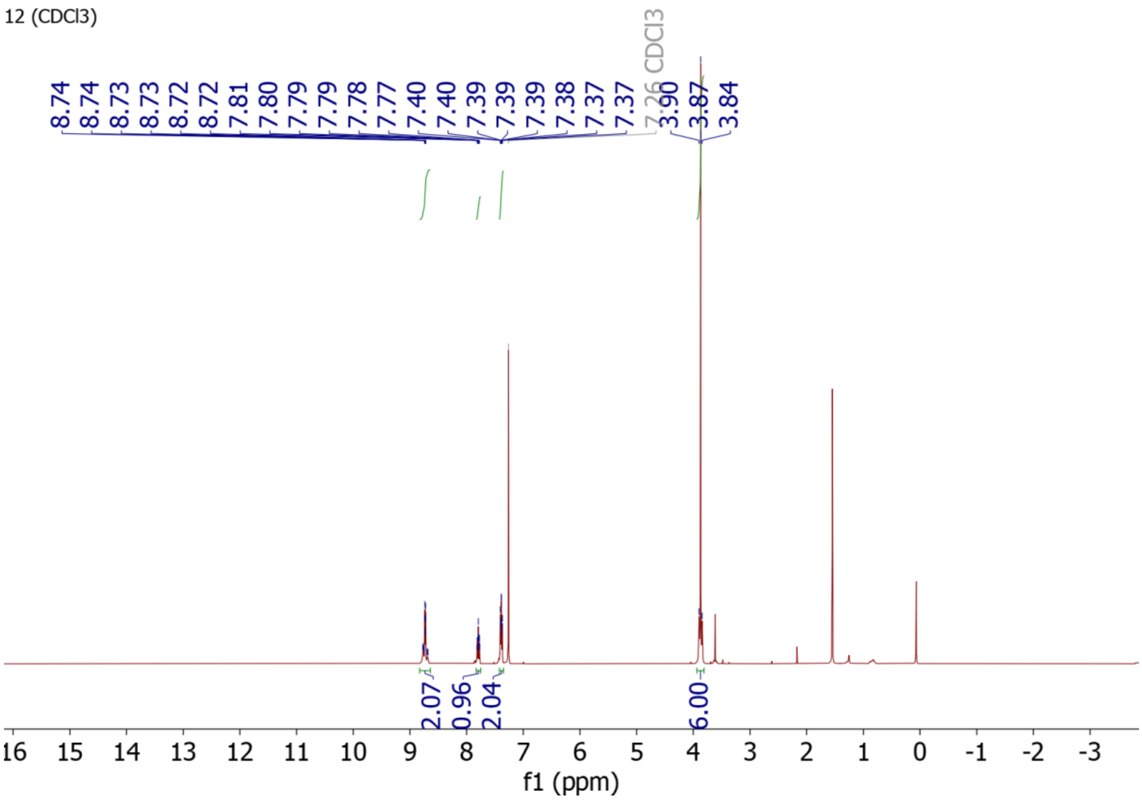

Supplementary Figure 90. ^{1}H NMR spectrum of (dimethylsulfoxide)diiodido(pyridine)platinum(II).

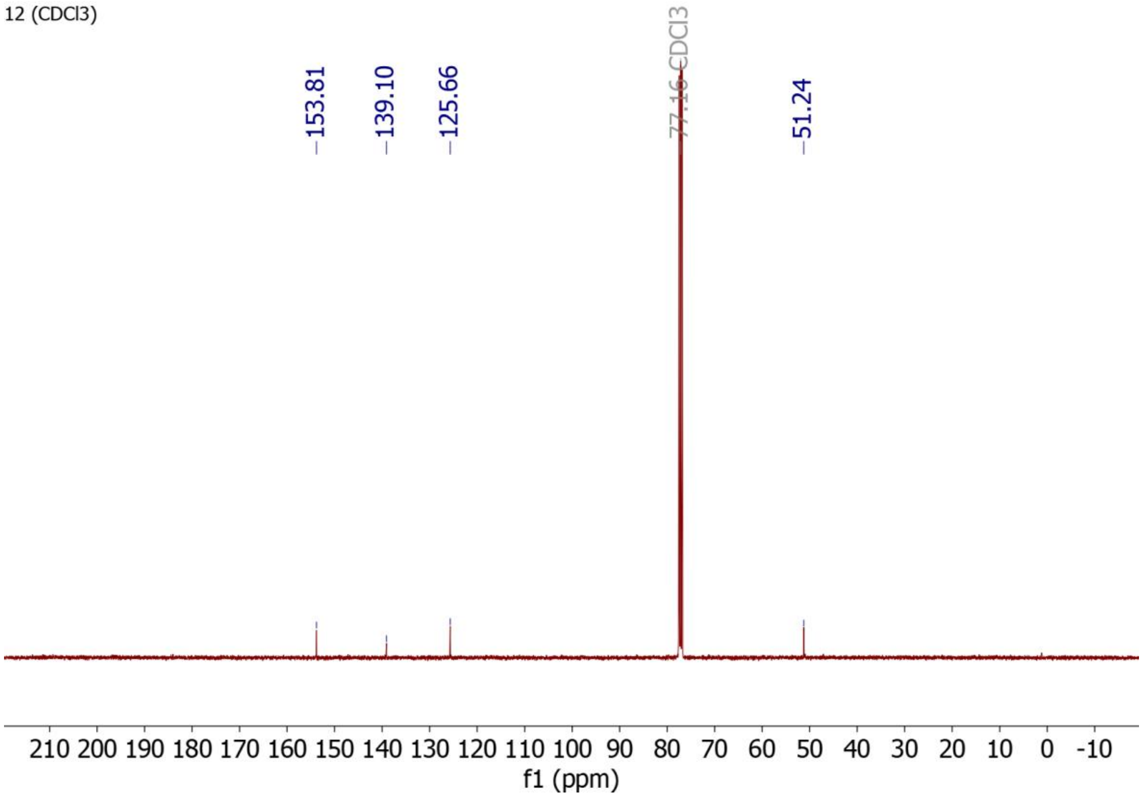

Supplementary Figure 91. ^{13}C NMR spectrum of (dimethylsulfoxide)diiodido(pyridine)platinum(II).

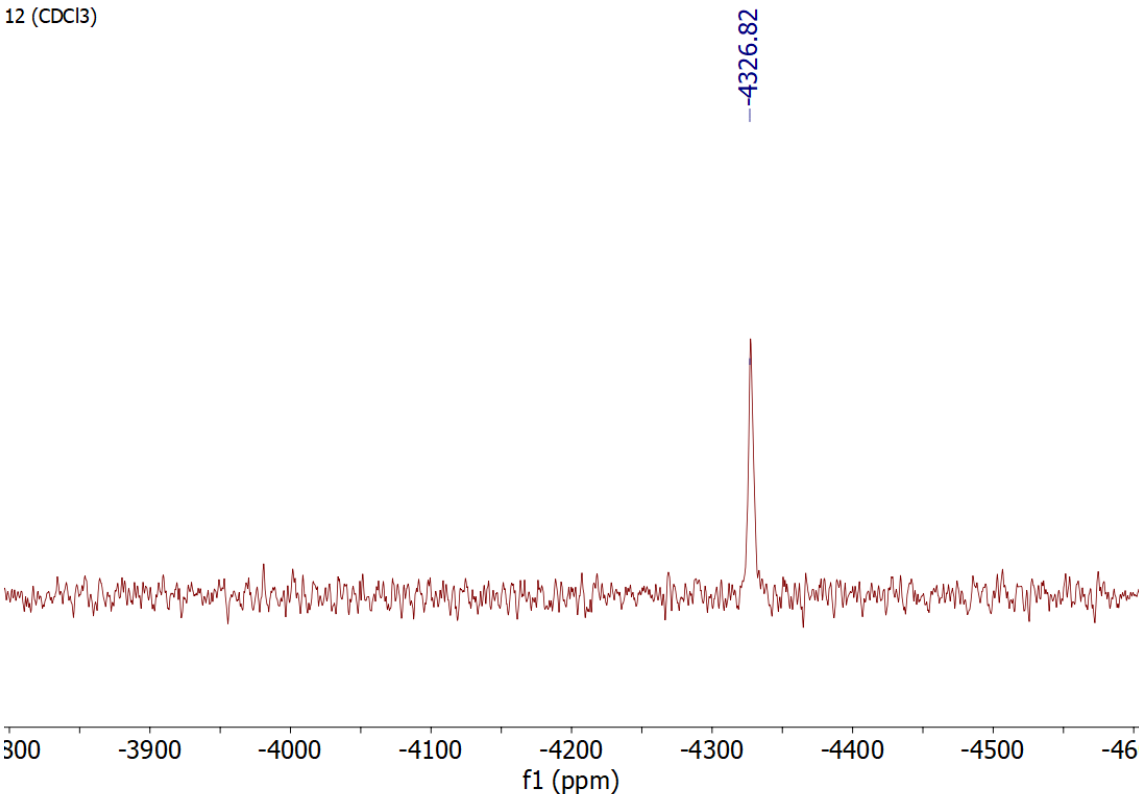

Supplementary Figure 92. ^{195}Pt NMR spectrum of (dimethylsulfoxide)diiodido(pyridine)platinum(II).

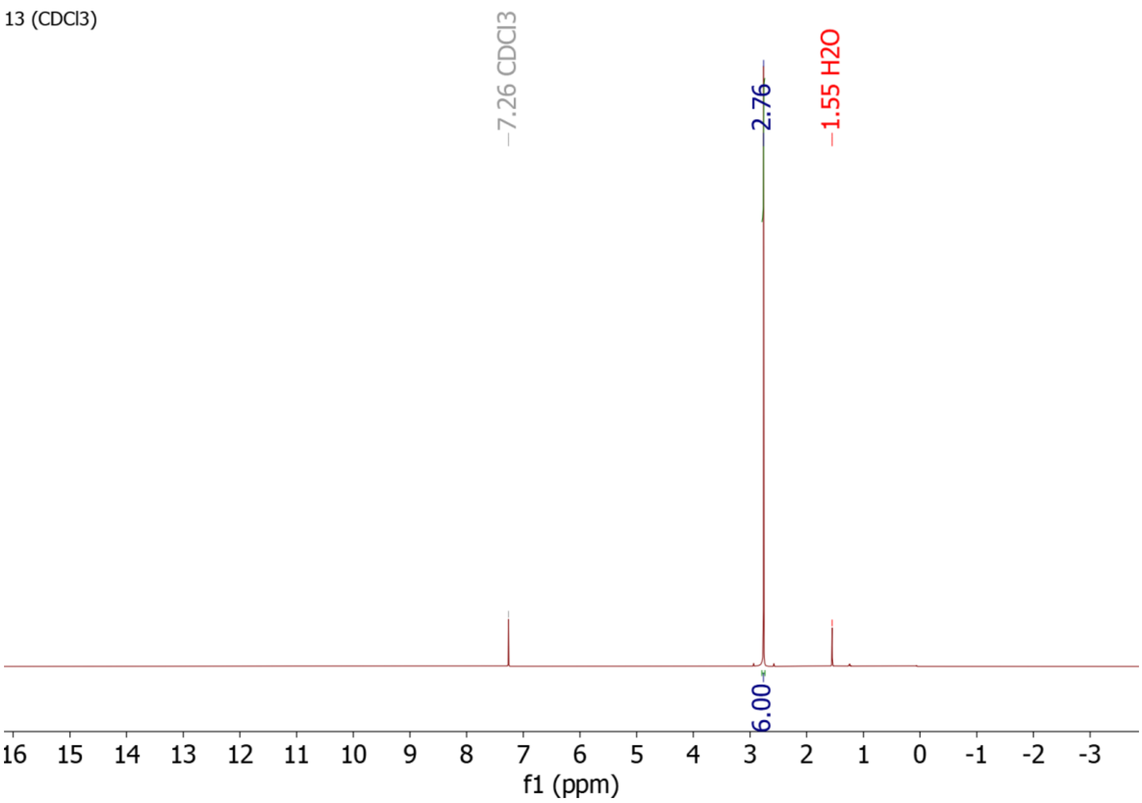

Supplementary Figure 93. ^{1}H NMR spectrum of AuDMSCl.

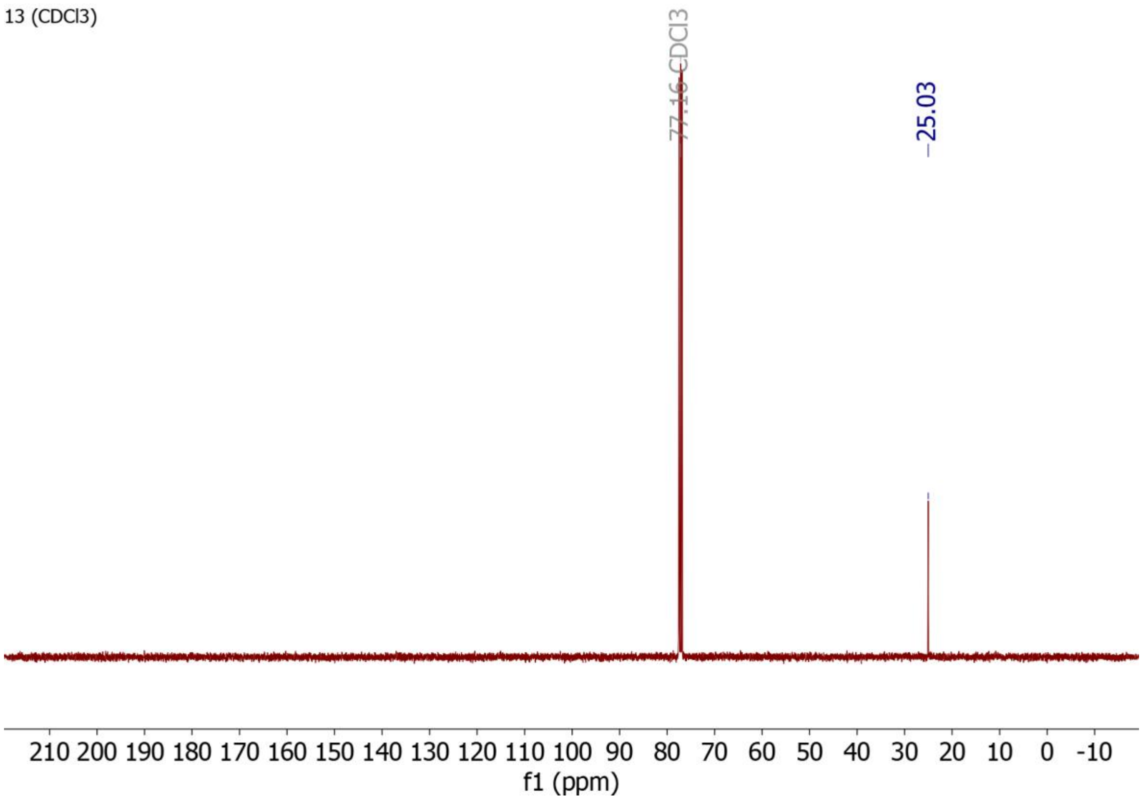

Supplementary Figure 94. ^{13}C NMR spectrum of AuDMSCl.

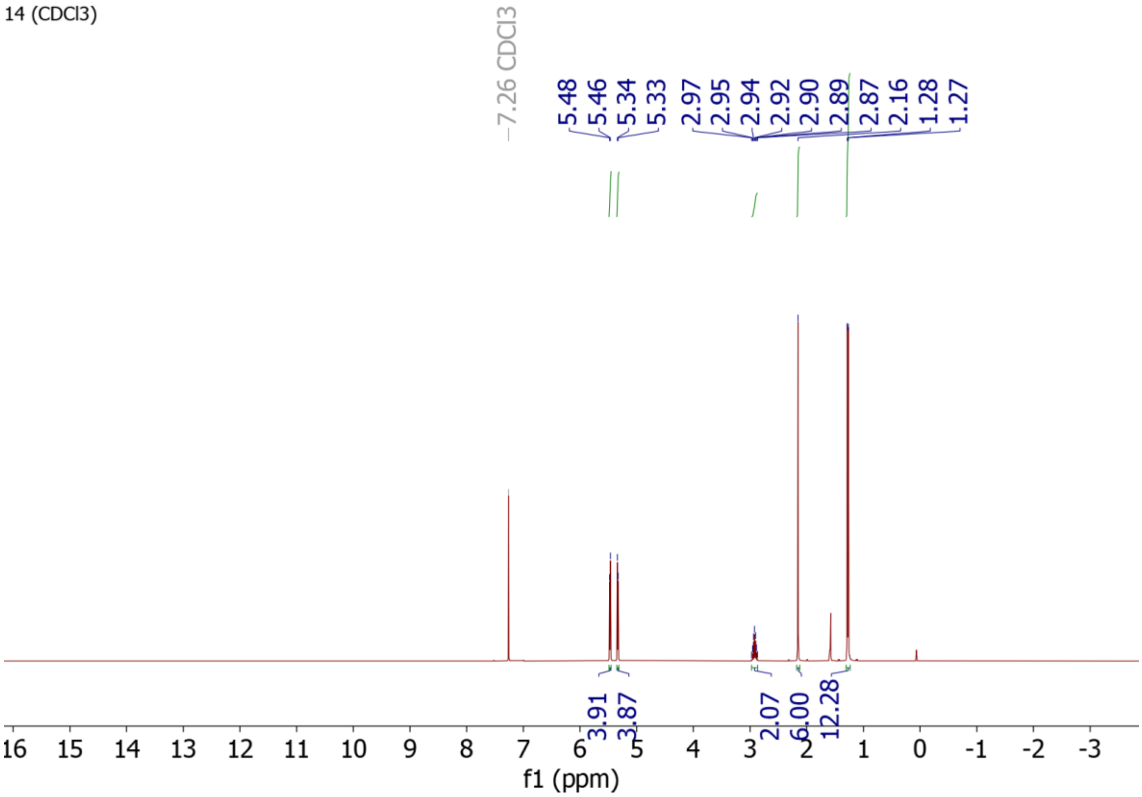

Supplementary Figure 95. ^{1}H NMR spectrum of $[Ru(\eta^6\text{-p-cymene})Cl_2]_2$.

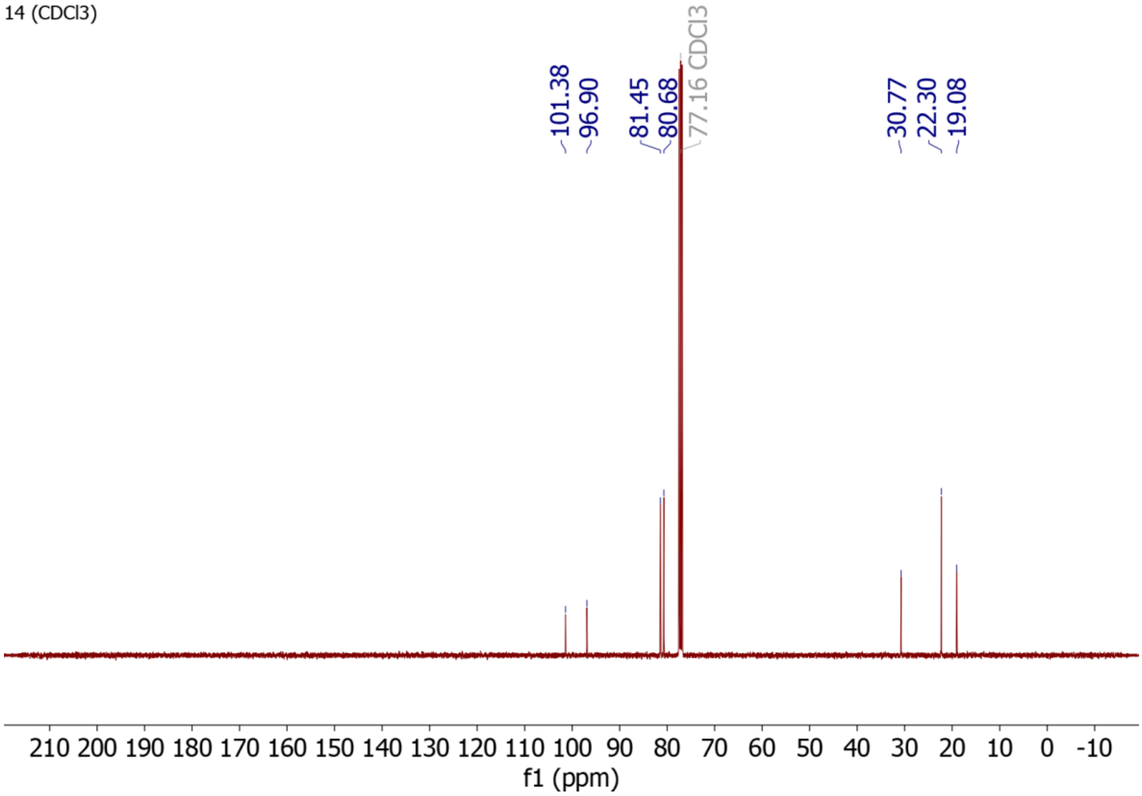

Supplementary Figure 96. ^{13}C NMR spectrum of [Ru(η^6-p-cymene)Cl_2]$_2$.

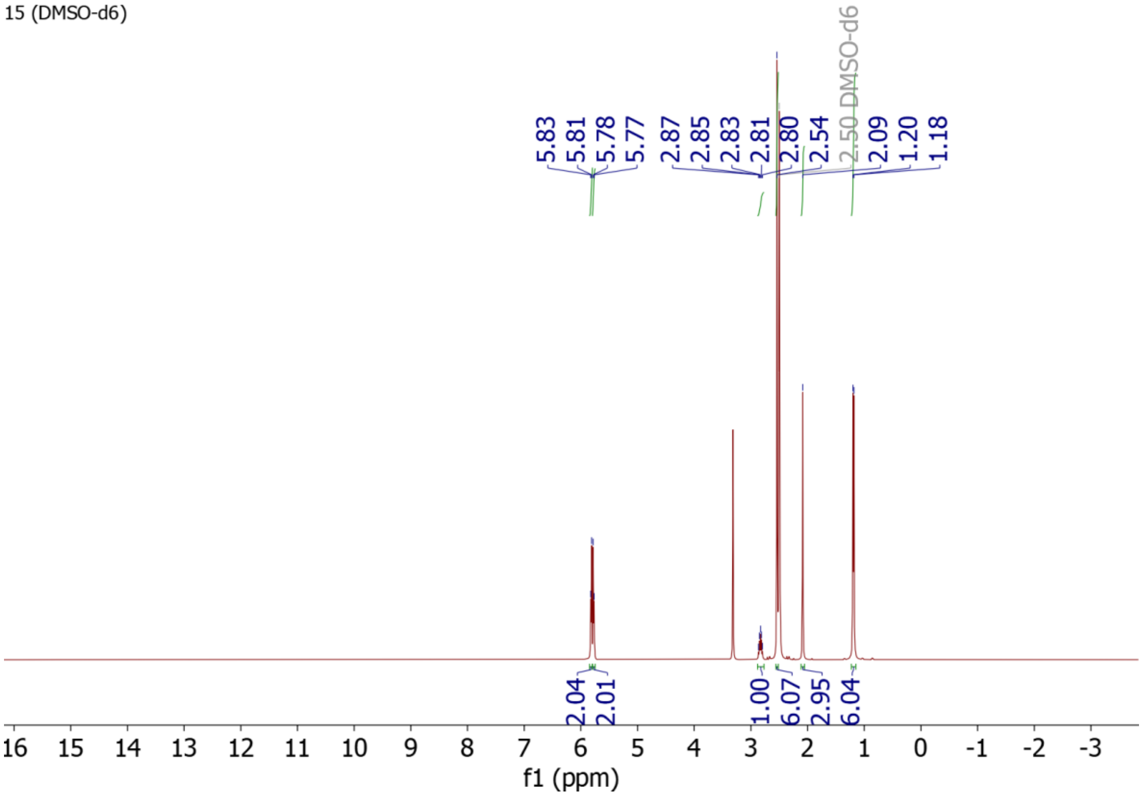

Supplementary Figure 97. ^{1}H NMR spectrum of (η^6-p-cymene)RuCl_2(DMSO).

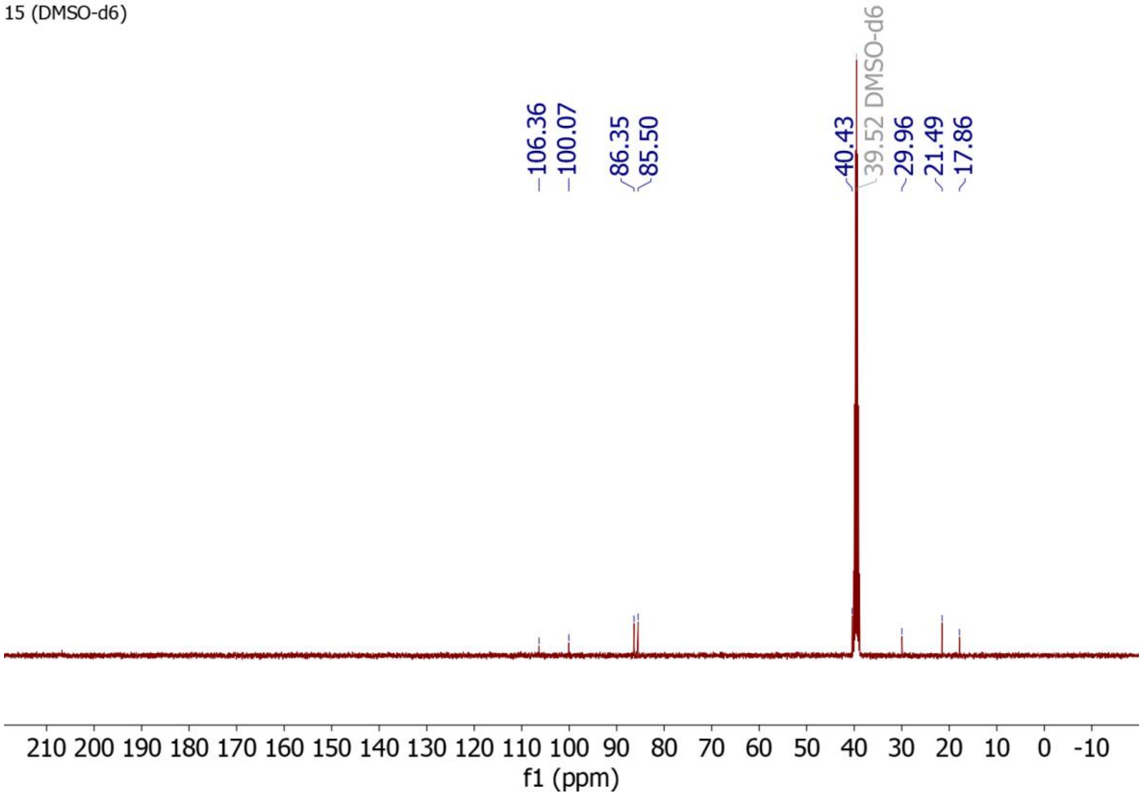

Supplementary Figure 98. ^{13}C NMR spectrum of (η^6-p-cymene)$RuCl_2$(DMSO).

Supplementary references

1 J. C. Mather, J. A. Wyllie, A. Hamilton, T. P. Soares Da Costa and P. J. Barnard, Antibacterial silver and gold complexes of imidazole and 1,2,4-triazole derived N-heterocyclic carbenes, *Dalton Trans.*, 2022, **51**, 12056–12070.

2 V. K. R. Garapati and M. Gravel, Oxazolium Salts as Organocatalysts for the Umpolung of Aldehydes, *Org. Lett.*, 2018, **20**, 6372–6375.

3 A. Kena Diba, C. Noll, M. Richter, M. T. Gieseler and M. Kalesse, Intramolecular Stereoselective Protonation of Aldehyde-Derived Enolates, *Angew Chem Int Ed*, 2010, **49**, 8367–8369.

4 W. J. Kerr, R. J. Mudd and J. A. Brown, Iridium(I) N-Heterocyclic Carbene (NHC)/Phosphine Catalysts for Mild and Chemoselective Hydrogenation Processes, *Chemistry A European J*, 2016, **22**, 4738–4742.

5 S. Shinkai, T. Yamashita, Y. Kusano and O. Manabe, Coenzyme models. 26. Facile oxidation of aldehydes and .alpha.-keto acids by flavin as catalyzed by thiazolium ion and cationic micelle, *J. Org. Chem.*, 1980, **45**, 4947–4952.

6 T. Rehm, M. Rothemund, A. Bär, T. Dietel, R. Kempe, H. Kostrhunova, V. Brabec, J. Kasparkova and R. Schobert, *N*,*N*-Dialkylbenzimidazol-2-ylidene platinum complexes – effects of alkyl residues and ancillary*cis*-ligands on anticancer activity, *Dalton Trans.*, 2018, **47**, 17367–17381.

7 T. Koizumi, N. Bashir, A. R. Kennedy and J. A. Murphy, Diazadithiafulvalenes as electron donor reagents, *J. Chem. Soc., Perkin Trans. 1*, 1999, 3637–3643.

8 V. Amendola, G. Bergamaschi, M. Boiocchi, L. Fabbrizzi and N. Fusco, The solution stability of copper(i) and silver(i) complexes with N-heterocyclic carbenes, *Dalton Trans.*, 2011, **40**, 8367.

9 S. Alwarsh, Y. Xu, S. Y. Qian and M. C. McIntosh, Radical [1,3] Rearrangements of Breslow Intermediates, *Angew Chem Int Ed*, 2016, **55**, 355–358.

10 M. Skander, P. Retailleau, B. Bourrié, L. Schio, P. Mailliet and A. Marinetti, N-heterocyclic carbene-amine Pt(II) complexes, a new chemical space for the development of platinum-based anticancer drugs, *Journal of Medicinal Chemistry*, 2010, **53**, 2146–2154.

11 R. Rubbiani, I. Kitanovic, H. Alborzinia, S. Can, A. Kitanovic, L. A. Onambele, M. Stefanopoulou, Y. Geldmacher, W. S. Sheldrick, G. Wolber, A. Prokop, S. Wölfl and I. Ott, Benzimidazol-2-ylidene gold(I) complexes are thioredoxin reductase inhibitors with multiple antitumor properties, *J. Med. Chem*, 2010, **53**, 8608–8618.

12 L. Oehninger, M. Stefanopoulou, H. Alborzinia, J. Schur, S. Ludewig, K. Namikawa, A. Muñoz-Castro, R. W. Köster, K. Baumann, S. Wölfl, W. S. Sheldrick and I. Ott, Evaluation of arene ruthenium(ii) N-heterocyclic carbene complexes as organometallics interacting with thiol and selenol containing biomolecules, *Dalton Transactions*, 2013, **42**, 1657–1666.

13 G. M. Sheldrick, *SHELXT* – Integrated space-group and crystal-structure determination, *Acta Crystallogr A Found Adv*, 2015, **71**, 3–8.

14 O. V. Dolomanov, L. J. Bourhis, R. J. Gildea, J. A. K. Howard and H. Puschmann, *OLEX2*: a complete structure solution, refinement and analysis program, *J Appl Crystallogr*, 2009, **42**, 339–341.

15 G. M. Sheldrick, Crystal structure refinement with *SHELXL*, *Acta Crystallogr C Struct Chem*, 2015, **71**, 3–8.
16 V. Y. Kukishkin, A. I. Moiseev and E. O. Sidorov, Relationship between the direction of the geometric isomerization of complexes of the [Pt(sulfoxide)(amine)Cl4] type and the melting points of the isomers, *Zh. Obshch. Khim.*, 1989, **59**, 1750–1758.
17 T. N. Hooper, C. P. Butts, M. Green, Mairi. F. Haddow, J. E. McGrady and C. A. Russell, Synthesis, Structure and Reactivity of Stable Homoleptic Gold(I) Alkene Cations, *Chemistry A European J*, 2009, **15**, 12196–12200.
18 (η^6-Hexamethylbenzene)Ruthenium Complexes, *Inorganic Syntheses*, 1982, 74–78.
19 M. Chandra, D. S. Pandey, M. C. Puerta and P. Valerga, A*p*-cymene-ruthenium(II)–DMSO complex, [(η^6-$C_{10}H_{14}$)$RuCl_2$(DMSO)], *Acta Crystallogr E Struct Rep Online*, 2002, **58**, m28–m29.
20 G. Meng, L. Kakalis, S. P. Nolan and M. Szostak, A simple 1H NMR method for determining the σ-donor properties of N-heterocyclic carbenes, *Tetrahedron Letters*, 2019, **60**, 378–381.
21 K. Verlinden, H. Buhl, W. Frank and C. Ganter, Determining the Ligand Properties of N-Heterocyclic Carbenes from ^{77} Se NMR Parameters, *Eur J Inorg Chem*, 2015, **2015**, 2416–2425.
22 A. Liske, K. Verlinden, H. Buhl, K. Schaper and C. Ganter, Determining the π-Acceptor Properties of N-Heterocyclic Carbenes by Measuring the ^{77} Se NMR Chemical Shifts of Their Selenium Adducts, *Organometallics*, 2013, **32**, 5269–5272.
23 OECD, *Test No. 107: Partition Coefficient (n-octanol/water): Shake Flask Method*, OECD, 1995.
24 F. Neese, The ORCA program system, *WIREs Comput Mol Sci*, 2012, **2**, 73–78.
25 P. J. Stephens, F. J. Devlin, C. F. Chabalowski and M. J. Frisch, Ab Initio Calculation of Vibrational Absorption and Circular Dichroism Spectra Using Density Functional Force Fields, *J. Phys. Chem.*, 1994, **98**, 11623–11627.
26 F. Weigend and R. Ahlrichs, Balanced basis sets of split valence, triple zeta valence and quadruple zeta valence quality for H to Rn: Design and assessment of accuracy, *Phys. Chem. Chem. Phys.*, 2005, **7**, 3297.
27 D. A. Pantazis, X.-Y. Chen, C. R. Landis and F. Neese, All-Electron Scalar Relativistic Basis Sets for Third-Row Transition Metal Atoms, *J. Chem. Theory Comput.*, 2008, **4**, 908–919.
28 T. Lu and F. Chen, Multiwfn: A multifunctional wavefunction analyzer, *J Comput Chem*, 2012, **33**, 580–592.
29 J. Mathew and C. H. Suresh, Use of Molecular Electrostatic Potential at the Carbene Carbon as a Simple and Efficient Electronic Parameter of N-heterocyclic Carbenes, *Inorg. Chem.*, 2010, **49**, 4665–4669.
30 M. A. A. Ibrahim and E. M. Z. Telb, σ-Hole and Lone-Pair Hole Interactions in Chalcogen-Containing Complexes: A Comparative Study, *ACS Omega*, 2020, **5**, 21631–21640.
31 M. A. A. Ibrahim, O. A. M. Ahmed, S. El-Taher, J. H. Al-Fahemi, N. A. M. Moussa and H. Moustafa, Cospatial σ-Hole and Lone Pair Interactions of Square-Pyramidal Pentavalent Halogen Compounds with π-Systems: A Quantum Mechanical Study, *ACS Omega*, 2021, **6**, 3319–3329.

32 V. Kundi and J. Ho, Predicting Octanol–Water Partition Coefficients: Are Quantum Mechanical Implicit Solvent Models Better than Empirical Fragment-Based Methods?, *J. Phys. Chem. B*, 2019, **123**, 6810–6822.
33 H. Jacobsen, A. Correa, A. Poater, C. Costabile and L. Cavallo, Understanding the M(NHC) (NHC=N-heterocyclic carbene) bond, *Coordination Chemistry Reviews*, 2009, **253**, 687–703.
34 L. Zhang, Z. Chen, Z. Liu, J. Bu, W. Ma, C. Yan, R. Bai, J. Lin, Q. Zhang, J. Liu, T. Wang and J. Zhang, Efficient electrocatalytic acetylene semihydrogenation by electron–rich metal sites in N–heterocyclic carbene metal complexes, *Nat Commun*, DOI:10.1038/s41467-021-26853-0.
35 I. V. Tetko, J. Gasteiger, R. Todeschini, A. Mauri, D. Livingstone, P. Ertl, V. A. Palyulin, E. V. Radchenko, N. S. Zefirov, A. S. Makarenko, V. Yu. Tanchuk and V. V. Prokopenko, Virtual Computational Chemistry Laboratory – Design and Description, *J Comput Aided Mol Des*, 2005, **19**, 453–463.
36 M. P. Sullivan, M. K. Nieuwoudt, G. A. Bowmaker, N. Y. S. Lam, D. Truong, D. C. Goldstone and C. G. Hartinger, Unexpected arene ligand exchange results in the oxidation of an organoruthenium anticancer agent: the first X-ray structure of a protein–Ru(carbene) adduct, *Chem. Commun.*, 2018, **54**, 6120–6123.
37 G. M. Morris, R. Huey, W. Lindstrom, M. F. Sanner, R. K. Belew, D. S. Goodsell and A. J. Olson, AutoDock4 and AutoDockTools4: Automated docking with selective receptor flexibility, *J Comput Chem*, 2009, **30**, 2785–2791.
38 G. Sciortino, J. Rodríguez-Guerra Pedregal, A. Lledós, E. Garribba and J. Maréchal, Prediction of the interaction of metallic moieties with proteins: An update for protein-ligand docking techniques, *J Comput Chem*, 2018, **39**, 42–51.
39 G. Sciortino, E. Garribba and J.-D. Maréchal, Validation and Applications of Protein–Ligand Docking Approaches Improved for Metalloligands with Multiple Vacant Sites, *Inorg. Chem.*, 2019, **58**, 294–306.
40 D. Santos-Martins, S. Forli, M. J. Ramos and A. J. Olson, AutoDock4 $_{Zn}$: An Improved AutoDock Force Field for Small-Molecule Docking to Zinc Metalloproteins, *J. Chem. Inf. Model.*, 2014, **54**, 2371–2379.
41 G. Bianco, S. Forli, D. S. Goodsell and A. J. Olson, Covalent docking using autodock: Two-point attractor and flexible side chain methods, *Protein Science*, 2016, **25**, 295–301.
42 G.-Y. Lin, Y.-C. Su, Y. L. Huang and K.-Y. Hsin, MESPEUS: a database of metal coordination groups in proteins, *Nucleic Acids Research*, 2024, **52**, D483–D493.
43 H.-C. Tai, R. Brodbeck, J. Kasparkova, N. J. Farrer, V. Brabec, P. J. Sadler and R. J. Deeth, Combined Theoretical and Computational Study of Interstrand DNA Guanine–Guanine Cross-Linking by *trans* -[Pt(pyridine)$_2$] Derived from the Photoactivated Prodrug *trans,trans,trans* -[Pt(N$_3$)$_2$ (OH)$_2$ (pyridine)$_2$], *Inorg. Chem.*, 2012, **51**, 6830–6841.
